# Densimetry for the Quantification of Sorption Phenomena on Nonporous Media Near the Dew Point of Fluid Mixtures

**DOI:** 10.1038/s41598-017-06228-6

**Published:** 2017-07-21

**Authors:** Markus Richter, Mark O. McLinden

**Affiliations:** 1 Thermodynamik, Ruhr-Universität Bochum, D-44780 Bochum, Germany; 2 Applied Chemicals and Materials Division, National Institute of Standards and Technology, Boulder, Colorado 80305 USA

## Abstract

Phase equilibria of fluid mixtures are important in numerous industrial applications and are, thus, a major focus of thermophysical property research. Improved data, particularly along the dew line, are needed to improve model predictions. Here we present experimental results utilizing highly accurate densimetry to quantify the effects of sorption and capillary condensation, which exert a distorting influence on measured properties near the dew line. We investigate the (pressure, density, temperature, composition) behaviour of binary (CH_4_ + C_3_H_8_) and (Ar + CO_2_) mixtures over the temperature range from (248.15 to 273.15) K starting at low pressures and increasing in pressure towards the dew point along isotherms. Three distinct regions are observed: (1) minor sorption effects in micropores at low pressures; (2) capillary condensation followed by wetting in macro-scale surface scratches beginning approximately 2% below the dew-point pressure; (3) bulk condensation. We hypothesize that the true dew point lies within the second region.

## Introduction

Fluid mixtures are important in many scientific and industrial applications. In this context, accurate knowledge about phase equilibria and in particular about the dew line, which separates the homogeneous vapour region from the heterogeneous two-phase (vapour-liquid) region, is essential. The investigation of the phase behaviour of fluid mixtures is a major focus of thermophysical property research for both a fundamental understanding of the behaviour of fluid mixtures and for practical applications including natural gas processing and carbon capture and storage (CCS). For example, equipment in gas pipelines must be protected from damage due to the condensation of the fluid being handled. Therefore, accurate property models are required for the design and operation of industrial processes. Such models are based on accurate experimental data, but there are significant gaps and deficiencies in the existing data sets, which impede the required improvement of mixture models.

Thermophysical property measurements in the vicinity of the dew line of fluid mixtures can be substantially distorted by sorption and condensation effects. This is due to the preferential sorption or condensation of one or more component(s), which changes the vapour composition from that originally loaded into the measuring cell of an experimental apparatus. (The condensed material is enriched in the least volatile component(s), although this can vary with adsorption due to other factors). The study of sorption phenomena is a very active and mature scientific field, with application to gas purification and gas separation, among many other application areas. However, sorption is usually studied on porous media (*i.e*., materials with a large surface area on the microscale) such as activated carbon, zeolites or metal-organic frameworks, but not on the simple metal surfaces (which are often highly polished) existing in property measurement instruments.

It has long been recognized that sorption effects and, in particular the phenomenon of capillary condensation (which is a type of precondensation), can affect and distort property measurements. (The thermophysical property literature often uses the terms “capillary condensation” and “precondensation” interchangeably.) For example, in 1972 Hall and Eubank^1^ considered the effects of adsorbing gases on the Burnett gas-expansion experiment for the determination of density virial coefficients. Kleinrahm and Wagner^2^ in the early 1980s developed a technique to cancel out sorption effects in the measurement of gas densities. Their method equalized the surface area of the two buoyancy sinkers in an Archimedes technique; this equalized the amount of adsorbed material on the two sinkers and thus cancelled the effect on the density measurement. However, since the composition of a mixture can be changed by preferential adsorption of the less-volatile components, the Kleinrahm and Wagner technique is fully effective only for pure fluids. More recently, Richter and Kleinrahm^3^ clearly demonstrated that sorption effects could cause an error in the measurement of the density of complex natural gas mixtures of as much as 0.10%.

There have been very few quantitative treatments of sorption effects in the field of thermophysical property measurements. These include for instance the studies of Mehl and Moldover^4^ in the field of acoustic measurements on pure substances and of Guianvarc’h *et al*.^5^ for mixtures at saturation. Kochsiek^6^ as well as Picard and Fang^7^ have investigated the sorption of atmospheric water vapour on highly polished metal surfaces in the context of mass metrology; however, they considered relative humidities of no more than 93% (*i.e*., conditions removed from the dew point). May *et al*.^8^ measured densities and dew points of light hydrocarbon mixtures, and their work has the most in common with our current study. For their measurements, May *et al*. modified a commercial gravimetric sorption analyser (incorporating a magnetic suspension coupling), which is actually designed to simultaneously measure sorption and density. Such instruments are generally intended to investigate porous media, hence, measurements at the level of accuracy achieved in the present work were not possible.

The few existing studies of sorption effects as applied to thermophysical property measurements are mostly concerned with minimizing these effects rather than quantifying and understanding them. More generally, when studying the vapour phase, experimenters often simply avoid regions being affected by sorption effects or (more commonly) just ignore the distorting impact, which means that inevitable systematic errors are introduced.

Against this background our motivation is to understand and take into account sorption and condensation phenomena. In this context it is not only desirable to recognise the influence of sorption effects close to the dew line but also in the homogeneous vapour phase. Here, we demonstrate a novel experimental technique for the accurate determination of the pressure and density along the dew line of binary mixtures combined with the simultaneous investigation of sorption phenomena. To implement this technique, a gravimetric apparatus (the NIST two-sinker magnetic suspension densimeter^9^) was modified, making it possible to measure the quantity of adsorbed material with microgram resolution. The (*p*, *ρ*, *T*, *x*) behaviour of binary (CH_4_ + C_3_H_8_) and (Ar + CO_2_) mixtures in the temperature range from (248.15 to 273.15) K is investigated, starting at low pressures in the homogeneous vapour region and extending into the two-phase region. We observe three distinct regions as the pressure is increased along an isotherm: (1) At pressures considerably below the dew point, sorption effects on the order of a few tens of micrograms are detected. (2) At pressures approximately 2% below the dew - point pressure, capillary condensation followed by wetting in macro-scale surface scratches occurs at levels up to a few milligrams. (3) Finally, bulk condensation occurs at the level of many milligrams. We hypothesize that the thermodynamic dew point lies within the second region.

## Results

The current work is the launch of a comprehensive project that aims to combine the well-established fields of density measurement (with the magnetic suspension technique^10, 11^) and sorption phenomena with a novel approach. We present the modification of an existing experimental apparatus, how it was used for the sorption studies, and the results obtained for three (CH_4_ + C_3_H_8_) mixtures with nominal CH_4_ mole fractions of 0.27, 0.51, and 0.75 as well as for two (Ar + CO_2_) mixtures with nominal Ar mole fractions of 0.25 and 0.50.

### Modification of gravimetric apparatus

The apparatus we used for the present work is located at the National Institute of Standards and Technology in Boulder (CO), USA; it is a so-called “two-sinker magnetic-suspension densimeter”. The basic measurement technique follows the Archimedes (buoyancy) principle and is described in the Methods section. Briefly, the apparatus weighs (with microgram resolution) two different objects (called sinkers) immersed in the fluid of interest; this is done in a pressure-tight measuring cell at a controlled temperature and pressure. The usual set of sinkers is optimised for highly accurate density measurements, meaning that they have approximately the same mass and same surface area but different volumes (due to different material densities). A large difference in volume maximizes the sensitivity to differences in buoyancy effects, and thus density. For the present work, we replaced the original sinkers with two sinkers of nearly the same mass and same volume, but different surface areas to emphasize sorption effects. Therefore, the accuracy for density measurements was reduced in exchange for a greatly increased sensitivity to the adsorption of gas molecules.

**Figure 1:**
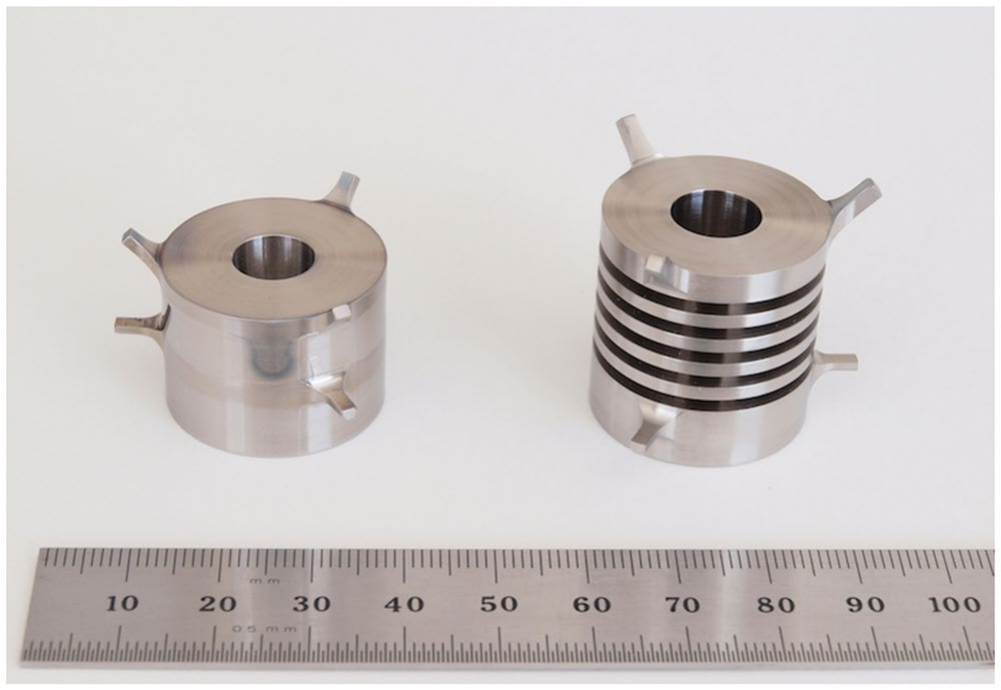
Photograph of the two sinkers for the sorption studies. Left: the “density sinker”, which was located at the top of the measuring cell; right: the “sorption sinker”, which was located at the bottom of the measuring cell. The top set of “pins” on each sinker were engaged by the “lifting fork” of the sinker changing mechanism, and the bottom set of pins engaged a “sinker rest” when it was not being weighed. (See Fig. 6 for a schematic diagram of these sinkers installed in the measuring cell.)

The two sinkers for the sorption studies, as shown in Fig. 1, were both made of titanium. The masses were approximately 42 g, and the volumes were approximately 9.3 cm^3^. The surface areas were approximately 34.1 cm^2^ for the so-called “density sinker” and 76.2 cm^2^ for the so-called “sorption sinker”, which yields a ratio in surface area of 2.24. The surface of the density sinker was polished with abrasives to obtain a smoother finish, whereas the finish of the sorption sinker was left “as machined”. The different surface treatments were intended to increase sorption and condensation effects on the sorption sinker. Although a larger difference in the surface areas would have been more favourable, the design of these sinkers was limited by the existing geometry of the measuring cell.

### Surface characterization of the sinkers

**Figure 2:**
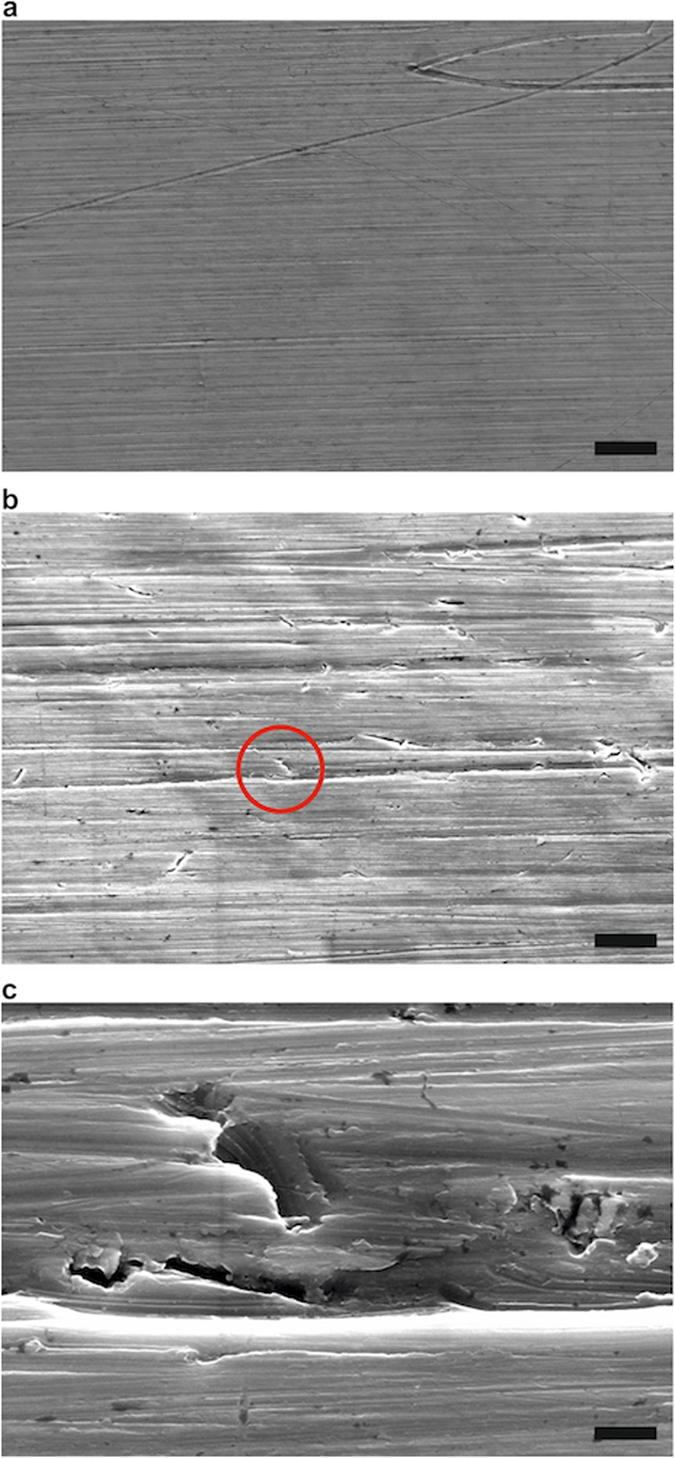
Characterization of the lateral surface of the sinkers using scanning electron microscopy. Images: (**a**) Polished lateral surface of density sinker (scale bar: 20 μm). (**b**) Surface of sorption sinker as machined (scale bar: 20 μm); the red circle indicates a selected imperfection, which is enlarged in (**c**) (scale bar: 2 μm).

**Figure 3:**
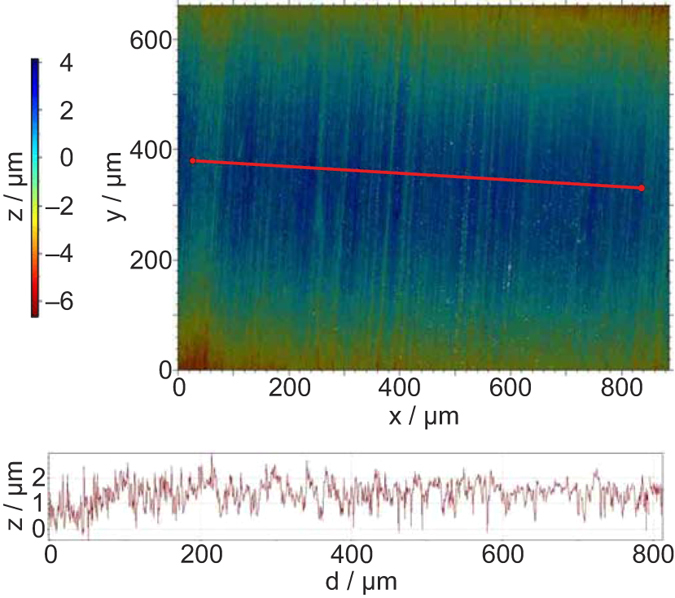
Characterization of the lateral surface of the sorption sinker using white light interferometry. Top panel: image from a white light interferometer with the relative heights depicted by the colour scale; the area imaged was the curved outer circumference of the sinker and, thus, the height falls off at the top and bottom of the image area. Bottom panel: relative height of the surface along the transect indicated by the red line in the top panel.

We characterized the surface characteristics of the sinkers with scanning electron microscopy (SEM) and white light interferometry (WLI) to receive an impression of the microscale nature of the surfaces. As can be seen in the SEM images presented in Fig. 2, the surface of the sorption sinker (panel b) was much rougher than the surface of the density sinker (panel a), which was expected due to the different fabrication procedures. Although the surface of the density sinker looks basically smooth, larger scratches (*e.g*., such as that caused by tools as seen at the upper right of panel a) can be found. The sorption sinker’s surface shows several imperfections, and one of them (red circle in panel b) is enlarged in panel (c). In general, the imperfections seem to result from not-perfectly-sharpened tools, such that material was pulled out of the surface and smeared in differently sized grooves. In Fig. 3, results of the characterization of the lateral surface of the sorption sinker by WLI are shown. These results indicate that the actual surface area, taking into account the increased area due to the machining process (*i.e*., tool marks from the lathe), is approximately a factor of two times larger than the area computed simply by the geometry of the sinker.

Basically, it is obvious that the scratches, gaps and grooves will be excellent nucleation sites for condensation phenomena. While we are aware that the images illustrated in Figs 2 and 3 give no information about the porosity and the detailed texture (pore size, pore size distribution, pore shape), they did confirm to us that sorption/condensation would be enhanced on the sorption sinker relative to the density sinker. Furthermore, the SEM provides information about the surface material, and it is clear that there is no pure titanium (Ti) at the sinkers’ surfaces but mostly titanium oxide (TiO_2_). We cleaned the sinkers with solvents in an ultrasonic bath, but this was not sufficient to completely remove residues of the abrasive paste and other contaminants. The detailed and targeted observation of the surface characteristics of the sinkers as well as more careful cleaning will become important for future work, when linking the experimental results to molecular dynamics simulation is the goal.

The effective size of the molecules considered here (as characterized by the van der Waals diameter) ranged from 0.24 nm for carbon dioxide to 0.43 nm for propane. Thus, a monolayer covering the surface area of the sorption sinker (including the enhancement due to the surface roughness) would be equivalent to a volume of about 3.8 × 10^−6^ cm^3^ and mass of 3.9 μg for carbon dioxide (based on its saturated liquid density at *T* = 293 K); the corresponding values for propane are 6.9 × 10^−6^ cm^3^ and 3.8 μg. The sorption sinker was fabricated primarily on a lathe (*i.e*., a process yielding parallel tool marks); if we approximate the surface as V-shaped grooves with an average depth of 1.0 μm, the volume of these grooves would be 3.8 × 10^−3^ cm^3^ or a mass of 3.9 mg for carbon dioxide and 2.1 mg for propane.

### Isothermal measurements

Tests were carried out along isotherms, starting at a low pressure well inside the homogeneous vapour region; additional sample was incrementally added to the measuring cell, increasing to a final pressure a few percent above the dew point. By weighing the sinkers after each increment, the density of the gas sample was determined as the pressure was increased. A modification of the normal working equation for this type of densimeter also allowed us to quantitatively track the amount of adsorbed or condensed material; details are provided in the Methods section. Measurements along a total of 24 separate isotherms were carried out on the different mixtures, and these data are reported in Supplementary Information. The following discussion presents results for a representative subset of these tests.

**Figure 4:**
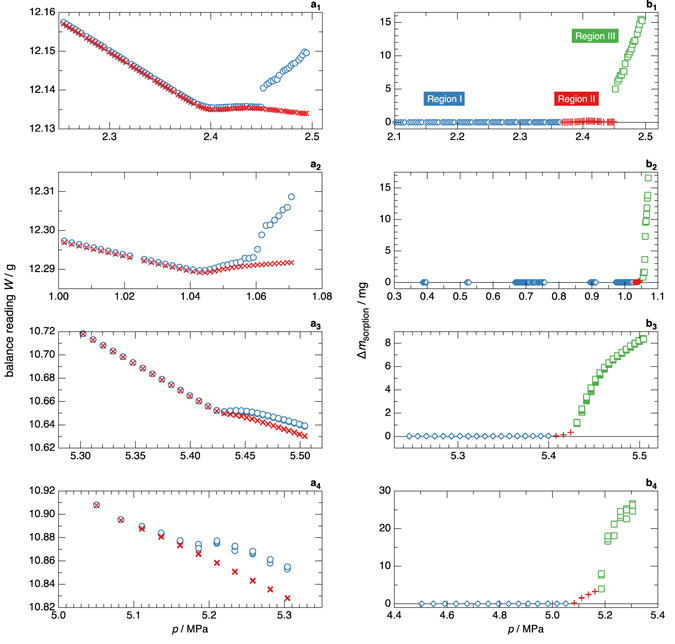
Weighing data and sorption data for several representative mixtures. Panels on the left side of figure plot the balance readings for the two sinkers versus the pressure of the fluid sample; ⚪, sorption sinker;  ×, density sinker; and panels on the right side plot the values of Δ*m*_sorption_ versus the pressure for the corresponding mixtures; ◇, Region I; +, Region II; ◽, Region III. The mixtures are (a_1_ and b_1_): 0.75 CH_4_ + 0.25 C_3_H_8_; (a_2_ and b_2_): 0.50 CH_4_ + 0.50 C_3_H_8_; (a_3_ and b_3_): 0.75 CO_2_ + 0.25 Ar; (a_4_ and b_4_): 0.50 CO_2_ + 0.50 Ar; the temperature was 273.15 K for all tests shown. Regions I, II, and III are defined in the section Isothermal Measurements.

The left side of Fig. 4 shows weighing data (*i.e*., balance readings) for several representative mixtures. (Note that these readings do not represent the total load; because of the limited electronic weighing range of the balance, 80 g of internal counterweights in the balance were used to offset a portion of the load). The weighing data are plotted versus pressure, and three distinct regions were observed for each test, as indicated in the Figure. Differences between the weighings of the two sinkers arise due to differences in the quantity of sample adsorbed or condensed onto the sinker surfaces plus a fixed value of 0.368 mg, because the sorption sinker was slightly heavier than the density sinker. Results for methane/propane mixtures are shown in panels (a_1_) and (a_2_). In the vapour phase at pressures less than about 0.98 · *p*_dew_, which we term “Region I”, the apparent weights of the sinkers (*i.e*., mass minus buoyancy force) decrease with increasing pressure (and, thus, increasing density) due to the increased buoyancy force. In Region II, a steady or increasing sinker weight was observed, despite the increasing fluid density. In Region III, the weight of the sorption sinker rose as material condensed onto it; meanwhile, the weight of the density sinker increased more slowly (panel a_2_), or even continued to decrease (panel a_1_) as the density of the vapour phase decreased due to the preferential condensation of the “heavier”, less volatile, propane onto the sorption sinker and also onto the walls of the measuring cell.

Panels (a_3_) and (a_4_) of Fig. 4 show similar weighing data for two further tests with argon/carbon dioxide mixtures. Here, the results for Regions I and II are qualitatively similar to those seen for the methane/propane mixtures. In Region III, however, the argon/carbon dioxide results are significantly different; rather than seeing an increase in the weight of the sorption sinker, the weight was nearly steady over a small range of pressure and then continued to decrease. We hypothesize that this is a result of the molecular weight of the remaining vapour phase as material adsorbed or condensed. For methane/propane the molar masses of the components are quite different (*M*_CH4_ = 16.0428 g · mol^−1^; *M*_C3H8_ = 44.0956 g · mol^−1^) while for argon/carbon dioxide they are similar (*M*_Ar_ = 39.948 g · mol^−1^; *M*_CO2_ = 44.0098 g · mol^−1^). The density of the gas phase will thus be less sensitive to changes in composition resulting from the preferential condensation of the less-volatile carbon dioxide compared to the methane/propane mixtures.

The results for the same tests presented in panels (a_1_–a_4_) on the left side of Fig. 4 are presented in terms of Δ*m*_sorption_ on the right side of Fig. 4 in panels (b_1_–b_4_); Δ*m*_sorption_ is the mass of material adsorbed or condensed onto sinker 2 (the sorption sinker) minus that adsorbed onto sinker 1. (See the Methods section for a further discussion of this quantity). When interpreted in terms of Δ*m*_sorption_, the methane/propane and argon/carbon dioxide results were similar. In Region I, Δ*m*_sorption_ was very small, on the order of a few tens of micrograms. In Region III, Δ*m*_sorption_ reached values between 8 mg and 28 mg. (The experiment was stopped once Δ*m*_sorption_ reached this order of magnitude). Only for Region II were significant differences observed between different mixtures and even between different tests on a given mixture. Region II covered a pressure range of 0.023 MPa (panel (b_3_)) to 0.11 MPa (panel (b_4_)); in terms of a percentage of the final pressure, the range was 0.4% (panel (b_3_)) to 3.4% (panel (b_1_)). In three of the four tests, there was continuity between the regions. For the test on methane/propane shown in panel (b_1_), however, there was a sudden increase in Δ*m*_sorption_ of nearly 5 mg between consecutive density determinations (which were separated by 30 min), shortly after the Region II/III transition.

### Testing of initial experimental conditions

**Figure 5:**
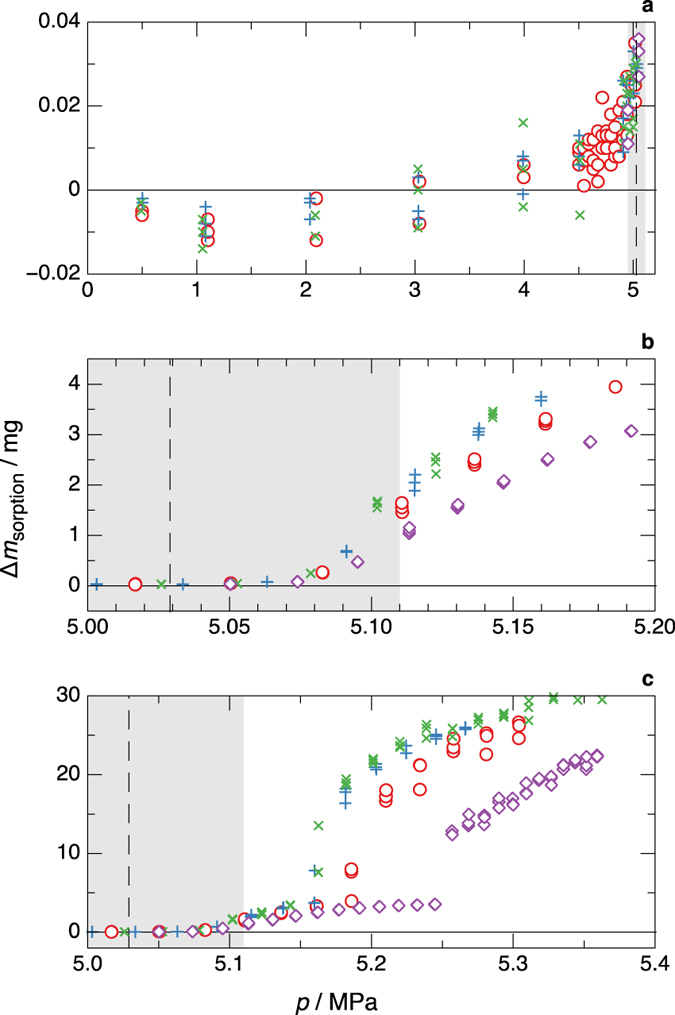
Sorption data for different initial conditions. + , brief purge; ×, brief purge (replicate test); ⚪, 16 h evacuation; ◇, 234 h evacuation. The mixture is 0.50 CO_2_ + 0.50 Ar at *T* = 253.15 K; plotted are the values of Δ*m*_sorption_ versus the pressure of the fluid sample; (**a**) vapour (Region I); (**b**) the Region I/II transition and Region II; (**c**) Region III; the vertical dashed line indicates the dew-point pressure calculated by the EOS-CG model of Gernert and Span^12^, and the shaded area is the uncertainty in the EOS-CG calculation.

We carried out a series of sorption experiments varying the initial conditions for an isothermal test, and these were seen to have a significant influence on the course of sorption. We varied primarily the treatment between consecutive tests; these ranged from a brief evacuation of the measuring cell followed by a purge with fresh sample to an evacuation of the cell lasting from 16 h to 234 h (see the Methods section for further details). These tests were carried out on 0.50 CO_2_ + 0.50 Ar at *T* = 253.15 K. The results are shown in Fig. 5.

Panel (a) of Fig. 5 shows the initial phase of the experiments, *i.e*., Region I. For clarity only a subset of the tests are shown; all of the data are presented in Supplementary Information. The experiments were essentially the “adsorption isotherms” that are well-known in the field of adsorption studies. We observed values of Δ*m*_sorption_ that increased steadily as the pressure increased, as expected for an adsorption process. Negative values of Δ*m*_sorption_ (approximately −10 μg) are observed at the lowest pressures due to the uncertainty in the sinker masses. (The uncertainty of the sinker masses was 20 μg, and the sinker mass could change over the course of the testing due to physical wear or the build-up of surface contamination). The different tests were consistent within ± 8 μg, which was the repeatability of the experiment. But in contrast to an isotherm on a “typical” adsorption sample (such as a zeolite), which might show an adsorbed mass on the order of grams, in our experiments we observe values of Δ*m*_sorption_ that were no more than about 20 μg in Region I, *i.e*., at pressures of less than approximately 0.98 · *p*_dew_.

Panel (b) shows Region II, including the transition between Regions I and II. This transition was quite repeatable (*p* = (5.067 to 5.099) MPa for the different tests), but the rate of change of Δ*m*_sorption_ with pressure varied with the initial conditions. The slope of Δ*m*_sorption_ versus pressure for the 234 h evacuation was 34% of that for the test with a brief purge; the test with a 16 h evacuation time was intermediate. It is well understood that capillary condensation takes place in small pits and scratches (mesopores) with dimensions of (2 to 50) nm. We hypothesize that following the brief purge cycle, at least some of these mesopores retained a small quantity of adsorbed material from the previous test, and that this material served to nucleate subsequent capillary condensation. Following an extended evacuation, on the other hand, these mesopores were completely (or nearly completely) cleared of residual material presenting an activation energy that slowed the sorption process.

The transition from Region II to Region III was also seen to be quite repeatable with the transition pressure varying from 5.130 MPa to 5.184 MPa for the different tests, except for the test following the 234 h evacuation, which was *p* = 5.251 MPa. All ten of the tests showed a relatively rapid initial onset of bulk condensation upon a small change in pressure, followed by a gradual tapering off as the pressure increased. The test following the 234 h evacuation showed a jump of approximately 10 mg in Δ*m*_sorption_; all of the tests showed a similar increase over the course of one or two consecutive increments of added sample.

### Determination of the dew-point pressure

The thermodynamic definition of the dew point corresponds to the onset of condensation, but it does not consider any effects of adsorption or capillary condensation or, in fact, any type of surface interaction. In other words, the strict thermodynamic definition would apply only for a perfectly smooth and planar surface. Thermodynamics also assumes an infinite time to achieve equilibrium, and metastable states (such as a subcooled vapour) are not considered. How then do we determine the thermodynamic dew point in the context of the present measurements? Here we will discuss the results for the Ar/CO_2_ mixture observed in the investigation of initial conditions (*i.e*., Fig. 5) as an example of the influence of sorption phenomena on the determination of dew points. All of our measurement results are presented in Supplementary Information.

As seen in Fig. 5(b), the intersection of Regions I and II at *p* ~ 5.077 MPa is quite consistent, with a standard deviation of 0.009 MPa (0.18%) and total variation of 0.031 MPa or 0.62% in pressure for the different experiments. The value of Δ*m*_sorption_ at the upper limit of Region I was observed to be approximately 20 μg; this would correspond to five molecular layers, which is what one would expect for capillary condensation into nano-sized pores. The pressure across a curved liquid-vapour interface is given by the Kelvin equation (see the Methods section), and the pressure inside a condensed liquid droplet with a radius of 2 nm would be 1.42 times the saturation (*i.e*., dew-point) pressure at that temperature. So clearly, the I/II transition is not the thermodynamic dew point, although it does represent the point at which sorption effects are starting to distort the composition of the sample.

The transition from Region II to III was observed to occur at Δ*m*_sorption_ ~ 4 mg; this roughly corresponds to the quantity of liquid needed to fill the macro-sized grooves resulting from the machining process. For a droplet with a radius of 1 μm, the Kelvin equation gives a pressure 1.0007 · *p*_dew_. But the more likely situation would be that the entire surfaces of the sinkers were wetted, and the Kelvin equation would not be applicable. This suggests that the true dew point lies somewhere within Region II.

The Region II/III intersection at *p* ~ 5.16 MPa in Fig. 5(c) shows a larger standard deviation of 0.023 MPa (0.45%) and a total variation of 0.077 MPa (1.50%) for the different initial conditions. (These statistics were computed based on all ten initial-condition tests; again, all the data are reported in Supplementary Information). This larger variation compared to the I/II intersection may be due to the distorting effects of adsorption and precondensation on the composition of the mixture—the composition of the sample had changed from the original value by the time that bulk condensation started in Region III. In addition to the quantified value of Δ*m*_sorption_ on the sinker, additional material was adsorbed onto the surfaces of the measuring cell, sinker rests, etc. thus multiplying the composition-shifting effect.

We compare the pressures of the I/II transition and the II/III transition with the dew-point pressures calculated with comprehensive mixture models in Table 1. The argon/carbon dioxide mixtures are calculated with the EOS-CG model of Gernert and Span,^12^ and the methane/propane mixtures are calculated with the GERG model of Kunz and Wagner^13^. The calculated dew-point pressures are seen to be different than the experimental pressures, but these differences are near or within the uncertainties of (1.6 to 1.7) % in the EOS-CG model and (1 to 3) % in the GERG model. Our transition pressures are compared to literature data in Supplementary Figs S1 and S2, and here the scatter in the data is seen to be very large, up to 10%. The literature data were measured with a wide range of instruments, and the large scatter in the data contributes to the uncertainty in the mixture models. Also, the precise criteria applied for the determination of the dew point are often not clear in the literature, so that the literature data may be reporting the onset of Region III, *i.e*., a transition that is strongly influenced by sorption effects.

**Table 1:** Summary of the pressures from the isothermal experiments given by the intersection of the adsorbed mass Δ*m*_sorption_ in regions I and II and the intersection of regions II and III; dew-point pressures calculated by an equation of state *p*_dew,EOS_ (the EOS-CG model of Gernert and Span^12^ for the argon/carbon dioxide mixtures and by the GERG model of Kunz and Wagner^13^ for the methane/propane mixtures); and relative differences of the experimental intersections from the model values Δ_*p*,I/II_ and Δ_*p*,II/II_.

*T* K	*p*_I/II_ MPa	*p*_II/III_ MPa	*p*_dew,EOS_ MPa	Δ_*p*,I/II_ %	Δ_*p*,II/II_ %
Argon/carbon dioxide*, x* = (0.25019/0.74981)					
253.149	2.874	2.873	2.848	0.91	0.88
253.149	2.844	2.848	2.848	−0.14	0.00
253.150	2.833	2.855	2.848	−0.53	0.25
253.149	2.859	2.839	2.848	0.39	−0.32
273.150	5.399	5.405	5.419	−0.37	−0.26
273.150	5.413	5.408	5.419	−0.11	−0.20
Argon/carbon dioxide*, x* = (0.49896/0.50104)					
253.150	5.163	5.173	5.029	2.66	2.86
253.150	5.103	5.160	5.029	1.47	2.60
253.149	5.130	5.139	5.029	2.01	2.19
253.150	5.124	5.130	5.029	1.89	2.01
253.150	5.138	5.145	5.029	2.17	2.31
253.149	5.133	5.138	5.029	2.07	2.17
253.150	5.158	5.163	5.029	2.57	2.66
253.149	5.086	5.184	5.029	1.13	3.08
253.149	5.135	5.251	5.029	2.11	4.41
253.150	5.122	5.146	5.029	1.85	2.33
Methane/propane*, x* = (0.26579/0.73421)					
273.148	0.6676	0.6753	0.6728	−0.77	0.37
273.147	0.6665	0.6723	0.6727	−0.92	−0.06
Methane/propane*, x* = (0.50688/0.49312)					
273.146	1.0414	1.0572	1.0628	−2.01	−0.53
Methane/propane*, x* = (0.74977/0.25023)					
248.148	0.9125	0.9555	0.905	0.83	5.58
273.146	2.361	2.403	2.449	−3.59	−1.88
273.146	2.375	2.422	2.449	−3.02	−1.10
273.146	2.411	2.428	2.449	−1.55	−0.86
273.145	2.375	2.410	2.449	−3.02	−1.59

The transition from Region I to Region II is more repeatable because the magnitude of the adsorption in Region I (order of a few tens of micrograms) is much smaller than the precondensation in Region II (order of several milligrams); *i.e*., the distortion of the composition is much smaller. This result indicates that the experiment itself is reproducible, and the variations seen are the result of differing sorption/precondensation effects resulting from different initial conditions or other factors. The magnitude of the effect will be larger or smaller depending on the nature of the experimental apparatus.

Thus, neither the models nor the literature data can provide any definite confirmation of where the thermodynamic dew point lies in terms of Δ*m*_sorption_. In retrospect, it would have been informative to have measured the pure components of our mixtures, since the saturation pressures of argon, carbon dioxide, methane, and propane are all well-known with uncertainties less than 0.02%, although the critical temperatures of argon and methane (150.69 K and 190.56 K, respectively) are below the lower temperature limit of the present densimeter. We will carry out such measurements in future work, where possible. A measurement of the “true” dew point with a density instrument of the type described here thus must await the development of a model that can take sorption effects into account and correct for them.

## Discussion

The thermodynamic definition of the dew point corresponds to the onset of condensation from a homogeneous vapour phase. The existence of adsorption (*i.e*., Region I) distorts the composition of the vapour phase, but for the present results in the present instrument, the magnitude of the adsorption was very small (order of a few tens of μg out of a total sample mass of as much as 18 g). The capillary condensation and wetting occurring in Region II, however, was significant enough to change the mixture composition from that originally loaded into the measuring cell.

The total variation seen the in the pressures observed for the Region I/II and II/III intersections were 0.62% and 1.50%, respectively, for the ten tests of the *T* = 253.15 K isotherm for the Ar/CO_2_ mixture with a nominal composition of 0.50 mole fraction argon. This variation arose from differing initial conditions, and it suggests that uncertainties of this order of magnitude are unavoidable in dew-point experiments. We note, however, that other experimental apparatus or fluid systems may be different.

We hypothesize that the best approximation of the thermodynamic dew-point conditions occur somewhere within Region II. Molecular simulation is expected to give us a qualitative understanding of the sorption processes on an atomic level, which will help us to identify the location of the true dew point within Region II. Insights from molecular modeling are expected to be combined with experimental results to develop an empirical model that would account for, and correct for, sorption effects.

This is the first time that such investigations have been carried out at a microgram-level precision. Nevertheless, the present instrument was not optimal for such investigations; for example, a larger difference in surface area between the sorption and density sinkers would be desirable, and the arrangement of the single filling port into the bottom of the measuring cell could result in condensation of sample in the filling line, and it did not allow flushing sample through the cell. But these results serve as a proof-of-concept for the use of densimetry to investigate sorption phenomena and their effects on the determination of fluid dew points. An instrument optimized for such studies is currently being built at Ruhr-Universität Bochum. Molecular simulation studies and the development of a model to quantify adsorption and precondensation phenomena in the context of thermophysical property measurement instruments in also currently in progress at Ruhr-Universität Bochum. Many more issues remain to be investigated, including further clarification of the effects of the initial conditions, characterizing the sorption behavior of the pure components of the binary mixture under investigation and a more complete characterization of the sinker surfaces.

## Methods

### Apparatus description

The present measurements made use of the two-sinker densimeter at NIST. This instrument has been described in detail by McLinden and Lösch-Will^9^, and only a brief description is given here. This type of instrument applies the Archimedes (buoyancy) principle to provide an absolute determination of the density; it was modified in the present work to investigate sorption phenomena.

**Figure 6:**
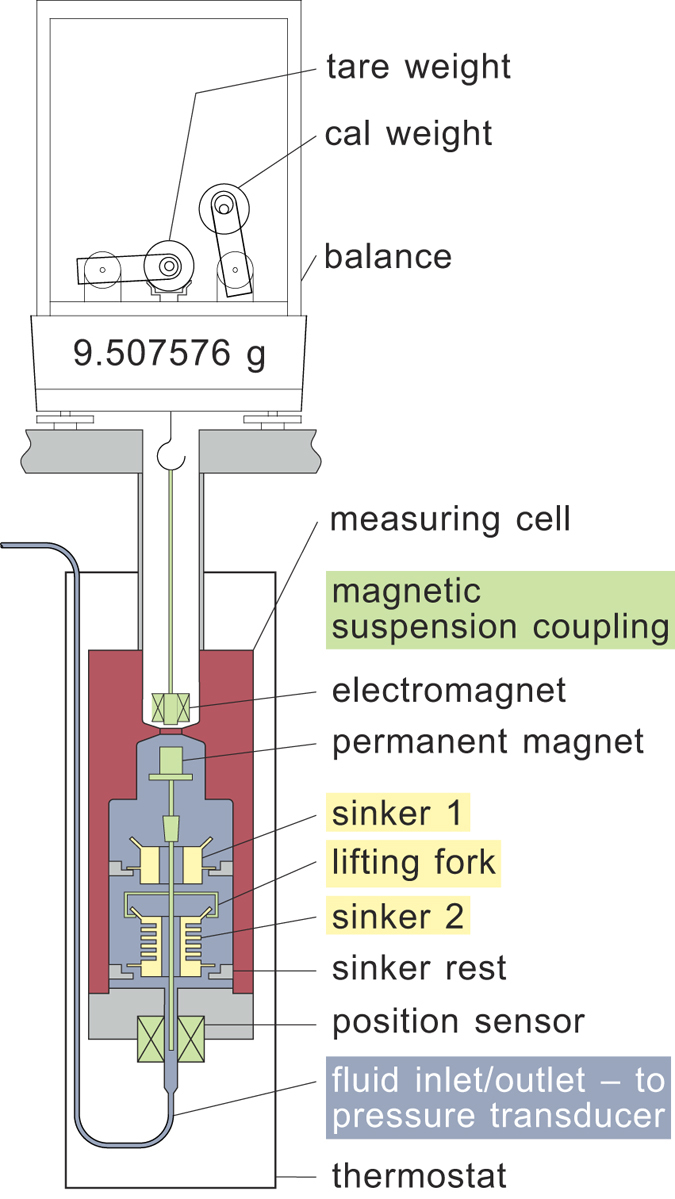
Simplified schematic diagram of the NIST two-sinker densimeter. Sinker 1 is the density sinker, and sinker 2 is the sorption sinker. The sinkers are weighed through a magnetic-suspension coupling comprising an electromagnet, a permanent magnet and a position sensor.

The densimeter is shown schematically in Fig. 6. Two sinkers, which were located inside a pressure-tight measuring cell, were separately weighed while they were immersed in the fluid of interest. A magnetic suspension coupling transmitted the gravity and buoyancy forces on the sinkers to the balance, thus isolating the fluid sample from the balance. The magnetic suspension coupling consisted of an electromagnet with a ferrite core attached to the under-pan hook of the balance (maximum load of 111 g, electronic weighing range of 31 g, and resolution of 1 μg) and (inside the measuring cell) a permanent magnet/“lifting fork” assembly to pick up the sinkers. A position sensor at the bottom of the measuring cell provided a signal to a feedback control circuit which continuously adjusted the current in the electromagnet to maintain the (permanent magnet + lifting fork + sinker) in a stable suspension. Two additional calibration masses (designated as “tare” and “cal”), which were placed directly on the balance pan, were also weighed as part of each measurement. The expanded uncertainty in density in the homogenous vapour phase (*i.e*., Region I) was (52 × 10^−6^ · *ρ*_fluid_ + 0.0007 kg · m^−3^) [9] for the range of temperature and pressure considered here. In Regions II and III the uncertainties in density were larger.

The measuring cell was housed in a multi-layer, vacuum-insulated thermostat. The measuring cell temperature was measured with a 25 ohm standard platinum resistance thermometer and a resistance bridge referenced to a thermostatted standard resistor; the temperature stability was 1.0 mK, and the expanded uncertainty in the temperature (including temperature gradients across the measuring cell) was 6 mK. Pressures were measured with one of two vibrating-quartz-crystal type pressure transducers having full-scale pressure ranges of 2.8 MPa or 6.9 MPa and expanded uncertainties of 0.005% of the full-scale pressure. The transducers and pressure manifold were thermostatted at *T* = 313.15 K to minimize the effects of variations in laboratory temperature and to avoid condensation of the sample gases.

### Sinkers

As indicated in the Results section, the original sinkers in this instrument, which were optimized for density measurements, were replaced with two sinkers fabricated specifically to investigate sorption effects. Table 2 compares these special sinkers, which are designated as the “sorption” and “density” sinkers, with those normally used. The special sinkers were fabricated from titanium bar stock as monolithic objects with no welds. The overall dimensions matched those of the corresponding “normal” sinkers so that modifications to the sinker rests and lifting forks were not required. Fins were cut into the “sorption sinker” to increase the surface area. The mass of the sorption sinker was adjusted to be as close as practical to that of the density sinker. The surface area of the sorption sinker was 2.24 times larger than that of the density sinker, as calculated based on the dimensions on the shop drawings. As discussed in the Results section, the surface roughness of the sorption sinker increased the effective surface area by a further factor of 2.

**Table 2:** Comparison of the special sorption sinkers used in the present testing with the “density sinkers” normally used in the two-sinker densimeter.

Sinker Name	Material	*m*/g	*V*/cm^3^ at *t*_0_ = 20 °C	*A*/cm^2^
Special sinkers for sorption studies				
S1 (“density”)	titanium	42.361466	9.389105	34.1*
S2 (“sorption”)	titanium	42.361834	9.389257	76.4*
Normal sinkers				
S1 (top)	tantalum	60.094633	3.603872	41.5*
S2 (bottom)	titanium	60.075386	13.315284	41.5*

^*^Surface area calculated from dimensions and geometry.

The masses of these sinkers were determined with an expanded uncertainty of 20 μg by a double-substitution weighing design^14^. Their volumes were determined at (*t*_0_ = 20 °C, *p*_0_ = 0.083 MPa) relative to density standards of single-crystal silicon in the hydrostatic comparator at NIST-Boulder; the procedure for these tests is described by McLinden and Splett^15^. The density of the silicon standards was determined at the Physikalisch-Technische Bundesanstalt using the pressure of floatation method^16^. The sinker volumes as a function of temperature were computed using the coefficient of thermal expansion for titanium given by Touloukian *et al*.^17^. The effect of pressure on the volume was computed with the bulk modulus of elasticity measured at NIST by resonant ultrasound spectroscopy on a cylindrical sample fabricated from the same stock of titanium.

### Experimental samples

The gas mixtures were prepared gravimetrically. The components had purities of 0.99999 mole fraction, or higher. We used the materials as received, but we did confirm their purity with our own analysis by gas chromatography/mass spectroscopy. The compositions of the prepared mixtures are given in Supplementary Table S1. They were prepared in aluminium gas cylinders of approximately 6 L or 10 L internal volume. The cylinders were loaded with the sample fluids to pressures corresponding to the dew-point pressure at *T* = 293.15 K. The sample cylinders were continuously heated to *T* > 313 K for the duration of the testing to ensure that only single-phase vapour was present. Further details on preparation of the mixtures are provided by Richter and McLinden^18^.

### Experimental procedures

Measurements were carried out along isotherms. Each isotherm started with the measuring cell evacuated, and sample was incrementally added to a final pressure several per cent above the dew-point pressure. For some of the tests, the evacuated measuring cell was first brought to temperature equilibrium, and then sample was slowly and continuously admitted to the measuring cell through a barely-open valve; in these tests a measurement of the density and sorption/condensation was made every 30 minutes. For the majority of the tests, however, sample was admitted to the measuring cell in discrete increments; this caused the temperature to increase because of adiabatic compression. A density measurement was made after each sample increment (allowing for reequilibration of the temperature and an additional specified time ranging from 30 to 60 minutes).

A single measurement consisted of sequentially weighing the objects in the order: density sinker, sorption sinker, cal weight, tare weight, tare weight (again), cal weight, sorption sinker, density sinker; the weighings were separated by 63 s to 77 s to allow for switching the objects and for the coupling and balance to stabilize. Average values of the two weighings of each of the objects were used in the analysis. The temperature and pressure were recorded between each of the weighings within each density determination and also before the first weighing and after the following weighing; averages of these nine *T* and *p* readings were used in the analysis. This weighing design compensated for any linear drift in the temperature, pressure, or density. A complete measurement sequence required 11.75 minutes. Three such replicates were measured before moving to the next pressure.

Upon completion of an isotherm, the sample in the measuring cell was vented and the cell was evacuated. The time of evacuation was varied from a few minutes to many days to investigate the effects of initial conditions (as presented in the Results section).

### Data analysis

The basic equation for the determination of fluid density with a two-sinker densimeter is

1 $${\rho }_{{\rm{fluid}}}=\frac{({m}_{1}-{m}_{2})-({W}_{1}-{W}_{2})}{({V}_{1}-{V}_{2})},$$

where *m* and *V* are the sinker mass and volume, *W* is the balance reading, and the subscripts refer to the two sinkers. Equation (1) must be expanded to account for magnetic effects of the measuring cell and of the fluid being measured, *i.e*., to account for the so-called “force-transmission error”; this analysis is presented by McLinden *et al*.^19^. This analysis was further extended by McLinden and Richter^20^ to also include a quantitative measure of sample adsorbed or condensed onto the sinkers. The key result was

2 $${\rm{\Delta }}{m}_{{\rm{sorption}}}=(\delta {m}_{2,{\rm{sorp}}}-\delta {m}_{1,{\rm{sorp}}})=\frac{{W}_{2}-{W}_{1}}{\alpha \varphi }+{\rho }_{{\rm{fluid}}}({V}_{2}-{V}_{1})-({m}_{2}-{m}_{1}),$$

where Δ*m*_sorption_ is the mass of material adsorbed onto sinker 2, *δm*_2,sorp_, minus that adsorbed onto sinker 1, *δm*_1,sorp_. It is not possible to solve for *δm*_1,sorp_ and *δm*_2,sorp_ individually. The sinker volumes in equation (2) will be increased by the volume of the adsorbed/condensed material, but this is a small effect; for example, 1 mg of material would change the sinker volume by approximately 0.01%. The *α* is a balance calibration factor; it is derived from the “cal” and “tare” weighings:

3 $$\alpha =\frac{({W}_{{\rm{cal}}}-{W}_{{\rm{tare}}})}{({m}_{{\rm{cal}}}-{m}_{{\rm{tare}}})-{\rho }_{{\rm{air}}}({V}_{{\rm{cal}}}-{V}_{{\rm{tare}}})},$$

where *ρ*_air_ is the density of air in the balance chamber. The “coupling factor” *ϕ* is a measure of the force transmission error. A value of *ϕ* = 1 would indicate a perfect magnetic suspension coupling; the departure of the value of *ϕ* from 1 indicates the magnitude of the force transmission error. For the measurements reported here, *ϕ* varied from 0.999 9997 to 1.000 0037. Note that due to the design of the present sinkers, *V*_1_ ≈ *V*_2_ and *m*_1_ ≈ *m*_2_; furthermore, *α* ≈ 1 and *ϕ* ≈ 1 so that

4 $${\rm{\Delta }}{m}_{{\rm{sorption}}}\approx ({W}_{2}-{W}_{1}).$$

But there are two problems for a complete solution of the above equations. First, because *V*_1_ ≈ *V*_2_ the denominator of equation (1) is approximately zero, and the fluid density is indeterminate. Second, there are not enough equations to determine *ρ*_fluid_, Δ*m*_sorption_, and *ϕ*. The solution is to treat the two-sinker densimeter as a single-sinker densimeter with an added sorption sinker. McLinden *et al*.^19^ provide an analysis of a single-sinker instrument, including methods to determine the *ϕ*.

For a single-sinker densimeter, the fluid density is given by

5 $${\rho }_{{\rm{fluid}}}={\rho }_{{\rm{S}}1}+\frac{1}{\varphi }\frac{{m}_{{\rm{cal}}}-({W}_{{\rm{S}}1}-{W}_{{\rm{cal}}})/\alpha }{{V}_{{\rm{S}}1}},$$

where *ρ*_S1_ is the density of sinker 1 (*i.e*., the density sinker). The coupling factor *ϕ* depends on the specific magnetic susceptibility of the sample fluid, *χ*_s_ and is given by

6 $$\varphi ={\varphi }_{0}+{\varepsilon }_{\rho }\frac{{\chi }_{{\rm{s}}}}{{\chi }_{{\rm{s}}0}}\frac{{\rho }_{{\rm{fluid}}}}{{\rho }_{0}},$$

where *ρ*_0_ = 1000 kg · m^−3^ and *χ*_s0_ = 10^−8^ m^3^ · kg^−1^ are reducing constants, and *ε*_*ρ*_ is an apparatus-specific constant. We previously determined *ε*_*ρ*_ = 52 × 10^−6^ for our two-sinker densimeter^19^. The values of *χ*_s_ were based on Landolt-Börnstein^21^. The value of *ϕ*_0_ was determined by weighings made with the measuring cell under vacuum; this test was regularly carried out to check for possible contamination of the sinkers and to reset the zero of the pressure transducers. Here, *ϕ*_0_ was found to be 1.000 0040. The result is a simultaneous determination of the fluid density, *ρ*_fluid_, and a measure of the adsorbed/condensed mass, Δ*m*_sorption_.

The Kelvin equation gives the pressure across a curved liquid-vapour interface:

7 $$\mathrm{ln}(\frac{p}{{p}_{{\rm{sat}}}})=\frac{2\sigma M}{r\rho RT},$$

where *p* is the pressure inside the droplet, *p*_sat_ is the saturation (dew-point) pressure of the sample fluid, *σ* is the surface tension, *M* is the molar mass, *r* is the radius of the interface, *ρ* is the liquid-phase density, *R* is the molar gas constant, and *T* is the temperature.

### Data availability

All of the data depicted in the figures or discussed in the text are provided in Supplementary Information, which also gives an estimate of experimental uncertainties.

## Electronic supplementary material

Supplementary Information
