# Supplementary material for: Densimetry for the Quantification of Sorption Phenomena on Nonporous Media Near the Dew Point of Fluid Mixtures
Source: Sci Rep. 2017 Jul 21;7:6185. doi: 10.1038/s41598-017-06228-6 (PMC5522488; doi:10.1038/s41598-017-06228-6)
Supplement: Supplementary file 1 — Supplementary Information [file 41598_2017_6228_MOESM1_ESM.pdf]

Densimetry for the Quantification of Sorption Phenomena on Nonporous Media Near the Dew Point of Fluid Mixtures

Markus Richter[1,*] **and Mark O. McLinden**[2]

[1] Thermodynamics, Ruhr-Universität Bochum, D-44780 Bochum, Germany
[2] Applied Chemicals and Materials Division,
National Institute of Standards and Technology, Boulder, Colorado 80305, USA
* m.richter@thermo.rub.de

Supplementary Information

LIST OF FIGURES

Figure S1. Experimental dew point pressures for argon/carbon dioxide mixtures compared to literature data; the zero line is the EOS-CG model. [12]

Figure S2. Experimental dew point pressures for methane/propane mixtures compared to literature data; the zero line is the GERG model. [13]

LIST OF TABLES

Table S1. Gravimetrically determined compositions (mole fraction) and average molar mass of the studied mixtures.

Table S2. Experimental (p, ρ, T, x) data and detailed uncertainty information for isotherms measured on the (0.25019 argon + 0.74981 carbon dioxide) mixture.

Table S3. Experimental (p, ρ, T, x) data and detailed uncertainty information for isotherms measured on the (0.48896 argon + 0.50104 carbon dioxide) mixture.

Table S4. Experimental (p, ρ, T, x) data and detailed uncertainty information for isotherms measured on the (0.26579 methane + 0.73421 propane) mixture.

Table S5. Experimental (p, ρ, T, x) data and detailed uncertainty information for isotherms measured on the (0.50688 methane + 0.49312 propane) mixture.

Table S6. Experimental (p, ρ, T, x) data and detailed uncertainty information for isotherms measured on the (0.74977 methane + 0.25023 propane) mixture.

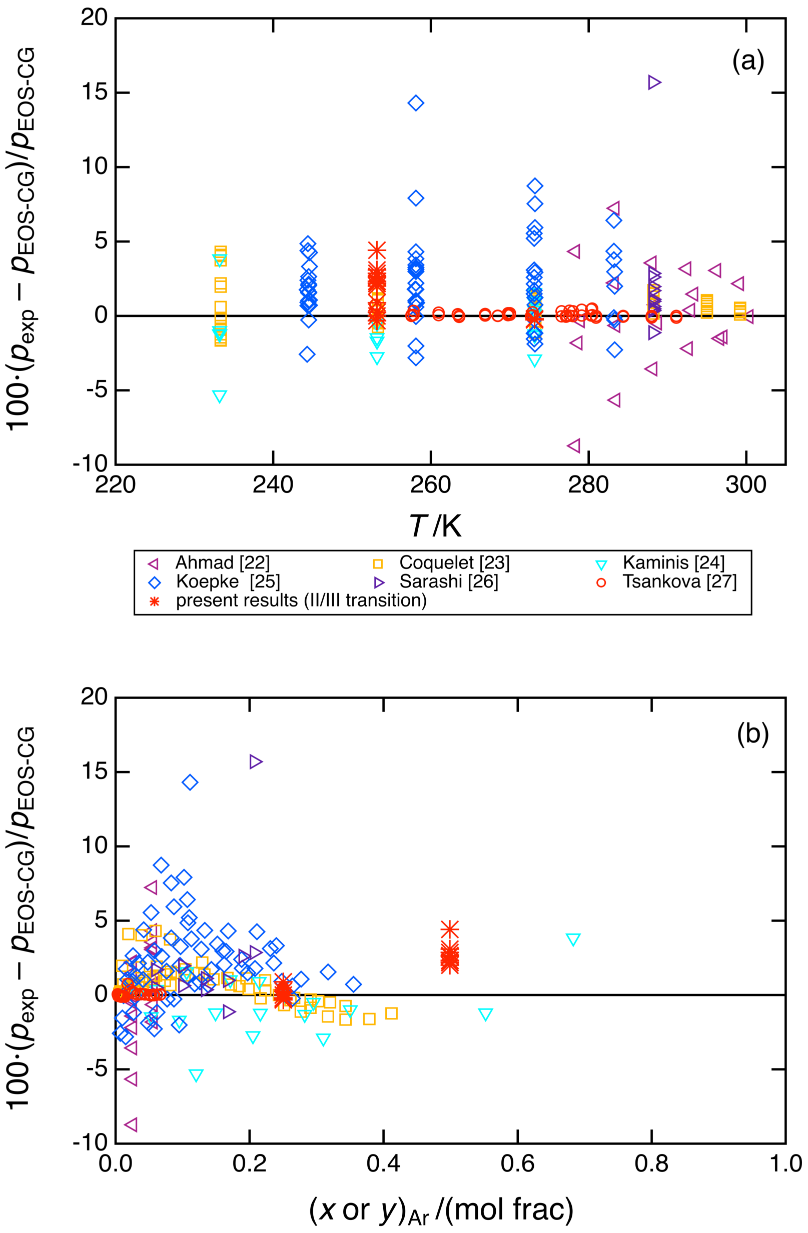

Figure S1. Comparison of present results for dew-point pressures on argon/carbon dioxide mixtures with literature vapor-liquid equilibrium data plotted as relative deviations of experimental dew-point and bubble-point pressures p_{exp} (*i.e.*, p_{dew} or p_{bubble}) from pressures p_{GERG} calculated with the EOS-CG model of Gernert and Span [12]; (a) plotted as a function of temperature; (b) plotted as a function of composition; ✳, present results; other symbols show literature data as indicated in the legend.

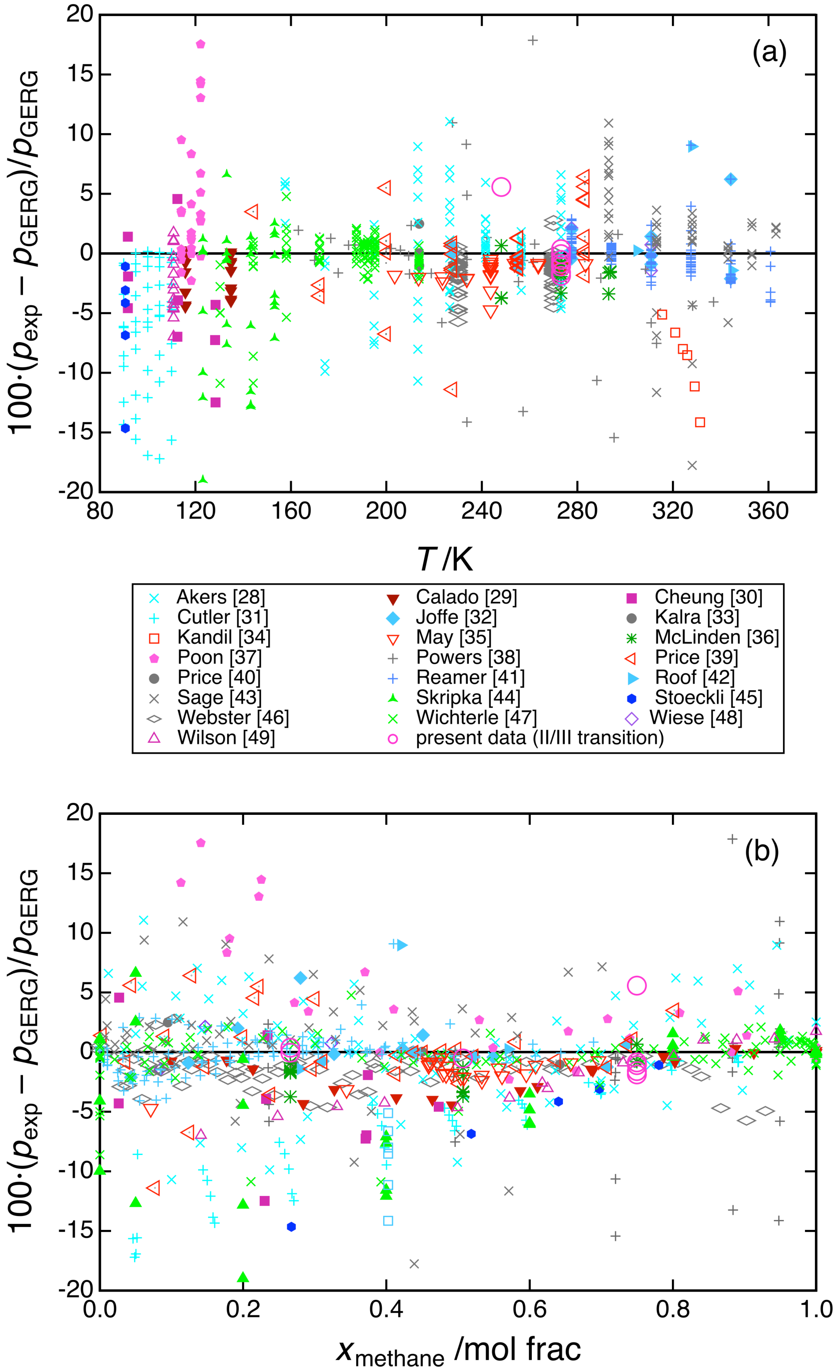

Figure S2. Comparison of present results for dew-point pressures on methane/propane mixtures with literature vapor-liquid equilibrium data plotted as relative deviations of experimental dew-point and bubble-point pressures p_{exp} (*i.e.*, p_{dew} or p_{bubble}) from pressures p_{GERG} calculated with the GERG-2008 equation of state of Kunz and Wagner [13]; (a) plotted as a function of temperature; (b) plotted as a function of composition; ✳, present results; other symbols show literature data as indicated in the legend.

Table S1. Gravimetrically determined compositions (mole fraction) and average molar mass of the studied mixtures. The uncertainty in the compositions was 0.00019 mole fraction, based on McLinden and Richter [20].

Component	Mixture				
	CH_4/C_3H_8 (75/25)	CH_4/C_3H_8 (50/50)	CH_4/C_3H_8 (27/73)	Ar/CO_2 (25/75)	Ar/CO_2 (50/50)
methane	0.74977	0.50688	0.26579		
propane	0.25023	0.49312	0.73421		
argon				0.25019	0.49896
carbon dioxide				0.74981	0.50104
M/g·mol^{-1}	23.0623	29.8761	36.6393	42.9933	41.9830

Tables S2 – S6: Nomenclature.

Heading	Explanation
T	temperature T/K
p	pressure p/MPa
rho	density ρ/kg·m^{-3}
sigma_T	standard deviation in the 9 temperature readings taken over the course of a single density determination; this is a measure of the steadiness of the experimental conditions
sigma_p	standard deviation in the 9 pressure readings taken over the course of a single density determination
sigma_rho	standard deviation in the weighings comprising a single density determination converted to their equivalent effect on density
u(T)	standard (k = 1) uncertainty in temperature
uc(p)	combined standard uncertainty in pressure, including transducer and hydrostatic head effects
u(rho)	standard uncertainty in density–measurement uncertainty only
u(MW)	standard uncertainty in molar mass (related to composition unc.)
u[rho(x)]	standard uncertainty in density including measurement uncertainty and effects of composition uncertainty
U_c/%	relative combined expanded uncertainty in density as defined by: $U_C(\rho) = 2\cdot\left\{ \begin{array}{l} \left[u(\rho)\right]^2 + \left[\left(\frac{\partial\rho}{\partial p}\right)_T u(p)\right]^2 \\ + \left[\left(\frac{\partial\rho}{\partial T}\right)_p u(T)\right]^2 + \left[\frac{\rho}{M} u(M)\right]^2 \end{array} \right\}^{0.5}$
m_sorb	$\Delta m_{\mathrm{sorption}}$/mg
p_trans	pressure transducer used:<br>1 = "high-range" 41.4 MPa full scale [not used in present meas.]<br>2 = "mid-range" 6.89 MPa full scale<br>3 = "low-range" 1.38 MPa full scale
date time	in format: yyyy mm dd hh:mm

Table S2. Experimental (p, rho, T, x) data and detailed uncertainty information for isotherms measured on the (0.25019 argon + 0.74981 carbon dioxide) mixture

```
# component(s): argon                        mole frac:    0.250191
#              CO2                                         0.749809
#
# molar mass:          42.993349
#
# Uncertainty factors [all for standard (k = 1) errors]:
# state point:
#   standard uncertainty in T/K:                           0.0030
#   (p_trans = 1): zero offset in p/kPa                    1.000
#                  relative uncertainty in p/ppm:         26.0
#   (p_trans = 2): zero offset in p/kPa                    0.150
#                  relative uncertainty in p/ppm:         20.0
#   (p_trans = 3): zero offset in p/kPa                    0.030
#                  relative uncertainty in p/ppm:         20.0
#   hydrostatic head, uncertainty in L/V height (m):       0.050
# sinker volume
#   u(V_sinker)/ppm at Tref                               28.0
#   u(V_sinker)_temperature coeff [ppm/K]                  0.375
#   u(V_sinker)_pressure coeff [ppm/MPa]                   0.625
# density [except for V_sinker]
#   u_rho/[kg/m^3]                                         0.00100
# sample
#   sample purity, u(rho) [relative error]:                0.000000
#   gravimetric prep [uncertainty in MW]                   0.000018
#   sorption of sample [uncertainty in MW]                 0.005200
```

T (K)	p (MPa)	rho (kg.m-3)	sigma_T (K)	sigma_p (MPa)	sigma_rho (kg.m-3)	u(T) (K)	u(p) (MPa)	u(rho) (kg.m-3)	u(MW) (g/mol)	u[rho(x)] (kg.m-3)	U_c/% (k = 2)	m_sorb (mg)	p_trans	date	time
# test: Ar_CO2_1511bcd.dat (long evacuation prior to test; 30 min equilibration time; 6 replicates per pressure)															
# chiS [specific magnetic susceptibility] = -0.6015E-08 [m3/kg]															
273.161	1.13078	22.6369	0.0007	0.000006	0.0004	0.0031	0.000152	0.0018	0.00520	0.0027	0.0405	0.004	2	2015 11 16	17:39
273.163	1.13080	22.6281	0.0001	0.000006	0.0023	0.0030	0.000152	0.0034	0.00520	0.0027	0.0481	0.007	2	2015 11 16	17:52
273.151	1.13076	22.6385	0.0005	0.000006	0.0016	0.0030	0.000152	0.0028	0.00520	0.0027	0.0446	0.002	2	2015 11 16	18:56
273.152	1.13075	22.6296	0.0001	0.000004	0.0034	0.0030	0.000152	0.0045	0.00520	0.0027	0.0546	0.003	2	2015 11 16	19:09
273.151	1.13075	22.6393	0.0005	0.000003	0.0007	0.0030	0.000152	0.0019	0.00520	0.0027	0.0411	0.002	2	2015 11 16	19:22
273.149	1.13075	22.6377	0.0003	0.000004	0.0031	0.0030	0.000152	0.0041	0.00520	0.0027	0.0522	0.003	2	2015 11 16	19:35
273.148	1.13075	22.6158	0.0001	0.000006	0.0025	0.0030	0.000152	0.0036	0.00520	0.0027	0.0492	0.003	2	2015 11 16	19:48
273.149	1.13075	22.6384	0.0004	0.000006	0.0003	0.0030	0.000152	0.0017	0.00520	0.0027	0.0403	0.002	2	2015 11 16	20:01
273.149	1.55370	31.8172	0.0011	0.000023	0.0011	0.0032	0.000155	0.0024	0.00520	0.0038	0.0359	-0.003	2	2015 11 16	20:52
273.147	1.55366	31.8184	0.0002	0.000006	0.0005	0.0030	0.000153	0.0021	0.00520	0.0038	0.0349	0.000	2	2015 11 16	21:07
273.149	1.55365	31.8209	0.0008	0.000007	0.0008	0.0031	0.000153	0.0022	0.00520	0.0038	0.0352	0.003	2	2015 11 16	21:20
273.152	1.55365	31.8227	0.0007	0.000005	0.0005	0.0031	0.000153	0.0020	0.00520	0.0038	0.0348	0.006	2	2015 11 16	21:33
273.152	1.55365	31.8219	0.0002	0.000005	0.0006	0.0030	0.000153	0.0021	0.00520	0.0038	0.0350	0.007	2	2015 11 16	21:46
273.151	1.55363	31.8140	0.0006	0.000006	0.0066	0.0031	0.000153	0.0077	0.00520	0.0038	0.0583	0.007	2	2015 11 16	21:59
273.151	2.12153	44.9029	0.0011	0.000027	0.0019	0.0032	0.000158	0.0033	0.00520	0.0054	0.0330	0.002	2	2015 11 16	22:51
273.148	2.12145	44.9063	0.0005	0.000013	0.0030	0.0030	0.000157	0.0043	0.00520	0.0054	0.0351	0.005	2	2015 11 16	23:04
273.148	2.12143	44.9055	0.0003	0.000007	0.0009	0.0030	0.000156	0.0026	0.00520	0.0054	0.0316	0.005	2	2015 11 16	23:17
273.150	2.12143	44.8906	0.0006	0.000007	0.0033	0.0031	0.000156	0.0045	0.00520	0.0054	0.0357	0.011	2	2015 11 16	23:30
273.152	2.12144	44.9018	0.0002	0.000006	0.0013	0.0030	0.000156	0.0029	0.00520	0.0054	0.0321	0.011	2	2015 11 16	23:43
273.151	2.12143	44.8821	0.0004	0.000011	0.0036	0.0030	0.000156	0.0049	0.00520	0.0054	0.0365	0.012	2	2015 11 16	23:56
273.146	3.05899	68.8023	0.0005	0.000011	0.0032	0.0030	0.000163	0.0047	0.00520	0.0083	0.0309	0.004	2	2015 11 17	01:09
273.149	3.05901	68.8150	0.0011	0.000013	0.0006	0.0032	0.000163	0.0031	0.00520	0.0083	0.0291	0.012	2	2015 11 17	01:22
273.153	3.05903	68.8061	0.0007	0.000010	0.0027	0.0031	0.000162	0.0043	0.00520	0.0083	0.0303	0.019	2	2015 11 17	01:35
273.153	3.05901	68.8080	0.0004	0.000018	0.0064	0.0030	0.000163	0.0077	0.00520	0.0083	0.0355	0.015	2	2015 11 17	01:48
273.151	3.05899	68.8010	0.0009	0.000014	0.0010	0.0031	0.000163	0.0033	0.00520	0.0083	0.0292	0.012	2	2015 11 17	02:01
273.148	3.05895	68.8122	0.0004	0.000010	0.0003	0.0030	0.000162	0.0030	0.00520	0.0083	0.0290	0.010	2	2015 11 17	02:14

test: Ar_CO2_1511bcd.dat (continued)

T	p	rho	sigma_T	sigma_p	sigma_rho	u(T)	u(p)	u(rho)	u(MW)	u[rho(x)]	U_c/%	m_sorb	p_trans	date	time
(K)	(MPa)	(kg.m-3)	(K)	(MPa)	(kg.m-3)	(K)	(MPa)	(kg.m-3)	(g/mol)	(kg.m-3)	(k = 2)	(mg)			
273.146	4.00259	96.7983	0.0008	0.000016	0.0008	0.0031	0.000171	0.0039	0.00520	0.0117	0.0283	0.013	2	2015 11 17	03:32
273.150	4.00265	96.7973	0.0012	0.000013	0.0005	0.0032	0.000171	0.0039	0.00520	0.0117	0.0283	0.023	2	2015 11 17	03:45
273.154	4.00268	96.7989	0.0002	0.000007	0.0032	0.0030	0.000170	0.0053	0.00520	0.0117	0.0292	0.020	2	2015 11 17	04:04
273.152	4.00264	96.7975	0.0009	0.000019	0.0044	0.0031	0.000171	0.0062	0.00520	0.0117	0.0300	0.015	2	2015 11 17	04:17
273.149	4.00257	96.7975	0.0007	0.000015	0.0001	0.0031	0.000171	0.0038	0.00520	0.0117	0.0282	0.013	2	2015 11 17	04:30
273.147	4.00256	96.7985	0.0001	0.000009	0.0002	0.0030	0.000171	0.0038	0.00520	0.0117	0.0282	0.014	2	2015 11 17	04:43
273.146	4.54546	115.4138	0.0007	0.000016	0.0006	0.0031	0.000177	0.0044	0.00520	0.0140	0.0282	0.009	2	2015 11 17	05:57
273.149	4.54554	115.4194	0.0013	0.000027	0.0009	0.0033	0.000178	0.0045	0.00520	0.0140	0.0283	0.026	2	2015 11 17	06:10
273.153	4.54559	115.4184	0.0004	0.000013	0.0022	0.0030	0.000176	0.0050	0.00520	0.0140	0.0284	0.035	2	2015 11 17	06:25
273.152	4.54557	115.4170	0.0007	0.000017	0.0048	0.0031	0.000177	0.0069	0.00520	0.0140	0.0296	0.023	2	2015 11 17	06:38
273.150	4.54550	115.4182	0.0009	0.000020	0.0008	0.0031	0.000177	0.0045	0.00520	0.0140	0.0282	0.023	2	2015 11 17	06:51
273.148	4.54545	115.4190	0.0002	0.000013	0.0011	0.0030	0.000176	0.0045	0.00520	0.0140	0.0282	0.023	2	2015 11 17	07:04
273.146	4.98522	132.3678	0.0003	0.000014	0.0014	0.0030	0.000181	0.0051	0.00520	0.0160	0.0283	0.015	2	2015 11 17	08:15
273.149	4.98527	132.3662	0.0009	0.000037	0.0006	0.0031	0.000185	0.0049	0.00520	0.0160	0.0284	0.031	2	2015 11 17	08:28
273.153	4.98536	132.3659	0.0002	0.000008	0.0018	0.0030	0.000181	0.0053	0.00520	0.0160	0.0284	0.037	2	2015 11 17	08:52
273.151	4.98530	132.3601	0.0009	0.000028	0.0015	0.0031	0.000183	0.0051	0.00520	0.0160	0.0284	0.022	2	2015 11 17	09:05
273.148	4.98524	132.3639	0.0005	0.000016	0.0007	0.0030	0.000182	0.0049	0.00520	0.0160	0.0283	0.022	2	2015 11 17	09:18
273.148	4.98525	132.3671	0.0003	0.000030	0.0009	0.0030	0.000183	0.0050	0.00520	0.0160	0.0283	0.028	2	2015 11 17	09:31
273.146	5.29058	145.4138	0.0002	0.000019	0.0021	0.0030	0.000186	0.0057	0.00520	0.0176	0.0286	0.024	2	2015 11 17	10:41
273.148	5.29066	145.4141	0.0010	0.000049	0.0004	0.0032	0.000191	0.0053	0.00520	0.0176	0.0287	0.041	2	2015 11 17	10:54
273.152	5.29079	145.4159	0.0008	0.000029	0.0008	0.0031	0.000187	0.0053	0.00520	0.0176	0.0285	0.022	2	2015 11 17	11:07
273.153	5.29081	145.4182	0.0002	0.000023	0.0018	0.0030	0.000186	0.0056	0.00520	0.0176	0.0286	0.041	2	2015 11 17	11:20
273.151	5.29073	145.4161	0.0007	0.000035	0.0007	0.0031	0.000188	0.0053	0.00520	0.0176	0.0286	0.042	2	2015 11 17	11:33
273.148	5.29065	145.4145	0.0006	0.000022	0.0006	0.0031	0.000186	0.0053	0.00520	0.0176	0.0285	0.029	2	2015 11 17	11:46
273.152	5.34617	147.9173	0.0005	0.000016	0.0010	0.0030	0.000187	0.0054	0.00520	0.0179	0.0285	0.038	2	2015 11 17	12:36
273.150	5.34607	147.9172	0.0007	0.000034	0.0004	0.0031	0.000189	0.0053	0.00520	0.0179	0.0286	0.045	2	2015 11 17	12:49
273.148	5.34602	147.9190	0.0002	0.000019	0.0012	0.0030	0.000187	0.0055	0.00520	0.0179	0.0285	0.041	2	2015 11 17	13:02
273.148	5.34605	147.9186	0.0005	0.000029	0.0003	0.0030	0.000188	0.0053	0.00520	0.0179	0.0285	0.035	2	2015 11 17	13:15
273.150	5.34612	147.9160	0.0006	0.000017	0.0010	0.0031	0.000187	0.0054	0.00520	0.0179	0.0286	0.037	2	2015 11 17	13:28
273.152	5.34615	147.9115	0.0002	0.000018	0.0014	0.0030	0.000187	0.0055	0.00520	0.0179	0.0286	0.037	2	2015 11 17	13:41
273.148	5.39968	150.3759	0.0001	0.000022	0.0007	0.0030	0.000188	0.0054	0.00520	0.0182	0.0286	0.080	2	2015 11 17	14:31
273.149	5.39975	150.3771	0.0006	0.000059	0.0014	0.0031	0.000196	0.0056	0.00520	0.0182	0.0288	0.110	2	2015 11 17	14:44
273.151	5.39981	150.3775	0.0006	0.000031	0.0004	0.0031	0.000189	0.0054	0.00520	0.0182	0.0286	0.075	2	2015 11 17	14:57
273.152	5.39981	150.3757	0.0002	0.000022	0.0003	0.0030	0.000188	0.0054	0.00520	0.0182	0.0285	0.090	2	2015 11 17	15:10
273.151	5.39977	150.3778	0.0006	0.000016	0.0013	0.0031	0.000187	0.0056	0.00520	0.0182	0.0286	0.094	2	2015 11 17	15:23
273.149	5.39969	150.3781	0.0004	0.000046	0.0011	0.0030	0.000192	0.0055	0.00520	0.0182	0.0287	0.095	2	2015 11 17	15:36
273.151	5.44276	152.0329	0.0007	0.000080	0.0210	0.0031	0.000204	0.0225	0.00520	0.0184	0.0408	1.737	2	2015 11 17	16:27
273.149	5.44258	152.0164	0.0004	0.000042	0.0165	0.0030	0.000192	0.0181	0.00520	0.0184	0.0366	1.835	2	2015 11 17	16:40
273.148	5.44259	152.0060	0.0002	0.000051	0.0112	0.0030	0.000194	0.0130	0.00520	0.0184	0.0327	1.952	2	2015 11 17	16:53
273.150	5.44274	151.9960	0.0007	0.000050	0.0074	0.0031	0.000194	0.0097	0.00520	0.0184	0.0306	2.076	2	2015 11 17	17:06
273.152	5.44287	151.9909	0.0004	0.000024	0.0036	0.0030	0.000189	0.0067	0.00520	0.0184	0.0291	2.176	2	2015 11 17	17:19
273.152	5.44285	151.9945	0.0001	0.000024	0.0026	0.0030	0.000189	0.0061	0.00520	0.0184	0.0288	2.241	2	2015 11 17	17:32
273.149	5.47351	152.8225	0.0003	0.000018	0.0259	0.0030	0.000189	0.0273	0.00520	0.0185	0.0452	2.990	2	2015 11 17	18:23
273.149	5.47356	152.8090	0.0003	0.000033	0.0197	0.0030	0.000191	0.0212	0.00520	0.0185	0.0393	3.163	2	2015 11 17	18:36
273.151	5.47367	152.7960	0.0005	0.000021	0.0148	0.0030	0.000189	0.0164	0.00520	0.0185	0.0351	3.326	2	2015 11 17	18:49
273.152	5.47371	152.7888	0.0002	0.000012	0.0097	0.0030	0.000188	0.0116	0.00520	0.0185	0.0316	3.476	2	2015 11 17	19:02
273.151	5.47362	152.7748	0.0003	0.000039	0.0083	0.0030	0.000192	0.0104	0.00520	0.0185	0.0309	3.587	2	2015 11 17	19:15
273.150	5.47344	152.7630	0.0005	0.000054	0.0099	0.0030	0.000195	0.0118	0.00520	0.0185	0.0319	3.654	2	2015 11 17	19:28
273.151	5.50385	153.6285	0.0008	0.000071	0.0220	0.0031	0.000201	0.0235	0.00520	0.0186	0.0415	3.946	2	2015 11 17	20:18
273.148	5.50363	153.6135	0.0004	0.000028	0.0202	0.0030	0.000190	0.0217	0.00520	0.0186	0.0396	3.917	2	2015 11 17	20:31
273.148	5.50366	153.5887	0.0002	0.000042	0.0156	0.0030	0.000193	0.0172	0.00520	0.0186	0.0357	3.892	2	2015 11 17	20:44
273.149	5.50384	153.5788	0.0006	0.000055	0.0130	0.0031	0.000196	0.0148	0.00520	0.0186	0.0339	3.903	2	2015 11 17	20:57
273.151	5.50395	153.5960	0.0003	0.000030	0.0090	0.0030	0.000191	0.0110	0.00520	0.0186	0.0312	3.926	2	2015 11 17	21:10
273.152	5.50391	153.5581	0.0001	0.000028	0.0060	0.0030	0.000190	0.0085	0.00520	0.0186	0.0299	3.945	2	2015 11 17	21:23
273.148	5.53363	154.4360	0.0003	0.000026	0.0241	0.0030	0.000191	0.0255	0.00520	0.0187	0.0432	4.199	2	2015 11 17	22:14
273.148	5.53370	154.4244	0.0004	0.000034	0.0187	0.0030	0.000192	0.0203	0.00520	0.0187	0.0382	4.159	2	2015 11 17	22:27

test: Ar_CO2_1511bcd.dat (continued)

T (K)	p (MPa)	rho (kg.m-3)	sigma_T (K)	sigma_p (MPa)	sigma_rho (kg.m-3)	u(T) (K)	u(p) (MPa)	u(rho) (kg.m-3)	u(MW) (g/mol)	u[rho(x)] (kg.m-3)	U_c/% (k = 2)	m_sorb (mg)	p_trans	date	time
273.150	5.53385	154.4244	0.0006	0.000042	0.0137	0.0031	0.000193	0.0154	0.00520	0.0187	0.0343	4.174	2	2015 11 17	22:40
273.152	5.53392	154.3942	0.0003	0.000012	0.0093	0.0030	0.000189	0.0113	0.00520	0.0187	0.0314	4.188	2	2015 11 17	22:53
273.151	5.53383	154.3959	0.0003	0.000054	0.0068	0.0030	0.000196	0.0091	0.00520	0.0187	0.0303	4.178	2	2015 11 17	23:06
273.150	5.53365	154.3890	0.0005	0.000051	0.0069	0.0030	0.000195	0.0092	0.00520	0.0187	0.0303	4.141	2	2015 11 17	23:19
273.151	5.56396	155.2857	0.0008	0.000055	0.0132	0.0031	0.000197	0.0150	0.00520	0.0188	0.0340	11.787	2	2015 11 18	00:10
273.149	5.56380	155.2658	0.0005	0.000039	0.0126	0.0030	0.000193	0.0144	0.00520	0.0188	0.0335	11.709	2	2015 11 18	00:23
273.148	5.56381	155.2748	0.0001	0.000034	0.0109	0.0030	0.000192	0.0128	0.00520	0.0188	0.0323	9.585	2	2015 11 18	00:36
273.149	5.56397	155.2407	0.0005	0.000051	0.0106	0.0030	0.000196	0.0125	0.00520	0.0188	0.0323	10.021	2	2015 11 18	00:49
273.151	5.56412	155.2299	0.0004	0.000025	0.0077	0.0030	0.000191	0.0099	0.00520	0.0188	0.0306	10.554	2	2015 11 18	01:02
273.152	5.56412	155.2220	0.0001	0.000022	0.0043	0.0030	0.000191	0.0073	0.00520	0.0188	0.0293	8.096	2	2015 11 18	01:15
273.149	5.59339	156.1258	0.0004	0.000045	0.0137	0.0030	0.000195	0.0155	0.00520	0.0189	0.0342	5.104	2	2015 11 18	02:06
273.148	5.59341	156.1173	0.0002	0.000047	0.0108	0.0030	0.000196	0.0127	0.00520	0.0189	0.0323	5.072	2	2015 11 18	02:19
273.150	5.59359	156.1046	0.0007	0.000058	0.0098	0.0031	0.000198	0.0118	0.00520	0.0189	0.0318	5.023	2	2015 11 18	02:32
273.152	5.59373	156.0836	0.0004	0.000025	0.0054	0.0030	0.000191	0.0080	0.00520	0.0189	0.0297	5.009	2	2015 11 18	02:45
273.152	5.59366	156.0791	0.0003	0.000041	0.0024	0.0030	0.000194	0.0061	0.00520	0.0189	0.0290	4.984	2	2015 11 18	02:58
273.150	5.59348	156.0780	0.0006	0.000057	0.0024	0.0031	0.000198	0.0062	0.00520	0.0189	0.0291	4.953	2	2015 11 18	03:11
273.150	5.62291	156.9724	0.0005	0.000060	0.0131	0.0030	0.000199	0.0149	0.00520	0.0190	0.0338	11.235	2	2015 11 18	04:01
273.149	5.62283	156.9611	0.0001	0.000015	0.0090	0.0030	0.000191	0.0111	0.00520	0.0190	0.0312	11.654	2	2015 11 18	04:14
273.149	5.62288	156.9517	0.0003	0.000038	0.0091	0.0030	0.000194	0.0112	0.00520	0.0190	0.0313	11.936	2	2015 11 18	04:27
273.150	5.62298	156.9515	0.0004	0.000024	0.0077	0.0030	0.000192	0.0099	0.00520	0.0190	0.0306	7.626	2	2015 11 18	04:40
273.151	5.62297	156.9311	0.0001	0.000022	0.0046	0.0030	0.000192	0.0075	0.00520	0.0190	0.0294	8.109	2	2015 11 18	04:53
273.150	5.62284	156.9382	0.0004	0.000043	0.0031	0.0030	0.000195	0.0065	0.00520	0.0190	0.0291	8.264	2	2015 11 18	05:06
273.150	5.65192	158.0687	0.0004	0.000044	0.0105	0.0030	0.000196	0.0125	0.00520	0.0191	0.0321	9.254	2	2015 11 18	05:57
273.149	5.65185	158.0539	0.0001	0.000013	0.0087	0.0030	0.000191	0.0108	0.00520	0.0191	0.0310	9.255	2	2015 11 18	06:10
273.149	5.65190	158.0464	0.0002	0.000026	0.0071	0.0030	0.000192	0.0095	0.00520	0.0191	0.0304	9.262	2	2015 11 18	06:23
273.151	5.65201	158.0368	0.0003	0.000020	0.0054	0.0030	0.000192	0.0081	0.00520	0.0191	0.0297	9.267	2	2015 11 18	06:36
273.151	5.65203	158.0478	0.0001	0.000014	0.0028	0.0030	0.000191	0.0064	0.00520	0.0191	0.0290	9.262	2	2015 11 18	06:49
273.151	5.65192	158.0260	0.0002	0.000043	0.0026	0.0030	0.000195	0.0063	0.00520	0.0191	0.0291	9.257	2	2015 11 18	07:02
273.149	5.68051	158.9102	0.0005	0.000046	0.0095	0.0030	0.000197	0.0116	0.00520	0.0192	0.0316	14.601	2	2015 11 18	07:53
273.148	5.68043	159.0195	0.0001	0.000012	0.0039	0.0030	0.000192	0.0070	0.00520	0.0192	0.0292	17.157	2	2015 11 18	08:06
273.149	5.68053	159.0198	0.0004	0.000038	0.0066	0.0030	0.000195	0.0091	0.00520	0.0192	0.0302	16.962	2	2015 11 18	08:19
273.151	5.68066	159.0260	0.0005	0.000038	0.0046	0.0030	0.000195	0.0075	0.00520	0.0192	0.0295	13.684	2	2015 11 18	08:32
273.152	5.68070	159.0037	0.0001	0.000024	0.0019	0.0030	0.000193	0.0060	0.00520	0.0192	0.0289	13.330	2	2015 11 18	08:45
273.151	5.68057	159.0112	0.0004	0.000044	0.0011	0.0030	0.000196	0.0058	0.00520	0.0192	0.0289	12.851	2	2015 11 18	08:58
273.152	5.70902	158.7107	0.0003	0.000018	0.0648	0.0030	0.000193	0.0660	0.00520	0.0192	0.0877	10.570	2	2015 11 18	10:45
273.152	5.70892	158.6869	0.0002	0.000058	0.0599	0.0030	0.000200	0.0611	0.00520	0.0192	0.0819	10.499	2	2015 11 18	10:58
273.150	5.70872	158.6882	0.0005	0.000056	0.0492	0.0030	0.000200	0.0504	0.00520	0.0192	0.0694	10.494	2	2015 11 18	11:11
273.148	5.70859	158.6906	0.0004	0.000022	0.0451	0.0030	0.000193	0.0463	0.00520	0.0192	0.0647	10.522	2	2015 11 18	11:24
273.148	5.70864	158.6683	0.0002	0.000031	0.0259	0.0030	0.000194	0.0273	0.00520	0.0192	0.0443	10.462	2	2015 11 18	11:37
273.150	5.70878	158.6468	0.0006	0.000058	0.0321	0.0031	0.000200	0.0335	0.00520	0.0192	0.0507	10.491	2	2015 11 18	11:50
273.148	5.73641	159.4784	0.0008	0.000060	0.0666	0.0031	0.000201	0.0678	0.00520	0.0193	0.0895	10.677	2	2015 11 18	12:41
273.147	5.73643	159.5446	0.0003	0.000059	0.0048	0.0030	0.000201	0.0077	0.00520	0.0193	0.0298	11.028	2	2015 11 18	12:54
273.149	5.73671	159.5233	0.0009	0.000074	0.0034	0.0031	0.000206	0.0068	0.00520	0.0193	0.0296	12.650	2	2015 11 18	13:07
273.152	5.73688	159.5038	0.0006	0.000029	0.0085	0.0031	0.000194	0.0107	0.00520	0.0193	0.0310	11.026	2	2015 11 18	13:20
273.153	5.73681	159.5140	0.0003	0.000055	0.0091	0.0030	0.000200	0.0112	0.00520	0.0193	0.0314	11.144	2	2015 11 18	13:33
273.151	5.73656	159.5201	0.0007	0.000089	0.0054	0.0031	0.000212	0.0081	0.00520	0.0193	0.0302	11.160	2	2015 11 18	13:46
273.151	5.76457	160.2645	0.0002	0.000031	0.0913	0.0030	0.000195	0.0925	0.00520	0.0194	0.1187	10.986	2	2015 11 18	14:36
273.150	5.76446	160.3347	0.0002	0.000021	0.0069	0.0030	0.000194	0.0093	0.00520	0.0194	0.0303	11.523	2	2015 11 18	14:49
273.150	5.76439	160.3359	0.0001	0.000021	0.0134	0.0030	0.000194	0.0152	0.00520	0.0194	0.0338	11.550	2	2015 11 18	15:02
273.150	5.76438	160.3163	0.0001	0.000042	0.0280	0.0030	0.000197	0.0293	0.00520	0.0194	0.0461	11.466	2	2015 11 18	15:15
273.150	5.76443	160.3115	0.0002	0.000011	0.0046	0.0030	0.000193	0.0076	0.00520	0.0194	0.0295	11.592	2	2015 11 18	15:28
273.151	5.76444	160.3029	0.0002	0.000022	0.0123	0.0030	0.000194	0.0141	0.00520	0.0194	0.0330	11.510	2	2015 11 18	15:41
273.148	5.79164	161.3668	0.0006	0.000042	0.0106	0.0031	0.000198	0.0126	0.00520	0.0195	0.0322	14.864	2	2015 11 18	16:32
273.148	5.79171	161.3571	0.0004	0.000036	0.0132	0.0030	0.000196	0.0150	0.00520	0.0195	0.0337	14.170	2	2015 11 18	16:47
273.150	5.79190	161.3468	0.0007	0.000066	0.0122	0.0031	0.000204	0.0141	0.00520	0.0195	0.0332	12.064	2	2015 11 18	17:00
273.152	5.79201	161.3447	0.0004	0.000020	0.0093	0.0030	0.000194	0.0114	0.00520	0.0195	0.0314	12.070	2	2015 11 18	17:13

Table S2. Experimental (p, rho, T, x) data and detailed uncertainty information for isotherms measured on the (0.25019 argon + 0.74981 carbon dioxide) mixture (continued)

test: Ar_CO2_1511ef.dat (brief purge prior to test, 30 min equilibration time; 6 replicates per pressure; fine pressure steps near dew point)
chiS [specific magnetic susceptibility] = -0.6015E-08 [m3/kg]

T (K)	p (MPa)	rho (kg.m-3)	sigma_T (K)	sigma_p (MPa)	sigma_rho (kg.m-3)	u(T) (K)	u(p) (MPa)	u(rho) (kg.m-3)	u(MW) (g/mol)	u[rho(x)] (kg.m-3)	U_c/% (k = 2)	m_sorb (mg)	p_trans	date	time
273.153	1.11868	22.3837	0.0002	0.000008	0.0011	0.0030	0.000152	0.0022	0.00520	0.0027	0.0426	0.010	2	2015 11 18	19:18
273.151	1.11870	22.3812	0.0008	0.000005	0.0009	0.0031	0.000152	0.0021	0.00520	0.0027	0.0419	0.009	2	2015 11 18	19:31
273.148	1.11870	22.3838	0.0006	0.000007	0.0008	0.0031	0.000152	0.0020	0.00520	0.0027	0.0417	0.009	2	2015 11 18	19:44
273.148	1.11871	22.3834	0.0003	0.000005	0.0008	0.0030	0.000152	0.0021	0.00520	0.0027	0.0418	0.009	2	2015 11 18	19:57
273.149	1.11872	22.3826	0.0008	0.000008	0.0004	0.0031	0.000152	0.0018	0.00520	0.0027	0.0408	0.008	2	2015 11 18	20:10
273.152	1.11873	22.3812	0.0004	0.000004	0.0005	0.0030	0.000152	0.0018	0.00520	0.0027	0.0410	0.011	2	2015 11 18	20:23
273.148	1.52895	31.2738	0.0002	0.000007	0.0008	0.0030	0.000153	0.0022	0.00520	0.0038	0.0355	0.005	2	2015 11 18	21:24
273.150	1.52895	31.2733	0.0009	0.000006	0.0002	0.0031	0.000153	0.0019	0.00520	0.0038	0.0349	0.009	2	2015 11 18	21:37
273.152	1.52895	31.2741	0.0006	0.000006	0.0006	0.0031	0.000153	0.0021	0.00520	0.0038	0.0353	0.008	2	2015 11 18	21:50
273.153	1.52894	31.2741	0.0003	0.000007	0.0006	0.0030	0.000153	0.0021	0.00520	0.0038	0.0353	0.012	2	2015 11 18	22:04
273.151	1.52893	31.2739	0.0008	0.000009	0.0005	0.0031	0.000153	0.0021	0.00520	0.0038	0.0351	0.010	2	2015 11 18	22:17
273.152	2.08082	43.9367	0.0010	0.000032	0.0002	0.0032	0.000159	0.0023	0.00520	0.0053	0.0316	0.003	2	2015 11 18	23:09
273.149	2.08077	43.9369	0.0007	0.000007	0.0006	0.0031	0.000156	0.0024	0.00520	0.0053	0.0315	0.004	2	2015 11 18	23:22
273.148	2.08074	43.9359	0.0003	0.000006	0.0006	0.0030	0.000156	0.0024	0.00520	0.0053	0.0315	0.009	2	2015 11 18	23:35
273.150	2.08074	43.9362	0.0007	0.000011	0.0006	0.0031	0.000156	0.0024	0.00520	0.0053	0.0316	0.012	2	2015 11 18	23:48
273.152	2.08074	43.9363	0.0005	0.000008	0.0031	0.0030	0.000156	0.0044	0.00520	0.0053	0.0356	0.012	2	2015 11 19	00:01
273.153	2.08074	43.9370	0.0003	0.000010	0.0010	0.0030	0.000156	0.0026	0.00520	0.0053	0.0319	0.014	2	2015 11 19	00:14
273.147	2.99504	67.0781	0.0005	0.000009	0.0003	0.0030	0.000162	0.0030	0.00520	0.0081	0.0291	0.004	2	2015 11 19	01:25
273.150	2.99504	67.0757	0.0011	0.000011	0.0002	0.0032	0.000162	0.0030	0.00520	0.0081	0.0291	0.018	2	2015 11 19	01:38
273.153	2.99506	67.0776	0.0005	0.000008	0.0005	0.0030	0.000162	0.0030	0.00520	0.0081	0.0291	0.014	2	2015 11 19	01:51
273.153	2.99505	67.0770	0.0004	0.000011	0.0003	0.0030	0.000162	0.0030	0.00520	0.0081	0.0291	0.015	2	2015 11 19	02:04
273.151	2.99501	67.0734	0.0008	0.000012	0.0006	0.0031	0.000162	0.0031	0.00520	0.0081	0.0292	0.014	2	2015 11 19	02:17
273.146	4.01731	97.2792	0.0003	0.000017	0.0005	0.0030	0.000171	0.0039	0.00520	0.0118	0.0282	0.015	2	2015 11 19	03:31
273.149	4.01736	97.2775	0.0011	0.000032	0.0007	0.0032	0.000174	0.0039	0.00520	0.0118	0.0284	0.018	2	2015 11 19	03:44
273.153	4.01739	97.2777	0.0008	0.000012	0.0003	0.0031	0.000171	0.0038	0.00520	0.0118	0.0282	0.021	2	2015 11 19	03:57
273.154	4.01739	97.2719	0.0003	0.000008	0.0007	0.0030	0.000171	0.0039	0.00520	0.0118	0.0282	0.019	2	2015 11 19	04:10
273.152	4.01735	97.2778	0.0007	0.000020	0.0012	0.0031	0.000172	0.0041	0.00520	0.0118	0.0284	0.021	2	2015 11 19	04:23
273.149	4.01730	97.2715	0.0005	0.000027	0.0012	0.0030	0.000173	0.0041	0.00520	0.0118	0.0284	0.009	2	2015 11 19	04:36
273.147	4.53326	114.9733	0.0006	0.000012	0.0014	0.0031	0.000176	0.0046	0.00520	0.0139	0.0283	0.012	2	2015 11 19	05:49
273.150	4.53332	114.9728	0.0011	0.000027	0.0003	0.0032	0.000178	0.0044	0.00520	0.0139	0.0282	0.019	2	2015 11 19	06:02
273.153	4.53337	114.9738	0.0002	0.000022	0.0008	0.0030	0.000177	0.0044	0.00520	0.0139	0.0282	0.034	2	2015 11 19	06:19
273.152	4.53333	114.9723	0.0006	0.000017	0.0016	0.0031	0.000177	0.0047	0.00520	0.0139	0.0283	0.019	2	2015 11 19	06:32
273.150	4.53328	114.9744	0.0008	0.000019	0.0011	0.0031	0.000177	0.0045	0.00520	0.0139	0.0282	0.026	2	2015 11 19	06:45
273.148	4.53325	114.9729	0.0001	0.000012	0.0011	0.0030	0.000176	0.0045	0.00520	0.0139	0.0282	0.018	2	2015 11 19	06:58
273.146	5.01594	133.6284	0.0007	0.000036	0.0013	0.0031	0.000185	0.0051	0.00520	0.0162	0.0284	0.022	2	2015 11 19	08:12
273.150	5.01605	133.6279	0.0013	0.000039	0.0009	0.0033	0.000185	0.0050	0.00520	0.0162	0.0285	0.025	2	2015 11 19	08:25
273.154	5.01614	133.6275	0.0003	0.000005	0.0004	0.0030	0.000181	0.0049	0.00520	0.0162	0.0282	0.031	2	2015 11 19	08:42
273.153	5.01608	133.6253	0.0007	0.000019	0.0021	0.0031	0.000182	0.0054	0.00520	0.0162	0.0285	0.020	2	2015 11 19	08:55
273.149	5.01600	133.6284	0.0009	0.000035	0.0003	0.0031	0.000185	0.0049	0.00520	0.0162	0.0284	0.030	2	2015 11 19	09:08
273.148	5.01596	133.6266	0.0001	0.000025	0.0012	0.0030	0.000183	0.0051	0.00520	0.0162	0.0283	0.011	2	2015 11 19	09:22
273.151	5.13518	138.6222	0.0011	0.000038	0.0006	0.0032	0.000187	0.0051	0.00520	0.0168	0.0285	0.025	2	2015 11 19	10:14
273.148	5.13506	138.6225	0.0003	0.000018	0.0014	0.0030	0.000184	0.0053	0.00520	0.0168	0.0284	0.026	2	2015 11 19	10:29
273.148	5.13510	138.6202	0.0006	0.000029	0.0010	0.0031	0.000185	0.0052	0.00520	0.0168	0.0284	0.020	2	2015 11 19	10:42
273.151	5.13519	138.6217	0.0008	0.000044	0.0008	0.0031	0.000188	0.0051	0.00520	0.0168	0.0285	0.029	2	2015 11 19	10:55
273.153	5.13523	138.6191	0.0002	0.000012	0.0003	0.0030	0.000183	0.0051	0.00520	0.0168	0.0283	0.024	2	2015 11 19	11:08
273.152	5.13519	138.6228	0.0005	0.000025	0.0009	0.0030	0.000184	0.0051	0.00520	0.0168	0.0284	0.034	2	2015 11 19	11:21
273.150	5.24690	143.4691	0.0003	0.000030	0.0012	0.0030	0.000187	0.0054	0.00520	0.0174	0.0285	0.028	2	2015 11 19	12:13
273.149	5.24687	143.4668	0.0001	0.000020	0.0015	0.0030	0.000185	0.0055	0.00520	0.0174	0.0285	0.012	2	2015 11 19	12:26
273.150	5.24688	143.4667	0.0003	0.000029	0.0004	0.0030	0.000187	0.0052	0.00520	0.0174	0.0284	0.012	2	2015 11 19	12:39
273.151	5.24692	143.4685	0.0003	0.000031	0.0005	0.0030	0.000187	0.0052	0.00520	0.0174	0.0284	0.025	2	2015 11 19	12:52
273.152	5.24694	143.4671	0.0001	0.000013	0.0002	0.0030	0.000185	0.0052	0.00520	0.0174	0.0284	0.031	2	2015 11 19	13:05

T (K)	p (MPa)	rho (kg.m−3)	sigma_T (K)	sigma_p (MPa)	sigma_rho (kg.m−3)	u(T) (K)	u(p) (MPa)	u(rho) (kg.m−3)	u(MW) (g/mol)	u[rho(x)] (kg.m−3)	U_c/% (k = 2)	m_sorb (mg)	p_trans	date	time
# test: Ar_CO2_1511ef.dat (continued)															
273.150	5.25634	143.8858	0.0002	0.000032	0.0002	0.0030	0.000187	0.0052	0.00520	0.0174	0.0284	0.026	2	2015 11 19	13:55
273.151	5.25637	143.8830	0.0004	0.000014	0.0005	0.0030	0.000185	0.0052	0.00520	0.0174	0.0284	0.018	2	2015 11 19	14:08
273.152	5.25639	143.8838	0.0001	0.000021	0.0005	0.0030	0.000186	0.0052	0.00520	0.0174	0.0284	0.018	2	2015 11 19	14:21
273.151	5.25636	143.8830	0.0003	0.000018	0.0010	0.0030	0.000185	0.0053	0.00520	0.0174	0.0284	0.019	2	2015 11 19	14:34
273.150	5.25632	143.8855	0.0002	0.000032	0.0007	0.0030	0.000187	0.0052	0.00520	0.0174	0.0285	0.027	2	2015 11 19	14:47
273.150	5.25634	143.8841	0.0002	0.000010	0.0001	0.0030	0.000185	0.0052	0.00520	0.0174	0.0284	0.017	2	2015 11 19	15:00
273.150	5.26577	144.3031	0.0003	0.000027	0.0013	0.0030	0.000187	0.0054	0.00520	0.0175	0.0285	0.023	2	2015 11 19	15:51
273.149	5.26574	144.3010	0.0001	0.000026	0.0014	0.0030	0.000186	0.0054	0.00520	0.0175	0.0285	0.034	2	2015 11 19	16:04
273.150	5.26574	144.3022	0.0003	0.000032	0.0006	0.0030	0.000187	0.0053	0.00520	0.0175	0.0285	0.027	2	2015 11 19	16:17
273.151	5.26580	144.3024	0.0004	0.000020	0.0004	0.0030	0.000186	0.0052	0.00520	0.0175	0.0284	0.025	2	2015 11 19	16:30
273.152	5.26580	144.3013	0.0002	0.000009	0.0011	0.0030	0.000185	0.0053	0.00520	0.0175	0.0284	0.029	2	2015 11 19	16:43
273.152	5.26578	144.3008	0.0002	0.000043	0.0009	0.0030	0.000190	0.0053	0.00520	0.0175	0.0285	0.021	2	2015 11 19	16:56
273.151	5.27504	144.7123	0.0002	0.000015	0.0007	0.0030	0.000185	0.0053	0.00520	0.0175	0.0284	0.022	2	2015 11 19	17:47
273.151	5.27504	144.7112	0.0001	0.000018	0.0013	0.0030	0.000186	0.0054	0.00520	0.0175	0.0285	0.031	2	2015 11 19	18:00
273.151	5.27501	144.7116	0.0002	0.000020	0.0008	0.0030	0.000186	0.0053	0.00520	0.0175	0.0284	0.027	2	2015 11 19	18:13
273.150	5.27499	144.7106	0.0002	0.000015	0.0005	0.0030	0.000185	0.0053	0.00520	0.0175	0.0284	0.017	2	2015 11 19	18:26
273.150	5.27500	144.7116	0.0003	0.000024	0.0002	0.0030	0.000186	0.0052	0.00520	0.0175	0.0284	0.019	2	2015 11 19	18:39
273.151	5.27503	144.7100	0.0003	0.000018	0.0002	0.0030	0.000186	0.0052	0.00520	0.0175	0.0284	0.032	2	2015 11 19	18:52
273.150	5.28427	145.1253	0.0002	0.000017	0.0012	0.0030	0.000186	0.0054	0.00520	0.0176	0.0285	0.018	2	2015 11 19	19:42
273.149	5.28428	145.1269	0.0002	0.000018	0.0009	0.0030	0.000186	0.0053	0.00520	0.0176	0.0285	0.026	2	2015 11 19	19:55
273.150	5.28431	145.1255	0.0003	0.000017	0.0010	0.0030	0.000186	0.0053	0.00520	0.0176	0.0285	0.025	2	2015 11 19	20:08
273.152	5.28434	145.1260	0.0003	0.000023	0.0007	0.0030	0.000186	0.0053	0.00520	0.0176	0.0285	0.031	2	2015 11 19	20:21
273.152	5.28433	145.1258	0.0002	0.000009	0.0010	0.0030	0.000185	0.0053	0.00520	0.0176	0.0284	0.034	2	2015 11 19	20:34
273.151	5.28431	145.1253	0.0003	0.000016	0.0002	0.0030	0.000186	0.0052	0.00520	0.0176	0.0284	0.019	2	2015 11 19	20:47
273.151	5.29361	145.5392	0.0001	0.000012	0.0005	0.0030	0.000185	0.0053	0.00520	0.0176	0.0284	0.017	2	2015 11 19	21:38
273.151	5.29358	145.5407	0.0001	0.000014	0.0007	0.0030	0.000186	0.0053	0.00520	0.0176	0.0284	0.028	2	2015 11 19	21:51
273.150	5.29353	145.5396	0.0003	0.000017	0.0006	0.0030	0.000186	0.0053	0.00520	0.0176	0.0284	0.021	2	2015 11 19	22:04
273.150	5.29353	145.5400	0.0001	0.000010	0.0008	0.0030	0.000185	0.0053	0.00520	0.0176	0.0284	0.012	2	2015 11 19	22:17
273.150	5.29352	145.5374	0.0003	0.000013	0.0013	0.0030	0.000185	0.0054	0.00520	0.0176	0.0285	0.017	2	2015 11 19	22:30
273.151	5.29357	145.5387	0.0003	0.000018	0.0007	0.0030	0.000186	0.0053	0.00520	0.0176	0.0285	0.032	2	2015 11 19	22:43
273.149	5.30274	145.9541	0.0001	0.000024	0.0005	0.0030	0.000187	0.0053	0.00520	0.0177	0.0285	0.041	2	2015 11 19	23:34
273.150	5.30276	145.9556	0.0003	0.000030	0.0009	0.0030	0.000188	0.0054	0.00520	0.0177	0.0285	0.030	2	2015 11 19	23:47
273.151	5.30280	145.9551	0.0004	0.000025	0.0008	0.0030	0.000187	0.0053	0.00520	0.0177	0.0285	0.034	2	2015 11 20	00:00
273.152	5.30283	145.9533	0.0002	0.000026	0.0007	0.0030	0.000187	0.0053	0.00520	0.0177	0.0285	0.033	2	2015 11 20	00:13
273.152	5.30281	145.9548	0.0003	0.000016	0.0020	0.0030	0.000186	0.0057	0.00520	0.0177	0.0286	0.034	2	2015 11 20	00:26
273.150	5.30276	145.9547	0.0003	0.000025	0.0005	0.0030	0.000187	0.0053	0.00520	0.0177	0.0285	0.031	2	2015 11 20	00:39
273.151	5.31204	146.3675	0.0000	0.000020	0.0006	0.0030	0.000186	0.0053	0.00520	0.0177	0.0285	−0.000	2	2015 11 20	01:30
273.151	5.31198	146.3683	0.0002	0.000013	0.0012	0.0030	0.000186	0.0054	0.00520	0.0177	0.0285	0.029	2	2015 11 20	01:43
273.150	5.31196	146.3675	0.0002	0.000014	0.0002	0.0030	0.000186	0.0053	0.00520	0.0177	0.0284	0.034	2	2015 11 20	01:56
273.150	5.31195	146.3657	0.0002	0.000016	0.0008	0.0030	0.000186	0.0053	0.00520	0.0177	0.0285	0.026	2	2015 11 20	02:09
273.151	5.31202	146.3691	0.0003	0.000035	0.0014	0.0030	0.000188	0.0055	0.00520	0.0177	0.0286	0.027	2	2015 11 20	02:22
273.152	5.31203	146.3667	0.0002	0.000016	0.0006	0.0030	0.000186	0.0053	0.00520	0.0177	0.0285	0.025	2	2015 11 20	02:35
273.149	5.32110	146.7790	0.0001	0.000016	0.0007	0.0030	0.000186	0.0053	0.00520	0.0178	0.0285	0.023	2	2015 11 20	03:25
273.150	5.32113	146.7787	0.0004	0.000030	0.0013	0.0030	0.000188	0.0055	0.00520	0.0178	0.0286	0.022	2	2015 11 20	03:38
273.152	5.32120	146.7786	0.0004	0.000021	0.0002	0.0030	0.000187	0.0053	0.00520	0.0178	0.0285	0.010	2	2015 11 20	03:51
273.152	5.32120	146.7796	0.0001	0.000022	0.0016	0.0030	0.000187	0.0056	0.00520	0.0178	0.0286	0.031	2	2015 11 20	04:04
273.151	5.32116	146.7829	0.0004	0.000033	0.0006	0.0030	0.000188	0.0053	0.00520	0.0178	0.0285	0.037	2	2015 11 20	04:17
273.150	5.32110	146.7794	0.0003	0.000015	0.0004	0.0030	0.000186	0.0053	0.00520	0.0178	0.0285	0.020	2	2015 11 20	04:30
273.152	5.33028	147.1940	0.0003	0.000018	0.0010	0.0030	0.000186	0.0054	0.00520	0.0178	0.0285	0.023	2	2015 11 20	05:21
273.150	5.33021	147.1906	0.0005	0.000033	0.0012	0.0030	0.000188	0.0054	0.00520	0.0178	0.0286	0.023	2	2015 11 20	05:34
273.149	5.33017	147.1905	0.0002	0.000013	0.0004	0.0030	0.000186	0.0053	0.00520	0.0178	0.0285	0.026	2	2015 11 20	05:47
273.149	5.33019	147.1907	0.0004	0.000016	0.0005	0.0030	0.000186	0.0053	0.00520	0.0178	0.0285	0.022	2	2015 11 20	06:00
273.151	5.33027	147.1920	0.0006	0.000024	0.0006	0.0031	0.000187	0.0053	0.00520	0.0178	0.0285	0.026	2	2015 11 20	06:13
273.153	5.33031	147.1908	0.0004	0.000035	0.0006	0.0030	0.000189	0.0053	0.00520	0.0178	0.0285	0.006	2	2015 11 20	06:26

T	p	rho	sigma_T	sigma_p	sigma_rho	u(T)	u(p)	u(rho)	u(MW)	u[rho(x)]	U_c/%	m_sorb	p_trans	date	time
(K)	(MPa)	(kg.m-3)	(K)	(MPa)	(kg.m-3)	(K)	(MPa)	(kg.m-3)	(g/mol)	(kg.m-3)	(k = 2)	(mg)			
# test: Ar_CO2_1511ef.dat (continued)															
273.148	5.33916	147.5995	0.0002	0.000022	0.0003	0.0030	0.000187	0.0053	0.00520	0.0179	0.0285	0.021	2	2015 11 20	07:17
273.150	5.33923	147.5997	0.0006	0.000015	0.0011	0.0031	0.000186	0.0055	0.00520	0.0179	0.0285	0.022	2	2015 11 20	07:30
273.152	5.33930	147.5993	0.0004	0.000024	0.0009	0.0030	0.000187	0.0054	0.00520	0.0179	0.0285	0.023	2	2015 11 20	07:43
273.152	5.33930	147.5994	0.0002	0.000013	0.0012	0.0030	0.000186	0.0055	0.00520	0.0179	0.0285	0.031	2	2015 11 20	07:56
273.151	5.33925	147.6015	0.0005	0.000022	0.0005	0.0030	0.000187	0.0053	0.00520	0.0179	0.0285	0.024	2	2015 11 20	08:09
273.150	5.33922	147.5996	0.0002	0.000011	0.0014	0.0030	0.000186	0.0055	0.00520	0.0179	0.0285	0.019	2	2015 11 20	08:22
273.152	5.34835	148.0135	0.0001	0.000019	0.0007	0.0030	0.000187	0.0054	0.00520	0.0179	0.0285	0.027	2	2015 11 20	09:13
273.151	5.34834	148.0132	0.0002	0.000015	0.0010	0.0030	0.000186	0.0054	0.00520	0.0179	0.0285	0.028	2	2015 11 20	09:26
273.150	5.34832	148.0120	0.0002	0.000037	0.0010	0.0030	0.000189	0.0054	0.00520	0.0179	0.0286	0.020	2	2015 11 20	09:39
273.150	5.34832	148.0140	0.0001	0.000017	0.0006	0.0030	0.000187	0.0054	0.00520	0.0179	0.0285	0.026	2	2015 11 20	09:52
273.150	5.34830	148.0155	0.0000	0.000024	0.0002	0.0030	0.000187	0.0053	0.00520	0.0179	0.0285	0.022	2	2015 11 20	10:05
273.151	5.35727	148.4219	0.0003	0.000019	0.0003	0.0030	0.000187	0.0053	0.00520	0.0180	0.0285	0.033	2	2015 11 20	10:55
273.149	5.35722	148.4216	0.0002	0.000014	0.0003	0.0030	0.000187	0.0053	0.00520	0.0180	0.0285	0.037	2	2015 11 20	11:08
273.150	5.35722	148.4194	0.0002	0.000034	0.0013	0.0030	0.000189	0.0055	0.00520	0.0180	0.0286	0.034	2	2015 11 20	11:21
273.151	5.35729	148.4206	0.0004	0.000038	0.0017	0.0030	0.000190	0.0056	0.00520	0.0180	0.0287	0.031	2	2015 11 20	11:34
273.152	5.35731	148.4240	0.0003	0.000030	0.0011	0.0030	0.000188	0.0055	0.00520	0.0180	0.0286	0.037	2	2015 11 20	11:47
273.152	5.35728	148.4202	0.0003	0.000021	0.0010	0.0030	0.000187	0.0054	0.00520	0.0180	0.0285	0.026	2	2015 11 20	12:00
273.151	5.36608	148.8255	0.0003	0.000032	0.0017	0.0030	0.000189	0.0057	0.00520	0.0180	0.0286	0.035	2	2015 11 20	12:51
273.151	5.36611	148.8243	0.0002	0.000018	0.0009	0.0030	0.000187	0.0054	0.00520	0.0180	0.0285	0.030	2	2015 11 20	13:04
273.151	5.36610	148.8225	0.0002	0.000019	0.0014	0.0030	0.000187	0.0056	0.00520	0.0180	0.0286	0.029	2	2015 11 20	13:17
273.150	5.36606	148.8249	0.0003	0.000032	0.0011	0.0030	0.000189	0.0055	0.00520	0.0180	0.0286	0.031	2	2015 11 20	13:30
273.149	5.36608	148.8239	0.0001	0.000020	0.0006	0.0030	0.000187	0.0054	0.00520	0.0180	0.0285	0.026	2	2015 11 20	13:43
273.150	5.36605	148.8225	0.0003	0.000019	0.0013	0.0030	0.000187	0.0055	0.00520	0.0180	0.0286	0.038	2	2015 11 20	13:56
273.150	5.37476	149.2255	0.0003	0.000025	0.0005	0.0030	0.000188	0.0054	0.00520	0.0180	0.0285	0.037	2	2015 11 20	14:47
273.149	5.37471	149.2216	0.0001	0.000027	0.0006	0.0030	0.000188	0.0054	0.00520	0.0180	0.0285	0.051	2	2015 11 20	15:00
273.149	5.37471	149.2196	0.0002	0.000031	0.0004	0.0030	0.000189	0.0054	0.00520	0.0180	0.0285	0.022	2	2015 11 20	15:13
273.150	5.37477	149.2221	0.0004	0.000021	0.0007	0.0030	0.000187	0.0054	0.00520	0.0180	0.0285	0.028	2	2015 11 20	15:26
273.151	5.37475	149.2208	0.0002	0.000015	0.0005	0.0030	0.000187	0.0054	0.00520	0.0180	0.0285	0.026	2	2015 11 20	15:39
273.151	5.37476	149.2216	0.0002	0.000030	0.0003	0.0030	0.000189	0.0054	0.00520	0.0180	0.0285	0.037	2	2015 11 20	15:52
273.150	5.38332	149.6163	0.0001	0.000026	0.0018	0.0030	0.000188	0.0057	0.00520	0.0181	0.0287	0.042	2	2015 11 20	16:42
273.150	5.38330	149.6169	0.0001	0.000036	0.0010	0.0030	0.000190	0.0055	0.00520	0.0181	0.0286	0.044	2	2015 11 20	16:55
273.150	5.38331	149.6183	0.0001	0.000030	0.0019	0.0030	0.000189	0.0058	0.00520	0.0181	0.0287	0.029	2	2015 11 20	17:08
273.150	5.38329	149.6158	0.0002	0.000035	0.0014	0.0030	0.000190	0.0056	0.00520	0.0181	0.0286	0.033	2	2015 11 20	17:21
273.150	5.38331	149.6143	0.0001	0.000020	0.0004	0.0030	0.000187	0.0054	0.00520	0.0181	0.0285	0.039	2	2015 11 20	17:34
273.149	5.39171	150.0042	0.0002	0.000013	0.0015	0.0030	0.000187	0.0056	0.00520	0.0181	0.0286	0.035	2	2015 11 20	18:29
273.150	5.39174	150.0021	0.0002	0.000020	0.0012	0.0030	0.000188	0.0055	0.00520	0.0181	0.0286	0.027	2	2015 11 20	18:42
273.150	5.39176	150.0035	0.0001	0.000016	0.0007	0.0030	0.000187	0.0054	0.00520	0.0181	0.0285	0.035	2	2015 11 20	18:55
273.150	5.39172	150.0063	0.0002	0.000008	0.0008	0.0030	0.000187	0.0055	0.00520	0.0181	0.0285	0.042	2	2015 11 20	19:08
273.149	5.39168	150.0035	0.0002	0.000012	0.0010	0.0030	0.000187	0.0055	0.00520	0.0181	0.0285	0.039	2	2015 11 20	19:21
273.149	5.39169	150.0022	0.0001	0.000012	0.0005	0.0030	0.000187	0.0054	0.00520	0.0181	0.0285	0.031	2	2015 11 20	19:34
273.150	5.40014	150.3928	0.0004	0.000031	0.0009	0.0030	0.000189	0.0055	0.00520	0.0182	0.0286	0.036	2	2015 11 20	20:24
273.149	5.40012	150.3937	0.0003	0.000010	0.0007	0.0030	0.000187	0.0055	0.00520	0.0182	0.0285	0.048	2	2015 11 20	20:37
273.149	5.40011	150.3912	0.0001	0.000013	0.0014	0.0030	0.000187	0.0056	0.00520	0.0182	0.0286	0.048	2	2015 11 20	20:50
273.150	5.40013	150.3932	0.0003	0.000028	0.0009	0.0030	0.000189	0.0055	0.00520	0.0182	0.0286	0.054	2	2015 11 20	21:03
273.151	5.40018	150.3920	0.0003	0.000014	0.0003	0.0030	0.000187	0.0054	0.00520	0.0182	0.0285	0.044	2	2015 11 20	21:16
273.151	5.40019	150.3967	0.0001	0.000011	0.0006	0.0030	0.000187	0.0054	0.00520	0.0182	0.0285	0.055	2	2015 11 20	21:29
273.149	5.40849	150.7790	0.0004	0.000016	0.0022	0.0030	0.000187	0.0059	0.00520	0.0182	0.0287	0.054	2	2015 11 20	22:19
273.150	5.40853	150.7785	0.0003	0.000017	0.0007	0.0030	0.000187	0.0055	0.00520	0.0182	0.0286	0.066	2	2015 11 20	22:32
273.151	5.40856	150.7812	0.0001	0.000013	0.0005	0.0030	0.000187	0.0054	0.00520	0.0182	0.0285	0.064	2	2015 11 20	22:45
273.150	5.40853	150.7782	0.0004	0.000014	0.0008	0.0030	0.000187	0.0055	0.00520	0.0182	0.0286	0.062	2	2015 11 20	22:58
273.149	5.40847	150.7782	0.0002	0.000015	0.0004	0.0030	0.000187	0.0054	0.00520	0.0182	0.0285	0.069	2	2015 11 20	23:11
273.148	5.40845	150.7777	0.0001	0.000016	0.0006	0.0030	0.000187	0.0054	0.00520	0.0182	0.0285	0.067	2	2015 11 20	23:24

T (K)	p (MPa)	rho (kg.m−3)	sigma_T (K)	sigma_p (MPa)	sigma_rho (kg.m−3)	u(T) (K)	u(p) (MPa)	u(rho) (kg.m−3)	u(MW) (g/mol)	u[rho(x)] (kg.m−3)	U_c/% (k = 2)	m_sorb (mg)	p_trans	date	time
# test: Ar_CO2_1511ef.dat (continued)															
273.151	5.41680	151.1602	0.0005	0.000029	0.0005	0.0030	0.000189	0.0054	0.00520	0.0183	0.0286	0.128	2	2015 11 21	00:15
273.149	5.41672	151.1635	0.0003	0.000029	0.0006	0.0030	0.000189	0.0054	0.00520	0.0183	0.0286	0.134	2	2015 11 21	00:28
273.149	5.41671	151.1585	0.0001	0.000021	0.0009	0.0030	0.000188	0.0055	0.00520	0.0183	0.0286	0.132	2	2015 11 21	00:41
273.149	5.41677	151.1580	0.0005	0.000026	0.0004	0.0030	0.000189	0.0054	0.00520	0.0183	0.0286	0.135	2	2015 11 21	00:54
273.151	5.41678	151.1585	0.0002	0.000024	0.0011	0.0030	0.000188	0.0055	0.00520	0.0183	0.0286	0.139	2	2015 11 21	01:07
273.151	5.41677	151.1592	0.0002	0.000028	0.0006	0.0030	0.000189	0.0054	0.00520	0.0183	0.0286	0.127	2	2015 11 21	01:20
273.150	5.42472	151.5091	0.0003	0.000025	0.0009	0.0030	0.000189	0.0055	0.00520	0.0183	0.0286	0.360	2	2015 11 21	02:11
273.151	5.42477	151.5100	0.0002	0.000014	0.0004	0.0030	0.000188	0.0054	0.00520	0.0183	0.0286	0.353	2	2015 11 21	02:24
273.151	5.42476	151.5082	0.0001	0.000014	0.0007	0.0030	0.000187	0.0055	0.00520	0.0183	0.0286	0.356	2	2015 11 21	02:37
273.150	5.42472	151.5078	0.0003	0.000024	0.0009	0.0030	0.000189	0.0055	0.00520	0.0183	0.0286	0.342	2	2015 11 21	02:50
273.149	5.42467	151.5090	0.0002	0.000016	0.0010	0.0030	0.000188	0.0055	0.00520	0.0183	0.0286	0.362	2	2015 11 21	03:03
273.149	5.42467	151.5059	0.0001	0.000007	0.0007	0.0030	0.000187	0.0055	0.00520	0.0183	0.0286	0.360	2	2015 11 21	03:16
273.150	5.43159	151.7529	0.0005	0.000044	0.0066	0.0030	0.000192	0.0089	0.00520	0.0184	0.0302	1.107	2	2015 11 21	04:06
273.148	5.43148	151.7457	0.0003	0.000025	0.0048	0.0030	0.000189	0.0075	0.00520	0.0184	0.0294	1.136	2	2015 11 21	04:19
273.148	5.43147	151.7428	0.0001	0.000028	0.0023	0.0030	0.000189	0.0060	0.00520	0.0184	0.0288	1.170	2	2015 11 21	04:32
273.149	5.43158	151.7447	0.0005	0.000028	0.0005	0.0030	0.000189	0.0055	0.00520	0.0184	0.0286	1.207	2	2015 11 21	04:45
273.151	5.43167	151.7477	0.0004	0.000025	0.0027	0.0030	0.000189	0.0062	0.00520	0.0184	0.0289	1.217	2	2015 11 21	04:58
273.151	5.43167	151.7503	0.0002	0.000023	0.0013	0.0030	0.000188	0.0056	0.00520	0.0184	0.0286	1.194	2	2015 11 21	05:11
273.149	5.43706	151.8832	0.0003	0.000035	0.0092	0.0030	0.000190	0.0112	0.00520	0.0184	0.0314	2.069	2	2015 11 21	06:02
273.150	5.43717	151.8799	0.0004	0.000031	0.0043	0.0030	0.000190	0.0072	0.00520	0.0184	0.0293	2.210	2	2015 11 21	06:15
273.151	5.43722	151.8747	0.0002	0.000020	0.0020	0.0030	0.000188	0.0059	0.00520	0.0184	0.0287	2.295	2	2015 11 21	06:28
273.151	5.43712	151.8744	0.0004	0.000036	0.0011	0.0030	0.000191	0.0056	0.00520	0.0184	0.0287	2.342	2	2015 11 21	06:41
273.149	5.43698	151.8756	0.0004	0.000042	0.0037	0.0030	0.000192	0.0067	0.00520	0.0184	0.0291	2.358	2	2015 11 21	06:54
273.148	5.43694	151.8704	0.0001	0.000023	0.0034	0.0030	0.000189	0.0066	0.00520	0.0184	0.0290	2.373	2	2015 11 21	07:07
273.150	5.44215	152.0068	0.0005	0.000057	0.0087	0.0030	0.000196	0.0108	0.00520	0.0184	0.0313	3.037	2	2015 11 21	07:58
273.148	5.44204	151.9891	0.0003	0.000024	0.0077	0.0030	0.000189	0.0099	0.00520	0.0184	0.0306	3.121	2	2015 11 21	08:11
273.148	5.44207	151.9785	0.0002	0.000029	0.0074	0.0030	0.000190	0.0096	0.00520	0.0184	0.0304	3.185	2	2015 11 21	08:24
273.150	5.44219	151.9736	0.0005	0.000031	0.0046	0.0030	0.000190	0.0074	0.00520	0.0184	0.0294	3.258	2	2015 11 21	08:37
273.151	5.44227	151.9754	0.0002	0.000016	0.0013	0.0030	0.000188	0.0056	0.00520	0.0184	0.0286	3.329	2	2015 11 21	08:50
273.151	5.44218	151.9704	0.0002	0.000036	0.0008	0.0030	0.000191	0.0055	0.00520	0.0184	0.0287	3.387	2	2015 11 21	09:03
273.150	5.44718	152.1044	0.0003	0.000025	0.0103	0.0030	0.000189	0.0122	0.00520	0.0184	0.0320	3.852	2	2015 11 21	09:54
273.151	5.44724	152.0943	0.0002	0.000019	0.0057	0.0030	0.000188	0.0082	0.00520	0.0184	0.0297	3.934	2	2015 11 21	10:07
273.150	5.44719	152.1019	0.0002	0.000028	0.0035	0.0030	0.000189	0.0067	0.00520	0.0184	0.0290	4.032	2	2015 11 21	10:20
273.150	5.44708	152.0945	0.0003	0.000033	0.0040	0.0030	0.000190	0.0070	0.00520	0.0184	0.0292	4.106	2	2015 11 21	10:33
273.149	5.44705	152.0875	0.0001	0.000007	0.0042	0.0030	0.000187	0.0071	0.00520	0.0184	0.0292	4.145	2	2015 11 21	10:46
273.149	5.44711	152.0986	0.0003	0.000033	0.0035	0.0030	0.000190	0.0066	0.00520	0.0184	0.0291	4.185	2	2015 11 21	10:59
273.149	5.45196	152.2168	0.0003	0.000016	0.0078	0.0030	0.000188	0.0100	0.00520	0.0184	0.0306	4.638	2	2015 11 21	11:49
273.149	5.45199	152.2215	0.0002	0.000036	0.0072	0.0030	0.000191	0.0095	0.00520	0.0184	0.0304	4.702	2	2015 11 21	12:02
273.150	5.45207	152.2133	0.0003	0.000026	0.0065	0.0030	0.000189	0.0088	0.00520	0.0184	0.0300	4.752	2	2015 11 21	12:15
273.151	5.45209	152.2046	0.0001	0.000014	0.0033	0.0030	0.000188	0.0065	0.00520	0.0184	0.0290	4.817	2	2015 11 21	12:28
273.150	5.45205	152.2044	0.0002	0.000018	0.0018	0.0030	0.000188	0.0058	0.00520	0.0184	0.0287	4.866	2	2015 11 21	12:41
273.149	5.45195	152.2097	0.0004	0.000034	0.0011	0.0030	0.000190	0.0056	0.00520	0.0184	0.0287	4.913	2	2015 11 21	12:54
273.150	5.45694	152.3422	0.0001	0.000015	0.0066	0.0030	0.000188	0.0090	0.00520	0.0184	0.0301	5.269	2	2015 11 21	13:45
273.150	5.45694	152.3257	0.0001	0.000014	0.0050	0.0030	0.000188	0.0077	0.00520	0.0184	0.0294	5.335	2	2015 11 21	13:58
273.150	5.45689	152.3341	0.0001	0.000023	0.0045	0.0030	0.000189	0.0073	0.00520	0.0184	0.0293	5.385	2	2015 11 21	14:11
273.149	5.45684	152.3262	0.0002	0.000017	0.0039	0.0030	0.000188	0.0069	0.00520	0.0184	0.0291	5.428	2	2015 11 21	14:24
273.149	5.45684	152.3216	0.0001	0.000009	0.0035	0.0030	0.000188	0.0066	0.00520	0.0184	0.0290	5.450	2	2015 11 21	14:37
273.149	5.45686	152.3141	0.0001	0.000019	0.0034	0.0030	0.000188	0.0066	0.00520	0.0184	0.0290	5.479	2	2015 11 21	14:50
273.150	5.46176	152.4590	0.0001	0.000009	0.0065	0.0030	0.000188	0.0089	0.00520	0.0184	0.0300	5.767	2	2015 11 21	15:41
273.150	5.46180	152.4566	0.0002	0.000015	0.0056	0.0030	0.000188	0.0081	0.00520	0.0184	0.0297	5.820	2	2015 11 21	15:54
273.151	5.46183	152.4508	0.0001	0.000008	0.0046	0.0030	0.000188	0.0074	0.00520	0.0184	0.0293	5.858	2	2015 11 21	16:07
273.151	5.46181	152.4508	0.0001	0.000018	0.0031	0.0030	0.000188	0.0064	0.00520	0.0184	0.0289	5.898	2	2015 11 21	16:20
273.150	5.46177	152.4434	0.0001	0.000012	0.0036	0.0030	0.000188	0.0068	0.00520	0.0184	0.0290	5.930	2	2015 11 21	16:33
273.150	5.46175	152.4344	0.0001	0.000012	0.0026	0.0030	0.000188	0.0062	0.00520	0.0184	0.0288	5.946	2	2015 11 21	16:46

test: Ar_CO2_1511ef.dat (continued)

T	p	rho	sigma_T	sigma_p	sigma_rho	u(T)	u(p)	u(rho)	u(MW)	u[rho(x)]	U_c/%	m_sorb	p_trans	date	time
(K)	(MPa)	(kg.m-3)	(K)	(MPa)	(kg.m-3)	(K)	(MPa)	(kg.m-3)	(g/mol)	(kg.m-3)	(k = 2)	(mg)			
273.151	5.46665	152.5969	0.0000	0.000011	0.0053	0.0030	0.000188	0.0079	0.00520	0.0185	0.0295	6.175	2	2015 11 21	17:36
273.150	5.46661	152.5928	0.0002	0.000011	0.0047	0.0030	0.000188	0.0075	0.00520	0.0185	0.0294	6.240	2	2015 11 21	17:49
273.150	5.46662	152.5850	0.0001	0.000016	0.0041	0.0030	0.000188	0.0071	0.00520	0.0185	0.0292	6.264	2	2015 11 21	18:02
273.151	5.46664	152.5723	0.0001	0.000011	0.0043	0.0030	0.000188	0.0072	0.00520	0.0185	0.0292	6.298	2	2015 11 21	18:15
273.151	5.46666	152.5701	0.0001	0.000021	0.0031	0.0030	0.000189	0.0064	0.00520	0.0185	0.0289	6.307	2	2015 11 21	18:28
273.151	5.46665	152.5707	0.0001	0.000007	0.0026	0.0030	0.000188	0.0062	0.00520	0.0185	0.0288	6.326	2	2015 11 21	18:41
273.150	5.47141	152.7215	0.0001	0.000010	0.0051	0.0030	0.000188	0.0078	0.00520	0.0185	0.0295	6.550	2	2015 11 21	19:32
273.150	5.47143	152.7036	0.0001	0.000017	0.0057	0.0030	0.000189	0.0083	0.00520	0.0185	0.0297	6.598	2	2015 11 21	19:45
273.151	5.47148	152.7080	0.0002	0.000017	0.0048	0.0030	0.000189	0.0075	0.00520	0.0185	0.0294	6.623	2	2015 11 21	19:58
273.151	5.47146	152.6953	0.0001	0.000021	0.0027	0.0030	0.000189	0.0062	0.00520	0.0185	0.0289	6.647	2	2015 11 21	20:11
273.150	5.47139	152.6993	0.0002	0.000020	0.0027	0.0030	0.000189	0.0062	0.00520	0.0185	0.0289	6.657	2	2015 11 21	20:24
273.150	5.47136	152.7057	0.0001	0.000013	0.0023	0.0030	0.000188	0.0060	0.00520	0.0185	0.0288	6.665	2	2015 11 21	20:37
273.151	5.47624	152.8399	0.0002	0.000019	0.0053	0.0030	0.000189	0.0079	0.00520	0.0185	0.0296	6.851	2	2015 11 21	21:28
273.150	5.47618	152.8331	0.0002	0.000032	0.0043	0.0030	0.000191	0.0072	0.00520	0.0185	0.0293	6.899	2	2015 11 21	21:41
273.150	5.47617	152.8255	0.0001	0.000022	0.0036	0.0030	0.000189	0.0067	0.00520	0.0185	0.0291	6.923	2	2015 11 21	21:54
273.150	5.47623	152.8257	0.0002	0.000032	0.0040	0.0030	0.000191	0.0070	0.00520	0.0185	0.0292	6.933	2	2015 11 21	22:07
273.151	5.47631	152.8177	0.0003	0.000022	0.0037	0.0030	0.000189	0.0068	0.00520	0.0185	0.0291	6.939	2	2015 11 21	22:20
273.151	5.47632	152.8219	0.0001	0.000021	0.0021	0.0030	0.000189	0.0059	0.00520	0.0185	0.0288	6.950	2	2015 11 21	22:33
273.149	5.48098	152.9702	0.0003	0.000028	0.0082	0.0030	0.000190	0.0103	0.00520	0.0185	0.0308	7.108	2	2015 11 21	23:24
273.151	5.48110	152.9614	0.0004	0.000040	0.0067	0.0030	0.000192	0.0091	0.00520	0.0185	0.0302	7.163	2	2015 11 21	23:37
273.152	5.48115	152.9500	0.0002	0.000016	0.0037	0.0030	0.000189	0.0068	0.00520	0.0185	0.0291	7.197	2	2015 11 21	23:50
273.152	5.48107	152.9621	0.0003	0.000033	0.0014	0.0030	0.000191	0.0057	0.00520	0.0185	0.0287	7.202	2	2015 11 22	00:03
273.150	5.48096	152.9540	0.0003	0.000031	0.0014	0.0030	0.000190	0.0057	0.00520	0.0185	0.0287	7.217	2	2015 11 22	00:16
273.149	5.48092	152.9453	0.0001	0.000016	0.0025	0.0030	0.000189	0.0061	0.00520	0.0185	0.0288	7.227	2	2015 11 22	00:29
273.152	5.48588	153.0834	0.0004	0.000049	0.0056	0.0030	0.000194	0.0081	0.00520	0.0185	0.0298	7.401	2	2015 11 22	01:19
273.150	5.48571	153.0953	0.0004	0.000046	0.0048	0.0030	0.000193	0.0076	0.00520	0.0185	0.0295	7.467	2	2015 11 22	01:32
273.149	5.48566	153.0923	0.0001	0.000011	0.0055	0.0030	0.000188	0.0081	0.00520	0.0185	0.0296	7.488	2	2015 11 22	01:45
273.149	5.48575	153.0824	0.0003	0.000042	0.0074	0.0030	0.000193	0.0097	0.00520	0.0185	0.0305	7.494	2	2015 11 22	01:58
273.151	5.48591	153.0873	0.0006	0.000041	0.0067	0.0031	0.000192	0.0091	0.00520	0.0185	0.0302	7.496	2	2015 11 22	02:11
273.152	5.48597	153.0747	0.0002	0.000022	0.0030	0.0030	0.000189	0.0064	0.00520	0.0185	0.0289	7.498	2	2015 11 22	02:24
273.149	5.49052	153.2226	0.0003	0.000046	0.0075	0.0030	0.000194	0.0097	0.00520	0.0185	0.0306	7.643	2	2015 11 22	03:15
273.151	5.49068	153.2067	0.0005	0.000037	0.0079	0.0030	0.000192	0.0101	0.00520	0.0185	0.0307	7.686	2	2015 11 22	03:28
273.152	5.49075	153.2074	0.0002	0.000013	0.0047	0.0030	0.000189	0.0075	0.00520	0.0185	0.0294	7.723	2	2015 11 22	03:41
273.152	5.49068	153.2026	0.0003	0.000041	0.0015	0.0030	0.000192	0.0057	0.00520	0.0185	0.0288	7.743	2	2015 11 22	03:54
273.150	5.49051	153.1919	0.0005	0.000037	0.0021	0.0030	0.000192	0.0059	0.00520	0.0185	0.0289	7.772	2	2015 11 22	04:07
273.149	5.49045	153.1930	0.0002	0.000019	0.0038	0.0030	0.000189	0.0069	0.00520	0.0185	0.0291	7.780	2	2015 11 22	04:20
273.151	5.49534	153.3490	0.0005	0.000051	0.0032	0.0030	0.000195	0.0065	0.00520	0.0185	0.0291	7.920	2	2015 11 22	05:11
273.149	5.49521	153.3311	0.0003	0.000019	0.0045	0.0030	0.000189	0.0074	0.00520	0.0185	0.0293	8.007	2	2015 11 22	05:24
273.149	5.49519	153.3279	0.0001	0.000033	0.0057	0.0030	0.000191	0.0083	0.00520	0.0185	0.0298	8.017	2	2015 11 22	05:37
273.150	5.49534	153.3337	0.0005	0.000051	0.0068	0.0030	0.000195	0.0092	0.00520	0.0185	0.0303	8.005	2	2015 11 22	05:50
273.151	5.49548	153.3211	0.0003	0.000017	0.0046	0.0030	0.000189	0.0074	0.00520	0.0185	0.0293	7.988	2	2015 11 22	06:03
273.152	5.49546	153.3204	0.0001	0.000033	0.0013	0.0030	0.000191	0.0057	0.00520	0.0185	0.0287	7.997	2	2015 11 22	06:16
273.149	5.50001	153.4654	0.0003	0.000049	0.0081	0.0030	0.000195	0.0102	0.00520	0.0186	0.0309	8.136	2	2015 11 22	07:06
273.151	5.50021	153.4655	0.0006	0.000056	0.0080	0.0031	0.000196	0.0102	0.00520	0.0186	0.0309	8.178	2	2015 11 22	07:19
273.153	5.50031	153.4453	0.0002	0.000022	0.0035	0.0030	0.000189	0.0067	0.00520	0.0186	0.0290	8.212	2	2015 11 22	07:32
273.152	5.50020	153.4427	0.0004	0.000046	0.0009	0.0030	0.000194	0.0056	0.00520	0.0186	0.0288	8.243	2	2015 11 22	07:45
273.150	5.49999	153.4485	0.0005	0.000042	0.0015	0.0030	0.000193	0.0057	0.00520	0.0186	0.0288	8.244	2	2015 11 22	07:58
273.149	5.49987	153.4503	0.0003	0.000016	0.0036	0.0030	0.000189	0.0067	0.00520	0.0186	0.0291	8.223	2	2015 11 22	08:11
273.152	5.50489	153.5985	0.0005	0.000041	0.0024	0.0030	0.000193	0.0061	0.00520	0.0186	0.0289	8.335	2	2015 11 22	09:02
273.150	5.50471	153.5986	0.0004	0.000043	0.0034	0.0030	0.000193	0.0066	0.00520	0.0186	0.0291	8.408	2	2015 11 22	09:15
273.149	5.50466	153.5952	0.0001	0.000020	0.0042	0.0030	0.000189	0.0071	0.00520	0.0186	0.0292	8.404	2	2015 11 22	09:28
273.150	5.50479	153.5903	0.0004	0.000051	0.0060	0.0030	0.000195	0.0085	0.00520	0.0186	0.0300	8.385	2	2015 11 22	09:41
273.152	5.50495	153.5814	0.0004	0.000040	0.0054	0.0030	0.000192	0.0080	0.00520	0.0186	0.0297	8.376	2	2015 11 22	09:54
273.152	5.50496	153.5844	0.0001	0.000023	0.0012	0.0030	0.000190	0.0056	0.00520	0.0186	0.0287	8.354	2	2015 11 22	10:07

Table S2. Experimental (p, rho, T, x) data and detailed uncertainty information for isotherms measured on the (0.25019 argon + 0.74981 carbon dioxide) mixture (continued)

test: Ar_CO2_1511g.dat (brief purge prior to test, 30 min equilibration time; 4 replicates per pressure)
chiS [specific magnetic susceptibility] = −0.6015E−08 [m3/kg]

T (K)	p (MPa)	rho (kg.m−3)	sigma_T (K)	sigma_p (MPa)	sigma_rho (kg.m−3)	u(T) (K)	u(p) (MPa)	u(rho) (kg.m−3)	u(MW) (g/mol)	u[rho(x)] (kg.m−3)	U_c/% (k = 2)	m_sorb (mg)	p_trans	date	time
253.154	0.52915	11.1715	0.0002	0.000003	0.0010	0.0030	0.000150	0.0020	0.00520	0.0014	0.0732	−0.035	2	2015 11 22	18:54
253.153	0.52914	11.1733	0.0007	0.000004	0.0005	0.0031	0.000150	0.0016	0.00520	0.0014	0.0698	−0.030	2	2015 11 22	19:07
253.150	0.52914	11.1722	0.0008	0.000005	0.0012	0.0031	0.000150	0.0022	0.00520	0.0014	0.0751	−0.031	2	2015 11 22	19:20
253.149	0.52913	11.1736	0.0002	0.000004	0.0004	0.0030	0.000150	0.0015	0.00520	0.0014	0.0692	−0.031	2	2015 11 22	19:33
253.146	1.11737	24.5491	0.0002	0.000009	0.0004	0.0030	0.000152	0.0019	0.00520	0.0030	0.0410	−0.038	2	2015 11 22	20:42
253.148	1.11735	24.5508	0.0008	0.000007	0.0003	0.0031	0.000152	0.0019	0.00520	0.0030	0.0409	−0.034	2	2015 11 22	20:55
253.151	1.11735	24.5505	0.0007	0.000003	0.0004	0.0031	0.000152	0.0019	0.00520	0.0030	0.0409	−0.034	2	2015 11 22	21:08
253.152	1.11734	24.5457	0.0001	0.000006	0.0007	0.0030	0.000152	0.0020	0.00520	0.0030	0.0414	−0.031	2	2015 11 22	21:21
253.146	1.62884	37.1903	0.0001	0.000016	0.0001	0.0030	0.000154	0.0022	0.00520	0.0045	0.0344	−0.037	2	2015 11 22	22:21
253.148	1.62882	37.1924	0.0007	0.000007	0.0003	0.0031	0.000154	0.0022	0.00520	0.0045	0.0344	−0.033	2	2015 11 22	22:35
253.150	1.62882	37.1925	0.0006	0.000006	0.0005	0.0031	0.000154	0.0023	0.00520	0.0045	0.0346	−0.030	2	2015 11 22	22:48
253.145	2.07506	49.1717	0.0002	0.000007	0.0016	0.0030	0.000156	0.0032	0.00520	0.0059	0.0328	−0.033	2	2015 11 22	23:52
253.148	2.07506	49.1765	0.0009	0.000005	0.0002	0.0031	0.000156	0.0026	0.00520	0.0059	0.0319	−0.027	2	2015 11 23	00:05
253.150	2.07507	49.1761	0.0006	0.000005	0.0006	0.0031	0.000156	0.0027	0.00520	0.0059	0.0320	−0.025	2	2015 11 23	00:18
253.146	2.07502	49.1747	0.0002	0.000005	0.0005	0.0030	0.000156	0.0027	0.00520	0.0059	0.0320	−0.026	2	2015 11 23	01:07
253.148	2.07504	49.1783	0.0006	0.000003	0.0004	0.0031	0.000156	0.0026	0.00520	0.0059	0.0319	−0.026	2	2015 11 23	01:20
253.150	2.07504	49.1737	0.0005	0.000007	0.0004	0.0030	0.000156	0.0026	0.00520	0.0059	0.0319	−0.024	2	2015 11 23	01:33
253.150	2.07505	49.1750	0.0002	0.000007	0.0003	0.0030	0.000156	0.0026	0.00520	0.0059	0.0319	−0.021	2	2015 11 23	01:46
253.145	2.69902	67.8897	0.0002	0.000015	0.0006	0.0030	0.000160	0.0033	0.00520	0.0082	0.0303	−0.027	2	2015 11 23	02:52
253.147	2.69901	67.8907	0.0008	0.000010	0.0010	0.0031	0.000160	0.0034	0.00520	0.0082	0.0304	−0.023	2	2015 11 23	03:05
253.150	2.69903	67.8893	0.0008	0.000004	0.0003	0.0031	0.000160	0.0032	0.00520	0.0082	0.0302	−0.016	2	2015 11 23	03:18
253.151	2.69902	67.8883	0.0001	0.000009	0.0005	0.0030	0.000160	0.0032	0.00520	0.0082	0.0303	−0.017	2	2015 11 23	03:31
253.147	2.80789	71.4474	0.0001	0.000012	0.0011	0.0030	0.000161	0.0035	0.00520	0.0086	0.0303	−0.012	2	2015 11 23	04:22
253.148	2.80788	71.4465	0.0004	0.000007	0.0007	0.0030	0.000161	0.0034	0.00520	0.0086	0.0301	−0.009	2	2015 11 23	04:35
253.149	2.80788	71.4471	0.0003	0.000005	0.0003	0.0030	0.000161	0.0033	0.00520	0.0086	0.0301	−0.005	2	2015 11 23	04:48
253.149	2.80787	71.4457	0.0001	0.000006	0.0005	0.0030	0.000161	0.0033	0.00520	0.0086	0.0301	−0.005	2	2015 11 23	05:01
253.147	2.87468	73.3975	0.0007	0.000028	0.0153	0.0031	0.000164	0.0164	0.00520	0.0089	0.0532	0.776	2	2015 11 23	05:52
253.146	2.87470	73.3805	0.0002	0.000032	0.0163	0.0030	0.000164	0.0174	0.00520	0.0089	0.0555	0.901	2	2015 11 23	06:05
253.148	2.87486	73.3626	0.0008	0.000054	0.0159	0.0031	0.000170	0.0171	0.00520	0.0089	0.0549	1.045	2	2015 11 23	06:18
253.150	2.87497	73.3435	0.0004	0.000011	0.0105	0.0030	0.000162	0.0117	0.00520	0.0089	0.0429	1.188	2	2015 11 23	06:31
253.145	2.91718	74.3699	0.0002	0.000031	0.0192	0.0030	0.000165	0.0204	0.00520	0.0090	0.0619	20.515	2	2015 11 23	07:32
253.147	2.91735	74.3479	0.0009	0.000055	0.0188	0.0031	0.000171	0.0200	0.00520	0.0090	0.0611	17.225	2	2015 11 23	07:45
253.150	2.91750	74.3316	0.0007	0.000018	0.0125	0.0031	0.000163	0.0138	0.00520	0.0090	0.0468	15.107	2	2015 11 23	07:58
253.151	2.91747	74.3175	0.0002	0.000031	0.0067	0.0030	0.000165	0.0081	0.00520	0.0090	0.0361	15.760	2	2015 11 23	08:11
253.146	2.95930	75.3451	0.0001	0.000022	0.0087	0.0030	0.000164	0.0100	0.00520	0.0091	0.0390	33.419	2	2015 11 23	09:10
253.147	2.95943	75.3339	0.0007	0.000046	0.0165	0.0031	0.000168	0.0176	0.00520	0.0091	0.0550	33.930	2	2015 11 23	09:23
253.150	2.95953	75.3293	0.0005	0.000039	0.0112	0.0030	0.000167	0.0125	0.00520	0.0091	0.0439	33.025	2	2015 11 23	09:36
253.150	2.95947	75.3135	0.0003	0.000034	0.0041	0.0030	0.000166	0.0057	0.00520	0.0091	0.0325	32.611	2	2015 11 23	09:49

Table S2. Experimental (p, rho, T, x) data and detailed uncertainty information for isotherms measured on the (0.25019 argon + 0.74981 carbon dioxide) mixture (continued)

T (K)	p (MPa)	rho (kg.m-3)	sigma_T (K)	sigma_p (MPa)	sigma_rho (kg.m-3)	u(T) (K)	u(p) (MPa)	u(rho) (kg.m-3)	u(MW) (g/mol)	u[rho(x)] (kg.m-3)	U_c/% (k = 2)	m_sorb (mg)	p_trans	date	time
# test: Ar_CO2_1511h.dat (brief purge prior to test, 30 min equilibration time; 4 replicates per pressure; fine pressure steps near dew point)															
# chiS [specific magnetic susceptibility] = -0.6015E-08 [m3/kg]															
253.147	0.52125	10.9997	0.0002	0.000004	0.0010	0.0030	0.000150	0.0021	0.00520	0.0013	0.0748	-0.031	2	2015 11 23	13:14
253.148	0.52126	11.0013	0.0006	0.000006	0.0009	0.0031	0.000150	0.0020	0.00520	0.0013	0.0738	-0.029	2	2015 11 23	13:27
253.150	0.52126	11.0018	0.0005	0.000005	0.0009	0.0030	0.000150	0.0019	0.00520	0.0013	0.0733	-0.029	2	2015 11 23	13:40
253.151	0.52127	11.0013	0.0001	0.000004	0.0008	0.0030	0.000150	0.0019	0.00520	0.0013	0.0728	-0.028	2	2015 11 23	13:53
253.147	1.10174	24.1789	0.0001	0.000011	0.0007	0.0030	0.000152	0.0021	0.00520	0.0029	0.0420	-0.036	2	2015 11 23	14:55
253.148	1.10172	24.1818	0.0006	0.000006	0.0009	0.0031	0.000152	0.0022	0.00520	0.0029	0.0423	-0.031	2	2015 11 23	15:08
253.150	1.10172	24.1840	0.0005	0.000002	0.0007	0.0030	0.000152	0.0021	0.00520	0.0029	0.0419	-0.026	2	2015 11 23	15:21
253.151	1.10171	24.1827	0.0002	0.000005	0.0007	0.0030	0.000152	0.0021	0.00520	0.0029	0.0419	-0.028	2	2015 11 23	15:34
253.147	1.60670	36.6225	0.0002	0.000010	0.0012	0.0030	0.000154	0.0027	0.00520	0.0044	0.0356	-0.032	2	2015 11 23	16:37
253.148	1.60668	36.6217	0.0007	0.000005	0.0007	0.0031	0.000154	0.0023	0.00520	0.0044	0.0349	-0.030	2	2015 11 23	16:50
253.151	1.60669	36.6236	0.0006	0.000007	0.0011	0.0031	0.000154	0.0026	0.00520	0.0044	0.0354	-0.026	2	2015 11 23	17:03
253.152	1.60668	36.6210	0.0002	0.000008	0.0006	0.0030	0.000154	0.0023	0.00520	0.0044	0.0348	-0.027	2	2015 11 23	17:16
253.147	2.04795	48.4200	0.0002	0.000014	0.0003	0.0030	0.000156	0.0026	0.00520	0.0059	0.0320	-0.030	2	2015 11 23	18:18
253.149	2.04793	48.4172	0.0007	0.000007	0.0002	0.0031	0.000156	0.0025	0.00520	0.0059	0.0320	-0.027	2	2015 11 23	18:31
253.151	2.04793	48.4214	0.0005	0.000005	0.0002	0.0030	0.000156	0.0026	0.00520	0.0059	0.0320	-0.024	2	2015 11 23	18:44
253.151	2.04792	48.4197	0.0003	0.000007	0.0004	0.0030	0.000156	0.0026	0.00520	0.0059	0.0320	-0.022	2	2015 11 23	18:57
253.148	2.43456	59.6381	0.0001	0.000014	0.0009	0.0030	0.000159	0.0031	0.00520	0.0072	0.0310	-0.026	2	2015 11 23	20:00
253.149	2.43455	59.6374	0.0005	0.000004	0.0002	0.0030	0.000158	0.0029	0.00520	0.0072	0.0308	-0.022	2	2015 11 23	20:13
253.151	2.43456	59.6392	0.0005	0.000006	0.0004	0.0030	0.000158	0.0029	0.00520	0.0072	0.0308	-0.018	2	2015 11 23	20:26
253.151	2.43455	59.6327	0.0002	0.000006	0.0010	0.0030	0.000158	0.0031	0.00520	0.0072	0.0310	-0.016	2	2015 11 23	20:39
253.147	2.66567	66.8250	0.0001	0.000008	0.0026	0.0030	0.000160	0.0044	0.00520	0.0081	0.0316	-0.021	2	2015 11 23	21:41
253.149	2.66566	66.8227	0.0005	0.000010	0.0003	0.0030	0.000160	0.0032	0.00520	0.0081	0.0303	-0.017	2	2015 11 23	21:54
253.150	2.66567	66.8247	0.0003	0.000007	0.0005	0.0030	0.000160	0.0032	0.00520	0.0081	0.0303	-0.015	2	2015 11 23	22:07
253.151	2.66567	66.8225	0.0001	0.000008	0.0006	0.0030	0.000160	0.0032	0.00520	0.0081	0.0303	-0.013	2	2015 11 23	22:20
253.149	2.77396	70.3326	0.0002	0.000006	0.0004	0.0030	0.000160	0.0033	0.00520	0.0085	0.0301	-0.010	2	2015 11 23	23:21
253.150	2.77395	70.3298	0.0002	0.000005	0.0008	0.0030	0.000160	0.0034	0.00520	0.0085	0.0302	-0.010	2	2015 11 23	23:34
253.150	2.77395	70.3315	0.0000	0.000003	0.0004	0.0030	0.000160	0.0033	0.00520	0.0085	0.0301	-0.008	2	2015 11 23	23:47
253.150	2.77394	70.3310	0.0002	0.000008	0.0008	0.0030	0.000161	0.0034	0.00520	0.0085	0.0302	-0.009	2	2015 11 24	00:00
253.150	2.80925	71.4977	0.0001	0.000009	0.0002	0.0030	0.000161	0.0033	0.00520	0.0086	0.0301	-0.003	2	2015 11 24	01:01
253.150	2.80923	71.4939	0.0002	0.000011	0.0002	0.0030	0.000161	0.0033	0.00520	0.0086	0.0301	-0.001	2	2015 11 24	01:14
253.149	2.80923	71.4954	0.0002	0.000006	0.0005	0.0030	0.000161	0.0033	0.00520	0.0086	0.0301	0.000	2	2015 11 24	01:27
253.149	2.80922	71.4968	0.0001	0.000006	0.0003	0.0030	0.000161	0.0033	0.00520	0.0086	0.0301	0.001	2	2015 11 24	01:40
253.149	2.82662	72.0742	0.0003	0.000009	0.0005	0.0030	0.000161	0.0033	0.00520	0.0087	0.0301	0.015	2	2015 11 24	02:41
253.148	2.82660	72.0694	0.0001	0.000007	0.0008	0.0030	0.000161	0.0034	0.00520	0.0087	0.0301	0.010	2	2015 11 24	02:54
253.149	2.82661	72.0694	0.0002	0.000007	0.0003	0.0030	0.000161	0.0033	0.00520	0.0087	0.0300	0.015	2	2015 11 24	03:07
253.150	2.82662	72.0730	0.0003	0.000008	0.0005	0.0030	0.000161	0.0033	0.00520	0.0087	0.0301	0.019	2	2015 11 24	03:20
253.148	2.84328	72.6206	0.0002	0.000009	0.0023	0.0030	0.000161	0.0043	0.00520	0.0088	0.0309	0.304	2	2015 11 24	04:10
253.148	2.84327	72.6174	0.0002	0.000005	0.0022	0.0030	0.000161	0.0042	0.00520	0.0088	0.0308	0.308	2	2015 11 24	04:23
253.149	2.84328	72.6188	0.0003	0.000007	0.0003	0.0030	0.000161	0.0033	0.00520	0.0088	0.0300	0.308	2	2015 11 24	04:36
253.150	2.84329	72.6183	0.0002	0.000008	0.0004	0.0030	0.000161	0.0033	0.00520	0.0088	0.0300	0.298	2	2015 11 24	04:49

T (K)	p (MPa)	rho (kg.m−3)	sigma_T (K)	sigma_p (MPa)	sigma_rho (kg.m−3)	u(T) (K)	u(p) (MPa)	u(rho) (kg.m−3)	u(MW) (g/mol)	u[rho(x)] (kg.m−3)	U_c/% (k = 2)	m_sorb (mg)	p_trans	date	time
# test: Ar_CO2_1511h.dat (continued)															
253.149	2.85339	72.8793	0.0001	0.000008	0.0175	0.0030	0.000161	0.0187	0.00520	0.0088	0.0587	1.367	2	2015 11 24	05:39
253.149	2.85341	72.8436	0.0001	0.000011	0.0153	0.0030	0.000161	0.0165	0.00520	0.0088	0.0535	2.200	2	2015 11 24	05:52
253.149	2.85345	72.8015	0.0002	0.000014	0.0138	0.0030	0.000162	0.0150	0.00520	0.0088	0.0502	2.009	2	2015 11 24	06:05
253.150	2.85348	72.8838	0.0002	0.000008	0.0111	0.0030	0.000161	0.0123	0.00520	0.0088	0.0442	2.319	2	2015 11 24	06:18
253.149	2.86052	72.9841	0.0003	0.000005	0.0096	0.0030	0.000161	0.0108	0.00520	0.0088	0.0412	7.073	2	2015 11 24	07:09
253.148	2.86055	72.9885	0.0001	0.000024	0.0110	0.0030	0.000163	0.0122	0.00520	0.0088	0.0441	7.830	2	2015 11 24	07:22
253.149	2.86063	72.9630	0.0003	0.000021	0.0120	0.0030	0.000163	0.0132	0.00520	0.0088	0.0462	8.719	2	2015 11 24	07:35
253.150	2.86067	72.9373	0.0002	0.000009	0.0192	0.0030	0.000161	0.0203	0.00520	0.0088	0.0627	9.731	2	2015 11 24	07:48
253.148	2.86759	73.1039	0.0003	0.000009	0.0068	0.0030	0.000161	0.0082	0.00520	0.0088	0.0363	22.423	2	2015 11 24	08:39
253.148	2.86762	73.1091	0.0001	0.000027	0.0105	0.0030	0.000164	0.0117	0.00520	0.0088	0.0430	21.260	2	2015 11 24	08:52
253.149	2.86771	73.0664	0.0004	0.000024	0.0101	0.0030	0.000163	0.0114	0.00520	0.0088	0.0424	24.817	2	2015 11 24	09:05
253.150	2.86778	73.0950	0.0003	0.000011	0.0082	0.0030	0.000162	0.0095	0.00520	0.0088	0.0387	22.845	2	2015 11 24	09:18
253.148	2.87471	73.2396	0.0003	0.000012	0.0046	0.0030	0.000162	0.0062	0.00520	0.0089	0.0332	29.098	2	2015 11 24	10:09
253.148	2.87473	73.2492	0.0001	0.000014	0.0113	0.0030	0.000162	0.0126	0.00520	0.0089	0.0447	33.228	2	2015 11 24	10:22
253.149	2.87480	73.2322	0.0003	0.000020	0.0082	0.0030	0.000163	0.0095	0.00520	0.0089	0.0387	31.351	2	2015 11 24	10:35
253.150	2.87485	73.2296	0.0002	0.000009	0.0065	0.0030	0.000162	0.0079	0.00520	0.0089	0.0357	31.128	2	2015 11 24	10:48

Table S2. Experimental (p, rho, T, x) data and detailed uncertainty information for isotherms measured on the (0.25019 argon + 0.74981 carbon dioxide) mixture (continued)

test: Ar_CO2_1511i.dat (brief purge prior to test, 30 min equilibration time; 4 replicates per pressure; fine pressure steps near dew point)
chiS [specific magnetic susceptibility] = −0.6015E−08 [m3/kg]

T (K)	p (MPa)	rho (kg.m−3)	sigma_T (K)	sigma_p (MPa)	sigma_rho (kg.m−3)	u(T) (K)	u(p) (MPa)	u(rho) (kg.m−3)	u(MW) (g/mol)	u[rho(x)] (kg.m−3)	U_c/% (k = 2)	m_sorb (mg)	p_trans	date	time
253.140	0.50184	10.5845	0.0015	0.000024	0.0009	0.0033	0.000152	0.0020	0.00520	0.0013	0.0770	−0.008	2	2015 11 24	12:25
253.146	0.50191	10.5840	0.0018	0.000019	0.0011	0.0035	0.000152	0.0022	0.00520	0.0013	0.0786	−0.014	2	2015 11 24	12:38
253.152	0.50198	10.5815	0.0002	0.000011	0.0014	0.0030	0.000151	0.0024	0.00520	0.0013	0.0805	−0.021	2	2015 11 24	13:00
253.150	0.50199	10.5829	0.0008	0.000005	0.0004	0.0031	0.000150	0.0015	0.00520	0.0013	0.0725	−0.021	2	2015 11 24	13:13
253.150	1.07588	23.5662	0.0006	0.000015	0.0007	0.0031	0.000152	0.0020	0.00520	0.0029	0.0426	−0.030	2	2015 11 24	14:16
253.149	1.07586	23.5726	0.0002	0.000008	0.0010	0.0030	0.000152	0.0023	0.00520	0.0029	0.0433	−0.028	2	2015 11 24	14:29
253.149	1.07585	23.5692	0.0004	0.000005	0.0007	0.0030	0.000152	0.0020	0.00520	0.0029	0.0425	−0.026	2	2015 11 24	14:42
253.151	1.07585	23.5685	0.0005	0.000006	0.0008	0.0030	0.000152	0.0021	0.00520	0.0029	0.0427	−0.026	2	2015 11 24	14:55
253.147	1.57550	35.8234	0.0002	0.000013	0.0003	0.0030	0.000154	0.0022	0.00520	0.0043	0.0349	−0.028	2	2015 11 24	15:57
253.149	1.57548	35.8240	0.0008	0.000012	0.0013	0.0031	0.000154	0.0028	0.00520	0.0043	0.0362	−0.027	2	2015 11 24	16:10
253.152	1.57548	35.8239	0.0008	0.000009	0.0007	0.0031	0.000154	0.0023	0.00520	0.0043	0.0352	−0.021	2	2015 11 24	16:23
253.153	1.57548	35.8245	0.0001	0.000010	0.0003	0.0030	0.000154	0.0022	0.00520	0.0043	0.0349	−0.022	2	2015 11 24	16:36
253.147	2.01245	47.4321	0.0001	0.000014	0.0014	0.0030	0.000156	0.0031	0.00520	0.0057	0.0330	−0.026	2	2015 11 24	17:40
253.149	2.01244	47.4291	0.0008	0.000004	0.0004	0.0031	0.000155	0.0026	0.00520	0.0057	0.0322	−0.023	2	2015 11 24	17:53
253.152	2.01245	47.4280	0.0006	0.000008	0.0016	0.0031	0.000156	0.0032	0.00520	0.0057	0.0332	−0.022	2	2015 11 24	18:06
253.152	2.01244	47.4299	0.0001	0.000005	0.0016	0.0030	0.000156	0.0032	0.00520	0.0057	0.0332	−0.018	2	2015 11 24	18:19
253.148	2.39502	58.4470	0.0002	0.000013	0.0004	0.0030	0.000158	0.0029	0.00520	0.0071	0.0309	−0.016	2	2015 11 24	19:22
253.149	2.39501	58.4479	0.0006	0.000010	0.0006	0.0031	0.000158	0.0030	0.00520	0.0071	0.0310	−0.015	2	2015 11 24	19:35
253.151	2.39502	58.4467	0.0006	0.000005	0.0004	0.0031	0.000158	0.0029	0.00520	0.0071	0.0309	−0.005	2	2015 11 24	19:48
253.152	2.39502	58.4483	0.0001	0.000011	0.0004	0.0030	0.000158	0.0029	0.00520	0.0071	0.0309	−0.006	2	2015 11 24	20:01
253.148	2.62374	65.4892	0.0001	0.000011	0.0003	0.0030	0.000160	0.0031	0.00520	0.0079	0.0304	−0.010	2	2015 11 24	21:02
253.149	2.62375	65.4894	0.0007	0.000006	0.0006	0.0031	0.000159	0.0032	0.00520	0.0079	0.0304	−0.008	2	2015 11 24	21:15
253.152	2.62376	65.4879	0.0006	0.000002	0.0010	0.0031	0.000159	0.0033	0.00520	0.0079	0.0305	−0.003	2	2015 11 24	21:28
253.153	2.62377	65.4910	0.0001	0.000009	0.0008	0.0030	0.000159	0.0032	0.00520	0.0079	0.0305	−0.000	2	2015 11 24	21:41
253.149	2.83362	72.3039	0.0002	0.000007	0.0005	0.0030	0.000161	0.0034	0.00520	0.0087	0.0301	0.055	2	2015 11 24	22:42
253.150	2.83362	72.2991	0.0006	0.000013	0.0002	0.0031	0.000161	0.0033	0.00520	0.0087	0.0301	0.049	2	2015 11 24	22:55
253.152	2.83363	72.3031	0.0004	0.000013	0.0009	0.0030	0.000161	0.0035	0.00520	0.0087	0.0302	0.056	2	2015 11 24	23:08
253.152	2.83362	72.3015	0.0002	0.000007	0.0004	0.0030	0.000161	0.0033	0.00520	0.0087	0.0300	0.058	2	2015 11 24	23:21
253.150	2.83358	72.2989	0.0004	0.000011	0.0004	0.0030	0.000161	0.0033	0.00520	0.0087	0.0301	0.054	2	2015 11 25	00:20
253.152	2.83361	72.3014	0.0003	0.000008	0.0010	0.0030	0.000161	0.0035	0.00520	0.0087	0.0302	0.054	2	2015 11 25	00:33
253.151	2.83360	72.2989	0.0002	0.000012	0.0003	0.0030	0.000161	0.0033	0.00520	0.0087	0.0300	0.050	2	2015 11 25	00:46
253.150	2.83358	72.3012	0.0004	0.000010	0.0003	0.0030	0.000161	0.0033	0.00520	0.0087	0.0300	0.047	2	2015 11 25	00:59
253.152	2.84825	72.7612	0.0001	0.000013	0.0047	0.0030	0.000162	0.0062	0.00520	0.0088	0.0333	1.017	2	2015 11 25	01:50
253.151	2.84822	72.7568	0.0003	0.000015	0.0032	0.0030	0.000162	0.0050	0.00520	0.0088	0.0317	1.027	2	2015 11 25	02:03
253.150	2.84814	72.7542	0.0004	0.000022	0.0030	0.0030	0.000162	0.0048	0.00520	0.0088	0.0316	1.028	2	2015 11 25	02:16
253.149	2.84811	72.7569	0.0001	0.000007	0.0027	0.0030	0.000161	0.0046	0.00520	0.0088	0.0312	1.039	2	2015 11 25	02:29
253.150	2.85597	72.9067	0.0004	0.000022	0.0130	0.0030	0.000163	0.0142	0.00520	0.0088	0.0484	1.727	2	2015 11 25	03:20
253.149	2.85594	72.8974	0.0002	0.000007	0.0127	0.0030	0.000161	0.0140	0.00520	0.0088	0.0478	2.396	2	2015 11 25	03:33
253.150	2.85600	72.9051	0.0003	0.000026	0.0130	0.0030	0.000163	0.0142	0.00520	0.0088	0.0484	2.184	2	2015 11 25	03:46
253.151	2.85607	72.9005	0.0004	0.000019	0.0108	0.0030	0.000162	0.0120	0.00520	0.0088	0.0437	2.734	2	2015 11 25	03:59
253.149	2.86282	73.0393	0.0003	0.000009	0.0096	0.0030	0.000161	0.0108	0.00520	0.0088	0.0412	15.630	2	2015 11 25	04:50
253.149	2.86289	73.0187	0.0003	0.000033	0.0122	0.0030	0.000164	0.0134	0.00520	0.0088	0.0467	14.973	2	2015 11 25	05:03
253.151	2.86300	73.0093	0.0005	0.000032	0.0114	0.0030	0.000164	0.0126	0.00520	0.0088	0.0449	18.657	2	2015 11 25	05:16
253.152	2.86305	73.0059	0.0002	0.000013	0.0064	0.0030	0.000162	0.0078	0.00520	0.0088	0.0357	18.290	2	2015 11 25	05:29
253.149	2.86971	73.1477	0.0001	0.000020	0.0107	0.0030	0.000162	0.0120	0.00520	0.0088	0.0434	25.200	2	2015 11 25	06:20
253.150	2.86981	73.1426	0.0005	0.000032	0.0110	0.0030	0.000164	0.0122	0.00520	0.0088	0.0440	24.062	2	2015 11 25	06:33
253.151	2.86988	73.1309	0.0003	0.000009	0.0080	0.0030	0.000161	0.0093	0.00520	0.0088	0.0383	27.338	2	2015 11 25	06:46
253.152	2.86986	73.1187	0.0002	0.000011	0.0042	0.0030	0.000162	0.0058	0.00520	0.0088	0.0328	26.635	2	2015 11 25	06:59

T (K)	p (MPa)	rho (kg.m-3)	sigma_T (K)	sigma_p (MPa)	sigma_rho (kg.m-3)	u(T) (K)	u(p) (MPa)	u(rho) (kg.m-3)	u(MW) (g/mol)	u[rho(x)] (kg.m-3)	U_c/% (k = 2)	m_sorb (mg)	p_trans	date	time
# test: Ar_CO2_1511i.dat (continued)															
253.150	2.87663	73.2713	0.0001	0.000008	0.0068	0.0030	0.000162	0.0082	0.00520	0.0089	0.0362	31.123	2	2015 11 25	07:50
253.150	2.87665	73.2757	0.0001	0.000011	0.0076	0.0030	0.000162	0.0090	0.00520	0.0089	0.0377	32.771	2	2015 11 25	08:03
253.151	2.87667	73.2716	0.0001	0.000008	0.0062	0.0030	0.000162	0.0076	0.00520	0.0089	0.0354	31.764	2	2015 11 25	08:16
253.151	2.87668	73.2693	0.0001	0.000007	0.0049	0.0030	0.000161	0.0064	0.00520	0.0089	0.0335	34.222	2	2015 11 25	08:29
253.150	2.88347	73.4217	0.0003	0.000008	0.0050	0.0030	0.000162	0.0065	0.00520	0.0089	0.0337	35.625	2	2015 11 25	09:20
253.150	2.88347	73.4150	0.0000	0.000012	0.0069	0.0030	0.000162	0.0083	0.00520	0.0089	0.0364	36.989	2	2015 11 25	09:33
253.150	2.88352	73.4065	0.0003	0.000020	0.0089	0.0030	0.000163	0.0102	0.00520	0.0089	0.0399	36.530	2	2015 11 25	09:46
253.151	2.88357	73.4113	0.0003	0.000008	0.0060	0.0030	0.000162	0.0075	0.00520	0.0089	0.0351	38.232	2	2015 11 25	09:59
253.149	2.89029	73.5634	0.0001	0.000016	0.0062	0.0030	0.000162	0.0076	0.00520	0.0089	0.0353	39.728	2	2015 11 25	10:50
253.150	2.89037	73.5589	0.0004	0.000032	0.0086	0.0030	0.000165	0.0099	0.00520	0.0089	0.0394	37.947	2	2015 11 25	11:03
253.151	2.89044	73.5465	0.0003	0.000011	0.0067	0.0030	0.000162	0.0081	0.00520	0.0089	0.0360	42.780	2	2015 11 25	11:16

Table S2. Experimental (p, rho, T, x) data and detailed uncertainty information for isotherms measured on the (0.25019 argon + 0.74981 carbon dioxide) mixture (continued)

test: Ar_CO2_1511k.dat (long evacuation prior to test, 30 min equilibration time; 4 replicates per pressure)
chiS [specific magnetic susceptibility] = −0.6015E−08 [m3/kg]

T (K)	p (MPa)	rho (kg.m−3)	sigma_T (K)	sigma_p (MPa)	sigma_rho (kg.m−3)	u(T) (K)	u(p) (MPa)	u(rho) (kg.m−3)	u(MW) (g/mol)	u[rho(x)] (kg.m−3)	U_c/% (k = 2)	m_sorb (mg)	p_trans	date	time
253.158	0.45934	9.6560	0.0002	0.000005	0.0003	0.0030	0.000150	0.0014	0.00520	0.0012	0.0776	−0.025	2	2015 11 28	17:50
253.158	0.45933	9.6531	0.0005	0.000004	0.0019	0.0030	0.000150	0.0029	0.00520	0.0012	0.0939	−0.024	2	2015 11 28	18:03
253.160	0.45932	9.6552	0.0001	0.000006	0.0004	0.0030	0.000150	0.0015	0.00520	0.0012	0.0783	−0.023	2	2015 11 28	18:16
253.159	0.45932	9.6525	0.0007	0.000005	0.0013	0.0031	0.000150	0.0023	0.00520	0.0012	0.0859	−0.023	2	2015 11 28	18:29
253.152	0.84678	18.2576	0.0002	0.000009	0.0007	0.0030	0.000151	0.0019	0.00520	0.0022	0.0493	−0.026	2	2015 11 28	19:30
253.152	0.84676	18.2562	0.0001	0.000006	0.0006	0.0030	0.000151	0.0018	0.00520	0.0022	0.0491	−0.026	2	2015 11 28	19:43
253.152	0.84674	18.2569	0.0000	0.000002	0.0013	0.0030	0.000151	0.0024	0.00520	0.0022	0.0520	−0.024	2	2015 11 28	19:56
253.152	0.84673	18.2536	0.0003	0.000005	0.0002	0.0030	0.000151	0.0016	0.00520	0.0022	0.0482	−0.022	2	2015 11 28	20:09
253.148	1.20016	26.5270	0.0003	0.000014	0.0002	0.0030	0.000153	0.0019	0.00520	0.0032	0.0394	−0.028	2	2015 11 28	21:10
253.148	1.20014	26.5258	0.0005	0.000007	0.0009	0.0030	0.000152	0.0022	0.00520	0.0032	0.0403	−0.025	2	2015 11 28	21:23
253.150	1.20014	26.5276	0.0006	0.000003	0.0010	0.0031	0.000152	0.0023	0.00520	0.0032	0.0405	−0.024	2	2015 11 28	21:36
253.151	1.20013	26.5274	0.0002	0.000003	0.0006	0.0030	0.000152	0.0020	0.00520	0.0032	0.0397	−0.022	2	2015 11 28	21:49
253.146	1.81859	42.1634	0.0002	0.000009	0.0004	0.0030	0.000155	0.0024	0.00520	0.0051	0.0332	−0.024	2	2015 11 28	22:53
253.148	1.81858	42.1646	0.0008	0.000004	0.0011	0.0031	0.000154	0.0027	0.00520	0.0051	0.0338	−0.022	2	2015 11 28	23:06
253.150	1.81857	42.1679	0.0005	0.000003	0.0019	0.0030	0.000154	0.0034	0.00520	0.0051	0.0350	−0.017	2	2015 11 28	23:19
253.151	1.81856	42.1642	0.0003	0.000003	0.0003	0.0030	0.000154	0.0024	0.00520	0.0051	0.0331	−0.020	2	2015 11 28	23:32
253.146	2.21554	53.1647	0.0002	0.000009	0.0010	0.0030	0.000157	0.0030	0.00520	0.0064	0.0317	−0.022	2	2015 11 29	00:36
253.148	2.21553	53.1664	0.0006	0.000008	0.0010	0.0031	0.000157	0.0030	0.00520	0.0064	0.0317	−0.023	2	2015 11 29	00:49
253.150	2.21554	53.1684	0.0005	0.000010	0.0003	0.0030	0.000157	0.0027	0.00520	0.0064	0.0314	−0.018	2	2015 11 29	01:02
253.145	2.56437	63.6250	0.0003	0.000016	0.0005	0.0030	0.000160	0.0031	0.00520	0.0077	0.0305	−0.020	2	2015 11 29	02:06
253.147	2.56438	63.6270	0.0010	0.000014	0.0007	0.0032	0.000159	0.0031	0.00520	0.0077	0.0306	−0.017	2	2015 11 29	02:19
253.151	2.56440	63.6242	0.0002	0.000008	0.0001	0.0030	0.000159	0.0030	0.00520	0.0077	0.0305	−0.010	2	2015 11 29	02:39
253.150	2.56438	63.6246	0.0006	0.000010	0.0007	0.0031	0.000159	0.0031	0.00520	0.0077	0.0306	−0.014	2	2015 11 29	02:52
253.148	2.77354	70.3189	0.0003	0.000012	0.0005	0.0030	0.000161	0.0033	0.00520	0.0085	0.0302	−0.007	2	2015 11 29	03:54
253.147	2.77351	70.3171	0.0001	0.000007	0.0005	0.0030	0.000160	0.0033	0.00520	0.0085	0.0301	−0.008	2	2015 11 29	04:07
253.148	2.77352	70.3170	0.0002	0.000008	0.0001	0.0030	0.000161	0.0032	0.00520	0.0085	0.0301	−0.006	2	2015 11 29	04:20
253.149	2.77352	70.3187	0.0003	0.000007	0.0004	0.0030	0.000160	0.0033	0.00520	0.0085	0.0301	−0.004	2	2015 11 29	04:33
253.148	2.77349	70.3171	0.0004	0.000009	0.0012	0.0030	0.000161	0.0035	0.00520	0.0085	0.0304	−0.010	2	2015 11 29	05:32
253.149	2.77351	70.3161	0.0003	0.000006	0.0002	0.0030	0.000160	0.0032	0.00520	0.0085	0.0301	−0.008	2	2015 11 29	05:45
253.149	2.77351	70.3158	0.0001	0.000007	0.0007	0.0030	0.000160	0.0033	0.00520	0.0085	0.0302	−0.008	2	2015 11 29	05:58
253.149	2.77350	70.3172	0.0002	0.000010	0.0006	0.0030	0.000161	0.0033	0.00520	0.0085	0.0302	−0.003	2	2015 11 29	06:11
253.148	2.80677	71.4149	0.0000	0.000011	0.0002	0.0030	0.000161	0.0033	0.00520	0.0086	0.0301	−0.003	2	2015 11 29	07:12
253.148	2.80677	71.4163	0.0001	0.000012	0.0008	0.0030	0.000161	0.0034	0.00520	0.0086	0.0302	−0.001	2	2015 11 29	07:25
253.148	2.80677	71.4149	0.0000	0.000007	0.0009	0.0030	0.000161	0.0034	0.00520	0.0086	0.0302	−0.003	2	2015 11 29	07:38
253.148	2.80677	71.4143	0.0001	0.000008	0.0003	0.0030	0.000161	0.0033	0.00520	0.0086	0.0301	−0.000	2	2015 11 29	07:51
253.148	2.82317	71.9587	0.0001	0.000010	0.0016	0.0030	0.000161	0.0038	0.00520	0.0087	0.0305	0.003	2	2015 11 29	08:42
253.148	2.82317	71.9605	0.0002	0.000013	0.0005	0.0030	0.000161	0.0033	0.00520	0.0087	0.0301	0.003	2	2015 11 29	08:55
253.148	2.82316	71.9586	0.0001	0.000010	0.0003	0.0030	0.000161	0.0033	0.00520	0.0087	0.0301	0.003	2	2015 11 29	09:08
253.148	2.82316	71.9585	0.0001	0.000011	0.0006	0.0030	0.000161	0.0034	0.00520	0.0087	0.0301	0.009	2	2015 11 29	09:21
253.148	2.83930	72.4936	0.0002	0.000010	0.0003	0.0030	0.000161	0.0033	0.00520	0.0088	0.0300	0.028	2	2015 11 29	10:12
253.148	2.83929	72.4955	0.0002	0.000016	0.0006	0.0030	0.000162	0.0034	0.00520	0.0088	0.0301	0.020	2	2015 11 29	10:25
253.147	2.83928	72.4934	0.0001	0.000005	0.0009	0.0030	0.000161	0.0035	0.00520	0.0088	0.0302	0.026	2	2015 11 29	10:38
253.148	2.83929	72.4919	0.0002	0.000009	0.0010	0.0030	0.000161	0.0035	0.00520	0.0088	0.0302	0.029	2	2015 11 29	10:51
253.147	2.85084	72.8284	0.0002	0.000012	0.0081	0.0030	0.000161	0.0094	0.00520	0.0088	0.0386	0.251	2	2015 11 29	11:42
253.147	2.85085	72.8204	0.0001	0.000014	0.0069	0.0030	0.000162	0.0083	0.00520	0.0088	0.0365	−0.528	2	2015 11 29	11:55
253.148	2.85090	72.8215	0.0003	0.000015	0.0060	0.0030	0.000162	0.0074	0.00520	0.0088	0.0351	−0.520	2	2015 11 29	12:08
253.149	2.85094	72.8165	0.0002	0.000007	0.0031	0.0030	0.000161	0.0048	0.00520	0.0088	0.0315	0.359	2	2015 11 29	12:21

test: Ar_CO2_1511k.dat (continued)

T	p	rho	sigma_T	sigma_p	sigma_rho	u(T)	u(p)	u(rho)	u(MW)	u[rho(x)]	U_c/%	m_sorb	p_trans	date	time
(K)	(MPa)	(kg.m-3)	(K)	(MPa)	(kg.m-3)	(K)	(MPa)	(kg.m-3)	(g/mol)	(kg.m-3)	(k = 2)	(mg)			
253.147	2.85779	72.9603	0.0001	0.000010	0.0099	0.0030	0.000161	0.0112	0.00520	0.0088	0.0419	-1.046	2	2015 11 29	13:12
253.148	2.85785	72.9689	0.0002	0.000022	0.0099	0.0030	0.000163	0.0112	0.00520	0.0088	0.0420	-0.044	2	2015 11 29	13:25
253.149	2.85792	72.9620	0.0003	0.000017	0.0075	0.0030	0.000162	0.0088	0.00520	0.0088	0.0375	-0.470	2	2015 11 29	13:38
253.149	2.85793	72.9557	0.0003	0.000013	0.0039	0.0030	0.000162	0.0056	0.00520	0.0088	0.0324	0.017	2	2015 11 29	13:51
253.148	2.86459	73.1063	0.0001	0.000008	0.0077	0.0030	0.000161	0.0091	0.00520	0.0088	0.0378	0.056	2	2015 11 29	14:42
253.149	2.86461	73.1075	0.0001	0.000009	0.0072	0.0030	0.000161	0.0086	0.00520	0.0088	0.0370	0.883	2	2015 11 29	14:55
253.149	2.86463	73.0850	0.0001	0.000005	0.0059	0.0030	0.000161	0.0073	0.00520	0.0088	0.0349	-0.121	2	2015 11 29	15:08
253.149	2.86463	73.0984	0.0000	0.000005	0.0048	0.0030	0.000161	0.0063	0.00520	0.0088	0.0334	-0.860	2	2015 11 29	15:21
253.149	2.87126	73.2383	0.0002	0.000013	0.0047	0.0030	0.000162	0.0063	0.00520	0.0089	0.0333	11.936	2	2015 11 29	16:12
253.148	2.87126	73.2455	0.0001	0.000007	0.0053	0.0030	0.000161	0.0068	0.00520	0.0089	0.0341	5.749	2	2015 11 29	16:25
253.148	2.87128	73.2430	0.0001	0.000016	0.0067	0.0030	0.000162	0.0081	0.00520	0.0089	0.0361	12.237	2	2015 11 29	16:38
253.149	2.87133	73.2354	0.0002	0.000013	0.0061	0.0030	0.000162	0.0075	0.00520	0.0089	0.0352	11.182	2	2015 11 29	16:51
253.148	2.87787	73.3813	0.0002	0.000012	0.0049	0.0030	0.000162	0.0064	0.00520	0.0089	0.0335	18.690	2	2015 11 29	17:42
253.148	2.87790	73.3825	0.0002	0.000021	0.0077	0.0030	0.000163	0.0090	0.00520	0.0089	0.0377	20.872	2	2015 11 29	17:55
253.149	2.87798	73.3844	0.0004	0.000026	0.0075	0.0030	0.000163	0.0088	0.00520	0.0089	0.0375	19.027	2	2015 11 29	18:08
253.150	2.87804	73.3702	0.0003	0.000007	0.0050	0.0030	0.000161	0.0065	0.00520	0.0089	0.0336	18.064	2	2015 11 29	18:21
253.147	2.88450	73.5301	0.0002	0.000011	0.0062	0.0030	0.000162	0.0076	0.00520	0.0089	0.0353	25.927	2	2015 11 29	19:12
253.148	2.88456	73.5258	0.0004	0.000029	0.0083	0.0030	0.000164	0.0096	0.00520	0.0089	0.0388	25.689	2	2015 11 29	19:25
253.149	2.88466	73.5224	0.0004	0.000025	0.0073	0.0030	0.000163	0.0087	0.00520	0.0089	0.0371	27.715	2	2015 11 29	19:38
253.150	2.88469	73.5188	0.0002	0.000008	0.0029	0.0030	0.000162	0.0047	0.00520	0.0089	0.0313	26.110	2	2015 11 29	19:51
253.147	2.89116	73.6711	0.0001	0.000019	0.0077	0.0030	0.000163	0.0091	0.00520	0.0089	0.0377	27.676	2	2015 11 29	20:42
253.148	2.89126	73.6629	0.0005	0.000034	0.0087	0.0030	0.000165	0.0100	0.00520	0.0089	0.0395	28.680	2	2015 11 29	20:55
253.150	2.89134	73.6608	0.0004	0.000010	0.0055	0.0030	0.000162	0.0070	0.00520	0.0089	0.0343	31.942	2	2015 11 29	21:08
253.150	2.89132	73.6639	0.0002	0.000023	0.0007	0.0030	0.000163	0.0035	0.00520	0.0089	0.0301	27.239	2	2015 11 29	21:21
253.148	2.89784	73.8128	0.0000	0.000007	0.0060	0.0030	0.000162	0.0074	0.00520	0.0089	0.0349	32.774	2	2015 11 29	22:12
253.149	2.89788	73.8004	0.0002	0.000015	0.0068	0.0030	0.000162	0.0082	0.00520	0.0089	0.0363	33.050	2	2015 11 29	22:25
253.150	2.89792	73.8079	0.0003	0.000010	0.0052	0.0030	0.000162	0.0067	0.00520	0.0089	0.0338	31.399	2	2015 11 29	22:38
253.150	2.89793	73.7973	0.0000	0.000007	0.0026	0.0030	0.000162	0.0045	0.00520	0.0089	0.0311	34.640	2	2015 11 29	22:51
253.148	2.90443	73.9545	0.0002	0.000005	0.0044	0.0030	0.000162	0.0060	0.00520	0.0089	0.0328	34.950	2	2015 11 29	23:42
253.148	2.90446	73.9498	0.0002	0.000013	0.0069	0.0030	0.000162	0.0083	0.00520	0.0089	0.0363	34.363	2	2015 11 29	23:55
253.149	2.90451	73.9428	0.0003	0.000008	0.0053	0.0030	0.000162	0.0068	0.00520	0.0089	0.0340	34.782	2	2015 11 30	00:08
253.150	2.90453	73.9393	0.0001	0.000004	0.0033	0.0030	0.000162	0.0050	0.00520	0.0089	0.0316	37.021	2	2015 11 30	00:21
253.148	2.91099	74.1004	0.0002	0.000008	0.0052	0.0030	0.000162	0.0067	0.00520	0.0090	0.0338	36.142	2	2015 11 30	01:12
253.148	2.91107	74.0907	0.0004	0.000031	0.0083	0.0030	0.000165	0.0096	0.00520	0.0090	0.0387	38.176	2	2015 11 30	01:25
253.150	2.91115	74.0858	0.0004	0.000009	0.0058	0.0030	0.000162	0.0072	0.00520	0.0090	0.0346	38.578	2	2015 11 30	01:38
253.150	2.91114	74.0876	0.0001	0.000015	0.0016	0.0030	0.000162	0.0039	0.00520	0.0090	0.0304	39.661	2	2015 11 30	01:51
253.149	2.91764	74.2342	0.0001	0.000011	0.0058	0.0030	0.000162	0.0072	0.00520	0.0090	0.0346	38.898	2	2015 11 30	02:42
253.149	2.91767	74.2349	0.0001	0.000011	0.0063	0.0030	0.000162	0.0077	0.00520	0.0090	0.0353	38.624	2	2015 11 30	02:55
253.149	2.91768	74.2365	0.0001	0.000008	0.0039	0.0030	0.000162	0.0055	0.00520	0.0090	0.0322	37.498	2	2015 11 30	03:08
253.149	2.91766	74.2256	0.0000	0.000010	0.0025	0.0030	0.000162	0.0045	0.00520	0.0090	0.0310	38.368	2	2015 11 30	03:21
253.149	2.92425	74.3833	0.0000	0.000009	0.0048	0.0030	0.000162	0.0064	0.00520	0.0090	0.0333	41.522	2	2015 11 30	04:12
253.149	2.92427	74.3836	0.0001	0.000007	0.0052	0.0030	0.000162	0.0068	0.00520	0.0090	0.0338	42.163	2	2015 11 30	04:25
253.150	2.92428	74.3804	0.0001	0.000007	0.0041	0.0030	0.000162	0.0058	0.00520	0.0090	0.0325	38.644	2	2015 11 30	04:38
253.150	2.92426	74.3778	0.0001	0.000014	0.0022	0.0030	0.000162	0.0042	0.00520	0.0090	0.0308	38.826	2	2015 11 30	04:51
253.148	2.93076	74.5227	0.0001	0.000011	0.0057	0.0030	0.000162	0.0072	0.00520	0.0090	0.0344	39.826	2	2015 11 30	05:42
253.149	2.93081	74.5187	0.0003	0.000017	0.0067	0.0030	0.000163	0.0081	0.00520	0.0090	0.0359	41.324	2	2015 11 30	05:55
253.150	2.93085	74.5192	0.0002	0.000010	0.0046	0.0030	0.000162	0.0061	0.00520	0.0090	0.0330	44.520	2	2015 11 30	06:08
253.150	2.93084	74.5170	0.0001	0.000008	0.0032	0.0030	0.000162	0.0050	0.00520	0.0090	0.0315	43.078	2	2015 11 30	06:21
253.149	2.93733	74.6650	0.0001	0.000008	0.0055	0.0030	0.000162	0.0070	0.00520	0.0090	0.0342	42.157	2	2015 11 30	07:12
253.149	2.93735	74.6583	0.0002	0.000013	0.0064	0.0030	0.000162	0.0078	0.00520	0.0090	0.0354	45.618	2	2015 11 30	07:25
253.149	2.93737	74.6648	0.0002	0.000002	0.0045	0.0030	0.000162	0.0061	0.00520	0.0090	0.0329	42.969	2	2015 11 30	07:38
253.149	2.93737	74.6527	0.0001	0.000007	0.0028	0.0030	0.000162	0.0047	0.00520	0.0090	0.0312	41.409	2	2015 11 30	07:51
253.148	2.94385	74.8099	0.0002	0.000007	0.0038	0.0030	0.000162	0.0055	0.00520	0.0090	0.0321	45.691	2	2015 11 30	08:42
253.148	2.94386	74.8064	0.0000	0.000008	0.0052	0.0030	0.000162	0.0067	0.00520	0.0090	0.0338	47.012	2	2015 11 30	08:55
253.149	2.94389	74.8010	0.0002	0.000015	0.0051	0.0030	0.000163	0.0066	0.00520	0.0090	0.0336	45.437	2	2015 11 30	09:08
253.149	2.94389	74.7943	0.0001	0.000006	0.0038	0.0030	0.000162	0.0055	0.00520	0.0090	0.0321	44.635	2	2015 11 30	09:21

Table S3. Experimental (p, rho, T, x) data and detailed uncertainty information for isotherms measured on the (0.49896 argon + 0.50104 carbon dioxide) mixture

```
# component(s): argon                        mole frac:    0.498959
#              CO2                                         0.501041
#
# molar mass:          41.982978
# nc =  2
# Uncertainty factors [all for standard (k = 1) errors]:
# state point:
#   standard uncertainty in T/K:                           0.0030
#   (p_trans = 1): zero offset in p/kPa                    1.000
#                  relative uncertainty in p/ppm:         26.0
#   (p_trans = 2): zero offset in p/kPa                    0.150
#                  relative uncertainty in p/ppm:         20.0
#   (p_trans = 3): zero offset in p/kPa                    0.030
#                  relative uncertainty in p/ppm:         20.0
#   hydrostatic head, uncertainty in L/V height (m):       0.050
# sinker volume
#   u(V_sinker)/ppm at Tref                               28.0
#   u(V_sinker)_temperature coeff [ppm/K]                  0.375
#   u(V_sinker)_pressure coeff [ppm/MPa]                   0.625
# density [except for V_sinker]
#   u_rho/[kg/m^3]                                         0.00100
# sample
#   sample purity, u(rho) [relative error]:                0.000000
#   gravimetric prep [uncertainty in MW]                   0.000022
#   sorption of sample [uncertainty in MW]                 0.005200
```

```
# test:  Ar_CO2_1507mn.dat (long evacuation prior to test; 30 min equilibration time; 3 replicates per pressure)
# chiS [specific magnetic susceptibility] = -0.6035E-08 [m3/kg]
```

T (K)	p (MPa)	rho (kg.m-3)	sigma_T (K)	sigma_p (MPa)	sigma_rho (kg.m-3)	u(T) (K)	u(p) (MPa)	u(rho) (kg.m-3)	u(MW) (g/mol)	u[rho(x)] (kg.m-3)	U_c/% (k = 2)	m_sorb (mg)	p_trans	date	time
253.147	0.49795	10.1388	0.0001	0.000004	0.0003	0.0030	0.000150	0.0015	0.00520	0.0013	0.0725	-0.006	2	2015 07 27	18:24
253.149	0.49795	10.1400	0.0009	0.000004	0.0002	0.0031	0.000150	0.0014	0.00520	0.0013	0.0716	-0.005	2	2015 07 27	18:37
253.153	0.49796	10.1369	0.0009	0.000006	0.0001	0.0031	0.000150	0.0013	0.00520	0.0013	0.0715	-0.006	2	2015 07 27	18:50
253.148	1.10079	22.9779	0.0002	0.000010	0.0002	0.0030	0.000152	0.0018	0.00520	0.0028	0.0411	-0.012	2	2015 07 27	19:55
253.151	1.10078	22.9753	0.0009	0.000005	0.0001	0.0031	0.000152	0.0017	0.00520	0.0028	0.0410	-0.010	2	2015 07 27	20:08
253.154	1.10077	22.9747	0.0007	0.000005	0.0002	0.0031	0.000152	0.0018	0.00520	0.0028	0.0411	-0.007	2	2015 07 27	20:21
253.148	2.09470	45.7183	0.0002	0.000020	0.0007	0.0030	0.000157	0.0026	0.00520	0.0057	0.0320	-0.012	2	2015 07 27	21:28
253.150	2.09467	45.7198	0.0010	0.000014	0.0006	0.0032	0.000156	0.0026	0.00520	0.0057	0.0319	-0.002	2	2015 07 27	21:41
253.154	2.09468	45.7178	0.0008	0.000007	0.0002	0.0031	0.000156	0.0025	0.00520	0.0057	0.0317	-0.002	2	2015 07 27	21:54
253.148	3.04042	69.5659	0.0004	0.000015	0.0009	0.0030	0.000163	0.0034	0.00520	0.0086	0.0296	-0.008	2	2015 07 27	23:05
253.151	3.04041	69.5650	0.0011	0.000009	0.0008	0.0032	0.000162	0.0034	0.00520	0.0086	0.0295	0.002	2	2015 07 27	23:19
253.147	3.99677	96.4398	0.0005	0.000010	0.0014	0.0030	0.000171	0.0044	0.00520	0.0119	0.0287	0.003	2	2015 07 28	00:35
253.151	3.99680	96.4354	0.0011	0.000016	0.0010	0.0032	0.000171	0.0042	0.00520	0.0119	0.0286	0.006	2	2015 07 28	00:49
253.148	4.50395	112.0796	0.0003	0.000013	0.0017	0.0030	0.000176	0.0050	0.00520	0.0139	0.0285	0.006	2	2015 07 28	01:59
253.150	4.50397	112.0774	0.0011	0.000011	0.0009	0.0032	0.000176	0.0047	0.00520	0.0139	0.0284	0.009	2	2015 07 28	02:12
253.154	4.50404	112.0781	0.0004	0.000017	0.0012	0.0030	0.000177	0.0048	0.00520	0.0139	0.0284	0.010	2	2015 07 28	02:27
253.149	4.54852	113.5052	0.0002	0.000014	0.0003	0.0030	0.000177	0.0046	0.00520	0.0141	0.0283	0.001	2	2015 07 28	03:18
253.151	4.54855	113.5072	0.0007	0.000018	0.0004	0.0031	0.000177	0.0046	0.00520	0.0141	0.0283	0.010	2	2015 07 28	03:31
253.153	4.54857	113.5056	0.0005	0.000013	0.0009	0.0030	0.000177	0.0047	0.00520	0.0141	0.0283	0.007	2	2015 07 28	03:44
253.149	4.59209	114.9106	0.0002	0.000018	0.0004	0.0030	0.000178	0.0047	0.00520	0.0142	0.0283	0.011	2	2015 07 28	04:35
253.151	4.59213	114.9109	0.0006	0.000011	0.0006	0.0031	0.000177	0.0047	0.00520	0.0142	0.0283	0.008	2	2015 07 28	04:48
253.153	4.59215	114.9098	0.0004	0.000010	0.0002	0.0030	0.000177	0.0047	0.00520	0.0142	0.0283	0.012	2	2015 07 28	05:01
253.149	4.63469	116.2907	0.0001	0.000012	0.0006	0.0030	0.000178	0.0048	0.00520	0.0144	0.0283	0.005	2	2015 07 28	05:52
253.150	4.63471	116.2917	0.0006	0.000015	0.0003	0.0031	0.000178	0.0047	0.00520	0.0144	0.0283	0.007	2	2015 07 28	06:05
253.153	4.63475	116.2917	0.0006	0.000025	0.0003	0.0031	0.000179	0.0047	0.00520	0.0144	0.0283	0.012	2	2015 07 28	06:18

test: Ar_CO2_1507m.dat (continued)

T (K)	p (MPa)	rho (kg.m-3)	sigma_T (K)	sigma_p (MPa)	sigma_rho (kg.m-3)	u(T) (K)	u(p) (MPa)	u(rho) (kg.m-3)	u(MW) (g/mol)	u[rho(x)] (kg.m-3)	U_c/% (k = 2)	m_sorb (mg)	p_trans	date	time
253.149	4.67637	117.6532	0.0001	0.000022	0.0009	0.0030	0.000179	0.0049	0.00520	0.0146	0.0283	0.006	2	2015 07 28	07:09
253.150	4.67640	117.6527	0.0006	0.000020	0.0004	0.0031	0.000179	0.0048	0.00520	0.0146	0.0283	0.002	2	2015 07 28	07:22
253.152	4.67642	117.6538	0.0005	0.000014	0.0007	0.0030	0.000178	0.0048	0.00520	0.0146	0.0283	0.014	2	2015 07 28	07:35
253.149	4.71709	118.9917	0.0002	0.000014	0.0001	0.0030	0.000179	0.0048	0.00520	0.0147	0.0282	0.010	2	2015 07 28	08:26
253.150	4.71710	118.9908	0.0005	0.000015	0.0007	0.0030	0.000179	0.0049	0.00520	0.0147	0.0283	0.013	2	2015 07 28	08:39
253.152	4.71716	118.9902	0.0007	0.000025	0.0007	0.0031	0.000180	0.0049	0.00520	0.0147	0.0283	0.022	2	2015 07 28	08:52
253.150	4.75699	120.3082	0.0002	0.000011	0.0008	0.0030	0.000179	0.0049	0.00520	0.0149	0.0283	0.014	2	2015 07 28	09:43
253.150	4.75701	120.3091	0.0005	0.000025	0.0002	0.0030	0.000180	0.0048	0.00520	0.0149	0.0283	0.013	2	2015 07 28	09:56
253.152	4.75706	120.3060	0.0004	0.000020	0.0007	0.0030	0.000180	0.0049	0.00520	0.0149	0.0283	0.010	2	2015 07 28	10:09
253.149	4.79580	121.5988	0.0001	0.000023	0.0009	0.0030	0.000180	0.0050	0.00520	0.0151	0.0283	0.013	2	2015 07 28	10:59
253.150	4.79584	121.5967	0.0006	0.000025	0.0002	0.0031	0.000181	0.0049	0.00520	0.0151	0.0283	0.006	2	2015 07 28	11:12
253.153	4.79589	121.5974	0.0006	0.000014	0.0004	0.0031	0.000180	0.0049	0.00520	0.0151	0.0282	0.018	2	2015 07 28	11:25
253.149	4.83405	122.8772	0.0002	0.000007	0.0003	0.0030	0.000180	0.0049	0.00520	0.0152	0.0282	0.008	2	2015 07 28	12:15
253.150	4.83408	122.8751	0.0005	0.000018	0.0009	0.0030	0.000180	0.0050	0.00520	0.0152	0.0283	0.015	2	2015 07 28	12:28
253.152	4.83413	122.8751	0.0007	0.000039	0.0008	0.0031	0.000184	0.0050	0.00520	0.0152	0.0283	0.010	2	2015 07 28	12:41
253.150	4.87156	124.1325	0.0003	0.000022	0.0009	0.0030	0.000181	0.0051	0.00520	0.0154	0.0283	0.008	2	2015 07 28	13:32
253.150	4.87158	124.1330	0.0006	0.000021	0.0004	0.0031	0.000181	0.0050	0.00520	0.0154	0.0282	0.008	2	2015 07 28	13:45
253.152	4.87164	124.1345	0.0006	0.000022	0.0003	0.0031	0.000181	0.0050	0.00520	0.0154	0.0282	0.019	2	2015 07 28	13:58
253.149	4.90826	125.3740	0.0002	0.000009	0.0001	0.0030	0.000181	0.0050	0.00520	0.0155	0.0282	0.013	2	2015 07 28	14:49
253.150	4.90829	125.3739	0.0005	0.000024	0.0008	0.0030	0.000182	0.0051	0.00520	0.0155	0.0283	0.012	2	2015 07 28	15:02
253.152	4.90834	125.3733	0.0006	0.000014	0.0004	0.0031	0.000181	0.0050	0.00520	0.0155	0.0282	0.021	2	2015 07 28	15:15
253.150	4.94421	126.5927	0.0002	0.000016	0.0003	0.0030	0.000182	0.0050	0.00520	0.0157	0.0282	0.013	2	2015 07 28	16:05
253.150	4.94426	126.5952	0.0005	0.000064	0.0028	0.0030	0.000192	0.0059	0.00520	0.0157	0.0288	0.027	2	2015 07 28	16:18
253.152	4.94436	126.5972	0.0005	0.000017	0.0005	0.0030	0.000182	0.0051	0.00520	0.0157	0.0282	0.018	2	2015 07 28	16:31
253.154	4.98309	127.9175	0.0002	0.000014	0.0004	0.0030	0.000182	0.0051	0.00520	0.0158	0.0282	0.026	2	2015 07 28	17:44
253.153	4.98298	127.9156	0.0008	0.000068	0.0016	0.0031	0.000194	0.0054	0.00520	0.0158	0.0286	0.025	2	2015 07 28	17:57
253.150	4.98286	127.9148	0.0007	0.000022	0.0003	0.0031	0.000183	0.0051	0.00520	0.0158	0.0282	0.025	2	2015 07 28	18:10
253.152	5.01734	129.0984	0.0001	0.000017	0.0004	0.0030	0.000182	0.0051	0.00520	0.0160	0.0282	0.035	2	2015 07 28	19:01
253.151	5.01730	129.0951	0.0006	0.000023	0.0003	0.0031	0.000183	0.0051	0.00520	0.0160	0.0282	0.021	2	2015 07 28	19:14
253.149	5.01725	129.0949	0.0003	0.000015	0.0003	0.0030	0.000182	0.0051	0.00520	0.0160	0.0282	0.025	2	2015 07 28	19:27
253.152	5.05099	130.2552	0.0003	0.000014	0.0006	0.0030	0.000183	0.0052	0.00520	0.0161	0.0282	0.049	2	2015 07 28	20:17
253.150	5.05093	130.2563	0.0003	0.000019	0.0003	0.0030	0.000183	0.0052	0.00520	0.0161	0.0282	0.048	2	2015 07 28	20:30
253.149	5.05091	130.2555	0.0002	0.000020	0.0004	0.0030	0.000183	0.0052	0.00520	0.0161	0.0282	0.049	2	2015 07 28	20:43
253.151	5.08337	131.3504	0.0004	0.000023	0.0018	0.0030	0.000184	0.0056	0.00520	0.0163	0.0284	0.260	2	2015 07 28	21:33
253.150	5.08329	131.3482	0.0003	0.000022	0.0020	0.0030	0.000184	0.0056	0.00520	0.0163	0.0284	0.264	2	2015 07 28	21:46
253.149	5.08326	131.3480	0.0001	0.000014	0.0009	0.0030	0.000183	0.0053	0.00520	0.0163	0.0282	0.267	2	2015 07 28	21:59
253.151	5.11139	132.1356	0.0005	0.000041	0.0177	0.0030	0.000188	0.0192	0.00520	0.0164	0.0398	1.460	2	2015 07 28	22:50
253.149	5.11130	132.1141	0.0002	0.000025	0.0141	0.0030	0.000185	0.0157	0.00520	0.0164	0.0360	1.552	2	2015 07 28	23:03
253.149	5.11130	132.1150	0.0002	0.000020	0.0098	0.0030	0.000184	0.0117	0.00520	0.0164	0.0324	1.642	2	2015 07 28	23:16
253.150	5.13692	132.7816	0.0008	0.000047	0.0204	0.0031	0.000189	0.0218	0.00520	0.0164	0.0427	2.401	2	2015 07 29	00:07
253.149	5.13682	132.7684	0.0002	0.000013	0.0196	0.0030	0.000184	0.0210	0.00520	0.0164	0.0417	2.458	2	2015 07 29	00:20
253.149	5.13691	132.7261	0.0004	0.000039	0.0177	0.0030	0.000187	0.0192	0.00520	0.0164	0.0396	2.513	2	2015 07 29	00:33
253.150	5.16186	133.4162	0.0008	0.000044	0.0200	0.0031	0.000189	0.0215	0.00520	0.0165	0.0421	3.212	2	2015 07 29	01:23
253.148	5.16184	133.4300	0.0002	0.000031	0.0220	0.0030	0.000186	0.0234	0.00520	0.0165	0.0443	3.270	2	2015 07 29	01:36
253.150	5.16199	133.3782	0.0006	0.000048	0.0214	0.0031	0.000190	0.0228	0.00520	0.0165	0.0437	3.310	2	2015 07 29	01:49
253.149	5.18649	134.0673	0.0008	0.000038	0.0182	0.0031	0.000188	0.0197	0.00520	0.0166	0.0400	7.669	2	2015 07 29	02:39
253.148	5.18644	134.0666	0.0001	0.000030	0.0199	0.0030	0.000186	0.0213	0.00520	0.0166	0.0418	7.993	2	2015 07 29	02:52
253.149	5.18659	134.0432	0.0007	0.000059	0.0218	0.0031	0.000193	0.0232	0.00520	0.0166	0.0440	3.946	2	2015 07 29	03:05
253.149	5.21075	134.7162	0.0005	0.000027	0.0178	0.0030	0.000186	0.0193	0.00520	0.0167	0.0395	16.662	2	2015 07 29	03:59
253.148	5.21080	134.7148	0.0003	0.000033	0.0185	0.0030	0.000187	0.0200	0.00520	0.0167	0.0402	17.251	2	2015 07 29	04:12
253.150	5.21096	134.6946	0.0007	0.000050	0.0180	0.0031	0.000191	0.0196	0.00520	0.0167	0.0398	18.022	2	2015 07 29	04:25
253.149	5.23472	135.3799	0.0006	0.000032	0.0168	0.0031	0.000187	0.0184	0.00520	0.0168	0.0384	18.101	2	2015 07 29	05:16
253.148	5.23476	135.3594	0.0003	0.000050	0.0189	0.0030	0.000191	0.0204	0.00520	0.0168	0.0406	21.234	2	2015 07 29	05:29
253.150	5.23491	135.3118	0.0007	0.000047	0.0178	0.0031	0.000190	0.0193	0.00520	0.0168	0.0395	21.172	2	2015 07 29	05:42

T	p	rho	sigma_T	sigma_p	sigma_rho	u(T)	u(p)	u(rho)	u(MW)	u[rho(x)]	U_c/%	m_sorb	p_trans	date	time
(K)	(MPa)	(kg.m-3)	(K)	(MPa)	(kg.m-3)	(K)	(MPa)	(kg.m-3)	(g/mol)	(kg.m-3)	(k = 2)	(mg)			
# test: Ar_CO2_1507m.dat (continued)															
253.149	5.25836	136.0114	0.0006	0.000030	0.0153	0.0031	0.000187	0.0169	0.00520	0.0168	0.0368	22.947	2	2015 07 29	06:33
253.149	5.25839	135.9932	0.0003	0.000032	0.0185	0.0030	0.000188	0.0200	0.00520	0.0168	0.0400	23.448	2	2015 07 29	06:46
253.150	5.25853	135.9707	0.0006	0.000044	0.0166	0.0031	0.000190	0.0182	0.00520	0.0168	0.0381	24.554	2	2015 07 29	06:59
253.149	5.28159	136.6453	0.0004	0.000032	0.0157	0.0030	0.000188	0.0173	0.00520	0.0169	0.0371	22.576	2	2015 07 29	07:50
253.148	5.28163	136.6239	0.0004	0.000044	0.0199	0.0030	0.000190	0.0214	0.00520	0.0169	0.0415	25.281	2	2015 07 29	08:03
253.151	5.28183	136.6115	0.0009	0.000048	0.0175	0.0031	0.000191	0.0191	0.00520	0.0169	0.0390	24.931	2	2015 07 29	08:16
253.148	5.30444	137.2636	0.0004	0.000025	0.0157	0.0030	0.000187	0.0173	0.00520	0.0170	0.0370	26.654	2	2015 07 29	09:07
253.148	5.30452	137.2378	0.0004	0.000046	0.0172	0.0030	0.000191	0.0188	0.00520	0.0170	0.0386	24.615	2	2015 07 29	09:20
253.150	5.30470	137.2363	0.0008	0.000060	0.0162	0.0031	0.000195	0.0177	0.00520	0.0170	0.0376	26.239	2	2015 07 29	09:33

Table S3. Experimental (p, rho, T, x) data and detailed uncertainty information for isotherms measured on the (0.49896 argon + 0.50104 carbon dioxide) mixture (continued)

test: Ar_CO2_1507o.dat (brief purge prior to test; 30 min equilibration time; 3 replicates per pressure)
chiS [specific magnetic susceptibility] = −0.6035E−08 [m3/kg]

T (K)	p (MPa)	rho (kg.m−3)	sigma_T (K)	sigma_p (MPa)	sigma_rho (kg.m−3)	u(T) (K)	u(p) (MPa)	u(rho) (kg.m−3)	u(MW) (g/mol)	u[rho(x)] (kg.m−3)	U_c/% (k = 2)	m_sorb (mg)	p_trans	date	time
253.146	0.50142	10.2120	0.0002	0.000007	0.0012	0.0030	0.000151	0.0022	0.00520	0.0013	0.0789	−0.002	2	2015 07 29	11:58
253.147	0.50144	10.2121	0.0007	0.000010	0.0012	0.0031	0.000151	0.0022	0.00520	0.0013	0.0791	−0.002	2	2015 07 29	12:11
253.150	0.50147	10.2120	0.0008	0.000007	0.0010	0.0031	0.000151	0.0021	0.00520	0.0013	0.0776	−0.003	2	2015 07 29	12:24
253.147	1.08040	22.5347	0.0002	0.000010	0.0003	0.0030	0.000152	0.0018	0.00520	0.0028	0.0416	−0.011	2	2015 07 29	13:26
253.149	1.08039	22.5350	0.0008	0.000007	0.0003	0.0031	0.000152	0.0018	0.00520	0.0028	0.0416	−0.008	2	2015 07 29	13:39
253.152	1.08038	22.5318	0.0007	0.000005	0.0004	0.0031	0.000152	0.0018	0.00520	0.0028	0.0417	−0.004	2	2015 07 29	13:52
253.147	2.03684	44.3364	0.0005	0.000017	0.0003	0.0030	0.000156	0.0024	0.00520	0.0055	0.0320	−0.007	2	2015 07 29	15:02
253.150	2.03680	44.3362	0.0010	0.000010	0.0004	0.0032	0.000156	0.0025	0.00520	0.0055	0.0320	−0.003	2	2015 07 29	15:15
253.153	2.03679	44.3352	0.0003	0.000008	0.0005	0.0030	0.000156	0.0025	0.00520	0.0055	0.0320	−0.002	2	2015 07 29	15:28
253.147	3.03077	69.3123	0.0001	0.000012	0.0002	0.0030	0.000162	0.0032	0.00520	0.0086	0.0294	−0.007	2	2015 07 29	16:38
253.149	3.03076	69.3093	0.0009	0.000010	0.0008	0.0031	0.000162	0.0034	0.00520	0.0086	0.0296	−0.005	2	2015 07 29	16:51
253.152	3.03076	69.3087	0.0007	0.000012	0.0003	0.0031	0.000162	0.0032	0.00520	0.0086	0.0294	0.003	2	2015 07 29	17:04
253.146	3.98974	96.2303	0.0004	0.000016	0.0009	0.0030	0.000171	0.0042	0.00520	0.0119	0.0286	−0.001	2	2015 07 29	18:18
253.150	3.98977	96.2292	0.0011	0.000022	0.0004	0.0032	0.000172	0.0041	0.00520	0.0119	0.0286	0.007	2	2015 07 29	18:31
253.153	3.98981	96.2289	0.0007	0.000014	0.0006	0.0031	0.000171	0.0041	0.00520	0.0119	0.0286	0.008	2	2015 07 29	18:44
253.148	4.50438	112.0953	0.0002	0.000009	0.0003	0.0030	0.000176	0.0046	0.00520	0.0139	0.0283	0.006	2	2015 07 29	19:49
253.150	4.50436	112.0943	0.0007	0.000013	0.0006	0.0031	0.000176	0.0046	0.00520	0.0139	0.0283	0.008	2	2015 07 29	20:02
253.153	4.50441	112.0944	0.0007	0.000016	0.0002	0.0031	0.000176	0.0046	0.00520	0.0139	0.0283	0.013	2	2015 07 29	20:15
253.148	4.90863	125.3909	0.0003	0.000020	0.0012	0.0030	0.000181	0.0052	0.00520	0.0155	0.0283	0.009	2	2015 07 29	21:24
253.151	4.90868	125.3904	0.0010	0.000038	0.0002	0.0032	0.000184	0.0050	0.00520	0.0155	0.0283	0.017	2	2015 07 29	21:37
253.154	4.90874	125.3897	0.0003	0.000009	0.0006	0.0030	0.000181	0.0050	0.00520	0.0155	0.0282	0.026	2	2015 07 29	21:54
253.150	4.94098	126.4890	0.0003	0.000013	0.0005	0.0030	0.000181	0.0051	0.00520	0.0157	0.0282	0.012	2	2015 07 29	22:45
253.152	4.94102	126.4883	0.0006	0.000021	0.0004	0.0031	0.000182	0.0051	0.00520	0.0157	0.0282	0.020	2	2015 07 29	22:58
253.154	4.94106	126.4895	0.0004	0.000014	0.0007	0.0030	0.000181	0.0051	0.00520	0.0157	0.0282	0.025	2	2015 07 29	23:11
253.150	4.97263	127.5694	0.0001	0.000011	0.0003	0.0030	0.000182	0.0051	0.00520	0.0158	0.0282	0.019	2	2015 07 30	00:02
253.151	4.97266	127.5698	0.0005	0.000017	0.0003	0.0030	0.000182	0.0051	0.00520	0.0158	0.0282	0.022	2	2015 07 30	00:15
253.153	4.97269	127.5672	0.0004	0.000018	0.0004	0.0030	0.000182	0.0051	0.00520	0.0158	0.0282	0.022	2	2015 07 30	00:28
253.150	5.00365	128.6335	0.0001	0.000018	0.0008	0.0030	0.000182	0.0052	0.00520	0.0159	0.0282	0.023	2	2015 07 30	01:19
253.151	5.00366	128.6327	0.0005	0.000018	0.0002	0.0030	0.000182	0.0051	0.00520	0.0159	0.0282	0.026	2	2015 07 30	01:32
253.153	5.00370	128.6322	0.0005	0.000019	0.0003	0.0030	0.000182	0.0051	0.00520	0.0159	0.0282	0.033	2	2015 07 30	01:45
253.150	5.03405	129.6794	0.0003	0.000012	0.0004	0.0030	0.000182	0.0052	0.00520	0.0161	0.0282	0.029	2	2015 07 30	02:35
253.150	5.03405	129.6788	0.0003	0.000006	0.0006	0.0030	0.000182	0.0052	0.00520	0.0161	0.0282	0.030	2	2015 07 30	02:48
253.152	5.03409	129.6786	0.0005	0.000020	0.0002	0.0030	0.000183	0.0051	0.00520	0.0161	0.0282	0.028	2	2015 07 30	03:01
253.150	5.06375	130.7043	0.0006	0.000028	0.0011	0.0031	0.000184	0.0053	0.00520	0.0162	0.0283	0.076	2	2015 07 30	03:51
253.150	5.06373	130.7014	0.0002	0.000019	0.0006	0.0030	0.000183	0.0052	0.00520	0.0162	0.0282	0.073	2	2015 07 30	04:04
253.151	5.06378	130.7022	0.0006	0.000019	0.0003	0.0031	0.000183	0.0052	0.00520	0.0162	0.0282	0.080	2	2015 07 30	04:17
253.150	5.09177	131.6198	0.0005	0.000039	0.0056	0.0030	0.000187	0.0080	0.00520	0.0163	0.0297	0.665	2	2015 07 30	05:08
253.150	5.09174	131.6142	0.0004	0.000013	0.0026	0.0030	0.000183	0.0060	0.00520	0.0163	0.0285	0.687	2	2015 07 30	05:21
253.152	5.09178	131.6183	0.0007	0.000034	0.0007	0.0031	0.000186	0.0053	0.00520	0.0163	0.0283	0.692	2	2015 07 30	05:34
253.149	5.11578	132.2452	0.0005	0.000025	0.0220	0.0030	0.000185	0.0234	0.00520	0.0164	0.0446	1.888	2	2015 07 30	06:25
253.149	5.11582	132.2454	0.0004	0.000036	0.0181	0.0030	0.000186	0.0196	0.00520	0.0164	0.0402	2.050	2	2015 07 30	06:38
253.152	5.11596	132.2166	0.0007	0.000036	0.0131	0.0031	0.000186	0.0148	0.00520	0.0164	0.0352	2.207	2	2015 07 30	06:51
253.150	5.13828	132.8365	0.0006	0.000024	0.0191	0.0031	0.000185	0.0206	0.00520	0.0165	0.0412	2.994	2	2015 07 30	07:41
253.149	5.13834	132.8073	0.0004	0.000034	0.0197	0.0030	0.000186	0.0211	0.00520	0.0164	0.0418	3.055	2	2015 07 30	07:54
253.152	5.13852	132.7929	0.0008	0.000058	0.0164	0.0031	0.000192	0.0179	0.00520	0.0164	0.0383	3.125	2	2015 07 30	08:07

T (K)	p (MPa)	rho (kg.m-3)	sigma_T (K)	sigma_p (MPa)	sigma_rho (kg.m-3)	u(T) (K)	u(p) (MPa)	u(rho) (kg.m-3)	u(MW) (g/mol)	u[rho(x)] (kg.m-3)	U_c/% (k = 2)	m_sorb (mg)	p_trans	date	time
# test: Ar_CO2_1507m.dat (continued)															
253.150	5.16034	133.3999	0.0006	0.000024	0.0167	0.0031	0.000185	0.0183	0.00520	0.0165	0.0385	3.672	2	2015 07 30	08:57
253.149	5.16039	133.3857	0.0003	0.000046	0.0184	0.0030	0.000189	0.0198	0.00520	0.0165	0.0403	3.747	2	2015 07 30	09:10
253.151	5.16058	133.4217	0.0008	0.000062	0.0172	0.0031	0.000194	0.0187	0.00520	0.0165	0.0391	7.822	2	2015 07 30	09:23
253.150	5.18222	134.0201	0.0005	0.000038	0.0160	0.0030	0.000188	0.0176	0.00520	0.0166	0.0377	17.753	2	2015 07 30	10:14
253.149	5.18224	133.9730	0.0003	0.000045	0.0172	0.0030	0.000189	0.0187	0.00520	0.0166	0.0389	16.374	2	2015 07 30	10:27
253.152	5.18242	133.9743	0.0007	0.000044	0.0171	0.0031	0.000189	0.0187	0.00520	0.0166	0.0389	18.241	2	2015 07 30	10:40
253.150	5.20373	134.5787	0.0005	0.000023	0.0147	0.0030	0.000186	0.0163	0.00520	0.0167	0.0364	20.959	2	2015 07 30	11:31
253.150	5.20380	134.5580	0.0003	0.000041	0.0166	0.0030	0.000189	0.0181	0.00520	0.0167	0.0383	21.348	2	2015 07 30	11:44
253.152	5.20398	134.5446	0.0007	0.000046	0.0157	0.0031	0.000190	0.0172	0.00520	0.0167	0.0374	20.648	2	2015 07 30	11:57
253.150	5.22505	135.1418	0.0006	0.000038	0.0132	0.0031	0.000188	0.0149	0.00520	0.0167	0.0350	23.676	2	2015 07 30	12:47
253.149	5.22505	135.1386	0.0002	0.000035	0.0149	0.0030	0.000188	0.0165	0.00520	0.0167	0.0365	22.721	2	2015 07 30	13:00
253.151	5.22523	135.1255	0.0007	0.000055	0.0153	0.0031	0.000192	0.0169	0.00520	0.0167	0.0370	22.730	2	2015 07 30	13:14
253.151	5.24607	135.7142	0.0004	0.000020	0.0134	0.0030	0.000186	0.0151	0.00520	0.0168	0.0350	25.094	2	2015 07 30	14:04
253.150	5.24610	135.7068	0.0001	0.000036	0.0137	0.0030	0.000188	0.0154	0.00520	0.0168	0.0353	24.538	2	2015 07 30	14:17
253.152	5.24622	135.7049	0.0005	0.000031	0.0139	0.0030	0.000187	0.0156	0.00520	0.0168	0.0356	24.869	2	2015 07 30	14:30
253.151	5.26682	136.2955	0.0005	0.000016	0.0120	0.0030	0.000186	0.0137	0.00520	0.0169	0.0338	25.730	2	2015 07 30	15:20
253.150	5.26682	136.2794	0.0001	0.000023	0.0139	0.0030	0.000186	0.0155	0.00520	0.0169	0.0354	26.017	2	2015 07 30	15:33
253.151	5.26697	136.2723	0.0005	0.000055	0.0128	0.0030	0.000193	0.0145	0.00520	0.0169	0.0346	25.857	2	2015 07 30	15:46

Table S3. Experimental (p, rho, T, x) data and detailed uncertainty information for isotherms measured on the (0.49896 argon + 0.50104 carbon dioxide) mixture (continued)

test: Ar_CO2_1507p.dat (long evacuation prior to test; 30 min equilibration time; 3 replicates per pressure; replicate of Ar_CO2_1507o)
chiS [specific magnetic susceptibility] = −0.6035E−08 [m3/kg]

T (K)	p (MPa)	rho (kg.m−3)	sigma_T (K)	sigma_p (MPa)	sigma_rho (kg.m−3)	u(T) (K)	u(p) (MPa)	u(rho) (kg.m−3)	u(MW) (g/mol)	u[rho(x)] (kg.m−3)	U_c/% (k = 2)	m_sorb (mg)	p_trans	date	time
253.146	0.49082	9.9905	0.0001	0.000009	0.0010	0.0030	0.000151	0.0020	0.00520	0.0012	0.0788	−0.003	2	2015 07 30	18:15
253.148	0.49084	9.9910	0.0008	0.000011	0.0012	0.0031	0.000151	0.0022	0.00520	0.0012	0.0804	−0.005	2	2015 07 30	18:28
253.151	0.49087	9.9910	0.0008	0.000006	0.0011	0.0031	0.000150	0.0022	0.00520	0.0012	0.0800	−0.005	2	2015 07 30	18:41
253.147	1.05321	21.9388	0.0002	0.000010	0.0004	0.0030	0.000152	0.0018	0.00520	0.0027	0.0424	−0.014	2	2015 07 30	19:42
253.149	1.05319	21.9352	0.0009	0.000003	0.0004	0.0031	0.000152	0.0018	0.00520	0.0027	0.0423	−0.010	2	2015 07 30	19:55
253.152	1.05319	21.9369	0.0008	0.000007	0.0006	0.0031	0.000152	0.0019	0.00520	0.0027	0.0427	−0.007	2	2015 07 30	20:08
253.147	2.08555	45.5017	0.0005	0.000014	0.0005	0.0030	0.000156	0.0025	0.00520	0.0056	0.0319	−0.011	2	2015 07 30	21:19
253.150	2.08553	45.4993	0.0011	0.000009	0.0003	0.0032	0.000156	0.0025	0.00520	0.0056	0.0318	−0.006	2	2015 07 30	21:32
253.147	3.02755	69.2212	0.0003	0.000027	0.0008	0.0030	0.000164	0.0034	0.00520	0.0086	0.0296	−0.009	2	2015 07 30	22:43
253.150	3.02755	69.2279	0.0010	0.000011	0.0004	0.0032	0.000162	0.0032	0.00520	0.0086	0.0295	−0.000	2	2015 07 30	22:56
253.153	3.02756	69.2236	0.0005	0.000014	0.0003	0.0030	0.000163	0.0032	0.00520	0.0086	0.0294	0.005	2	2015 07 30	23:11
253.146	3.99215	96.2998	0.0003	0.000014	0.0011	0.0030	0.000171	0.0043	0.00520	0.0119	0.0286	−0.004	2	2015 07 31	00:25
253.149	3.99216	96.3011	0.0011	0.000017	0.0004	0.0032	0.000171	0.0041	0.00520	0.0119	0.0286	0.005	2	2015 07 31	00:38
253.154	3.99219	96.3002	0.0002	0.000027	0.0011	0.0030	0.000172	0.0043	0.00520	0.0119	0.0287	0.016	2	2015 07 31	01:02
253.148	4.51098	112.3077	0.0001	0.000018	0.0005	0.0030	0.000177	0.0046	0.00520	0.0139	0.0283	−0.006	2	2015 07 31	02:10
253.150	4.51098	112.3059	0.0008	0.000018	0.0003	0.0031	0.000177	0.0046	0.00520	0.0139	0.0283	0.011	2	2015 07 31	02:23
253.152	4.51102	112.3048	0.0006	0.000018	0.0004	0.0031	0.000177	0.0046	0.00520	0.0139	0.0283	0.007	2	2015 07 31	02:36
253.148	4.91290	125.5369	0.0003	0.000017	0.0015	0.0030	0.000181	0.0053	0.00520	0.0155	0.0283	0.011	2	2015 07 31	03:45
253.150	4.91294	125.5357	0.0009	0.000034	0.0005	0.0031	0.000184	0.0050	0.00520	0.0155	0.0283	0.015	2	2015 07 31	03:58
253.153	4.91300	125.5347	0.0007	0.000019	0.0003	0.0031	0.000181	0.0050	0.00520	0.0155	0.0282	0.026	2	2015 07 31	04:11
253.148	4.94213	126.5303	0.0002	0.000019	0.0003	0.0030	0.000182	0.0051	0.00520	0.0157	0.0282	0.019	2	2015 07 31	05:01
253.150	4.94216	126.5300	0.0007	0.000021	0.0007	0.0031	0.000182	0.0051	0.00520	0.0157	0.0283	0.020	2	2015 07 31	05:14
253.152	4.94223	126.5298	0.0006	0.000011	0.0006	0.0031	0.000181	0.0051	0.00520	0.0157	0.0282	0.023	2	2015 07 31	05:27
253.148	4.97079	127.5083	0.0002	0.000019	0.0007	0.0030	0.000182	0.0051	0.00520	0.0158	0.0282	0.014	2	2015 07 31	06:18
253.149	4.97081	127.5069	0.0006	0.000021	0.0004	0.0031	0.000182	0.0051	0.00520	0.0158	0.0282	0.023	2	2015 07 31	06:31
253.152	4.97087	127.5070	0.0007	0.000029	0.0009	0.0031	0.000183	0.0052	0.00520	0.0158	0.0283	0.027	2	2015 07 31	06:44
253.149	4.99888	128.4707	0.0001	0.000029	0.0007	0.0030	0.000184	0.0052	0.00520	0.0159	0.0282	0.029	2	2015 07 31	07:35
253.150	4.99891	128.4673	0.0005	0.000021	0.0004	0.0030	0.000183	0.0051	0.00520	0.0159	0.0282	0.017	2	2015 07 31	07:48
253.152	4.99896	128.4693	0.0005	0.000024	0.0004	0.0030	0.000183	0.0051	0.00520	0.0159	0.0282	0.015	2	2015 07 31	08:01
253.149	5.02645	129.4176	0.0001	0.000074	0.0028	0.0030	0.000196	0.0060	0.00520	0.0160	0.0288	0.032	2	2015 07 31	08:52
253.150	5.02654	129.4190	0.0004	0.000029	0.0002	0.0030	0.000184	0.0051	0.00520	0.0160	0.0282	0.026	2	2015 07 31	09:05
253.152	5.02655	129.4184	0.0006	0.000017	0.0002	0.0031	0.000183	0.0051	0.00520	0.0160	0.0282	0.030	2	2015 07 31	09:18
253.149	5.05327	130.3437	0.0002	0.000014	0.0001	0.0030	0.000183	0.0052	0.00520	0.0161	0.0282	0.042	2	2015 07 31	10:09
253.149	5.05326	130.3425	0.0004	0.000020	0.0005	0.0030	0.000183	0.0052	0.00520	0.0161	0.0282	0.044	2	2015 07 31	10:22
253.151	5.05331	130.3425	0.0006	0.000017	0.0003	0.0031	0.000183	0.0052	0.00520	0.0161	0.0282	0.046	2	2015 07 31	10:35
253.149	5.07913	131.2261	0.0004	0.000018	0.0019	0.0030	0.000183	0.0056	0.00520	0.0163	0.0284	0.242	2	2015 07 31	11:26
253.149	5.07914	131.2242	0.0004	0.000024	0.0007	0.0030	0.000184	0.0052	0.00520	0.0163	0.0282	0.249	2	2015 07 31	11:39
253.151	5.07919	131.2231	0.0007	0.000018	0.0002	0.0031	0.000183	0.0052	0.00520	0.0163	0.0282	0.246	2	2015 07 31	11:52
253.149	5.10258	131.9192	0.0003	0.000029	0.0110	0.0030	0.000185	0.0128	0.00520	0.0163	0.0333	1.547	2	2015 07 31	12:43
253.149	5.10256	131.9096	0.0004	0.000025	0.0048	0.0030	0.000184	0.0074	0.00520	0.0163	0.0293	1.635	2	2015 07 31	12:56
253.151	5.10266	131.9136	0.0005	0.000016	0.0016	0.0030	0.000184	0.0055	0.00520	0.0163	0.0283	1.677	2	2015 07 31	13:09
253.149	5.12315	132.4583	0.0003	0.000022	0.0182	0.0030	0.000184	0.0197	0.00520	0.0164	0.0402	2.461	2	2015 07 31	13:59
253.149	5.12324	132.4444	0.0004	0.000035	0.0152	0.0030	0.000186	0.0168	0.00520	0.0164	0.0371	2.554	2	2015 07 31	14:12
253.151	5.12334	132.4171	0.0005	0.000040	0.0111	0.0030	0.000187	0.0129	0.00520	0.0164	0.0334	2.217	2	2015 07 31	14:25
253.150	5.14333	132.9863	0.0004	0.000021	0.0159	0.0030	0.000185	0.0175	0.00520	0.0165	0.0377	3.338	2	2015 07 31	15:15
253.150	5.14331	132.9688	0.0001	0.000023	0.0160	0.0030	0.000185	0.0176	0.00520	0.0165	0.0378	3.405	2	2015 07 31	15:28
253.150	5.14339	132.9621	0.0004	0.000034	0.0133	0.0030	0.000186	0.0150	0.00520	0.0165	0.0353	3.462	2	2015 07 31	15:41
253.150	5.16310	133.5087	0.0006	0.000025	0.0084	0.0031	0.000185	0.0104	0.00520	0.0165	0.0313	7.583	2	2015 07 31	16:32
253.149	5.16312	133.4987	0.0001	0.000040	0.0141	0.0030	0.000188	0.0157	0.00520	0.0165	0.0359	7.614	2	2015 07 31	16:45

T	p	rho	sigma_T	sigma_p	sigma_rho	u(T)	u(p)	u(rho)	u(MW)	u[rho(x)]	U_c/%	m_sorb	p_trans	date	time
(K)	(MPa)	(kg.m−3)	(K)	(MPa)	(kg.m−3)	(K)	(MPa)	(kg.m−3)	(g/mol)	(kg.m−3)	(k = 2)	(mg)			

test: Ar_CO2_1507p.dat (continued)

253.150	5.16328	133.4888	0.0007	0.000050	0.0158	0.0031	0.000190	0.0174	0.00520	0.0165	0.0377	13.511	2	2015 07 31	16:58
253.149	5.18270	134.0312	0.0006	0.000037	0.0119	0.0031	0.000188	0.0137	0.00520	0.0166	0.0339	18.618	2	2015 07 31	17:49
253.149	5.18272	134.0368	0.0003	0.000032	0.0159	0.0030	0.000187	0.0174	0.00520	0.0166	0.0376	19.469	2	2015 07 31	18:02
253.150	5.18277	134.0237	0.0007	0.000042	0.0160	0.0031	0.000189	0.0176	0.00520	0.0166	0.0378	18.953	2	2015 07 31	18:15
253.149	5.20174	134.5568	0.0007	0.000039	0.0124	0.0031	0.000188	0.0141	0.00520	0.0167	0.0343	21.797	2	2015 07 31	19:05
253.148	5.20175	134.5461	0.0002	0.000034	0.0141	0.0030	0.000187	0.0158	0.00520	0.0167	0.0358	21.418	2	2015 07 31	19:18
253.149	5.20189	134.5301	0.0006	0.000047	0.0148	0.0031	0.000190	0.0164	0.00520	0.0167	0.0365	22.014	2	2015 07 31	19:31
253.149	5.22068	135.0782	0.0007	0.000031	0.0101	0.0031	0.000187	0.0120	0.00520	0.0167	0.0324	23.659	2	2015 07 31	20:21
253.148	5.22067	135.0571	0.0001	0.000029	0.0130	0.0030	0.000187	0.0147	0.00520	0.0167	0.0347	24.211	2	2015 07 31	20:34
253.149	5.22080	135.0418	0.0006	0.000061	0.0143	0.0031	0.000194	0.0160	0.00520	0.0167	0.0361	23.459	2	2015 07 31	20:47
253.149	5.23933	135.5840	0.0005	0.000019	0.0112	0.0030	0.000186	0.0130	0.00520	0.0168	0.0332	25.873	2	2015 07 31	21:38
253.148	5.23935	135.5730	0.0002	0.000039	0.0133	0.0030	0.000189	0.0150	0.00520	0.0168	0.0350	24.627	2	2015 07 31	21:51
253.150	5.23947	135.5504	0.0005	0.000038	0.0140	0.0030	0.000189	0.0156	0.00520	0.0168	0.0356	26.370	2	2015 07 31	22:04
253.149	5.25771	136.0710	0.0004	0.000027	0.0110	0.0030	0.000187	0.0129	0.00520	0.0169	0.0331	24.844	2	2015 07 31	22:55
253.148	5.25776	136.0736	0.0003	0.000046	0.0128	0.0030	0.000190	0.0145	0.00520	0.0169	0.0345	25.875	2	2015 07 31	23:08
253.150	5.25789	136.0764	0.0006	0.000033	0.0141	0.0031	0.000188	0.0157	0.00520	0.0169	0.0356	25.829	2	2015 07 31	23:21
253.149	5.27588	136.5864	0.0004	0.000018	0.0112	0.0030	0.000186	0.0130	0.00520	0.0169	0.0331	26.436	2	2015 08 01	00:11
253.148	5.27592	136.5897	0.0002	0.000028	0.0132	0.0030	0.000187	0.0149	0.00520	0.0169	0.0348	26.969	2	2015 08 01	00:24
253.150	5.27603	136.5762	0.0005	0.000031	0.0129	0.0030	0.000188	0.0146	0.00520	0.0169	0.0346	27.271	2	2015 08 01	00:37
253.148	5.29382	137.0750	0.0004	0.000009	0.0100	0.0030	0.000186	0.0120	0.00520	0.0170	0.0322	27.272	2	2015 08 01	01:27
253.148	5.29384	137.0831	0.0003	0.000023	0.0131	0.0030	0.000187	0.0148	0.00520	0.0170	0.0347	27.606	2	2015 08 01	01:40
253.150	5.29399	137.0828	0.0007	0.000061	0.0133	0.0031	0.000195	0.0150	0.00520	0.0170	0.0349	27.794	2	2015 08 01	01:53
253.148	5.31144	137.5655	0.0002	0.000028	0.0103	0.0030	0.000188	0.0122	0.00520	0.0170	0.0324	28.541	2	2015 08 01	02:49
253.148	5.31153	137.5607	0.0004	0.000054	0.0136	0.0030	0.000193	0.0153	0.00520	0.0170	0.0351	26.861	2	2015 08 01	03:02
253.150	5.31169	137.5408	0.0008	0.000057	0.0125	0.0031	0.000194	0.0143	0.00520	0.0170	0.0343	29.373	2	2015 08 01	03:15
253.148	5.32889	138.0516	0.0005	0.000021	0.0101	0.0030	0.000187	0.0120	0.00520	0.0171	0.0322	29.525	2	2015 08 01	04:05
253.148	5.32893	138.0652	0.0004	0.000046	0.0136	0.0030	0.000191	0.0153	0.00520	0.0171	0.0351	30.367	2	2015 08 01	04:18
253.150	5.32910	138.0409	0.0007	0.000055	0.0135	0.0031	0.000194	0.0152	0.00520	0.0171	0.0350	29.851	2	2015 08 01	04:31
253.149	5.34610	138.5273	0.0005	0.000028	0.0108	0.0030	0.000188	0.0126	0.00520	0.0172	0.0327	30.745	2	2015 08 01	05:21
253.148	5.34613	138.5249	0.0002	0.000032	0.0123	0.0030	0.000189	0.0141	0.00520	0.0172	0.0339	29.470	2	2015 08 01	05:34
253.150	5.34627	138.5068	0.0007	0.000040	0.0123	0.0031	0.000190	0.0141	0.00520	0.0172	0.0339	30.060	2	2015 08 01	05:47
253.148	5.36303	138.9923	0.0003	0.000018	0.0100	0.0030	0.000187	0.0119	0.00520	0.0172	0.0321	30.458	2	2015 08 01	06:38
253.148	5.36308	139.0021	0.0004	0.000047	0.0123	0.0030	0.000192	0.0141	0.00520	0.0172	0.0339	31.035	2	2015 08 01	06:51
253.150	5.36325	139.0028	0.0006	0.000049	0.0122	0.0031	0.000192	0.0140	0.00520	0.0172	0.0338	29.529	2	2015 08 01	07:04
253.148	5.37978	139.4752	0.0004	0.000019	0.0093	0.0030	0.000187	0.0113	0.00520	0.0173	0.0316	30.807	2	2015 08 01	07:55
253.148	5.37982	139.4696	0.0003	0.000041	0.0122	0.0030	0.000191	0.0140	0.00520	0.0173	0.0338	31.438	2	2015 08 01	08:08
253.150	5.37998	139.4584	0.0007	0.000045	0.0121	0.0031	0.000192	0.0139	0.00520	0.0173	0.0337	30.201	2	2015 08 01	08:21

Table S3. Experimental (p, rho, T, x) data and detailed uncertainty information for isotherms measured on the (0.49896 argon + 0.50104 carbon dioxide) mixture (continued)

test: Ar_CO2_1508a.dat (brief purge prior to test; 60 min equilibration time; 6 replicates per pressure)
chiS [specific magnetic susceptibility] = −0.6035E−08 [m3/kg]

T (K)	p (MPa)	rho (kg.m−3)	sigma_T (K)	sigma_p (MPa)	sigma_rho (kg.m−3)	u(T) (K)	u(p) (MPa)	u(rho) (kg.m−3)	u(MW) (g/mol)	u[rho(x)] (kg.m−3)	U_c/% (k = 2)	m_sorb (mg)	p_trans	date	time
253.151	0.49841	10.1472	0.0005	0.000009	0.0012	0.0030	0.000151	0.0023	0.00520	0.0013	0.0799	−0.007	2	2015 08 01	13:12
253.152	0.49842	10.1485	0.0003	0.000007	0.0011	0.0030	0.000150	0.0021	0.00520	0.0013	0.0783	−0.006	2	2015 08 01	13:25
253.150	0.49843	10.1490	0.0006	0.000005	0.0010	0.0031	0.000150	0.0020	0.00520	0.0013	0.0776	−0.007	2	2015 08 01	13:38
253.148	0.49844	10.1534	0.0003	0.000004	0.0009	0.0030	0.000150	0.0019	0.00520	0.0013	0.0765	−0.007	2	2015 08 01	13:51
253.149	0.49845	10.1538	0.0005	0.000008	0.0009	0.0030	0.000151	0.0020	0.00520	0.0013	0.0767	−0.007	2	2015 08 01	14:04
253.151	0.49846	10.1504	0.0006	0.000009	0.0011	0.0031	0.000151	0.0021	0.00520	0.0013	0.0787	−0.006	2	2015 08 01	14:17
253.151	1.03898	21.6313	0.0007	0.000005	0.0008	0.0031	0.000152	0.0021	0.00520	0.0027	0.0438	−0.007	2	2015 08 01	15:41
253.153	1.03899	21.6333	0.0002	0.000004	0.0006	0.0030	0.000152	0.0019	0.00520	0.0027	0.0430	−0.006	2	2015 08 01	15:54
253.152	1.03897	21.6340	0.0006	0.000009	0.0004	0.0031	0.000152	0.0018	0.00520	0.0027	0.0427	−0.006	2	2015 08 01	16:07
253.149	1.03897	21.6316	0.0006	0.000010	0.0006	0.0031	0.000152	0.0019	0.00520	0.0027	0.0432	−0.005	2	2015 08 01	16:20
253.148	1.03897	21.6315	0.0001	0.000002	0.0004	0.0030	0.000151	0.0018	0.00520	0.0027	0.0426	−0.006	2	2015 08 01	16:33
253.150	1.03897	21.6313	0.0005	0.000006	0.0005	0.0030	0.000152	0.0019	0.00520	0.0027	0.0430	−0.005	2	2015 08 01	16:46
253.152	2.03469	44.2855	0.0007	0.000009	0.0005	0.0031	0.000156	0.0025	0.00520	0.0055	0.0321	−0.003	2	2015 08 01	18:23
253.153	2.03469	44.2859	0.0002	0.000009	0.0006	0.0030	0.000156	0.0025	0.00520	0.0055	0.0321	−0.004	2	2015 08 01	18:36
253.151	2.03467	44.2857	0.0007	0.000014	0.0006	0.0031	0.000156	0.0025	0.00520	0.0055	0.0322	0.001	2	2015 08 01	18:49
253.149	2.03465	44.2880	0.0006	0.000011	0.0004	0.0031	0.000156	0.0025	0.00520	0.0055	0.0320	−0.002	2	2015 08 01	19:02
253.149	2.03465	44.2887	0.0003	0.000010	0.0009	0.0030	0.000156	0.0027	0.00520	0.0055	0.0324	−0.003	2	2015 08 01	19:15
253.150	2.03465	44.2826	0.0006	0.000012	0.0005	0.0031	0.000156	0.0025	0.00520	0.0055	0.0321	0.002	2	2015 08 01	19:28
253.154	3.01603	68.9184	0.0004	0.000009	0.0006	0.0030	0.000162	0.0033	0.00520	0.0085	0.0295	0.003	2	2015 08 01	21:10
253.153	3.01601	68.9180	0.0005	0.000017	0.0002	0.0030	0.000163	0.0032	0.00520	0.0085	0.0295	0.004	2	2015 08 01	21:23
253.150	3.01597	68.9199	0.0008	0.000010	0.0010	0.0031	0.000162	0.0034	0.00520	0.0085	0.0296	0.008	2	2015 08 01	21:36
253.148	3.01594	68.9200	0.0003	0.000012	0.0009	0.0030	0.000162	0.0034	0.00520	0.0085	0.0296	0.002	2	2015 08 01	21:49
253.149	3.01596	68.9190	0.0004	0.000010	0.0001	0.0030	0.000162	0.0032	0.00520	0.0085	0.0294	0.002	2	2015 08 01	22:02
253.151	3.01599	68.9211	0.0007	0.000017	0.0003	0.0031	0.000163	0.0032	0.00520	0.0085	0.0295	−0.000	2	2015 08 01	22:15
253.153	4.01921	97.1052	0.0007	0.000012	0.0003	0.0031	0.000171	0.0041	0.00520	0.0120	0.0285	0.008	2	2015 08 01	23:57
253.153	4.01919	97.1061	0.0003	0.000032	0.0007	0.0030	0.000174	0.0042	0.00520	0.0120	0.0286	0.015	2	2015 08 02	00:10
253.151	4.01913	97.1059	0.0008	0.000029	0.0008	0.0031	0.000173	0.0042	0.00520	0.0120	0.0286	0.003	2	2015 08 02	00:23
253.148	4.01908	97.1040	0.0006	0.000010	0.0005	0.0031	0.000171	0.0041	0.00520	0.0120	0.0285	0.007	2	2015 08 02	00:36
253.148	4.01908	97.1031	0.0003	0.000021	0.0005	0.0030	0.000172	0.0041	0.00520	0.0120	0.0285	0.007	2	2015 08 02	00:49
253.150	4.01912	97.1057	0.0008	0.000015	0.0005	0.0031	0.000171	0.0041	0.00520	0.0120	0.0285	0.011	2	2015 08 02	01:02
253.153	4.52392	112.7140	0.0002	0.000013	0.0005	0.0030	0.000176	0.0046	0.00520	0.0140	0.0283	0.007	2	2015 08 02	02:44
253.152	4.52388	112.7154	0.0004	0.000015	0.0008	0.0030	0.000177	0.0047	0.00520	0.0140	0.0283	0.014	2	2015 08 02	02:57
253.150	4.52383	112.7161	0.0006	0.000015	0.0002	0.0031	0.000177	0.0046	0.00520	0.0140	0.0283	0.007	2	2015 08 02	03:10
253.149	4.52382	112.7148	0.0002	0.000029	0.0010	0.0030	0.000178	0.0047	0.00520	0.0140	0.0284	0.001	2	2015 08 02	03:23
253.150	4.52386	112.7171	0.0005	0.000013	0.0003	0.0030	0.000176	0.0046	0.00520	0.0140	0.0283	0.010	2	2015 08 02	03:36
253.151	4.52388	112.7163	0.0004	0.000018	0.0004	0.0030	0.000177	0.0046	0.00520	0.0140	0.0283	0.004	2	2015 08 02	03:49
253.152	4.90919	125.4058	0.0004	0.000016	0.0010	0.0030	0.000181	0.0051	0.00520	0.0155	0.0283	0.022	2	2015 08 02	05:27
253.153	4.90918	125.4062	0.0004	0.000021	0.0008	0.0030	0.000182	0.0051	0.00520	0.0155	0.0283	0.007	2	2015 08 02	05:40
253.151	4.90912	125.4067	0.0007	0.000032	0.0002	0.0031	0.000183	0.0050	0.00520	0.0155	0.0283	0.033	2	2015 08 02	05:53
253.149	4.90907	125.4044	0.0003	0.000018	0.0009	0.0030	0.000181	0.0051	0.00520	0.0155	0.0283	0.009	2	2015 08 02	06:06
253.149	4.90910	125.4082	0.0003	0.000023	0.0006	0.0030	0.000182	0.0050	0.00520	0.0155	0.0282	0.026	2	2015 08 02	06:19
253.151	4.90914	125.4052	0.0005	0.000023	0.0004	0.0030	0.000182	0.0050	0.00520	0.0155	0.0282	0.016	2	2015 08 02	06:32
253.151	4.93490	126.2807	0.0006	0.000030	0.0007	0.0031	0.000183	0.0051	0.00520	0.0156	0.0283	0.028	2	2015 08 02	07:53
253.152	4.93493	126.2789	0.0004	0.000011	0.0005	0.0030	0.000181	0.0051	0.00520	0.0156	0.0282	0.019	2	2015 08 02	08:06
253.152	4.93491	126.2807	0.0005	0.000025	0.0004	0.0030	0.000182	0.0051	0.00520	0.0156	0.0282	0.020	2	2015 08 02	08:19
253.150	4.93487	126.2803	0.0006	0.000022	0.0005	0.0031	0.000182	0.0051	0.00520	0.0156	0.0282	0.011	2	2015 08 02	08:32
253.148	4.93485	126.2801	0.0002	0.000019	0.0004	0.0030	0.000182	0.0050	0.00520	0.0156	0.0282	0.003	2	2015 08 02	08:45
253.149	4.93486	126.2794	0.0005	0.000031	0.0004	0.0030	0.000183	0.0051	0.00520	0.0156	0.0283	0.015	2	2015 08 02	08:58

T	p	rho	sigma_T	sigma_p	sigma_rho	u(T)	u(p)	u(rho)	u(MW)	u[rho(x)]	U_c/%	m_sorb	p_trans	date	time
(K)	(MPa)	(kg.m-3)	(K)	(MPa)	(kg.m-3)	(K)	(MPa)	(kg.m-3)	(g/mol)	(kg.m-3)	(k = 2)	(mg)			
# test: Ar_CO2_1508a.dat (continued)															
253.150	4.96000	127.1379	0.0007	0.000026	0.0006	0.0031	0.000183	0.0051	0.00520	0.0157	0.0283	0.023	2	2015 08 02	10:19
253.152	4.96005	127.1356	0.0003	0.000009	0.0003	0.0030	0.000181	0.0051	0.00520	0.0157	0.0282	0.014	2	2015 08 02	10:32
253.152	4.96005	127.1359	0.0003	0.000019	0.0005	0.0030	0.000182	0.0051	0.00520	0.0157	0.0282	0.017	2	2015 08 02	10:45
253.151	4.96001	127.1354	0.0006	0.000031	0.0006	0.0031	0.000184	0.0051	0.00520	0.0157	0.0283	0.019	2	2015 08 02	10:58
253.149	4.95995	127.1336	0.0004	0.000018	0.0010	0.0030	0.000182	0.0052	0.00520	0.0157	0.0283	0.017	2	2015 08 02	11:11
253.149	4.95996	127.1360	0.0003	0.000022	0.0005	0.0030	0.000182	0.0051	0.00520	0.0157	0.0282	0.014	2	2015 08 02	11:24
253.149	4.98457	127.9762	0.0003	0.000019	0.0003	0.0030	0.000182	0.0051	0.00520	0.0159	0.0282	0.016	2	2015 08 02	12:45
253.151	4.98461	127.9758	0.0004	0.000021	0.0001	0.0030	0.000183	0.0051	0.00520	0.0159	0.0282	0.018	2	2015 08 02	12:58
253.152	4.98460	127.9776	0.0001	0.000023	0.0006	0.0030	0.000183	0.0051	0.00520	0.0159	0.0282	0.029	2	2015 08 02	13:11
253.151	4.98461	127.9771	0.0004	0.000018	0.0001	0.0030	0.000182	0.0051	0.00520	0.0159	0.0282	0.023	2	2015 08 02	13:24
253.150	4.98455	127.9797	0.0004	0.000026	0.0011	0.0030	0.000183	0.0052	0.00520	0.0159	0.0283	0.029	2	2015 08 02	13:37
253.149	4.98454	127.9774	0.0001	0.000021	0.0011	0.0030	0.000182	0.0052	0.00520	0.0159	0.0283	0.026	2	2015 08 02	13:50
253.149	5.00869	128.8069	0.0001	0.000026	0.0010	0.0030	0.000183	0.0052	0.00520	0.0160	0.0283	0.028	2	2015 08 02	15:10
253.149	5.00874	128.8060	0.0004	0.000009	0.0002	0.0030	0.000182	0.0051	0.00520	0.0160	0.0282	0.024	2	2015 08 02	15:23
253.151	5.00875	128.8052	0.0003	0.000011	0.0001	0.0030	0.000182	0.0051	0.00520	0.0160	0.0282	0.020	2	2015 08 02	15:36
253.151	5.00878	128.8055	0.0001	0.000017	0.0005	0.0030	0.000182	0.0051	0.00520	0.0160	0.0282	0.019	2	2015 08 02	15:49
253.151	5.00874	128.8071	0.0003	0.000020	0.0003	0.0030	0.000183	0.0051	0.00520	0.0160	0.0282	0.028	2	2015 08 02	16:02
253.149	5.00872	128.8052	0.0003	0.000016	0.0004	0.0030	0.000182	0.0051	0.00520	0.0160	0.0282	0.031	2	2015 08 02	16:15
253.149	5.03228	129.6187	0.0001	0.000024	0.0006	0.0030	0.000183	0.0052	0.00520	0.0161	0.0282	0.038	2	2015 08 02	17:36
253.149	5.03231	129.6171	0.0004	0.000015	0.0003	0.0030	0.000183	0.0051	0.00520	0.0161	0.0282	0.036	2	2015 08 02	17:49
253.151	5.03234	129.6175	0.0005	0.000020	0.0004	0.0030	0.000183	0.0052	0.00520	0.0161	0.0282	0.034	2	2015 08 02	18:02
253.152	5.03237	129.6180	0.0001	0.000012	0.0006	0.0030	0.000182	0.0052	0.00520	0.0161	0.0282	0.033	2	2015 08 02	18:15
253.151	5.03235	129.6189	0.0004	0.000026	0.0003	0.0030	0.000184	0.0051	0.00520	0.0161	0.0282	0.040	2	2015 08 02	18:28
253.150	5.03229	129.6175	0.0004	0.000022	0.0004	0.0030	0.000183	0.0052	0.00520	0.0161	0.0282	0.034	2	2015 08 02	18:41
253.149	5.05544	130.4183	0.0002	0.000018	0.0003	0.0030	0.000183	0.0052	0.00520	0.0162	0.0282	0.055	2	2015 08 02	20:02
253.149	5.05547	130.4167	0.0002	0.000017	0.0004	0.0030	0.000183	0.0052	0.00520	0.0162	0.0282	0.050	2	2015 08 02	20:15
253.151	5.05553	130.4173	0.0004	0.000029	0.0003	0.0030	0.000184	0.0052	0.00520	0.0162	0.0282	0.057	2	2015 08 02	20:28
253.152	5.05554	130.4178	0.0002	0.000018	0.0002	0.0030	0.000183	0.0052	0.00520	0.0162	0.0282	0.046	2	2015 08 02	20:41
253.151	5.05551	130.4188	0.0004	0.000028	0.0006	0.0030	0.000184	0.0052	0.00520	0.0162	0.0282	0.052	2	2015 08 02	20:54
253.149	5.05546	130.4172	0.0005	0.000015	0.0007	0.0030	0.000183	0.0052	0.00520	0.0162	0.0282	0.046	2	2015 08 02	21:07
253.148	5.07773	131.1782	0.0002	0.000021	0.0010	0.0030	0.000184	0.0053	0.00520	0.0162	0.0282	0.208	2	2015 08 02	22:27
253.149	5.07775	131.1772	0.0004	0.000024	0.0005	0.0030	0.000184	0.0052	0.00520	0.0162	0.0282	0.204	2	2015 08 02	22:40
253.151	5.07779	131.1790	0.0005	0.000034	0.0012	0.0030	0.000186	0.0054	0.00520	0.0162	0.0283	0.217	2	2015 08 02	22:53
253.152	5.07784	131.1777	0.0003	0.000028	0.0008	0.0030	0.000185	0.0053	0.00520	0.0162	0.0283	0.201	2	2015 08 02	23:06
253.151	5.07782	131.1770	0.0004	0.000020	0.0010	0.0030	0.000184	0.0053	0.00520	0.0162	0.0283	0.201	2	2015 08 02	23:19
253.149	5.07776	131.1786	0.0004	0.000033	0.0008	0.0030	0.000186	0.0053	0.00520	0.0162	0.0283	0.199	2	2015 08 02	23:32
253.149	5.09827	131.8062	0.0001	0.000019	0.0038	0.0030	0.000184	0.0066	0.00520	0.0163	0.0289	1.190	2	2015 08 03	00:53
253.149	5.09829	131.8077	0.0004	0.000028	0.0008	0.0030	0.000185	0.0053	0.00520	0.0163	0.0283	1.189	2	2015 08 03	01:06
253.151	5.09837	131.8097	0.0005	0.000026	0.0024	0.0030	0.000185	0.0059	0.00520	0.0163	0.0285	1.190	2	2015 08 03	01:19
253.152	5.09839	131.8100	0.0002	0.000023	0.0009	0.0030	0.000184	0.0053	0.00520	0.0163	0.0282	1.159	2	2015 08 03	01:32
253.151	5.09835	131.8111	0.0004	0.000031	0.0004	0.0030	0.000185	0.0052	0.00520	0.0163	0.0282	1.141	2	2015 08 03	01:45
253.149	5.09825	131.8088	0.0006	0.000026	0.0017	0.0031	0.000185	0.0055	0.00520	0.0163	0.0284	1.125	2	2015 08 03	01:58
253.149	5.11645	132.2623	0.0004	0.000017	0.0100	0.0030	0.000184	0.0119	0.00520	0.0164	0.0325	2.596	2	2015 08 03	03:19
253.149	5.11647	132.2571	0.0002	0.000022	0.0091	0.0030	0.000184	0.0111	0.00520	0.0164	0.0318	2.701	2	2015 08 03	03:32
253.151	5.11655	132.2620	0.0005	0.000023	0.0061	0.0030	0.000184	0.0084	0.00520	0.0164	0.0299	2.794	2	2015 08 03	03:45
253.152	5.11659	132.2458	0.0003	0.000024	0.0020	0.0030	0.000185	0.0057	0.00520	0.0164	0.0284	2.868	2	2015 08 03	03:58
253.152	5.11653	132.2469	0.0002	0.000032	0.0018	0.0030	0.000186	0.0056	0.00520	0.0164	0.0284	2.916	2	2015 08 03	04:11
253.150	5.11642	132.2441	0.0005	0.000044	0.0016	0.0030	0.000188	0.0055	0.00520	0.0164	0.0284	2.939	2	2015 08 03	04:24

test: Ar_CO2_1508a.dat (continued)

T (K)	p (MPa)	rho (kg.m−3)	sigma_T (K)	sigma_p (MPa)	sigma_rho (kg.m−3)	u(T) (K)	u(p) (MPa)	u(rho) (kg.m−3)	u(MW) (g/mol)	u[rho(x)] (kg.m−3)	U_c/% (k = 2)	m_sorb (mg)	p_trans	date	time
253.149	5.13377	132.7076	0.0002	0.000018	0.0096	0.0030	0.000184	0.0115	0.00520	0.0164	0.0322	3.623	2	2015 08 03	05:44
253.149	5.13380	132.6988	0.0003	0.000026	0.0103	0.0030	0.000185	0.0122	0.00520	0.0164	0.0327	3.681	2	2015 08 03	05:57
253.150	5.13389	132.6986	0.0003	0.000025	0.0080	0.0030	0.000185	0.0100	0.00520	0.0164	0.0310	3.737	2	2015 08 03	06:10
253.151	5.13389	132.6847	0.0001	0.000019	0.0054	0.0030	0.000184	0.0078	0.00520	0.0164	0.0295	3.794	2	2015 08 03	06:23
253.151	5.13381	132.7176	0.0003	0.000042	0.0036	0.0030	0.000188	0.0066	0.00520	0.0164	0.0289	3.837	2	2015 08 03	06:36
253.149	5.13374	132.6848	0.0003	0.000023	0.0035	0.0030	0.000185	0.0065	0.00520	0.0164	0.0288	7.180	2	2015 08 03	06:49
253.149	5.15072	133.1400	0.0001	0.000045	0.0108	0.0030	0.000189	0.0126	0.00520	0.0165	0.0331	14.745	2	2015 08 03	08:10
253.150	5.15083	133.1412	0.0005	0.000024	0.0114	0.0030	0.000185	0.0132	0.00520	0.0165	0.0336	14.393	2	2015 08 03	08:23
253.151	5.15090	133.1298	0.0003	0.000014	0.0079	0.0030	0.000184	0.0100	0.00520	0.0165	0.0309	15.225	2	2015 08 03	08:36
253.152	5.15088	133.1141	0.0001	0.000029	0.0046	0.0030	0.000186	0.0072	0.00520	0.0165	0.0292	15.061	2	2015 08 03	08:49
253.151	5.15079	133.1247	0.0004	0.000026	0.0035	0.0030	0.000185	0.0065	0.00520	0.0165	0.0288	16.074	2	2015 08 03	09:02
253.149	5.15067	133.1199	0.0003	0.000033	0.0039	0.0030	0.000186	0.0068	0.00520	0.0165	0.0290	16.062	2	2015 08 03	09:15
253.149	5.16739	133.5830	0.0001	0.000017	0.0091	0.0030	0.000184	0.0111	0.00520	0.0165	0.0318	18.344	2	2015 08 03	10:37
253.149	5.16747	133.5693	0.0004	0.000052	0.0106	0.0030	0.000191	0.0124	0.00520	0.0165	0.0329	18.400	2	2015 08 03	10:50
253.151	5.16762	133.5570	0.0006	0.000038	0.0096	0.0031	0.000188	0.0115	0.00520	0.0165	0.0321	18.542	2	2015 08 03	11:03
253.152	5.16767	133.5494	0.0002	0.000018	0.0060	0.0030	0.000185	0.0083	0.00520	0.0165	0.0298	18.238	2	2015 08 03	11:16
253.151	5.16758	133.5453	0.0004	0.000032	0.0026	0.0030	0.000186	0.0060	0.00520	0.0165	0.0286	18.892	2	2015 08 03	11:29
253.150	5.16744	133.5664	0.0005	0.000033	0.0030	0.0030	0.000187	0.0062	0.00520	0.0165	0.0287	19.837	2	2015 08 03	11:42
253.149	5.18407	134.0089	0.0004	0.000030	0.0082	0.0030	0.000186	0.0102	0.00520	0.0166	0.0311	22.464	2	2015 08 03	13:03
253.149	5.18410	134.0145	0.0003	0.000031	0.0096	0.0030	0.000186	0.0115	0.00520	0.0166	0.0321	21.032	2	2015 08 03	13:16
253.150	5.18421	134.0065	0.0006	0.000040	0.0105	0.0031	0.000188	0.0123	0.00520	0.0166	0.0328	20.811	2	2015 08 03	13:29
253.151	5.18431	134.0016	0.0003	0.000020	0.0076	0.0030	0.000185	0.0097	0.00520	0.0166	0.0307	22.787	2	2015 08 03	13:42
253.152	5.18425	133.9802	0.0002	0.000035	0.0048	0.0030	0.000187	0.0075	0.00520	0.0166	0.0293	21.854	2	2015 08 03	13:55
253.150	5.18414	133.9836	0.0004	0.000030	0.0039	0.0030	0.000186	0.0068	0.00520	0.0166	0.0289	22.738	2	2015 08 03	14:08
253.149	5.20033	134.4501	0.0002	0.000025	0.0087	0.0030	0.000186	0.0107	0.00520	0.0167	0.0314	23.845	2	2015 08 03	15:28
253.149	5.20032	134.4403	0.0002	0.000026	0.0093	0.0030	0.000186	0.0113	0.00520	0.0167	0.0319	24.612	2	2015 08 03	15:41
253.150	5.20039	134.4207	0.0003	0.000023	0.0086	0.0030	0.000186	0.0106	0.00520	0.0166	0.0313	24.534	2	2015 08 03	15:54
253.151	5.20043	134.4057	0.0002	0.000019	0.0080	0.0030	0.000185	0.0100	0.00520	0.0166	0.0309	24.506	2	2015 08 03	16:07
253.151	5.20042	134.4230	0.0001	0.000018	0.0058	0.0030	0.000185	0.0082	0.00520	0.0166	0.0297	23.897	2	2015 08 03	16:20
253.150	5.20040	134.4062	0.0001	0.000018	0.0048	0.0030	0.000185	0.0074	0.00520	0.0166	0.0293	25.724	2	2015 08 03	16:33

Table S3. Experimental (p, rho, T, x) data and detailed uncertainty information for isotherms measured on the (0.49896 argon + 0.50104 carbon dioxide) mixture (continued)

test: Ar_CO2_1508b.dat (overnight evacuation prior to test; 30 min equilibration time; 3 replicates per pressure)
chiS [specific magnetic susceptibility] = −0.6035E−08 [m3/kg]

T (K)	p (MPa)	rho (kg.m−3)	sigma_T (K)	sigma_p (MPa)	sigma_rho (kg.m−3)	u(T) (K)	u(p) (MPa)	u(rho) (kg.m−3)	u(MW) (g/mol)	u[rho(x)] (kg.m−3)	U_c/% (k = 2)	m_sorb (mg)	p_trans	date	time
253.147	0.49654	10.1117	0.0005	0.000006	0.0006	0.0030	0.000150	0.0016	0.00520	0.0013	0.0741	−0.005	2	2015 08 04	10:41
253.150	0.49655	10.1147	0.0009	0.000004	0.0004	0.0031	0.000150	0.0015	0.00520	0.0013	0.0732	−0.006	2	2015 08 04	10:54
253.153	0.49655	10.1096	0.0004	0.000004	0.0005	0.0030	0.000150	0.0016	0.00520	0.0013	0.0735	−0.006	2	2015 08 04	11:07
253.149	1.01861	21.1867	0.0004	0.000011	0.0006	0.0030	0.000152	0.0019	0.00520	0.0026	0.0436	−0.013	2	2015 08 04	12:03
253.149	1.01857	21.1850	0.0006	0.000011	0.0004	0.0031	0.000152	0.0018	0.00520	0.0026	0.0431	−0.009	2	2015 08 04	12:16
253.152	1.01856	21.1862	0.0008	0.000007	0.0003	0.0031	0.000152	0.0017	0.00520	0.0026	0.0431	−0.008	2	2015 08 04	12:29
253.147	2.07267	45.1926	0.0002	0.000022	0.0006	0.0030	0.000157	0.0026	0.00520	0.0056	0.0320	−0.016	2	2015 08 04	13:38
253.149	2.07264	45.1899	0.0010	0.000010	0.0002	0.0032	0.000156	0.0024	0.00520	0.0056	0.0318	−0.005	2	2015 08 04	13:51
253.152	2.07263	45.1945	0.0007	0.000011	0.0005	0.0031	0.000156	0.0025	0.00520	0.0056	0.0319	−0.005	2	2015 08 04	14:04
253.147	2.99804	68.4411	0.0004	0.000026	0.0010	0.0030	0.000164	0.0034	0.00520	0.0085	0.0297	−0.010	2	2015 08 04	15:14
253.149	2.99802	68.4428	0.0011	0.000011	0.0002	0.0032	0.000162	0.0032	0.00520	0.0085	0.0295	−0.003	2	2015 08 04	15:27
253.153	2.99803	68.4411	0.0007	0.000015	0.0004	0.0031	0.000162	0.0032	0.00520	0.0085	0.0295	0.005	2	2015 08 04	15:40
253.147	4.03055	97.4494	0.0003	0.000017	0.0010	0.0030	0.000172	0.0043	0.00520	0.0121	0.0286	0.000	2	2015 08 04	16:58
253.150	4.03057	97.4471	0.0010	0.000017	0.0005	0.0032	0.000172	0.0041	0.00520	0.0121	0.0285	0.006	2	2015 08 04	17:11
253.153	4.03058	97.4466	0.0003	0.000011	0.0005	0.0030	0.000171	0.0041	0.00520	0.0121	0.0285	0.009	2	2015 08 04	17:27
253.148	4.51949	112.5759	0.0001	0.000028	0.0008	0.0030	0.000178	0.0047	0.00520	0.0139	0.0284	0.004	2	2015 08 04	18:33
253.149	4.51948	112.5755	0.0006	0.000017	0.0008	0.0031	0.000177	0.0047	0.00520	0.0139	0.0283	0.005	2	2015 08 04	18:46
253.152	4.51951	112.5767	0.0006	0.000011	0.0003	0.0031	0.000176	0.0046	0.00520	0.0139	0.0283	0.016	2	2015 08 04	18:59
253.148	4.90884	125.3986	0.0002	0.000023	0.0012	0.0030	0.000182	0.0052	0.00520	0.0155	0.0283	0.009	2	2015 08 04	20:07
253.149	4.90883	125.3989	0.0008	0.000040	0.0006	0.0031	0.000185	0.0050	0.00520	0.0155	0.0283	0.019	2	2015 08 04	20:20
253.152	4.90888	125.3978	0.0007	0.000015	0.0004	0.0031	0.000181	0.0050	0.00520	0.0155	0.0282	0.023	2	2015 08 04	20:33
253.148	4.93092	126.1505	0.0001	0.000025	0.0008	0.0030	0.000182	0.0051	0.00520	0.0156	0.0283	0.022	2	2015 08 04	21:23
253.149	4.93093	126.1486	0.0005	0.000020	0.0003	0.0030	0.000182	0.0050	0.00520	0.0156	0.0282	0.017	2	2015 08 04	21:36
253.151	4.93098	126.1485	0.0006	0.000022	0.0006	0.0031	0.000182	0.0051	0.00520	0.0156	0.0283	0.023	2	2015 08 04	21:49
253.148	4.95260	126.8866	0.0002	0.000015	0.0012	0.0030	0.000182	0.0052	0.00520	0.0157	0.0283	0.018	2	2015 08 04	22:40
253.149	4.95263	126.8875	0.0006	0.000023	0.0002	0.0031	0.000182	0.0051	0.00520	0.0157	0.0282	0.016	2	2015 08 04	22:53
253.151	4.95268	126.8876	0.0006	0.000023	0.0009	0.0031	0.000182	0.0052	0.00520	0.0157	0.0283	0.025	2	2015 08 04	23:06
253.148	4.97391	127.6157	0.0002	0.000015	0.0004	0.0030	0.000182	0.0051	0.00520	0.0158	0.0282	0.018	2	2015 08 04	23:57
253.149	4.97395	127.6161	0.0007	0.000025	0.0004	0.0031	0.000183	0.0051	0.00520	0.0158	0.0282	0.021	2	2015 08 05	00:10
253.152	4.97400	127.6136	0.0006	0.000010	0.0001	0.0031	0.000181	0.0051	0.00520	0.0158	0.0282	0.021	2	2015 08 05	00:23
253.148	4.99495	128.3352	0.0002	0.000009	0.0009	0.0030	0.000182	0.0052	0.00520	0.0159	0.0282	0.019	2	2015 08 05	01:14
253.149	4.99496	128.3363	0.0007	0.000020	0.0004	0.0031	0.000183	0.0051	0.00520	0.0159	0.0282	0.017	2	2015 08 05	01:27
253.151	4.99504	128.3358	0.0007	0.000029	0.0005	0.0031	0.000184	0.0051	0.00520	0.0159	0.0283	0.026	2	2015 08 05	01:40
253.148	5.01563	129.0441	0.0004	0.000016	0.0001	0.0030	0.000182	0.0051	0.00520	0.0160	0.0282	0.025	2	2015 08 05	02:31
253.148	5.01562	129.0462	0.0005	0.000025	0.0005	0.0030	0.000183	0.0052	0.00520	0.0160	0.0282	0.031	2	2015 08 05	02:44
253.151	5.01571	129.0466	0.0008	0.000025	0.0008	0.0031	0.000183	0.0052	0.00520	0.0160	0.0283	0.041	2	2015 08 05	02:57
253.148	5.03594	129.7469	0.0003	0.000018	0.0001	0.0030	0.000183	0.0051	0.00520	0.0161	0.0282	0.032	2	2015 08 05	03:47
253.149	5.03597	129.7469	0.0004	0.000023	0.0003	0.0030	0.000183	0.0052	0.00520	0.0161	0.0282	0.043	2	2015 08 05	04:00
253.151	5.03603	129.7465	0.0008	0.000020	0.0001	0.0031	0.000183	0.0051	0.00520	0.0161	0.0282	0.042	2	2015 08 05	04:13
253.148	5.05586	130.4326	0.0002	0.000025	0.0006	0.0030	0.000184	0.0052	0.00520	0.0162	0.0282	0.076	2	2015 08 05	05:03
253.149	5.05588	130.4293	0.0005	0.000013	0.0011	0.0030	0.000183	0.0053	0.00520	0.0162	0.0283	0.068	2	2015 08 05	05:16
253.151	5.05593	130.4329	0.0007	0.000018	0.0004	0.0031	0.000183	0.0052	0.00520	0.0162	0.0282	0.076	2	2015 08 05	05:29

T (K)	p (MPa)	rho (kg.m−3)	sigma_T (K)	sigma_p (MPa)	sigma_rho (kg.m−3)	u(T) (K)	u(p) (MPa)	u(rho) (kg.m−3)	u(MW) (g/mol)	u[rho(x)] (kg.m−3)	U_c/% (k = 2)	m_sorb (mg)	p_trans	date	time
# test: Ar_CO2_1508b.dat (continued)															
253.149	5.07517	131.0878	0.0002	0.000021	0.0020	0.0030	0.000184	0.0056	0.00520	0.0162	0.0284	0.282	2	2015 08 05	06:20
253.149	5.07519	131.0898	0.0004	0.000032	0.0002	0.0030	0.000185	0.0052	0.00520	0.0162	0.0282	0.287	2	2015 08 05	06:33
253.151	5.07525	131.0906	0.0006	0.000015	0.0004	0.0031	0.000183	0.0052	0.00520	0.0162	0.0282	0.290	2	2015 08 05	06:46
253.149	5.09317	131.6455	0.0002	0.000014	0.0043	0.0030	0.000183	0.0070	0.00520	0.0163	0.0291	1.096	2	2015 08 05	07:37
253.150	5.09320	131.6422	0.0003	0.000023	0.0022	0.0030	0.000184	0.0058	0.00520	0.0163	0.0285	1.118	2	2015 08 05	07:50
253.151	5.09327	131.6465	0.0006	0.000030	0.0010	0.0031	0.000185	0.0053	0.00520	0.0163	0.0283	1.131	2	2015 08 05	08:03
253.149	5.10910	132.0574	0.0003	0.000027	0.0164	0.0030	0.000185	0.0180	0.00520	0.0164	0.0384	2.265	2	2015 08 05	08:53
253.149	5.10912	132.0397	0.0004	0.000026	0.0108	0.0030	0.000185	0.0126	0.00520	0.0164	0.0332	2.427	2	2015 08 05	09:06
253.151	5.10923	132.0409	0.0007	0.000024	0.0065	0.0031	0.000184	0.0087	0.00520	0.0164	0.0302	2.590	2	2015 08 05	09:19
253.149	5.12415	132.4234	0.0005	0.000029	0.0185	0.0030	0.000185	0.0200	0.00520	0.0164	0.0406	3.360	2	2015 08 05	10:09
253.149	5.12419	132.3947	0.0003	0.000042	0.0164	0.0030	0.000188	0.0179	0.00520	0.0164	0.0384	3.476	2	2015 08 05	10:22
253.151	5.12435	132.4030	0.0006	0.000049	0.0127	0.0031	0.000189	0.0144	0.00520	0.0164	0.0348	3.573	2	2015 08 05	10:35
253.148	5.13875	132.7844	0.0006	0.000023	0.0165	0.0031	0.000185	0.0180	0.00520	0.0164	0.0384	4.322	2	2015 08 05	11:26
253.148	5.13882	132.7745	0.0003	0.000052	0.0174	0.0030	0.000191	0.0189	0.00520	0.0164	0.0394	4.395	2	2015 08 05	11:39
253.150	5.13897	132.7512	0.0008	0.000045	0.0170	0.0031	0.000189	0.0185	0.00520	0.0164	0.0389	4.468	2	2015 08 05	11:52
253.148	5.15310	133.1532	0.0006	0.000027	0.0135	0.0031	0.000185	0.0152	0.00520	0.0165	0.0354	5.104	2	2015 08 05	12:43
253.148	5.15315	133.1349	0.0003	0.000044	0.0166	0.0030	0.000189	0.0182	0.00520	0.0165	0.0385	9.113	2	2015 08 05	12:56
253.150	5.15331	133.0868	0.0006	0.000049	0.0156	0.0031	0.000190	0.0172	0.00520	0.0165	0.0375	14.007	2	2015 08 05	13:09
253.149	5.16726	133.4946	0.0004	0.000014	0.0138	0.0030	0.000184	0.0155	0.00520	0.0165	0.0356	16.408	2	2015 08 05	13:59
253.149	5.16732	133.4934	0.0005	0.000043	0.0116	0.0030	0.000188	0.0133	0.00520	0.0165	0.0337	17.907	2	2015 08 05	14:12
253.151	5.16748	133.4658	0.0008	0.000048	0.0163	0.0031	0.000190	0.0179	0.00520	0.0165	0.0382	17.530	2	2015 08 05	14:25
253.149	5.18116	133.8682	0.0005	0.000017	0.0124	0.0030	0.000185	0.0141	0.00520	0.0166	0.0343	20.894	2	2015 08 05	15:15
253.149	5.18120	133.8316	0.0003	0.000051	0.0151	0.0030	0.000191	0.0167	0.00520	0.0166	0.0369	19.565	2	2015 08 05	15:28
253.150	5.18137	133.8375	0.0007	0.000048	0.0158	0.0031	0.000190	0.0173	0.00520	0.0166	0.0376	19.545	2	2015 08 05	15:41
253.149	5.19489	134.2012	0.0004	0.000024	0.0127	0.0030	0.000186	0.0144	0.00520	0.0166	0.0346	22.778	2	2015 08 05	16:32
253.149	5.19494	134.2072	0.0004	0.000038	0.0150	0.0030	0.000188	0.0166	0.00520	0.0166	0.0367	23.295	2	2015 08 05	16:45
253.151	5.19508	134.1930	0.0006	0.000045	0.0147	0.0031	0.000189	0.0163	0.00520	0.0166	0.0364	22.353	2	2015 08 05	16:58

Table S3. Experimental (p, rho, T, x) data and detailed uncertainty information for isotherms measured on the (0.49896 argon + 0.50104 carbon dioxide) mixture (continued)

test: Ar_CO2_1508c.dat (overnight evacuation prior to test; 30 min equilibration time; 3 replicates per pressure; replicate of Ar_CO2_1508b, but not as far into 2-phase
chiS [specific magnetic susceptibility] = -0.6035E-08 [m3/kg]

T (K)	p (MPa)	rho (kg.m-3)	sigma_T (K)	sigma_p (MPa)	sigma_rho (kg.m-3)	u(T) (K)	u(p) (MPa)	u(rho) (kg.m-3)	u(MW) (g/mol)	u[rho(x)] (kg.m-3)	U_c/% (k = 2)	m_sorb (mg)	p_trans	date	time
253.150	0.48988	9.9617	0.0009	0.000003	0.0008	0.0031	0.000150	0.0041	0.00520	0.0012	0.0850	-0.007	2	2015 08 06	11:11
253.148	0.48987	9.9632	0.0004	0.000004	0.0022	0.0030	0.000150	0.0046	0.00520	0.0012	0.0957	-0.007	2	2015 08 06	11:24
253.148	0.48990	9.9648	0.0003	0.000007	0.0006	0.0030	0.000150	0.0041	0.00520	0.0012	0.0862	-0.008	2	2015 08 06	11:37
253.148	1.10934	23.1532	0.0002	0.000013	0.0002	0.0030	0.000152	0.0063	0.00520	0.0029	0.0594	-0.013	2	2015 08 06	12:47
253.149	1.10916	23.1489	0.0008	0.000006	0.0002	0.0031	0.000152	0.0051	0.00520	0.0029	0.0502	-0.009	2	2015 08 06	13:00
253.152	1.10904	23.1476	0.0007	0.000006	0.0014	0.0031	0.000152	0.0045	0.00520	0.0029	0.0462	-0.007	2	2015 08 06	13:13
253.147	2.01090	43.7072	0.0003	0.000017	0.0005	0.0030	0.000156	0.0066	0.00520	0.0054	0.0390	-0.012	2	2015 08 06	14:21
253.150	2.01070	43.6893	0.0010	0.000007	0.0002	0.0032	0.000156	0.0053	0.00520	0.0054	0.0347	-0.008	2	2015 08 06	14:34
253.153	2.01054	43.7025	0.0004	0.000010	0.0006	0.0030	0.000156	0.0042	0.00520	0.0054	0.0314	-0.005	2	2015 08 06	14:47
253.147	3.02980	69.2738	0.0002	0.000013	0.0020	0.0030	0.000163	0.0064	0.00520	0.0086	0.0309	-0.008	2	2015 08 06	15:59
253.150	3.02949	69.2731	0.0010	0.000012	0.0009	0.0032	0.000162	0.0041	0.00520	0.0086	0.0275	-0.003	2	2015 08 06	16:12
253.153	3.02928	69.2788	0.0005	0.000009	0.0015	0.0030	0.000162	0.0032	0.00520	0.0086	0.0264	0.001	2	2015 08 06	16:27
253.147	4.02448	97.2605	0.0003	0.000013	0.0013	0.0030	0.000171	0.0049	0.00520	0.0120	0.0267	-0.002	2	2015 08 06	17:44
253.149	4.02414	97.2618	0.0009	0.000011	0.0013	0.0031	0.000171	0.0032	0.00520	0.0120	0.0256	0.004	2	2015 08 06	17:57
253.152	4.02397	97.2627	0.0008	0.000008	0.0014	0.0031	0.000171	0.0031	0.00520	0.0120	0.0256	0.007	2	2015 08 06	18:10
253.147	4.50025	111.9706	0.0003	0.000015	0.0015	0.0030	0.000176	0.0039	0.00520	0.0139	0.0257	0.003	2	2015 08 06	19:21
253.150	4.50018	111.9709	0.0010	0.000009	0.0019	0.0031	0.000176	0.0037	0.00520	0.0139	0.0257	0.005	2	2015 08 06	19:34
253.153	4.49964	111.9701	0.0006	0.000011	0.0003	0.0031	0.000176	0.0046	0.00520	0.0139	0.0261	0.015	2	2015 08 06	19:47
253.148	4.91162	125.5098	0.0002	0.000011	0.0008	0.0030	0.000181	0.0031	0.00520	0.0155	0.0253	0.009	2	2015 08 06	20:57
253.149	4.91113	125.5080	0.0008	0.000020	0.0008	0.0031	0.000181	0.0054	0.00520	0.0155	0.0262	0.017	2	2015 08 06	21:10
253.152	4.91102	125.5099	0.0007	0.000012	0.0006	0.0031	0.000181	0.0063	0.00520	0.0155	0.0267	0.020	2	2015 08 06	21:23
253.148	4.92997	126.1557	0.0002	0.000014	0.0011	0.0030	0.000181	0.0060	0.00520	0.0156	0.0265	0.019	2	2015 08 06	22:14
253.149	4.93020	126.1522	0.0007	0.000021	0.0004	0.0031	0.000182	0.0046	0.00520	0.0156	0.0258	0.016	2	2015 08 06	22:27
253.152	4.93017	126.1542	0.0007	0.000020	0.0003	0.0031	0.000182	0.0052	0.00520	0.0156	0.0261	0.017	2	2015 08 06	22:40
253.149	4.94843	126.7907	0.0001	0.000020	0.0005	0.0030	0.000182	0.0073	0.00520	0.0157	0.0274	0.022	2	2015 08 06	23:31
253.150	4.94921	126.7903	0.0005	0.000018	0.0002	0.0030	0.000182	0.0031	0.00520	0.0157	0.0252	0.010	2	2015 08 06	23:44
253.151	4.94831	126.7910	0.0005	0.000020	0.0005	0.0030	0.000182	0.0085	0.00520	0.0157	0.0282	0.025	2	2015 08 06	23:57
253.148	4.96726	127.4186	0.0002	0.000015	0.0004	0.0030	0.000182	0.0044	0.00520	0.0158	0.0257	0.015	2	2015 08 07	00:47
253.149	4.96759	127.4175	0.0005	0.000015	0.0002	0.0030	0.000182	0.0030	0.00520	0.0158	0.0252	0.010	2	2015 08 07	01:00
253.151	4.96690	127.4168	0.0005	0.000018	0.0005	0.0030	0.000182	0.0072	0.00520	0.0158	0.0272	0.022	2	2015 08 07	01:13
253.148	4.98504	128.0361	0.0002	0.000013	0.0004	0.0030	0.000182	0.0061	0.00520	0.0159	0.0265	0.020	2	2015 08 07	02:03
253.149	4.98522	128.0354	0.0006	0.000014	0.0008	0.0031	0.000182	0.0052	0.00520	0.0159	0.0261	0.017	2	2015 08 07	02:16
253.151	4.98473	128.0354	0.0006	0.000014	0.0005	0.0031	0.000182	0.0086	0.00520	0.0159	0.0282	0.025	2	2015 08 07	02:29
253.149	5.00262	128.6443	0.0002	0.000015	0.0002	0.0030	0.000182	0.0073	0.00520	0.0159	0.0273	0.023	2	2015 08 07	03:20
253.149	5.00213	128.6439	0.0004	0.000020	0.0004	0.0030	0.000183	0.0106	0.00520	0.0159	0.0297	0.030	2	2015 08 07	03:33
253.151	5.00273	128.6417	0.0005	0.000026	0.0004	0.0030	0.000183	0.0068	0.00520	0.0159	0.0270	0.021	2	2015 08 07	03:46
253.149	5.02002	129.2431	0.0001	0.000013	0.0003	0.0030	0.000182	0.0076	0.00520	0.0160	0.0274	0.023	2	2015 08 07	04:37
253.150	5.01953	129.2433	0.0004	0.000021	0.0005	0.0030	0.000183	0.0110	0.00520	0.0160	0.0300	0.031	2	2015 08 07	04:50
253.151	5.01954	129.2412	0.0003	0.000010	0.0004	0.0030	0.000182	0.0113	0.00520	0.0160	0.0303	0.031	2	2015 08 07	05:03
253.149	5.03566	129.8339	0.0001	0.000014	0.0003	0.0030	0.000183	0.0176	0.00520	0.0161	0.0367	0.044	2	2015 08 07	05:53
253.149	5.03607	129.8330	0.0004	0.000009	0.0009	0.0030	0.000182	0.0149	0.00520	0.0161	0.0338	0.039	2	2015 08 07	06:06
253.151	5.03643	129.8324	0.0005	0.000012	0.0009	0.0030	0.000182	0.0126	0.00520	0.0161	0.0315	0.034	2	2015 08 07	06:19
253.148	5.05020	130.4124	0.0004	0.000016	0.0010	0.0030	0.000183	0.0330	0.00520	0.0162	0.0563	0.071	2	2015 08 07	07:09
253.148	5.05128	130.4094	0.0003	0.000020	0.0011	0.0030	0.000183	0.0255	0.00520	0.0162	0.0462	0.058	2	2015 08 07	07:22
253.150	5.05013	130.4121	0.0007	0.000024	0.0010	0.0031	0.000184	0.0339	0.00520	0.0162	0.0576	0.072	2	2015 08 07	07:35

T (K)	p (MPa)	rho (kg.m−3)	sigma_T (K)	sigma_p (MPa)	sigma_rho (kg.m−3)	u(T) (K)	u(p) (MPa)	u(rho) (kg.m−3)	u(MW) (g/mol)	u[rho(x)] (kg.m−3)	U_c/% (k = 2)	m_sorb (mg)	p_trans	date	time
# test: Ar_CO2_1508c.dat (continued)															
253.149	5.04113	130.9710	0.0005	0.000016	0.0008	0.0030	0.000183	0.2111	0.00520	0.0162	0.3232	0.201	2	2015 08 07	08:26
253.148	5.04014	130.9665	0.0003	0.000017	0.0006	0.0030	0.000183	0.2180	0.00520	0.0162	0.3338	0.204	2	2015 08 07	08:39
253.150	5.03496	130.9671	0.0007	0.000023	0.0007	0.0031	0.000183	0.2548	0.00520	0.0162	0.3898	0.215	2	2015 08 07	08:52
253.149	5.12071	131.4739	0.0005	0.000029	0.0028	0.0030	0.000185	0.2407	0.00520	0.0163	0.3670	0.662	2	2015 08 07	09:43
253.149	5.11984	131.4710	0.0002	0.000022	0.0016	0.0030	0.000184	0.2347	0.00520	0.0163	0.3579	0.681	2	2015 08 07	09:56
253.150	5.11949	131.4707	0.0006	0.000022	0.0002	0.0031	0.000184	0.2319	0.00520	0.0163	0.3537	0.691	2	2015 08 07	10:09
253.149	5.12261	131.8565	0.0004	0.000032	0.0092	0.0030	0.000186	0.1556	0.00520	0.0163	0.2373	1.720	2	2015 08 07	10:59
253.149	5.12239	131.8505	0.0001	0.000021	0.0056	0.0030	0.000184	0.1542	0.00520	0.0163	0.2353	1.781	2	2015 08 07	11:12
253.150	5.12229	131.8430	0.0004	0.000019	0.0036	0.0030	0.000184	0.1532	0.00520	0.0163	0.2337	1.837	2	2015 08 07	11:25
253.149	5.13391	132.1697	0.0004	0.000031	0.0152	0.0030	0.000186	0.1452	0.00520	0.0164	0.2211	2.625	2	2015 08 07	12:15
253.149	5.13380	132.1581	0.0001	0.000012	0.0123	0.0030	0.000184	0.1441	0.00520	0.0164	0.2195	2.742	2	2015 08 07	12:28
253.150	5.13378	132.1470	0.0005	0.000040	0.0083	0.0030	0.000187	0.1430	0.00520	0.0164	0.2178	2.873	2	2015 08 07	12:41
253.149	5.14594	132.4724	0.0005	0.000020	0.0154	0.0030	0.000184	0.1423	0.00520	0.0164	0.2163	3.103	2	2015 08 07	13:32
253.148	5.14561	132.4602	0.0002	0.000038	0.0152	0.0030	0.000187	0.1400	0.00520	0.0164	0.2128	3.658	2	2015 08 07	13:45
253.150	5.14592	132.4400	0.0006	0.000029	0.0131	0.0031	0.000186	0.1409	0.00520	0.0164	0.2142	3.367	2	2015 08 07	13:58
253.149	5.15756	132.7662	0.0005	0.000037	0.0140	0.0030	0.000187	0.1378	0.00520	0.0164	0.2090	4.386	2	2015 08 07	14:49
253.148	5.15754	132.7481	0.0002	0.000045	0.0151	0.0030	0.000189	0.1377	0.00520	0.0164	0.2090	4.454	2	2015 08 07	15:02
253.150	5.15770	132.7396	0.0007	0.000038	0.0154	0.0031	0.000187	0.1377	0.00520	0.0164	0.2089	4.509	2	2015 08 07	15:15
253.149	5.16865	133.0657	0.0004	0.000022	0.0128	0.0030	0.000185	0.1308	0.00520	0.0165	0.1982	14.667	2	2015 08 07	16:05
253.148	5.16869	133.0511	0.0002	0.000037	0.0151	0.0030	0.000187	0.1311	0.00520	0.0165	0.1987	14.425	2	2015 08 07	16:18
253.150	5.16882	133.0325	0.0006	0.000035	0.0149	0.0031	0.000187	0.1310	0.00520	0.0165	0.1986	14.712	2	2015 08 07	16:31

Table S3. Experimental (p, rho, T, x) data and detailed uncertainty information for isotherms measured on the (0.49896 argon + 0.50104 carbon dioxide) mixture (continued)

test: Ar_CO2_1508ef.dat (long evacuation prior to test; 30 min equilibration time; 3 replicates per pressure)
chiS [specific magnetic susceptibility] = −0.6035E−08 [m3/kg]

T (K)	p (MPa)	rho (kg.m−3)	sigma_T (K)	sigma_p (MPa)	sigma_rho (kg.m−3)	u(T) (K)	u(p) (MPa)	u(rho) (kg.m−3)	u(MW) (g/mol)	u[rho(x)] (kg.m−3)	U_c/% (k = 2)	m_sorb (mg)	p_trans	date	time
253.150	0.47860	9.7365	0.0004	0.000006	0.0010	0.0030	0.000150	0.0020	0.00520	0.0012	0.0805	−0.018	2	2015 08 10	16:38
253.151	0.47859	9.7358	0.0007	0.000005	0.0004	0.0031	0.000150	0.0015	0.00520	0.0012	0.0750	−0.013	2	2015 08 10	16:51
253.154	0.47859	9.7349	0.0007	0.000003	0.0004	0.0031	0.000150	0.0015	0.00520	0.0012	0.0750	−0.013	2	2015 08 10	17:04
253.148	1.07918	22.5050	0.0003	0.000014	0.0004	0.0030	0.000152	0.0018	0.00520	0.0028	0.0419	−0.018	2	2015 08 10	18:04
253.149	1.07915	22.5044	0.0006	0.000007	0.0005	0.0031	0.000152	0.0019	0.00520	0.0028	0.0419	−0.015	2	2015 08 10	18:17
253.152	1.07914	22.5019	0.0008	0.000008	0.0010	0.0031	0.000152	0.0022	0.00520	0.0028	0.0432	−0.013	2	2015 08 10	18:30
253.147	2.03955	44.4013	0.0004	0.000013	0.0003	0.0030	0.000156	0.0024	0.00520	0.0055	0.0432	−0.018	2	2015 08 10	19:41
253.149	2.03953	44.4009	0.0010	0.000014	0.0004	0.0032	0.000156	0.0025	0.00520	0.0055	0.0321	−0.011	2	2015 08 10	19:54
253.152	2.03952	44.4009	0.0006	0.000010	0.0004	0.0031	0.000156	0.0025	0.00520	0.0055	0.0320	−0.011	2	2015 08 10	20:07
253.147	3.00663	68.6688	0.0003	0.000019	0.0009	0.0030	0.000163	0.0034	0.00520	0.0085	0.0320	−0.016	2	2015 08 10	21:20
253.149	3.00663	68.6709	0.0010	0.000012	0.0005	0.0032	0.000162	0.0032	0.00520	0.0085	0.0295	−0.009	2	2015 08 10	21:33
253.152	3.00662	68.6718	0.0005	0.000009	0.0007	0.0030	0.000162	0.0033	0.00520	0.0085	0.0295	−0.006	2	2015 08 10	21:46
253.146	4.02342	97.2343	0.0004	0.000017	0.0014	0.0030	0.000172	0.0044	0.00520	0.0120	0.0287	−0.010	2	2015 08 10	23:06
253.149	4.02343	97.2339	0.0010	0.000017	0.0011	0.0032	0.000172	0.0043	0.00520	0.0120	0.0287	−0.003	2	2015 08 10	23:19
253.153	4.02346	97.2341	0.0002	0.000014	0.0010	0.0030	0.000171	0.0042	0.00520	0.0120	0.0286	0.005	2	2015 08 10	23:38
253.148	4.50814	112.2175	0.0006	0.000032	0.0005	0.0031	0.000179	0.0046	0.00520	0.0139	0.0284	0.005	2	2015 08 11	00:59
253.151	4.50816	112.2161	0.0007	0.000013	0.0003	0.0031	0.000176	0.0046	0.00520	0.0139	0.0283	0.008	2	2015 08 11	01:12
253.148	4.53185	112.9772	0.0002	0.000012	0.0002	0.0030	0.000176	0.0046	0.00520	0.0140	0.0283	0.002	2	2015 08 11	02:03
253.149	4.53185	112.9775	0.0006	0.000030	0.0003	0.0031	0.000179	0.0046	0.00520	0.0140	0.0283	0.018	2	2015 08 11	02:16
253.151	4.53189	112.9767	0.0004	0.000018	0.0004	0.0030	0.000177	0.0046	0.00520	0.0140	0.0283	0.014	2	2015 08 11	02:29
253.148	4.55516	113.7251	0.0002	0.000012	0.0003	0.0030	0.000177	0.0046	0.00520	0.0141	0.0283	0.003	2	2015 08 11	03:20
253.149	4.55518	113.7245	0.0004	0.000018	0.0003	0.0030	0.000177	0.0046	0.00520	0.0141	0.0283	0.002	2	2015 08 11	03:33
253.151	4.55520	113.7235	0.0003	0.000025	0.0006	0.0030	0.000178	0.0047	0.00520	0.0141	0.0283	−0.003	2	2015 08 11	03:46
253.149	4.57802	114.4582	0.0001	0.000016	0.0002	0.0030	0.000177	0.0047	0.00520	0.0142	0.0283	0.005	2	2015 08 11	04:37
253.150	4.57805	114.4605	0.0004	0.000026	0.0004	0.0030	0.000178	0.0047	0.00520	0.0142	0.0283	0.004	2	2015 08 11	04:50
253.151	4.57806	114.4615	0.0002	0.000021	0.0008	0.0030	0.000178	0.0047	0.00520	0.0142	0.0283	0.015	2	2015 08 11	05:03
253.149	4.60043	115.1863	0.0001	0.000024	0.0010	0.0030	0.000178	0.0048	0.00520	0.0143	0.0284	0.003	2	2015 08 11	05:53
253.150	4.60046	115.1856	0.0003	0.000017	0.0006	0.0030	0.000178	0.0047	0.00520	0.0143	0.0283	0.011	2	2015 08 11	06:06
253.151	4.60047	115.1847	0.0001	0.000024	0.0005	0.0030	0.000178	0.0047	0.00520	0.0143	0.0283	−0.002	2	2015 08 11	06:19
253.149	4.62240	115.8967	0.0000	0.000015	0.0005	0.0030	0.000178	0.0047	0.00520	0.0144	0.0283	0.003	2	2015 08 11	07:09
253.150	4.62241	115.8978	0.0001	0.000020	0.0007	0.0030	0.000178	0.0048	0.00520	0.0144	0.0283	0.010	2	2015 08 11	07:22
253.150	4.62241	115.8978	0.0001	0.000023	0.0004	0.0030	0.000178	0.0047	0.00520	0.0144	0.0283	0.012	2	2015 08 11	07:35
253.150	4.64390	116.5948	0.0001	0.000022	0.0004	0.0030	0.000179	0.0047	0.00520	0.0144	0.0283	0.002	2	2015 08 11	08:26
253.150	4.64387	116.5977	0.0001	0.000017	0.0002	0.0030	0.000178	0.0047	0.00520	0.0144	0.0283	0.017	2	2015 08 11	08:39
253.150	4.64390	116.5978	0.0001	0.000010	0.0007	0.0030	0.000178	0.0048	0.00520	0.0144	0.0283	0.008	2	2015 08 11	08:52
253.149	4.66495	117.2865	0.0002	0.000015	0.0010	0.0030	0.000178	0.0049	0.00520	0.0145	0.0283	0.013	2	2015 08 11	09:43
253.149	4.66498	117.2865	0.0001	0.000020	0.0005	0.0030	0.000179	0.0048	0.00520	0.0145	0.0283	0.011	2	2015 08 11	09:56
253.150	4.66499	117.2825	0.0003	0.000031	0.0004	0.0030	0.000180	0.0048	0.00520	0.0145	0.0283	0.011	2	2015 08 11	10:09
253.149	4.68566	117.9616	0.0005	0.000009	0.0005	0.0030	0.000178	0.0048	0.00520	0.0146	0.0283	0.008	2	2015 08 11	10:59
253.148	4.68564	117.9630	0.0002	0.000023	0.0007	0.0030	0.000179	0.0048	0.00520	0.0146	0.0283	−0.003	2	2015 08 11	11:12
253.149	4.68567	117.9614	0.0006	0.000020	0.0003	0.0031	0.000179	0.0048	0.00520	0.0146	0.0283	0.012	2	2015 08 11	11:25
253.149	4.70593	118.6258	0.0005	0.000015	0.0014	0.0030	0.000179	0.0050	0.00520	0.0147	0.0284	0.001	2	2015 08 11	12:15
253.148	4.70593	118.6291	0.0002	0.000019	0.0003	0.0030	0.000179	0.0048	0.00520	0.0147	0.0283	−0.002	2	2015 08 11	12:28
253.149	4.70595	118.6274	0.0006	0.000019	0.0004	0.0031	0.000179	0.0048	0.00520	0.0147	0.0283	0.005	2	2015 08 11	12:41
253.149	4.72585	119.2867	0.0005	0.000017	0.0001	0.0030	0.000179	0.0048	0.00520	0.0148	0.0282	0.012	2	2015 08 11	13:32
253.148	4.72583	119.2846	0.0002	0.000018	0.0006	0.0030	0.000179	0.0049	0.00520	0.0148	0.0283	0.012	2	2015 08 11	13:45
253.149	4.72587	119.2849	0.0005	0.000018	0.0006	0.0030	0.000179	0.0049	0.00520	0.0148	0.0283	0.013	2	2015 08 11	13:58
253.149	4.74544	119.9318	0.0005	0.000024	0.0003	0.0030	0.000180	0.0048	0.00520	0.0149	0.0283	−0.003	2	2015 08 11	14:49
253.148	4.74543	119.9326	0.0002	0.000019	0.0002	0.0030	0.000179	0.0048	0.00520	0.0149	0.0282	0.010	2	2015 08 11	15:02
253.150	4.74546	119.9311	0.0008	0.000032	0.0005	0.0031	0.000181	0.0049	0.00520	0.0149	0.0283	0.016	2	2015 08 11	15:15

test: Ar_CO2_1508ef.dat (continued)

T (K)	p (MPa)	rho (kg.m-3)	sigma_T (K)	sigma_p (MPa)	sigma_rho (kg.m-3)	u(T) (K)	u(p) (MPa)	u(rho) (kg.m-3)	u(MW) (g/mol)	u[rho(x)] (kg.m-3)	U_c/% (k = 2)	m_sorb (mg)	p_trans	date	time
253.150	4.76478	120.5701	0.0005	0.000021	0.0011	0.0030	0.000180	0.0050	0.00520	0.0149	0.0283	0.009	2	2015 08 11	16:05
253.149	4.76481	120.5709	0.0003	0.000016	0.0002	0.0030	0.000179	0.0049	0.00520	0.0149	0.0282	0.003	2	2015 08 11	16:18
253.151	4.76484	120.5743	0.0006	0.000030	0.0005	0.0031	0.000181	0.0049	0.00520	0.0149	0.0283	0.016	2	2015 08 11	16:31
253.150	4.78382	121.2041	0.0002	0.000015	0.0002	0.0030	0.000179	0.0049	0.00520	0.0150	0.0282	0.003	2	2015 08 11	17:21
253.150	4.78381	121.2028	0.0003	0.000019	0.0007	0.0030	0.000180	0.0049	0.00520	0.0150	0.0283	0.001	2	2015 08 11	17:34
253.151	4.78387	121.2031	0.0006	0.000018	0.0001	0.0031	0.000180	0.0049	0.00520	0.0150	0.0282	0.006	2	2015 08 11	17:47
253.149	4.80241	121.8235	0.0002	0.000010	0.0004	0.0030	0.000179	0.0049	0.00520	0.0151	0.0282	0.010	2	2015 08 11	18:38
253.150	4.80241	121.8214	0.0004	0.000009	0.0004	0.0030	0.000179	0.0049	0.00520	0.0151	0.0282	0.009	2	2015 08 11	18:51
253.151	4.80244	121.8215	0.0005	0.000015	0.0005	0.0030	0.000180	0.0049	0.00520	0.0151	0.0282	0.016	2	2015 08 11	19:04
253.150	4.82064	122.4331	0.0002	0.000025	0.0006	0.0030	0.000181	0.0050	0.00520	0.0152	0.0283	0.018	2	2015 08 11	19:55
253.150	4.82064	122.4309	0.0003	0.000017	0.0003	0.0030	0.000180	0.0049	0.00520	0.0152	0.0282	0.017	2	2015 08 11	20:08
253.151	4.82066	122.4290	0.0003	0.000010	0.0004	0.0030	0.000180	0.0049	0.00520	0.0152	0.0282	0.010	2	2015 08 11	20:21
253.150	4.83847	123.0265	0.0002	0.000016	0.0005	0.0030	0.000180	0.0050	0.00520	0.0152	0.0282	0.011	2	2015 08 11	21:11
253.150	4.83845	123.0253	0.0002	0.000022	0.0006	0.0030	0.000181	0.0050	0.00520	0.0152	0.0282	0.011	2	2015 08 11	21:24
253.151	4.83849	123.0267	0.0003	0.000013	0.0004	0.0030	0.000180	0.0049	0.00520	0.0152	0.0282	0.011	2	2015 08 11	21:37
253.150	4.85598	123.6149	0.0003	0.000013	0.0003	0.0030	0.000180	0.0050	0.00520	0.0153	0.0282	0.013	2	2015 08 11	22:27
253.150	4.85596	123.6134	0.0002	0.000014	0.0004	0.0030	0.000180	0.0050	0.00520	0.0153	0.0282	0.005	2	2015 08 11	22:40
253.151	4.85599	123.6133	0.0003	0.000019	0.0002	0.0030	0.000181	0.0050	0.00520	0.0153	0.0282	0.013	2	2015 08 11	22:53
253.150	4.87315	124.1956	0.0003	0.000017	0.0003	0.0030	0.000181	0.0050	0.00520	0.0154	0.0282	0.004	2	2015 08 11	23:44
253.150	4.87313	124.1920	0.0002	0.000026	0.0009	0.0030	0.000182	0.0051	0.00520	0.0154	0.0283	0.022	2	2015 08 11	23:57
253.151	4.87317	124.1912	0.0003	0.000020	0.0003	0.0030	0.000181	0.0050	0.00520	0.0154	0.0282	0.008	2	2015 08 12	00:10
253.150	4.89002	124.7603	0.0001	0.000009	0.0005	0.0030	0.000180	0.0050	0.00520	0.0155	0.0282	0.013	2	2015 08 12	01:01
253.151	4.89004	124.7630	0.0003	0.000031	0.0004	0.0030	0.000183	0.0050	0.00520	0.0155	0.0283	0.007	2	2015 08 12	01:14
253.152	4.89005	124.7592	0.0003	0.000025	0.0004	0.0030	0.000182	0.0050	0.00520	0.0155	0.0282	-0.000	2	2015 08 12	01:27
253.150	4.90664	125.3209	0.0003	0.000014	0.0004	0.0030	0.000181	0.0050	0.00520	0.0155	0.0282	0.011	2	2015 08 12	02:17
253.150	4.90662	125.3210	0.0002	0.000013	0.0004	0.0030	0.000181	0.0050	0.00520	0.0155	0.0282	0.004	2	2015 08 12	02:30
253.151	4.90664	125.3218	0.0002	0.000019	0.0007	0.0030	0.000181	0.0051	0.00520	0.0155	0.0282	0.020	2	2015 08 12	02:43
253.150	4.92295	125.8751	0.0002	0.000013	0.0003	0.0030	0.000181	0.0050	0.00520	0.0156	0.0282	0.014	2	2015 08 12	03:33
253.151	4.92294	125.8725	0.0003	0.000021	0.0004	0.0030	0.000182	0.0050	0.00520	0.0156	0.0282	0.002	2	2015 08 12	03:46
253.151	4.92298	125.8771	0.0003	0.000019	0.0012	0.0030	0.000182	0.0052	0.00520	0.0156	0.0283	0.010	2	2015 08 12	03:59
253.150	4.93899	126.4203	0.0002	0.000023	0.0007	0.0030	0.000182	0.0051	0.00520	0.0157	0.0282	0.008	2	2015 08 12	04:50
253.150	4.93901	126.4200	0.0003	0.000018	0.0003	0.0030	0.000182	0.0050	0.00520	0.0157	0.0282	0.022	2	2015 08 12	05:03
253.152	4.93904	126.4199	0.0004	0.000016	0.0005	0.0030	0.000181	0.0051	0.00520	0.0157	0.0282	0.017	2	2015 08 12	05:16
253.150	4.95472	126.9558	0.0002	0.000019	0.0003	0.0030	0.000182	0.0051	0.00520	0.0157	0.0282	0.005	2	2015 08 12	06:07
253.150	4.95476	126.9561	0.0004	0.000020	0.0011	0.0030	0.000182	0.0052	0.00520	0.0157	0.0283	0.014	2	2015 08 12	06:20
253.151	4.95478	126.9563	0.0005	0.000018	0.0006	0.0030	0.000182	0.0051	0.00520	0.0157	0.0282	0.017	2	2015 08 12	06:33
253.149	4.95479	126.9587	0.0005	0.000026	0.0005	0.0030	0.000183	0.0051	0.00520	0.0157	0.0282	0.017	2	2015 08 12	10:12
253.152	4.95484	126.9578	0.0007	0.000023	0.0004	0.0031	0.000182	0.0051	0.00520	0.0157	0.0282	0.020	2	2015 08 12	10:25
253.153	4.95487	126.9577	0.0002	0.000022	0.0010	0.0030	0.000182	0.0052	0.00520	0.0157	0.0283	0.025	2	2015 08 12	10:38
253.150	4.97016	127.4823	0.0004	0.000014	0.0009	0.0030	0.000182	0.0052	0.00520	0.0158	0.0282	0.020	2	2015 08 12	11:29
253.151	4.97022	127.4846	0.0005	0.000022	0.0007	0.0030	0.000183	0.0051	0.00520	0.0158	0.0282	0.009	2	2015 08 12	11:42
253.153	4.97027	127.4843	0.0002	0.000024	0.0014	0.0030	0.000183	0.0053	0.00520	0.0158	0.0283	0.019	2	2015 08 12	11:55
253.149	4.98528	128.0015	0.0002	0.000017	0.0005	0.0030	0.000182	0.0051	0.00520	0.0159	0.0282	0.012	2	2015 08 12	12:46
253.151	4.98529	128.0008	0.0006	0.000026	0.0003	0.0031	0.000183	0.0051	0.00520	0.0159	0.0282	0.020	2	2015 08 12	12:59
253.152	4.98533	128.0002	0.0002	0.000016	0.0010	0.0030	0.000182	0.0052	0.00520	0.0159	0.0283	0.026	2	2015 08 12	13:12
253.149	5.00001	128.5073	0.0003	0.000014	0.0004	0.0030	0.000182	0.0051	0.00520	0.0159	0.0282	0.020	2	2015 08 12	14:03
253.151	5.00006	128.5074	0.0005	0.000019	0.0001	0.0030	0.000183	0.0051	0.00520	0.0159	0.0282	0.018	2	2015 08 12	14:16
253.152	5.00008	128.5100	0.0001	0.000012	0.0004	0.0030	0.000182	0.0051	0.00520	0.0159	0.0282	0.021	2	2015 08 12	14:29
253.150	5.01452	129.0061	0.0004	0.000015	0.0004	0.0030	0.000182	0.0051	0.00520	0.0160	0.0282	0.014	2	2015 08 12	15:20
253.151	5.01457	129.0056	0.0005	0.000007	0.0007	0.0030	0.000182	0.0052	0.00520	0.0160	0.0282	0.026	2	2015 08 12	15:33
253.152	5.01456	129.0048	0.0002	0.000021	0.0009	0.0030	0.000183	0.0052	0.00520	0.0160	0.0282	0.018	2	2015 08 12	15:46
253.150	5.02875	129.4973	0.0003	0.000013	0.0003	0.0030	0.000182	0.0051	0.00520	0.0160	0.0282	0.021	2	2015 08 12	16:37
253.151	5.02878	129.4944	0.0004	0.000014	0.0005	0.0030	0.000182	0.0052	0.00520	0.0160	0.0282	0.019	2	2015 08 12	16:50
253.152	5.02881	129.4982	0.0002	0.000024	0.0010	0.0030	0.000183	0.0052	0.00520	0.0160	0.0283	0.020	2	2015 08 12	17:03

T	p	rho	sigma_T	sigma_p	sigma_rho	u(T)	u(p)	u(rho)	u(MW)	u[rho(x)]	U_c/%	m_sorb	p_trans	date	time
(K)	(MPa)	(kg.m-3)	(K)	(MPa)	(kg.m-3)	(K)	(MPa)	(kg.m-3)	(g/mol)	(kg.m-3)	(k = 2)	(mg)			
# test: Ar_CO2_1508ef.dat (continued)															
253.149	5.04272	129.9795	0.0002	0.000021	0.0008	0.0030	0.000183	0.0052	0.00520	0.0161	0.0282	0.025	2	2015 08 12	17:53
253.150	5.04274	129.9774	0.0005	0.000019	0.0008	0.0030	0.000183	0.0052	0.00520	0.0161	0.0282	0.028	2	2015 08 12	18:06
253.152	5.04276	129.9770	0.0003	0.000009	0.0004	0.0030	0.000182	0.0052	0.00520	0.0161	0.0282	0.036	2	2015 08 12	18:19
253.149	5.05644	130.4526	0.0003	0.000031	0.0008	0.0030	0.000185	0.0052	0.00520	0.0162	0.0283	0.028	2	2015 08 12	19:09
253.151	5.05646	130.4496	0.0006	0.000028	0.0008	0.0031	0.000184	0.0052	0.00520	0.0162	0.0283	0.043	2	2015 08 12	19:22
253.152	5.05649	130.4496	0.0002	0.000032	0.0005	0.0030	0.000185	0.0052	0.00520	0.0162	0.0282	0.028	2	2015 08 12	19:35
253.150	5.06990	130.9121	0.0005	0.000028	0.0004	0.0030	0.000185	0.0052	0.00520	0.0162	0.0282	0.039	2	2015 08 12	20:26
253.152	5.06992	130.9090	0.0005	0.000024	0.0008	0.0030	0.000184	0.0053	0.00520	0.0162	0.0283	0.050	2	2015 08 12	20:39
253.153	5.06992	130.9116	0.0001	0.000025	0.0004	0.0030	0.000184	0.0052	0.00520	0.0162	0.0282	0.054	2	2015 08 12	20:52
253.150	5.08278	131.3485	0.0005	0.000032	0.0016	0.0030	0.000185	0.0055	0.00520	0.0163	0.0284	0.112	2	2015 08 12	21:43
253.151	5.08285	131.3433	0.0004	0.000013	0.0004	0.0030	0.000183	0.0052	0.00520	0.0163	0.0282	0.108	2	2015 08 12	21:56
253.152	5.08285	131.3418	0.0002	0.000012	0.0004	0.0030	0.000183	0.0052	0.00520	0.0163	0.0282	0.101	2	2015 08 12	22:09
253.150	5.09489	131.7160	0.0005	0.000026	0.0020	0.0030	0.000185	0.0057	0.00520	0.0163	0.0284	0.322	2	2015 08 12	22:59
253.152	5.09494	131.7086	0.0003	0.000023	0.0004	0.0030	0.000184	0.0052	0.00520	0.0163	0.0282	0.324	2	2015 08 12	23:12
253.152	5.09492	131.7086	0.0002	0.000024	0.0014	0.0030	0.000184	0.0054	0.00520	0.0163	0.0283	0.314	2	2015 08 12	23:25
253.151	5.10582	132.0213	0.0004	0.000021	0.0098	0.0030	0.000184	0.0116	0.00520	0.0164	0.0323	0.625	2	2015 08 13	00:15
253.152	5.10584	131.9919	0.0002	0.000020	0.0043	0.0030	0.000184	0.0070	0.00520	0.0163	0.0291	0.661	2	2015 08 13	00:28
253.152	5.10582	131.9903	0.0002	0.000039	0.0021	0.0030	0.000187	0.0057	0.00520	0.0163	0.0285	0.670	2	2015 08 13	00:41
253.151	5.11618	132.2500	0.0003	0.000030	0.0098	0.0030	0.000185	0.0116	0.00520	0.0164	0.0323	0.903	2	2015 08 13	01:32
253.152	5.11618	132.2414	0.0001	0.000022	0.0068	0.0030	0.000184	0.0090	0.00520	0.0164	0.0303	0.956	2	2015 08 13	01:45
253.152	5.11614	132.2421	0.0001	0.000015	0.0040	0.0030	0.000184	0.0068	0.00520	0.0164	0.0290	0.998	2	2015 08 13	01:58
253.151	5.12627	132.5306	0.0003	0.000016	0.0144	0.0030	0.000184	0.0160	0.00520	0.0164	0.0363	1.218	2	2015 08 13	02:49
253.151	5.12631	132.4814	0.0002	0.000012	0.0085	0.0030	0.000184	0.0105	0.00520	0.0164	0.0313	1.238	2	2015 08 13	03:02
253.151	5.12626	132.4773	0.0002	0.000034	0.0061	0.0030	0.000186	0.0084	0.00520	0.0164	0.0299	1.271	2	2015 08 13	03:15
253.151	5.13627	132.7703	0.0005	0.000031	0.0149	0.0030	0.000186	0.0165	0.00520	0.0164	0.0367	1.512	2	2015 08 13	04:05
253.152	5.13630	132.7231	0.0002	0.000018	0.0107	0.0030	0.000184	0.0125	0.00520	0.0164	0.0330	1.531	2	2015 08 13	04:18
253.152	5.13625	132.7143	0.0003	0.000026	0.0069	0.0030	0.000185	0.0091	0.00520	0.0164	0.0303	1.545	2	2015 08 13	04:31
253.151	5.14606	132.9701	0.0001	0.000017	0.0132	0.0030	0.000184	0.0149	0.00520	0.0165	0.0351	1.824	2	2015 08 13	05:21
253.151	5.14604	132.9575	0.0002	0.000023	0.0100	0.0030	0.000185	0.0119	0.00520	0.0165	0.0324	1.841	2	2015 08 13	05:34
253.151	5.14598	132.9561	0.0003	0.000034	0.0086	0.0030	0.000186	0.0106	0.00520	0.0165	0.0315	1.843	2	2015 08 13	05:47
253.151	5.15568	133.2132	0.0001	0.000019	0.0118	0.0030	0.000184	0.0136	0.00520	0.0165	0.0339	2.081	2	2015 08 13	06:38
253.151	5.15565	133.2038	0.0001	0.000026	0.0090	0.0030	0.000185	0.0110	0.00520	0.0165	0.0317	2.102	2	2015 08 13	06:51
253.150	5.15560	133.1823	0.0002	0.000020	0.0115	0.0030	0.000185	0.0133	0.00520	0.0165	0.0336	2.088	2	2015 08 13	07:04
253.151	5.16515	133.4591	0.0003	0.000019	0.0098	0.0030	0.000185	0.0117	0.00520	0.0165	0.0322	5.808	2	2015 08 13	07:55
253.150	5.16508	133.4386	0.0004	0.000018	0.0085	0.0030	0.000184	0.0105	0.00520	0.0165	0.0313	2.293	2	2015 08 13	08:08
253.149	5.16505	133.4365	0.0001	0.000022	0.0089	0.0030	0.000185	0.0108	0.00520	0.0165	0.0316	6.068	2	2015 08 13	08:21
253.152	5.17453	133.6924	0.0003	0.000022	0.0099	0.0030	0.000185	0.0118	0.00520	0.0166	0.0323	7.470	2	2015 08 13	09:11
253.151	5.17447	133.7009	0.0003	0.000025	0.0094	0.0030	0.000185	0.0113	0.00520	0.0166	0.0319	6.552	2	2015 08 13	09:24
253.150	5.17445	133.6614	0.0001	0.000015	0.0094	0.0030	0.000184	0.0113	0.00520	0.0166	0.0319	11.646	2	2015 08 13	09:37
253.151	5.18380	133.9102	0.0002	0.000022	0.0100	0.0030	0.000185	0.0119	0.00520	0.0166	0.0324	12.165	2	2015 08 13	10:30
253.150	5.18376	133.8905	0.0001	0.000012	0.0095	0.0030	0.000184	0.0115	0.00520	0.0166	0.0320	13.190	2	2015 08 13	10:43
253.150	5.18377	133.8886	0.0001	0.000007	0.0029	0.0030	0.000184	0.0062	0.00520	0.0166	0.0286	12.687	2	2015 08 13	10:56
253.150	5.19293	134.1567	0.0003	0.000019	0.0068	0.0030	0.000185	0.0090	0.00520	0.0166	0.0302	14.227	2	2015 08 13	12:00
253.150	5.19289	134.1487	0.0001	0.000020	0.0091	0.0030	0.000185	0.0111	0.00520	0.0166	0.0317	14.593	2	2015 08 13	12:13
253.150	5.19297	134.1396	0.0003	0.000041	0.0099	0.0030	0.000188	0.0118	0.00520	0.0166	0.0323	15.272	2	2015 08 13	12:26
253.150	5.20196	134.3686	0.0005	0.000017	0.0076	0.0030	0.000185	0.0098	0.00520	0.0166	0.0307	16.912	2	2015 08 13	13:17
253.149	5.20197	134.3756	0.0001	0.000024	0.0086	0.0030	0.000186	0.0106	0.00520	0.0166	0.0313	16.952	2	2015 08 13	13:30
253.150	5.20205	134.3397	0.0005	0.000037	0.0100	0.0030	0.000188	0.0119	0.00520	0.0166	0.0324	16.393	2	2015 08 13	13:43

Table S3. Experimental (p, rho, T, x) data and detailed uncertainty information for isotherms measured on the (0.49896 argon + 0.50104 carbon dioxide) mixture (continued)

T (K)	p (MPa)	rho (kg.m−3)	sigma_T (K)	sigma_p (MPa)	sigma_rho (kg.m−3)	u(T) (K)	u(p) (MPa)	u(rho) (kg.m−3)	u(MW) (g/mol)	u[rho(x)] (kg.m−3)	U_c/% (k = 2)	m_sorb (mg)	p_trans	date	time
# test: Ar_CO2_1508gh.dat (brief purge prior to test; starting p = 4.46 MPa; 30 min equilibration time; 3 replicates per pressure)															
# chiS [specific magnetic susceptibility] = −0.6035E−08 [m3/kg]															
253.145	4.46141	110.7407	0.0009	0.000015	0.0010	0.0031	0.000176	0.0047	0.00520	0.0137	0.0284	0.006	2	2015 08 13	15:24
253.149	4.46148	110.7448	0.0013	0.000041	0.0003	0.0033	0.000180	0.0045	0.00520	0.0137	0.0285	0.008	2	2015 08 13	15:37
253.153	4.46156	110.7389	0.0002	0.000016	0.0005	0.0030	0.000176	0.0046	0.00520	0.0137	0.0283	0.018	2	2015 08 13	15:56
253.148	4.89867	125.0614	0.0002	0.000019	0.0007	0.0030	0.000181	0.0051	0.00520	0.0155	0.0282	0.004	2	2015 08 13	17:13
253.149	4.89867	125.0612	0.0005	0.000015	0.0008	0.0030	0.000181	0.0051	0.00520	0.0155	0.0282	0.017	2	2015 08 13	17:26
253.151	4.89869	125.0609	0.0006	0.000024	0.0004	0.0031	0.000182	0.0050	0.00520	0.0155	0.0283	0.016	2	2015 08 13	17:39
253.153	5.04727	130.1407	0.0004	0.000018	0.0010	0.0030	0.000183	0.0053	0.00520	0.0161	0.0283	0.032	2	2015 08 13	19:01
253.151	5.04719	130.1418	0.0009	0.000041	0.0009	0.0031	0.000187	0.0053	0.00520	0.0161	0.0283	0.045	2	2015 08 13	19:14
253.148	5.04711	130.1408	0.0006	0.000018	0.0010	0.0031	0.000183	0.0053	0.00520	0.0161	0.0283	0.027	2	2015 08 13	19:27
253.152	5.07912	131.2291	0.0003	0.000016	0.0010	0.0030	0.000183	0.0053	0.00520	0.0163	0.0282	0.109	2	2015 08 13	20:19
253.150	5.07901	131.2294	0.0006	0.000034	0.0004	0.0031	0.000186	0.0052	0.00520	0.0163	0.0283	0.121	2	2015 08 13	20:32
253.148	5.07893	131.2286	0.0003	0.000013	0.0008	0.0030	0.000183	0.0053	0.00520	0.0163	0.0282	0.114	2	2015 08 13	20:45
253.151	5.10646	132.0145	0.0006	0.000054	0.0140	0.0031	0.000191	0.0156	0.00520	0.0164	0.0360	0.948	2	2015 08 13	21:38
253.149	5.10633	132.0015	0.0005	0.000029	0.0115	0.0030	0.000185	0.0133	0.00520	0.0163	0.0338	0.977	2	2015 08 13	21:51
253.148	5.10628	131.9832	0.0002	0.000014	0.0091	0.0030	0.000183	0.0111	0.00520	0.0163	0.0318	1.001	2	2015 08 13	22:04
253.150	5.12999	132.6282	0.0007	0.000066	0.0173	0.0031	0.000195	0.0188	0.00520	0.0164	0.0394	1.536	2	2015 08 13	22:56
253.148	5.12989	132.6327	0.0002	0.000020	0.0141	0.0030	0.000184	0.0158	0.00520	0.0164	0.0360	1.529	2	2015 08 13	23:09
253.148	5.12995	132.6053	0.0004	0.000033	0.0140	0.0030	0.000186	0.0156	0.00520	0.0164	0.0359	1.523	2	2015 08 13	23:22
253.149	5.15259	133.2618	0.0009	0.000042	0.0144	0.0031	0.000188	0.0160	0.00520	0.0165	0.0363	2.057	2	2015 08 14	00:15
253.147	5.15253	133.2254	0.0002	0.000020	0.0160	0.0030	0.000184	0.0176	0.00520	0.0165	0.0378	2.030	2	2015 08 14	00:28
253.148	5.15264	133.2229	0.0007	0.000066	0.0171	0.0031	0.000195	0.0186	0.00520	0.0165	0.0390	2.012	2	2015 08 14	00:41
253.149	5.17437	133.8250	0.0008	0.000045	0.0135	0.0031	0.000189	0.0152	0.00520	0.0166	0.0354	2.372	2	2015 08 14	01:33
253.147	5.17437	133.8250	0.0001	0.000029	0.0153	0.0030	0.000186	0.0168	0.00520	0.0166	0.0370	2.357	2	2015 08 14	01:46
253.149	5.17450	133.7920	0.0007	0.000054	0.0183	0.0031	0.000192	0.0198	0.00520	0.0166	0.0402	2.338	2	2015 08 14	01:59
253.148	5.19541	134.4093	0.0007	0.000035	0.0127	0.0031	0.000187	0.0144	0.00520	0.0166	0.0346	6.487	2	2015 08 14	02:52
253.148	5.19545	134.3789	0.0002	0.000046	0.0156	0.0030	0.000190	0.0172	0.00520	0.0166	0.0373	11.391	2	2015 08 14	03:05
253.150	5.19564	134.3637	0.0008	0.000053	0.0168	0.0031	0.000191	0.0183	0.00520	0.0166	0.0385	12.151	2	2015 08 14	03:18
253.148	5.21569	134.9502	0.0004	0.000018	0.0144	0.0030	0.000185	0.0160	0.00520	0.0167	0.0360	13.534	2	2015 08 14	04:10
253.148	5.21578	134.9157	0.0005	0.000052	0.0162	0.0030	0.000191	0.0178	0.00520	0.0167	0.0378	14.074	2	2015 08 14	04:23
253.150	5.21596	134.9047	0.0007	0.000046	0.0151	0.0031	0.000190	0.0167	0.00520	0.0167	0.0367	13.614	2	2015 08 14	04:36
253.148	5.23520	135.4467	0.0003	0.000018	0.0123	0.0030	0.000185	0.0140	0.00520	0.0168	0.0341	15.438	2	2015 08 14	05:29
253.149	5.23527	135.4582	0.0005	0.000031	0.0154	0.0030	0.000187	0.0170	0.00520	0.0168	0.0369	15.661	2	2015 08 14	05:42
253.151	5.23540	135.4443	0.0006	0.000045	0.0139	0.0031	0.000190	0.0155	0.00520	0.0168	0.0356	16.101	2	2015 08 14	05:55
253.148	5.25379	135.9632	0.0003	0.000016	0.0124	0.0030	0.000185	0.0141	0.00520	0.0168	0.0341	17.654	2	2015 08 14	06:47
253.148	5.25384	135.9604	0.0005	0.000053	0.0155	0.0030	0.000192	0.0171	0.00520	0.0168	0.0371	17.358	2	2015 08 14	07:00
253.151	5.25403	135.9467	0.0007	0.000061	0.0138	0.0031	0.000194	0.0154	0.00520	0.0168	0.0355	17.939	2	2015 08 14	07:13
253.147	5.27163	136.4693	0.0002	0.000023	0.0137	0.0030	0.000186	0.0154	0.00520	0.0169	0.0352	18.459	2	2015 08 14	08:06
253.148	5.27175	136.4388	0.0006	0.000057	0.0154	0.0031	0.000193	0.0171	0.00520	0.0169	0.0370	18.235	2	2015 08 14	08:19
253.151	5.27194	136.4558	0.0007	0.000061	0.0126	0.0031	0.000195	0.0144	0.00520	0.0169	0.0345	19.997	2	2015 08 14	08:32
253.147	5.28871	136.9206	0.0002	0.000020	0.0124	0.0030	0.000186	0.0141	0.00520	0.0170	0.0340	20.199	2	2015 08 14	09:25
253.148	5.28884	136.9073	0.0008	0.000064	0.0152	0.0031	0.000196	0.0168	0.00520	0.0170	0.0367	19.763	2	2015 08 14	09:38
253.151	5.28903	136.9051	0.0007	0.000042	0.0123	0.0031	0.000190	0.0140	0.00520	0.0170	0.0340	19.485	2	2015 08 14	09:51
253.147	5.30515	137.3919	0.0002	0.000023	0.0122	0.0030	0.000187	0.0139	0.00520	0.0170	0.0338	21.083	2	2015 08 14	10:43
253.148	5.30532	137.3677	0.0007	0.000092	0.0137	0.0031	0.000207	0.0154	0.00520	0.0170	0.0355	20.886	2	2015 08 14	10:56
253.151	5.30551	137.4133	0.0007	0.000049	0.0134	0.0031	0.000192	0.0151	0.00520	0.0170	0.0350	19.970	2	2015 08 14	11:09
253.148	5.32107	137.7953	0.0001	0.000037	0.0120	0.0030	0.000189	0.0138	0.00520	0.0171	0.0337	20.047	2	2015 08 14	12:02
253.149	5.32120	137.8119	0.0006	0.000049	0.0136	0.0031	0.000192	0.0153	0.00520	0.0171	0.0351	21.152	2	2015 08 14	12:15
253.151	5.32134	137.7961	0.0004	0.000024	0.0100	0.0030	0.000187	0.0119	0.00520	0.0171	0.0322	20.816	2	2015 08 14	12:28

test: Ar_CO2_1508gh.dat (continued)

T (K)	p (MPa)	rho (kg.m−3)	sigma_T (K)	sigma_p (MPa)	sigma_rho (kg.m−3)	u(T) (K)	u(p) (MPa)	u(rho) (kg.m−3)	u(MW) (g/mol)	u[rho(x)] (kg.m−3)	U_c/% (k = 2)	m_sorb (mg)	p_trans	date	time
253.148	5.33625	138.2381	0.0002	0.000015	0.0110	0.0030	0.000186	0.0129	0.00520	0.0171	0.0329	22.590	2	2015 08 14	13:20
253.149	5.33631	138.2371	0.0005	0.000050	0.0133	0.0030	0.000192	0.0150	0.00520	0.0171	0.0348	22.257	2	2015 08 14	13:33
253.151	5.33646	138.2209	0.0007	0.000047	0.0107	0.0031	0.000192	0.0125	0.00520	0.0171	0.0327	22.971	2	2015 08 14	13:46
253.148	5.35104	138.6469	0.0001	0.000018	0.0115	0.0030	0.000187	0.0133	0.00520	0.0172	0.0332	23.121	2	2015 08 14	14:39
253.149	5.35108	138.6301	0.0005	0.000048	0.0128	0.0030	0.000192	0.0145	0.00520	0.0172	0.0343	22.145	2	2015 08 14	14:52
253.151	5.35121	138.6293	0.0004	0.000022	0.0098	0.0030	0.000187	0.0118	0.00520	0.0172	0.0320	21.404	2	2015 08 14	15:05

Table S3. Experimental (p, rho, T, x) data and detailed uncertainty information for isotherms measured on the (0.49896 argon + 0.50104 carbon dioxide) mixture (continued)

test: Ar_CO2_1508i.dat (234 hours under rough vacuum prior to test; starting p = 4.67 MPa; 30 min equilibration time; 3 replicates per pressure)
chiS [specific magnetic susceptibility] = -0.6035E-08 [m3/kg]

T (K)	p (MPa)	rho (kg.m-3)	sigma_T (K)	sigma_p (MPa)	sigma_rho (kg.m-3)	u(T) (K)	u(p) (MPa)	u(rho) (kg.m-3)	u(MW) (g/mol)	u[rho(x)] (kg.m-3)	U_c/% (k = 2)	m_sorb (mg)	p_trans	date	time
253.143	4.66508	117.2788	0.0005	0.000038	0.0021	0.0030	0.000181	0.0053	0.00520	0.0145	0.0286	-0.403	2	2015 08 24	11:42
253.146	4.66520	117.2870	0.0013	0.000054	0.0012	0.0033	0.000185	0.0049	0.00520	0.0145	0.0286	-0.376	2	2015 08 24	11:55
253.150	4.66534	117.2863	0.0009	0.000041	0.0008	0.0031	0.000182	0.0048	0.00520	0.0145	0.0284	-0.388	2	2015 08 24	12:08
253.148	4.79830	121.6884	0.0002	0.000017	0.0006	0.0030	0.000180	0.0049	0.00520	0.0151	0.0282	-0.063	2	2015 08 24	13:09
253.150	4.79836	121.6884	0.0009	0.000016	0.0005	0.0031	0.000180	0.0049	0.00520	0.0151	0.0283	-0.055	2	2015 08 24	13:22
253.152	4.79840	121.6867	0.0004	0.000016	0.0007	0.0030	0.000180	0.0050	0.00520	0.0151	0.0283	-0.032	2	2015 08 24	13:35
253.149	4.95246	126.8857	0.0002	0.000017	0.0007	0.0030	0.000182	0.0051	0.00520	0.0157	0.0282	0.019	2	2015 08 24	14:40
253.150	4.95247	126.8827	0.0003	0.000015	0.0003	0.0030	0.000182	0.0051	0.00520	0.0157	0.0282	0.011	2	2015 08 24	14:53
253.151	4.95246	126.8880	0.0002	0.000020	0.0003	0.0030	0.000182	0.0051	0.00520	0.0157	0.0282	0.019	2	2015 08 24	15:06
253.149	5.05082	130.2639	0.0001	0.000021	0.0004	0.0030	0.000183	0.0052	0.00520	0.0161	0.0282	0.033	2	2015 08 24	16:07
253.150	5.05083	130.2638	0.0002	0.000015	0.0007	0.0030	0.000183	0.0052	0.00520	0.0161	0.0282	0.027	2	2015 08 24	16:23
253.151	5.05083	130.2650	0.0003	0.000019	0.0004	0.0030	0.000183	0.0052	0.00520	0.0161	0.0282	0.036	2	2015 08 24	16:36
253.149	5.07450	131.0761	0.0001	0.000018	0.0007	0.0030	0.000183	0.0052	0.00520	0.0162	0.0282	0.084	2	2015 08 24	17:29
253.150	5.07452	131.0772	0.0003	0.000020	0.0004	0.0030	0.000184	0.0052	0.00520	0.0162	0.0282	0.074	2	2015 08 24	17:42
253.151	5.07452	131.0758	0.0002	0.000025	0.0009	0.0030	0.000184	0.0053	0.00520	0.0162	0.0283	0.076	2	2015 08 24	17:55
253.149	5.09571	131.7358	0.0001	0.000013	0.0052	0.0030	0.000183	0.0077	0.00520	0.0163	0.0295	0.466	2	2015 08 24	18:47
253.150	5.09573	131.7495	0.0004	0.000013	0.0020	0.0030	0.000183	0.0056	0.00520	0.0163	0.0284	0.466	2	2015 08 24	19:00
253.151	5.09573	131.7304	0.0002	0.000019	0.0003	0.0030	0.000184	0.0052	0.00520	0.0163	0.0282	0.474	2	2015 08 24	19:13
253.148	5.11386	132.2225	0.0002	0.000012	0.0154	0.0030	0.000183	0.0170	0.00520	0.0164	0.0373	1.037	2	2015 08 24	20:06
253.149	5.11391	132.2080	0.0004	0.000020	0.0115	0.0030	0.000184	0.0132	0.00520	0.0164	0.0337	1.090	2	2015 08 24	20:19
253.150	5.11397	132.2269	0.0004	0.000022	0.0075	0.0030	0.000184	0.0096	0.00520	0.0164	0.0307	1.154	2	2015 08 24	20:32
253.148	5.13086	132.6587	0.0002	0.000016	0.0177	0.0030	0.000184	0.0192	0.00520	0.0164	0.0396	1.544	2	2015 08 24	21:25
253.149	5.13093	132.6426	0.0004	0.000040	0.0156	0.0030	0.000188	0.0172	0.00520	0.0164	0.0375	1.571	2	2015 08 24	21:38
253.150	5.13105	132.6481	0.0004	0.000026	0.0115	0.0030	0.000185	0.0133	0.00520	0.0164	0.0337	1.613	2	2015 08 24	21:51
253.148	5.14701	133.0859	0.0002	0.000018	0.0179	0.0030	0.000184	0.0194	0.00520	0.0165	0.0398	2.039	2	2015 08 24	22:43
253.148	5.14711	133.0642	0.0004	0.000040	0.0182	0.0030	0.000188	0.0197	0.00520	0.0165	0.0401	2.060	2	2015 08 24	22:56
253.150	5.14725	133.0488	0.0006	0.000037	0.0144	0.0031	0.000187	0.0160	0.00520	0.0165	0.0362	2.080	2	2015 08 24	23:09
253.147	5.16250	133.5032	0.0002	0.000020	0.0162	0.0030	0.000185	0.0177	0.00520	0.0165	0.0379	2.492	2	2015 08 25	00:02
253.148	5.16261	133.4592	0.0004	0.000073	0.0173	0.0030	0.000198	0.0188	0.00520	0.0165	0.0393	2.503	2	2015 08 25	00:15
253.150	5.16280	133.4566	0.0007	0.000063	0.0157	0.0031	0.000194	0.0172	0.00520	0.0165	0.0376	2.515	2	2015 08 25	00:28
253.147	5.17747	133.8633	0.0003	0.000027	0.0151	0.0030	0.000186	0.0167	0.00520	0.0166	0.0368	2.844	2	2015 08 25	01:20
253.148	5.17760	133.8583	0.0005	0.000050	0.0184	0.0030	0.000191	0.0199	0.00520	0.0166	0.0403	2.854	2	2015 08 25	01:33
253.151	5.17779	133.8421	0.0007	0.000045	0.0148	0.0031	0.000189	0.0164	0.00520	0.0166	0.0366	2.856	2	2015 08 25	01:46
253.148	5.19200	134.2477	0.0001	0.000022	0.0140	0.0030	0.000185	0.0156	0.00520	0.0166	0.0357	3.068	2	2015 08 25	02:39
253.149	5.19210	134.2310	0.0005	0.000039	0.0166	0.0030	0.000188	0.0182	0.00520	0.0166	0.0383	3.071	2	2015 08 25	02:52
253.151	5.19227	134.2249	0.0007	0.000052	0.0141	0.0031	0.000191	0.0157	0.00520	0.0166	0.0359	3.070	2	2015 08 25	03:05
253.148	5.20603	134.6109	0.0002	0.000018	0.0130	0.0030	0.000185	0.0147	0.00520	0.0167	0.0348	3.246	2	2015 08 25	03:57
253.148	5.20613	134.5986	0.0004	0.000045	0.0152	0.0030	0.000190	0.0168	0.00520	0.0167	0.0369	3.238	2	2015 08 25	04:10
253.150	5.20629	134.5926	0.0007	0.000036	0.0141	0.0031	0.000188	0.0157	0.00520	0.0167	0.0358	3.230	2	2015 08 25	04:23
253.148	5.21965	134.9800	0.0003	0.000013	0.0117	0.0030	0.000185	0.0134	0.00520	0.0167	0.0336	3.369	2	2015 08 25	05:16
253.149	5.21973	134.9664	0.0005	0.000046	0.0140	0.0030	0.000190	0.0156	0.00520	0.0167	0.0357	3.371	2	2015 08 25	05:29
253.151	5.21987	134.9517	0.0005	0.000030	0.0133	0.0030	0.000187	0.0150	0.00520	0.0167	0.0351	3.364	2	2015 08 25	05:42
253.148	5.23269	135.3193	0.0003	0.000014	0.0113	0.0030	0.000185	0.0131	0.00520	0.0168	0.0333	3.452	2	2015 08 25	06:34
253.149	5.23279	135.3014	0.0005	0.000051	0.0140	0.0030	0.000191	0.0157	0.00520	0.0168	0.0357	3.448	2	2015 08 25	06:47
253.151	5.23296	135.2808	0.0007	0.000039	0.0130	0.0031	0.000189	0.0147	0.00520	0.0168	0.0348	3.441	2	2015 08 25	07:00
253.148	5.24525	135.6583	0.0001	0.000021	0.0118	0.0030	0.000186	0.0136	0.00520	0.0168	0.0337	3.523	2	2015 08 25	07:53
253.149	5.24537	135.6289	0.0006	0.000040	0.0140	0.0031	0.000189	0.0157	0.00520	0.0168	0.0357	3.528	2	2015 08 25	08:06
253.151	5.24551	135.6306	0.0005	0.000029	0.0108	0.0030	0.000187	0.0126	0.00520	0.0168	0.0329	3.539	2	2015 08 25	08:19

T	p	rho	sigma_T	sigma_p	sigma_rho	u(T)	u(p)	u(rho)	u(MW)	u[rho(x)]	U_c/%	m_sorb	p_trans	date	time
(K)	(MPa)	(kg.m−3)	(K)	(MPa)	(kg.m−3)	(K)	(MPa)	(kg.m−3)	(g/mol)	(kg.m−3)	(k = 2)	(mg)			
# test: Ar_CO2_1508i.dat (continued)															
253.148	5.25732	135.9654	0.0001	0.000019	0.0108	0.0030	0.000186	0.0127	0.00520	0.0168	0.0329	12.844	2	2015 08 25	09:11
253.149	5.25743	135.9621	0.0005	0.000042	0.0133	0.0030	0.000189	0.0149	0.00520	0.0168	0.0349	12.414	2	2015 08 25	09:24
253.151	5.25757	135.9560	0.0005	0.000030	0.0109	0.0030	0.000187	0.0128	0.00520	0.0168	0.0330	12.365	2	2015 08 25	09:37
253.148	5.26898	136.2830	0.0003	0.000010	0.0103	0.0030	0.000185	0.0122	0.00520	0.0169	0.0324	13.513	2	2015 08 25	10:30
253.148	5.26908	136.2712	0.0005	0.000028	0.0132	0.0030	0.000187	0.0149	0.00520	0.0169	0.0348	13.822	2	2015 08 25	10:43
253.150	5.26925	136.2647	0.0007	0.000057	0.0111	0.0031	0.000194	0.0130	0.00520	0.0169	0.0332	14.954	2	2015 08 25	10:56
253.148	5.28004	136.5799	0.0002	0.000019	0.0095	0.0030	0.000186	0.0115	0.00520	0.0169	0.0319	13.647	2	2015 08 25	11:49
253.149	5.28015	136.5660	0.0005	0.000048	0.0128	0.0030	0.000191	0.0145	0.00520	0.0169	0.0345	14.826	2	2015 08 25	12:02
253.151	5.28024	136.5574	0.0005	0.000028	0.0108	0.0030	0.000187	0.0126	0.00520	0.0169	0.0328	14.565	2	2015 08 25	12:15
253.148	5.29038	136.8496	0.0002	0.000023	0.0096	0.0030	0.000187	0.0116	0.00520	0.0170	0.0319	16.543	2	2015 08 25	13:07
253.148	5.29046	136.8425	0.0005	0.000031	0.0120	0.0030	0.000188	0.0138	0.00520	0.0169	0.0338	16.987	2	2015 08 25	13:20
253.150	5.29059	136.8446	0.0006	0.000041	0.0115	0.0031	0.000190	0.0133	0.00520	0.0169	0.0334	15.768	2	2015 08 25	13:33
253.147	5.30014	137.1362	0.0001	0.000028	0.0118	0.0030	0.000187	0.0135	0.00520	0.0170	0.0335	16.976	2	2015 08 25	14:26
253.148	5.30030	137.1340	0.0006	0.000067	0.0125	0.0031	0.000197	0.0143	0.00520	0.0170	0.0343	16.265	2	2015 08 25	14:39
253.151	5.30047	137.1142	0.0007	0.000040	0.0113	0.0031	0.000190	0.0131	0.00520	0.0170	0.0333	16.163	2	2015 08 25	14:52
253.148	5.30962	137.3712	0.0001	0.000013	0.0091	0.0030	0.000186	0.0111	0.00520	0.0170	0.0315	17.698	2	2015 08 25	15:44
253.149	5.30971	137.3777	0.0005	0.000044	0.0107	0.0030	0.000191	0.0126	0.00520	0.0170	0.0328	18.938	2	2015 08 25	15:57
253.151	5.30984	137.3489	0.0004	0.000024	0.0088	0.0030	0.000187	0.0109	0.00520	0.0170	0.0314	17.576	2	2015 08 25	16:10
253.148	5.31860	137.6155	0.0001	0.000026	0.0101	0.0030	0.000187	0.0120	0.00520	0.0170	0.0322	19.293	2	2015 08 25	17:03
253.149	5.31869	137.5689	0.0004	0.000024	0.0104	0.0030	0.000187	0.0123	0.00520	0.0170	0.0325	19.472	2	2015 08 25	17:16
253.151	5.31877	137.5799	0.0004	0.000023	0.0092	0.0030	0.000187	0.0112	0.00520	0.0170	0.0316	19.504	2	2015 08 25	17:29
253.149	5.32729	137.8416	0.0001	0.000029	0.0086	0.0030	0.000188	0.0107	0.00520	0.0171	0.0312	19.571	2	2015 08 25	18:21
253.149	5.32738	137.8684	0.0004	0.000036	0.0097	0.0030	0.000189	0.0117	0.00520	0.0171	0.0320	19.758	2	2015 08 25	18:34
253.151	5.32747	137.8122	0.0003	0.000019	0.0083	0.0030	0.000187	0.0104	0.00520	0.0171	0.0310	18.684	2	2015 08 25	18:47
253.149	5.33585	138.0706	0.0001	0.000020	0.0080	0.0030	0.000187	0.0102	0.00520	0.0171	0.0308	21.249	2	2015 08 25	19:40
253.149	5.33594	138.0623	0.0005	0.000037	0.0094	0.0030	0.000190	0.0114	0.00520	0.0171	0.0318	20.664	2	2015 08 25	19:53
253.151	5.33605	138.0550	0.0004	0.000032	0.0095	0.0030	0.000188	0.0114	0.00520	0.0171	0.0318	21.182	2	2015 08 25	20:06
253.149	5.34416	138.2883	0.0001	0.000023	0.0087	0.0030	0.000187	0.0108	0.00520	0.0171	0.0313	21.521	2	2015 08 25	20:58
253.150	5.34423	138.2841	0.0004	0.000041	0.0104	0.0030	0.000190	0.0123	0.00520	0.0171	0.0325	21.561	2	2015 08 25	21:11
253.151	5.34434	138.2791	0.0005	0.000030	0.0089	0.0030	0.000188	0.0109	0.00520	0.0171	0.0314	21.850	2	2015 08 25	21:24
253.149	5.35207	138.5075	0.0001	0.000022	0.0080	0.0030	0.000187	0.0101	0.00520	0.0172	0.0308	20.682	2	2015 08 25	22:17
253.150	5.35216	138.4954	0.0005	0.000054	0.0089	0.0030	0.000194	0.0109	0.00520	0.0172	0.0315	21.142	2	2015 08 25	22:30
253.151	5.35228	138.4904	0.0005	0.000021	0.0088	0.0030	0.000187	0.0108	0.00520	0.0172	0.0313	22.285	2	2015 08 25	22:43
253.149	5.35964	138.7046	0.0001	0.000017	0.0072	0.0030	0.000187	0.0095	0.00520	0.0172	0.0303	22.246	2	2015 08 25	23:35
253.149	5.35970	138.7041	0.0004	0.000034	0.0096	0.0030	0.000189	0.0116	0.00520	0.0172	0.0319	22.459	2	2015 08 25	23:48
253.151	5.35979	138.6919	0.0004	0.000024	0.0085	0.0030	0.000188	0.0106	0.00520	0.0172	0.0311	22.341	2	2015 08 26	00:01
253.150	5.36027	138.5064	0.0004	0.000039	0.0064	0.0030	0.000190	0.0088	0.00520	0.0172	0.0300	32.446	2	2015 08 27	16:17
253.151	5.36034	138.4837	0.0003	0.000025	0.0047	0.0030	0.000188	0.0074	0.00520	0.0172	0.0292	30.720	2	2015 08 27	16:30
253.152	5.36036	138.4842	0.0001	0.000014	0.0025	0.0030	0.000187	0.0061	0.00520	0.0172	0.0285	31.319	2	2015 08 27	16:43

Table S3. Experimental (p, rho, T, x) data and detailed uncertainty information for isotherms measured on the (0.49896 argon + 0.50104 carbon dioxide) mixture (continued)

test: Ar_CO2_1508j.dat (brief purge prior to test, but 40 hour prior soak in 2-phase; starting p = 4.66 MPa; 30 min equilibration time; 3 replicates per pressure)
chiS [specific magnetic susceptibility] = -0.6035E-08 [m3/kg]

T (K)	p (MPa)	rho (kg.m-3)	sigma_T (K)	sigma_p (MPa)	sigma_rho (kg.m-3)	u(T) (K)	u(p) (MPa)	u(rho) (kg.m-3)	u(MW) (g/mol)	u[rho(x)] (kg.m-3)	U_c/% (k = 2)	m_sorb (mg)	p_trans	date	time
253.145	4.66382	117.2570	0.0008	0.000020	0.0018	0.0031	0.000179	0.0051	0.00520	0.0145	0.0285	0.006	2	2015 08 27	18:23
253.149	4.66386	117.2553	0.0013	0.000026	0.0011	0.0033	0.000179	0.0049	0.00520	0.0145	0.0284	0.024	2	2015 08 27	18:36
253.153	4.66391	117.2526	0.0004	0.000021	0.0004	0.0030	0.000179	0.0048	0.00520	0.0145	0.0283	0.017	2	2015 08 27	18:55
253.150	4.80063	121.7665	0.0004	0.000013	0.0012	0.0030	0.000180	0.0051	0.00520	0.0151	0.0283	0.014	2	2015 08 27	19:59
253.151	4.80063	121.7663	0.0005	0.000010	0.0005	0.0030	0.000179	0.0049	0.00520	0.0151	0.0282	0.014	2	2015 08 27	20:12
253.152	4.80060	121.7650	0.0002	0.000025	0.0004	0.0030	0.000181	0.0049	0.00520	0.0151	0.0283	0.013	2	2015 08 27	20:25
253.150	4.95251	126.8886	0.0004	0.000029	0.0005	0.0030	0.000183	0.0051	0.00520	0.0157	0.0283	0.015	2	2015 08 27	21:36
253.149	4.95242	126.8856	0.0002	0.000011	0.0007	0.0030	0.000181	0.0051	0.00520	0.0157	0.0282	0.012	2	2015 08 27	21:49
253.149	4.95241	126.8861	0.0002	0.000010	0.0002	0.0030	0.000181	0.0051	0.00520	0.0157	0.0282	0.014	2	2015 08 27	22:02
253.149	5.04802	130.1695	0.0002	0.000029	0.0010	0.0030	0.000184	0.0053	0.00520	0.0161	0.0283	0.033	2	2015 08 27	23:09
253.149	5.04801	130.1696	0.0005	0.000019	0.0006	0.0030	0.000183	0.0052	0.00520	0.0161	0.0282	0.034	2	2015 08 27	23:22
253.151	5.04804	130.1667	0.0006	0.000029	0.0006	0.0031	0.000184	0.0052	0.00520	0.0161	0.0283	0.045	2	2015 08 27	23:35
253.149	5.06331	130.6959	0.0002	0.000021	0.0006	0.0030	0.000184	0.0052	0.00520	0.0162	0.0282	0.061	2	2015 08 28	00:28
253.149	5.06333	130.6956	0.0004	0.000019	0.0003	0.0030	0.000183	0.0052	0.00520	0.0162	0.0282	0.059	2	2015 08 28	00:41
253.151	5.06340	130.6925	0.0005	0.000025	0.0004	0.0030	0.000184	0.0052	0.00520	0.0162	0.0282	0.062	2	2015 08 28	00:54
253.149	5.07776	131.1826	0.0002	0.000029	0.0016	0.0030	0.000185	0.0055	0.00520	0.0162	0.0283	0.128	2	2015 08 28	01:46
253.150	5.07777	131.1842	0.0004	0.000014	0.0004	0.0030	0.000183	0.0052	0.00520	0.0162	0.0282	0.125	2	2015 08 28	01:59
253.151	5.07780	131.1879	0.0004	0.000010	0.0006	0.0030	0.000183	0.0052	0.00520	0.0162	0.0282	0.124	2	2015 08 28	02:12
253.149	5.09094	131.6132	0.0002	0.000014	0.0027	0.0030	0.000183	0.0060	0.00520	0.0163	0.0285	0.392	2	2015 08 28	03:05
253.149	5.09093	131.6036	0.0004	0.000025	0.0008	0.0030	0.000184	0.0053	0.00520	0.0163	0.0283	0.404	2	2015 08 28	03:18
253.151	5.09100	131.6096	0.0006	0.000033	0.0012	0.0031	0.000186	0.0054	0.00520	0.0163	0.0283	0.396	2	2015 08 28	03:31
253.149	5.10270	131.9082	0.0003	0.000024	0.0091	0.0030	0.000184	0.0110	0.00520	0.0163	0.0318	1.023	2	2015 08 28	04:23
253.149	5.10269	131.8989	0.0003	0.000017	0.0051	0.0030	0.000184	0.0076	0.00520	0.0163	0.0294	1.058	2	2015 08 28	04:36
253.151	5.10274	131.8984	0.0005	0.000022	0.0015	0.0030	0.000184	0.0055	0.00520	0.0163	0.0283	1.077	2	2015 08 28	04:49
253.149	5.11327	132.2042	0.0003	0.000012	0.0124	0.0030	0.000183	0.0141	0.00520	0.0164	0.0345	1.488	2	2015 08 28	05:42
253.149	5.11331	132.1819	0.0003	0.000030	0.0116	0.0030	0.000185	0.0134	0.00520	0.0164	0.0338	1.530	2	2015 08 28	05:55
253.151	5.11342	132.1695	0.0005	0.000023	0.0088	0.0030	0.000184	0.0108	0.00520	0.0164	0.0316	1.569	2	2015 08 28	06:08
253.148	5.12330	132.4161	0.0002	0.000016	0.0109	0.0030	0.000184	0.0126	0.00520	0.0164	0.0331	1.943	2	2015 08 28	07:01
253.149	5.12338	132.3992	0.0005	0.000026	0.0127	0.0030	0.000185	0.0144	0.00520	0.0164	0.0347	1.976	2	2015 08 28	07:14
253.151	5.12352	132.3865	0.0007	0.000044	0.0101	0.0031	0.000188	0.0120	0.00520	0.0164	0.0326	2.005	2	2015 08 28	07:27
253.149	5.13293	132.6502	0.0003	0.000011	0.0099	0.0030	0.000184	0.0118	0.00520	0.0164	0.0324	2.326	2	2015 08 28	08:19
253.149	5.13302	132.6459	0.0006	0.000049	0.0122	0.0031	0.000190	0.0139	0.00520	0.0164	0.0344	2.360	2	2015 08 28	08:32
253.151	5.13314	132.6330	0.0006	0.000032	0.0105	0.0031	0.000186	0.0123	0.00520	0.0164	0.0329	2.387	2	2015 08 28	08:45
253.149	5.14224	132.8899	0.0001	0.000025	0.0093	0.0030	0.000185	0.0113	0.00520	0.0165	0.0319	6.234	2	2015 08 28	09:38
253.149	5.14234	132.8711	0.0005	0.000057	0.0118	0.0030	0.000192	0.0135	0.00520	0.0165	0.0340	2.663	2	2015 08 28	09:51
253.151	5.14249	132.8729	0.0007	0.000051	0.0109	0.0031	0.000190	0.0127	0.00520	0.0165	0.0332	2.694	2	2015 08 28	10:04
253.148	5.15114	133.1135	0.0002	0.000025	0.0106	0.0030	0.000185	0.0124	0.00520	0.0165	0.0329	11.495	2	2015 08 28	10:56
253.149	5.15126	133.1046	0.0005	0.000058	0.0114	0.0030	0.000192	0.0132	0.00520	0.0165	0.0337	11.726	2	2015 08 28	11:09
253.151	5.15139	133.0903	0.0006	0.000029	0.0107	0.0031	0.000186	0.0125	0.00520	0.0165	0.0330	12.239	2	2015 08 28	11:22
253.148	5.15955	133.3380	0.0001	0.000019	0.0101	0.0030	0.000184	0.0119	0.00520	0.0165	0.0325	13.093	2	2015 08 28	12:15
253.149	5.15965	133.3199	0.0004	0.000044	0.0115	0.0030	0.000189	0.0132	0.00520	0.0165	0.0336	13.680	2	2015 08 28	12:28
253.151	5.15978	133.3134	0.0005	0.000026	0.0106	0.0030	0.000185	0.0124	0.00520	0.0165	0.0329	13.858	2	2015 08 28	12:41
253.149	5.16750	133.5435	0.0001	0.000017	0.0088	0.0030	0.000184	0.0108	0.00520	0.0165	0.0315	15.861	2	2015 08 28	13:33
253.149	5.16759	133.5486	0.0003	0.000030	0.0106	0.0030	0.000186	0.0124	0.00520	0.0165	0.0328	14.460	2	2015 08 28	13:46
253.151	5.16772	133.5039	0.0005	0.000046	0.0096	0.0030	0.000189	0.0115	0.00520	0.0165	0.0322	14.919	2	2015 08 28	13:59
253.149	5.17515	133.7567	0.0003	0.000019	0.0080	0.0030	0.000185	0.0101	0.00520	0.0166	0.0310	15.995	2	2015 08 28	14:52
253.149	5.17522	133.7470	0.0004	0.000053	0.0106	0.0030	0.000191	0.0124	0.00520	0.0166	0.0329	15.902	2	2015 08 28	15:05
253.151	5.17532	133.7017	0.0005	0.000026	0.0102	0.0030	0.000186	0.0121	0.00520	0.0166	0.0326	17.413	2	2015 08 28	15:18

T	p	rho	sigma_T	sigma_p	sigma_rho	u(T)	u(p)	u(rho)	u(MW)	u[rho(x)]	U_c/%	m_sorb	p_trans	date	time
(K)	(MPa)	(kg.m−3)	(K)	(MPa)	(kg.m−3)	(K)	(MPa)	(kg.m−3)	(g/mol)	(kg.m−3)	(k = 2)	(mg)			
# test: Ar_CO2_1508j.dat (continued)															
253.148	5.18226	133.9005	0.0003	0.000019	0.0073	0.0030	0.000185	0.0095	0.00520	0.0166	0.0306	17.602	2	2015 08 28	16:10
253.148	5.18234	133.9012	0.0003	0.000043	0.0098	0.0030	0.000189	0.0117	0.00520	0.0166	0.0323	17.605	2	2015 08 28	16:23
253.151	5.18250	133.8840	0.0008	0.000044	0.0114	0.0031	0.000189	0.0132	0.00520	0.0166	0.0335	18.274	2	2015 08 28	16:36
253.148	5.18910	134.0718	0.0002	0.000028	0.0078	0.0030	0.000186	0.0099	0.00520	0.0166	0.0308	18.338	2	2015 08 28	17:29
253.149	5.18919	134.0668	0.0005	0.000040	0.0103	0.0030	0.000188	0.0121	0.00520	0.0166	0.0326	19.161	2	2015 08 28	17:42
253.151	5.18930	134.0492	0.0005	0.000022	0.0086	0.0030	0.000185	0.0106	0.00520	0.0166	0.0314	19.231	2	2015 08 28	17:55
253.149	5.19568	134.2396	0.0002	0.000012	0.0075	0.0030	0.000184	0.0097	0.00520	0.0166	0.0307	19.341	2	2015 08 28	18:47
253.149	5.19575	134.2369	0.0003	0.000040	0.0088	0.0030	0.000188	0.0107	0.00520	0.0166	0.0315	19.255	2	2015 08 28	19:00
253.150	5.19582	134.2472	0.0003	0.000014	0.0080	0.0030	0.000184	0.0101	0.00520	0.0166	0.0310	19.989	2	2015 08 28	19:13
253.148	5.20191	134.3997	0.0002	0.000014	0.0067	0.0030	0.000185	0.0089	0.00520	0.0166	0.0302	20.338	2	2015 08 28	20:06
253.149	5.20197	134.3936	0.0004	0.000031	0.0082	0.0030	0.000187	0.0102	0.00520	0.0166	0.0311	20.078	2	2015 08 28	20:19
253.150	5.20207	134.4070	0.0005	0.000026	0.0078	0.0030	0.000186	0.0099	0.00520	0.0166	0.0308	20.691	2	2015 08 28	20:32
253.149	5.20799	134.5774	0.0000	0.000018	0.0065	0.0030	0.000185	0.0088	0.00520	0.0167	0.0301	21.614	2	2015 08 28	21:25
253.150	5.20808	134.5413	0.0004	0.000043	0.0078	0.0030	0.000189	0.0099	0.00520	0.0167	0.0309	22.241	2	2015 08 28	21:38
253.151	5.20816	134.5314	0.0003	0.000022	0.0066	0.0030	0.000185	0.0089	0.00520	0.0167	0.0301	21.774	2	2015 08 28	21:51
253.148	5.21376	134.6914	0.0002	0.000020	0.0068	0.0030	0.000185	0.0091	0.00520	0.0167	0.0303	22.122	2	2015 08 28	22:43
253.149	5.21386	134.6924	0.0004	0.000037	0.0086	0.0030	0.000188	0.0106	0.00520	0.0167	0.0314	22.620	2	2015 08 28	22:56
253.150	5.21394	134.6846	0.0003	0.000013	0.0086	0.0030	0.000185	0.0106	0.00520	0.0167	0.0313	23.200	2	2015 08 28	23:09
253.148	5.21943	134.8393	0.0003	0.000012	0.0050	0.0030	0.000185	0.0076	0.00520	0.0167	0.0293	23.063	2	2015 08 29	00:02
253.149	5.21951	134.8383	0.0004	0.000040	0.0080	0.0030	0.000189	0.0101	0.00520	0.0167	0.0310	23.933	2	2015 08 29	00:15
253.150	5.21963	134.8422	0.0005	0.000033	0.0094	0.0030	0.000187	0.0114	0.00520	0.0167	0.0319	23.193	2	2015 08 29	00:28
253.149	5.22511	134.9980	0.0002	0.000012	0.0043	0.0030	0.000185	0.0071	0.00520	0.0167	0.0290	24.727	2	2015 08 29	01:20
253.149	5.22514	134.9904	0.0002	0.000027	0.0062	0.0030	0.000186	0.0086	0.00520	0.0167	0.0299	23.607	2	2015 08 29	01:33
253.150	5.22522	134.9681	0.0003	0.000017	0.0073	0.0030	0.000185	0.0095	0.00520	0.0167	0.0305	24.816	2	2015 08 29	01:46
253.149	5.23061	135.1433	0.0001	0.000015	0.0053	0.0030	0.000185	0.0078	0.00520	0.0167	0.0295	25.398	2	2015 08 29	02:39
253.149	5.23065	135.1129	0.0002	0.000029	0.0059	0.0030	0.000187	0.0083	0.00520	0.0167	0.0298	24.583	2	2015 08 29	02:52
253.150	5.23071	135.1105	0.0003	0.000020	0.0064	0.0030	0.000186	0.0087	0.00520	0.0167	0.0300	24.595	2	2015 08 29	03:05
253.150	5.23592	135.2566	0.0001	0.000015	0.0048	0.0030	0.000185	0.0075	0.00520	0.0168	0.0292	25.559	2	2015 08 29	03:57
253.149	5.23593	135.2590	0.0001	0.000017	0.0053	0.0030	0.000185	0.0079	0.00520	0.0168	0.0295	25.182	2	2015 08 29	04:10
253.150	5.23597	135.2627	0.0001	0.000018	0.0061	0.0030	0.000185	0.0085	0.00520	0.0168	0.0298	25.598	2	2015 08 29	04:23
253.150	5.24100	135.3959	0.0002	0.000020	0.0043	0.0030	0.000186	0.0071	0.00520	0.0168	0.0290	25.053	2	2015 08 29	05:16
253.149	5.24100	135.4000	0.0001	0.000019	0.0061	0.0030	0.000185	0.0085	0.00520	0.0168	0.0299	25.796	2	2015 08 29	05:29
253.150	5.24104	135.3839	0.0002	0.000014	0.0062	0.0030	0.000185	0.0086	0.00520	0.0168	0.0299	26.497	2	2015 08 29	05:42
253.150	5.24087	135.3382	0.0000	0.000016	0.0030	0.0030	0.000185	0.0063	0.00520	0.0168	0.0286	28.805	2	2015 08 29	11:17
253.150	5.24086	135.3279	0.0001	0.000012	0.0021	0.0030	0.000185	0.0058	0.00520	0.0168	0.0284	28.067	2	2015 08 29	11:30
253.150	5.24083	135.3321	0.0001	0.000019	0.0017	0.0030	0.000186	0.0057	0.00520	0.0168	0.0283	28.142	2	2015 08 29	11:43

Table S4. Experimental (p, rho, T, x) data and detailed uncertainty information for isotherms measured on the (0.26579 methane + 0.73421 propane) mixture

```
# component(s): methane                       mole frac:    0.265791
#              propane                                      0.734209
#
# molar mass:          36.639343
# nc =  2
# Uncertainty factors [all for standard (k = 1) errors]:
# state point:
#   standard uncertainty in T/K:                            0.0030
#   (p_trans = 1): zero offset in p/kPa                     1.000
#                  relative uncertainty in p/ppm:          26.0
#   (p_trans = 2): zero offset in p/kPa                     0.150
#                  relative uncertainty in p/ppm:          20.0
#   (p_trans = 3): zero offset in p/kPa                     0.030
#                  relative uncertainty in p/ppm:          20.0
#   hydrostatic head, uncertainty in L/V height (m):        0.050
# sinker volume
#   u(V_sinker)/ppm at Tref                                28.0
#   u(V_sinker)_temperature coeff [ppm/K]                   0.375
#   u(V_sinker)_pressure coeff [ppm/MPa]                    0.625
# density [except for V_sinker]
#   u_rho/[kg/m^3]                                          0.00100
# sample
#   sample purity, u(rho) [relative error]:                 0.000000
#   gravimetric prep [uncertainty in MW]                    0.001800
#   sorption of sample [uncertainty in MW]                  0.005200
```

T (K)	p (MPa)	rho (kg.m−3)	sigma_T (K)	sigma_p* (MPa)	sigma_rho* (kg.m−3)	u(T) (K)	u(p) (MPa)	u(rho) (kg.m−3)	u(MW) (g/mol)	u[rho(x)] (kg.m−3)	U_c/% (k = 2)	m_sorb (mg)	p_trans	date	time
# test: mp_1403n.dat* (pressure increasing due to leak + cycle valves manually)															
# chiS [specific magnetic susceptibility] = −0.1131E−07 [m3/kg]															
273.149	0.33852	5.7456	0.0011	0.000611	0.0198	0.0032	0.000612	0.0208	0.00550	0.0009	0.8209	−0.000	3	2014 03 18	13:12
273.151	0.34115	5.7925	0.0003	0.000610	0.0197	0.0030	0.000610	0.0207	0.00550	0.0009	0.8113	0.002	3	2014 03 18	13:28
273.149	0.34328	5.8303	0.0009	0.000608	0.0198	0.0031	0.000608	0.0208	0.00550	0.0009	0.8085	0.000	3	2014 03 18	13:41
273.146	0.34834	5.9219	0.0006	0.000607	0.0197	0.0031	0.000608	0.0207	0.00550	0.0009	0.7921	−0.000	3	2014 03 18	14:12
273.150	0.35144	5.9759	0.0005	0.000605	0.0196	0.0030	0.000606	0.0206	0.00550	0.0009	0.7822	−0.007	3	2014 03 18	14:31
273.150	0.35356	6.0147	0.0003	0.000603	0.0197	0.0030	0.000604	0.0207	0.00550	0.0009	0.7788	0.001	3	2014 03 18	14:44
273.147	0.41772	7.1825	0.0002	0.000536	0.0179	0.0030	0.000537	0.0189	0.00550	0.0011	0.5967	−0.000	3	2014 03 18	15:12
273.148	0.42076	7.2377	0.0003	0.000539	0.0178	0.0030	0.000540	0.0188	0.00550	0.0011	0.5894	0.002	3	2014 03 18	15:33
273.149	0.42264	7.2734	0.0003	0.000536	0.0176	0.0030	0.000537	0.0186	0.00550	0.0011	0.5818	0.002	3	2014 03 18	15:46
273.148	0.47763	8.2979	0.0004	0.000496	0.0168	0.0030	0.000497	0.0178	0.00550	0.0012	0.4854	0.002	3	2014 03 18	16:12
273.146	0.48070	8.3556	0.0001	0.000496	0.0171	0.0030	0.000497	0.0181	0.00550	0.0013	0.4907	0.004	3	2014 03 18	16:35
273.147	0.48243	8.3853	0.0003	0.000495	0.0167	0.0030	0.000496	0.0177	0.00550	0.0013	0.4797	0.002	3	2014 03 18	16:48
273.147	0.55039	9.6863	0.0005	0.000434	0.0152	0.0030	0.000435	0.0162	0.00550	0.0015	0.3798	0.003	3	2014 03 18	17:37
273.146	0.55191	9.7153	0.0001	0.000434	0.0150	0.0030	0.000435	0.0160	0.00550	0.0015	0.3750	0.003	3	2014 03 18	17:50
273.147	0.55343	9.7432	0.0005	0.000434	0.0151	0.0030	0.000435	0.0161	0.00550	0.0015	0.3758	0.003	3	2014 03 18	18:03
273.149	0.64055	11.4604	0.0001	0.000376	0.0140	0.0030	0.000377	0.0150	0.00550	0.0017	0.2953	0.011	3	2014 03 19	07:23
273.148	0.64186	11.4873	0.0003	0.000375	0.0138	0.0030	0.000376	0.0148	0.00550	0.0017	0.2915	0.010	3	2014 03 19	07:36
273.147	0.64317	11.5135	0.0002	0.000375	0.0136	0.0030	0.000376	0.0147	0.00550	0.0017	0.2889	0.010	3	2014 03 19	07:49
273.148	0.65617	11.7735	0.0003	0.000336	0.0121	0.0030	0.000338	0.0132	0.00550	0.0018	0.2543	0.016	3	2014 03 19	08:12
273.149	0.65859	11.8213	0.0002	0.000330	0.0120	0.0030	0.000332	0.0130	0.00550	0.0018	0.2504	0.020	3	2014 03 19	08:39
273.148	0.65975	11.8455	0.0003	0.000329	0.0120	0.0030	0.000331	0.0130	0.00550	0.0018	0.2493	0.022	3	2014 03 19	08:52
273.146	0.66151	11.8809	0.0000	0.000325	0.0117	0.0030	0.000327	0.0127	0.00550	0.0018	0.2432	0.027	3	2014 03 19	09:12
273.149	0.66469	11.9450	0.0001	0.000309	0.0100	0.0030	0.000311	0.0110	0.00550	0.0018	0.2151	0.047	3	2014 03 19	09:49
273.148	0.66589	11.9678	0.0004	0.000283	0.0066	0.0030	0.000285	0.0077	0.00550	0.0018	0.1641	0.064	3	2014 03 19	10:04

T (K)	p (MPa)	rho (kg.m-3)	sigma_T (K)	sigma_p (MPa)	sigma_rho (kg.m-3)	u(T) (K)	u(p) (MPa)	u(rho) (kg.m-3)	u(MW) (g/mol)	u[rho(x)] (kg.m-3)	U_c/% (k = 2)	m_sorb (mg)	p_trans	date	time
# test: mp_1403n.dat (continued)															
273.147	0.66804	11.9876	0.0005	0.000148	0.0092	0.0030	0.000151	0.0102	0.00550	0.0018	0.1801	0.239	3	2014 03 19	10:42
273.149	0.66852	11.9861	0.0005	0.000129	0.0084	0.0030	0.000133	0.0095	0.00550	0.0018	0.1670	0.320	3	2014 03 19	10:55
273.150	0.66894	11.9823	0.0002	0.000116	0.0057	0.0030	0.000121	0.0068	0.00550	0.0018	0.1240	0.384	3	2014 03 19	11:08
273.147	0.66997	11.9817	0.0001	0.000116	0.0066	0.0030	0.000121	0.0076	0.00550	0.0018	0.1374	0.514	3	2014 03 19	11:42
273.148	0.67044	11.9686	0.0005	0.000119	0.0099	0.0030	0.000124	0.0109	0.00550	0.0018	0.1896	0.986	3	2014 03 19	11:57
273.149	0.67135	11.9744	0.0003	0.000105	0.0023	0.0030	0.000110	0.0033	0.00550	0.0018	0.0734	0.741	3	2014 03 19	12:27
273.148	0.67836	11.9490	0.0004	0.000110	0.0082	0.0030	0.000115	0.0092	0.00550	0.0018	0.1614	11.025	3	2014 03 19	15:50
273.149	0.67873	11.9253	0.0003	0.000099	0.0045	0.0030	0.000104	0.0055	0.00550	0.0018	0.1041	11.453	3	2014 03 19	16:03
273.149	0.67907	11.9181	0.0004	0.000093	0.0059	0.0030	0.000099	0.0069	0.00550	0.0018	0.1239	12.887	3	2014 03 19	16:16

____

* Leaking valve resulted in continuously increasing pressure and resulted in large sigma_p and sigma_rho for this test, but time-symmetric weighing design compensated for this effect. Thus, the combined uncertainty is overstated; see McLinden, M. O., J. Chem. Eng. Data 2011, 56, 606-613.

Table S4. Experimental (p, rho, T, x) data and detailed uncertainty information for isotherms measured on the (0.26579 methane + 0.73421 propane) mixture (continued)

test: mp_1403pq.dat (equilibrium points, p adjusted manually; held at p = 660 kPa overnight)
chiS [specific magnetic susceptibility] = −0.1131E−07 [m3/kg]

T (K)	p (MPa)	rho (kg.m−3)	sigma_T (K)	sigma_p (MPa)	sigma_rho (kg.m−3)	u(T) (K)	u(p) (MPa)	u(rho) (kg.m−3)	u(MW) (g/mol)	u[rho(x)] (kg.m−3)	U_c/% (k = 2)	m_sorb (mg)	p_trans	date	time
273.146	0.50426	8.8019	0.0007	0.000001	0.0019	0.0031	0.000032	0.0029	0.00550	0.0013	0.0738	0.002	3	2014 03 20	10:58
273.148	0.50426	8.8020	0.0004	0.000002	0.0004	0.0030	0.000032	0.0015	0.00550	0.0013	0.0474	0.003	3	2014 03 20	11:11
273.149	0.50426	8.8034	0.0002	0.000001	0.0008	0.0030	0.000032	0.0018	0.00550	0.0013	0.0535	0.004	3	2014 03 20	11:24
273.147	0.50426	8.8014	0.0008	0.000001	0.0004	0.0031	0.000032	0.0015	0.00550	0.0013	0.0474	0.003	3	2014 03 20	11:37
273.149	0.55025	9.6842	0.0002	0.000002	0.0007	0.0030	0.000032	0.0017	0.00550	0.0015	0.0487	0.003	3	2014 03 20	12:36
273.147	0.55025	9.6841	0.0007	0.000002	0.0007	0.0031	0.000032	0.0017	0.00550	0.0015	0.0483	0.005	3	2014 03 20	12:49
273.145	0.55024	9.6838	0.0006	0.000002	0.0009	0.0031	0.000032	0.0019	0.00550	0.0015	0.0513	0.004	3	2014 03 20	13:02
273.144	0.55024	9.6842	0.0002	0.000001	0.0004	0.0030	0.000032	0.0015	0.00550	0.0015	0.0455	0.003	3	2014 03 20	13:15
273.148	0.60232	10.7002	0.0006	0.000003	0.0007	0.0031	0.000032	0.0018	0.00550	0.0016	0.0465	0.003	3	2014 03 20	13:59
273.146	0.60231	10.7021	0.0008	0.000003	0.0005	0.0031	0.000032	0.0016	0.00550	0.0016	0.0444	0.003	3	2014 03 20	14:12
273.144	0.60230	10.7007	0.0002	0.000001	0.0007	0.0030	0.000032	0.0017	0.00550	0.0016	0.0460	0.005	3	2014 03 20	14:25
273.145	0.60231	10.7015	0.0005	0.000001	0.0004	0.0030	0.000032	0.0015	0.00550	0.0016	0.0434	0.003	3	2014 03 20	14:38
273.147	0.62034	11.0581	0.0003	0.000003	0.0005	0.0030	0.000033	0.0016	0.00550	0.0017	0.0438	0.008	3	2014 03 20	15:19
273.145	0.62033	11.0606	0.0004	0.000002	0.0005	0.0030	0.000033	0.0016	0.00550	0.0017	0.0431	0.006	3	2014 03 20	15:32
273.145	0.62033	11.0602	0.0002	0.000002	0.0004	0.0030	0.000033	0.0015	0.00550	0.0017	0.0428	0.007	3	2014 03 20	15:45
273.146	0.62033	11.0577	0.0004	0.000002	0.0004	0.0030	0.000033	0.0015	0.00550	0.0017	0.0420	0.008	3	2014 03 20	15:58
273.146	0.64091	11.4711	0.0005	0.000002	0.0010	0.0030	0.000033	0.0021	0.00550	0.0017	0.0483	0.009	3	2014 03 20	16:40
273.145	0.64091	11.4697	0.0001	0.000001	0.0007	0.0030	0.000033	0.0018	0.00550	0.0017	0.0452	0.009	3	2014 03 20	16:53
273.145	0.64091	11.4688	0.0003	0.000001	0.0008	0.0030	0.000033	0.0019	0.00550	0.0017	0.0462	0.010	3	2014 03 20	17:06
273.147	0.64091	11.4705	0.0004	0.000002	0.0006	0.0030	0.000033	0.0017	0.00550	0.0017	0.0437	0.011	3	2014 03 20	17:19
273.146	0.65078	11.6662	0.0005	0.000002	0.0006	0.0030	0.000033	0.0017	0.00550	0.0018	0.0430	0.013	3	2014 03 20	17:58
273.145	0.65078	11.6680	0.0001	0.000002	0.0004	0.0030	0.000033	0.0016	0.00550	0.0018	0.0419	0.013	3	2014 03 20	18:11
273.145	0.65078	11.6695	0.0003	0.000002	0.0004	0.0030	0.000033	0.0015	0.00550	0.0018	0.0417	0.012	3	2014 03 20	18:24
273.147	0.65078	11.6682	0.0004	0.000001	0.0005	0.0030	0.000033	0.0016	0.00550	0.0018	0.0426	0.014	3	2014 03 20	18:37
273.150	0.66115	11.8751	0.0003	0.000001	0.0004	0.0030	0.000033	0.0015	0.00550	0.0018	0.0413	0.025	3	2014 03 20	21:12
273.150	0.66115	11.8761	0.0004	0.000001	0.0007	0.0030	0.000033	0.0018	0.00550	0.0018	0.0441	0.026	3	2014 03 20	21:25
273.148	0.66114	11.8758	0.0006	0.000001	0.0005	0.0031	0.000033	0.0016	0.00550	0.0018	0.0425	0.026	3	2014 03 20	21:38
273.146	0.66114	11.8768	0.0002	0.000001	0.0013	0.0030	0.000033	0.0023	0.00550	0.0018	0.0507	0.025	3	2014 03 20	21:51
273.148	0.66114	11.8766	0.0005	0.000002	0.0006	0.0030	0.000033	0.0017	0.00550	0.0018	0.0431	0.024	3	2014 03 20	22:12
273.149	0.66114	11.8764	0.0004	0.000002	0.0005	0.0030	0.000033	0.0016	0.00550	0.0018	0.0418	0.025	3	2014 03 20	22:42
273.147	0.66114	11.8752	0.0004	0.000001	0.0006	0.0030	0.000033	0.0017	0.00550	0.0018	0.0434	0.024	3	2014 03 20	22:55
273.146	0.66114	11.8757	0.0001	0.000001	0.0005	0.0030	0.000033	0.0016	0.00550	0.0018	0.0425	0.025	3	2014 03 20	23:08
273.149	0.66115	11.8760	0.0003	0.000001	0.0009	0.0030	0.000033	0.0020	0.00550	0.0018	0.0467	0.025	3	2014 03 20	23:42
273.148	0.66114	11.8748	0.0003	0.000002	0.0004	0.0030	0.000033	0.0015	0.00550	0.0018	0.0411	0.027	3	2014 03 21	00:07
273.147	0.66114	11.8741	0.0002	0.000001	0.0009	0.0030	0.000033	0.0020	0.00550	0.0018	0.0466	0.026	3	2014 03 21	00:20
273.147	0.66114	11.8750	0.0002	0.000002	0.0006	0.0030	0.000033	0.0017	0.00550	0.0018	0.0430	0.025	3	2014 03 21	00:33
273.147	0.66114	11.8740	0.0003	0.000001	0.0004	0.0030	0.000033	0.0016	0.00550	0.0018	0.0416	0.026	3	2014 03 21	01:32
273.147	0.66114	11.8752	0.0001	0.000001	0.0007	0.0030	0.000033	0.0018	0.00550	0.0018	0.0437	0.028	3	2014 03 21	01:45
273.147	0.66114	11.8743	0.0004	0.000001	0.0005	0.0030	0.000033	0.0016	0.00550	0.0018	0.0424	0.026	3	2014 03 21	01:58
273.148	0.66114	11.8747	0.0004	0.000002	0.0006	0.0030	0.000033	0.0017	0.00550	0.0018	0.0428	0.024	3	2014 03 21	02:42
273.146	0.66114	11.8751	0.0001	0.000001	0.0008	0.0030	0.000033	0.0019	0.00550	0.0018	0.0455	0.025	3	2014 03 21	03:05
273.147	0.66114	11.8744	0.0005	0.000002	0.0004	0.0030	0.000033	0.0016	0.00550	0.0018	0.0416	0.026	3	2014 03 21	03:18
273.149	0.66114	11.8755	0.0004	0.000001	0.0006	0.0030	0.000033	0.0017	0.00550	0.0018	0.0435	0.027	3	2014 03 21	03:31
273.146	0.66570	11.9639	0.0005	0.000001	0.0003	0.0030	0.000033	0.0014	0.00550	0.0018	0.0402	0.070	3	2014 03 21	08:12
273.149	0.66571	11.9628	0.0006	0.000002	0.0005	0.0031	0.000033	0.0016	0.00550	0.0018	0.0420	0.070	3	2014 03 21	08:25
273.150	0.66571	11.9641	0.0002	0.000001	0.0005	0.0030	0.000033	0.0016	0.00550	0.0018	0.0418	0.069	3	2014 03 21	08:38
273.149	0.66570	11.9638	0.0005	0.000003	0.0001	0.0030	0.000033	0.0014	0.00550	0.0018	0.0395	0.068	3	2014 03 21	08:51

T	p	rho	sigma_T	sigma_p	sigma_rho	u(T)	u(p)	u(rho)	u(MW)	u[rho(x)]	U_c/%	m_sorb	p_trans	date	time
(K)	(MPa)	(kg.m-3)	(K)	(MPa)	(kg.m-3)	(K)	(MPa)	(kg.m-3)	(g/mol)	(kg.m-3)	(k = 2)	(mg)			
# test: mp_1403pq.dat (continued)															
273.146	0.66741	11.9840	0.0004	0.000005	0.0016	0.0030	0.000033	0.0027	0.00550	0.0018	0.0551	0.190	3	2014 03 21	09:25
273.148	0.66741	11.9834	0.0007	0.000003	0.0008	0.0031	0.000033	0.0019	0.00550	0.0018	0.0454	0.199	3	2014 03 21	09:38
273.150	0.66742	11.9820	0.0003	0.000001	0.0006	0.0030	0.000033	0.0017	0.00550	0.0018	0.0428	0.200	3	2014 03 21	09:51
273.150	0.66741	11.9827	0.0004	0.000005	0.0002	0.0030	0.000033	0.0014	0.00550	0.0018	0.0401	0.195	3	2014 03 21	10:04
273.147	0.66984	11.9762	0.0002	0.000006	0.0099	0.0030	0.000033	0.0110	0.00550	0.0018	0.1858	0.490	3	2014 03 21	10:38
273.148	0.66987	11.9746	0.0004	0.000004	0.0095	0.0030	0.000033	0.0105	0.00550	0.0018	0.1786	0.604	3	2014 03 21	10:52
273.149	0.66987	11.9655	0.0001	0.000005	0.0045	0.0030	0.000033	0.0055	0.00550	0.0018	0.0977	0.648	3	2014 03 21	11:05
273.148	0.66984	11.9537	0.0004	0.000006	0.0061	0.0030	0.000033	0.0071	0.00550	0.0018	0.1234	0.641	3	2014 03 21	11:18
273.148	0.67235	11.9342	0.0000	0.000001	0.0064	0.0030	0.000033	0.0074	0.00550	0.0018	0.1277	3.109	3	2014 03 21	12:36
273.148	0.67236	11.9382	0.0000	0.000001	0.0054	0.0030	0.000033	0.0064	0.00550	0.0018	0.1123	0.976	3	2014 03 21	12:49
273.148	0.67236	11.9390	0.0001	0.000001	0.0051	0.0030	0.000033	0.0061	0.00550	0.0018	0.1071	1.010	3	2014 03 21	13:02
273.148	0.67235	11.9266	0.0001	0.000002	0.0034	0.0030	0.000033	0.0044	0.00550	0.0018	0.0808	3.172	3	2014 03 21	13:15
273.148	0.67490	11.9142	0.0001	0.000001	0.0048	0.0030	0.000033	0.0058	0.00550	0.0018	0.1021	9.583	3	2014 03 21	14:26
273.148	0.67490	11.9273	0.0001	0.000001	0.0053	0.0030	0.000033	0.0063	0.00550	0.0018	0.1106	10.003	3	2014 03 21	14:39
273.148	0.67491	11.9044	0.0001	0.000002	0.0048	0.0030	0.000033	0.0058	0.00550	0.0018	0.1025	10.498	3	2014 03 21	14:52
273.148	0.67491	11.8847	0.0002	0.000001	0.0032	0.0030	0.000033	0.0043	0.00550	0.0018	0.0786	11.058	3	2014 03 21	15:05
273.147	0.67487	11.9146	0.0003	0.000004	0.0023	0.0030	0.000033	0.0033	0.00550	0.0018	0.0647	11.645	3	2014 03 21	15:36
273.146	0.67487	11.8971	0.0001	0.000001	0.0044	0.0030	0.000033	0.0054	0.00550	0.0018	0.0969	11.623	3	2014 03 21	15:54
273.146	0.67487	11.9011	0.0002	0.000003	0.0058	0.0030	0.000033	0.0068	0.00550	0.0018	0.1184	12.854	3	2014 03 21	16:07
273.147	0.67488	11.8774	0.0002	0.000001	0.0043	0.0030	0.000033	0.0054	0.00550	0.0018	0.0958	13.853	3	2014 03 21	16:20
273.145	0.67484	11.8900	0.0000	0.000001	0.0053	0.0030	0.000033	0.0063	0.00550	0.0018	0.1105	15.782	3	2014 03 21	17:11
273.145	0.67485	11.8598	0.0003	0.000005	0.0056	0.0030	0.000033	0.0067	0.00550	0.0018	0.1167	14.696	3	2014 03 21	17:24
273.146	0.67487	11.8505	0.0004	0.000006	0.0082	0.0030	0.000034	0.0092	0.00550	0.0018	0.1590	15.757	3	2014 03 21	17:37
273.147	0.67488	11.8244	0.0002	0.000001	0.0026	0.0030	0.000033	0.0036	0.00550	0.0018	0.0694	15.678	3	2014 03 21	17:50

Table S5. Experimental (p, rho, T, x) data and detailed uncertainty information for isotherms measured on the (0.50688 methane + 0.49312 propane) mixture

```
# component(s): methane                       mole frac:    0.506875
#               propane                                     0.493124
#
# molar mass:          29.876130
# nc =  2
# Uncertainty factors [all for standard (k = 1) errors]:
# state point:
#   standard uncertainty in T/K:                            0.0030
#   (p_trans = 1): zero offset in p/kPa                     1.000
#                  relative uncertainty in p/ppm:          26.0
#   (p_trans = 2): zero offset in p/kPa                     0.150
#                  relative uncertainty in p/ppm:          20.0
#   (p_trans = 3): zero offset in p/kPa                     0.030
#                  relative uncertainty in p/ppm:          20.0
#   hydrostatic head, uncertainty in L/V height (m):        0.050
# sinker volume
#   u(V_sinker)/ppm at Tref                                28.0
#   u(V_sinker)_temperature coeff [ppm/K]                   0.375
#   u(V_sinker)_pressure coeff [ppm/MPa]                    0.625
# density [except for V_sinker]
#   u_rho/[kg/m^3]                                          0.00100
# sample
#   sample purity, u(rho) [relative error]:                 0.000000
#   gravimetric prep [uncertainty in MW]                    0.000780
#   sorption of sample [uncertainty in MW]                  0.005200
```

T (K)	p (MPa)	rho (kg.m-3)	sigma_T (K)	sigma_p* (MPa)	sigma_rho* (kg.m-3)	u(T) (K)	u(p) (MPa)	u(rho) (kg.m-3)	u(MW) (g/mol)	u[rho(x)] (kg.m-3)	U_c/% (k = 2)	m_sorb (mg)	p_trans	date	time
# test: mp_1403s.dat* (pressure increasing due to leaking valve)															
# chiS [specific magnetic susceptibility] = -0.1172E-07 [m3/kg]															
273.147	0.38605	5.2645	0.0004	0.000478	0.0125	0.0030	0.000479	0.0135	0.00526	0.0009	0.5759	-0.001	3	2014 03 22	13:12
273.149	0.38991	5.3200	0.0007	0.000478	0.0122	0.0031	0.000479	0.0132	0.00526	0.0009	0.5613	-0.001	3	2014 03 22	13:42
273.149	0.39375	5.3752	0.0005	0.000474	0.0123	0.0030	0.000475	0.0133	0.00526	0.0009	0.5565	-0.000	3	2014 03 22	14:12
273.146	0.39758	5.4322	0.0002	0.000473	0.0123	0.0030	0.000474	0.0133	0.00526	0.0010	0.5495	-0.001	3	2014 03 22	14:42
273.148	0.51989	7.1907	0.0007	0.000426	0.0115	0.0031	0.000428	0.0125	0.00526	0.0013	0.3901	-0.001	3	2014 03 22	15:42
273.146	0.52173	7.2169	0.0001	0.000428	0.0119	0.0030	0.000429	0.0129	0.00526	0.0013	0.3993	-0.006	3	2014 03 22	15:58
273.149	0.52680	7.2897	0.0003	0.000426	0.0115	0.0030	0.000428	0.0125	0.00526	0.0013	0.3853	0.000	3	2014 03 22	16:42
273.148	0.66647	9.3584	0.0001	0.000361	0.0100	0.0030	0.000362	0.0110	0.00526	0.0016	0.2647	-0.003	3	2014 03 22	17:12
273.148	0.66943	9.4067	0.0001	0.000368	0.0105	0.0030	0.000369	0.0115	0.00526	0.0017	0.2739	0.000	3	2014 03 22	17:42
273.148	0.67241	9.4521	0.0001	0.000367	0.0104	0.0030	0.000368	0.0114	0.00526	0.0017	0.2709	0.002	3	2014 03 22	18:12
273.148	0.67539	9.4960	0.0002	0.000369	0.0104	0.0030	0.000370	0.0114	0.00526	0.0017	0.2695	0.002	3	2014 03 22	18:42
273.148	0.67837	9.5395	0.0002	0.000368	0.0102	0.0030	0.000369	0.0112	0.00526	0.0017	0.2659	0.000	3	2014 03 22	19:12
273.147	0.68134	9.5833	0.0001	0.000369	0.0103	0.0030	0.000370	0.0113	0.00526	0.0017	0.2663	0.002	3	2014 03 22	19:42
273.148	0.68431	9.6318	0.0003	0.000367	0.0103	0.0030	0.000368	0.0113	0.00526	0.0017	0.2648	0.002	3	2014 03 22	20:12
273.148	0.68728	9.6759	0.0003	0.000366	0.0104	0.0030	0.000368	0.0114	0.00526	0.0017	0.2649	0.002	3	2014 03 22	20:42
273.147	0.69023	9.7196	0.0002	0.000366	0.0103	0.0030	0.000367	0.0113	0.00526	0.0017	0.2614	0.002	3	2014 03 22	21:12
273.149	0.69319	9.7659	0.0002	0.000366	0.0103	0.0030	0.000368	0.0113	0.00526	0.0017	0.2604	0.002	3	2014 03 22	21:42
273.147	0.69613	9.8101	0.0004	0.000364	0.0102	0.0030	0.000365	0.0112	0.00526	0.0017	0.2575	0.002	3	2014 03 22	22:12
273.147	0.69908	9.8558	0.0004	0.000365	0.0102	0.0030	0.000366	0.0112	0.00526	0.0017	0.2564	0.002	3	2014 03 22	22:42
273.149	0.70202	9.8996	0.0002	0.000362	0.0102	0.0030	0.000364	0.0112	0.00526	0.0017	0.2551	0.001	3	2014 03 22	23:12
273.146	0.70495	9.9435	0.0001	0.000363	0.0103	0.0030	0.000364	0.0113	0.00526	0.0018	0.2557	0.002	3	2014 03 22	23:42
273.149	0.70788	9.9870	0.0004	0.000362	0.0103	0.0030	0.000364	0.0113	0.00526	0.0018	0.2543	0.001	3	2014 03 23	00:12

T	p	rho	sigma_T	sigma_p	sigma_rho	u(T)	u(p)	u(rho)	u(MW)	u[rho(x)]	U_c/%	m_sorb	p_trans	date	time
(K)	(MPa)	(kg.m-3)	(K)	(MPa)	(kg.m-3)	(K)	(MPa)	(kg.m-3)	(g/mol)	(kg.m-3)	(k = 2)	(mg)			
# test: mp_1403s.dat (continued)															
273.147	0.71080	10.0322	0.0005	0.000360	0.0104	0.0030	0.000361	0.0115	0.00526	0.0018	0.2560	0.002	3	2014 03 23	00:42
273.149	0.71663	10.1217	0.0002	0.000359	0.0103	0.0030	0.000361	0.0113	0.00526	0.0018	0.2507	0.002	3	2014 03 23	01:42
273.146	0.71953	10.1658	0.0002	0.000359	0.0103	0.0030	0.000361	0.0113	0.00526	0.0018	0.2501	0.003	3	2014 03 23	02:12
273.149	0.72244	10.2105	0.0005	0.000360	0.0102	0.0030	0.000362	0.0112	0.00526	0.0018	0.2474	0.003	3	2014 03 23	02:42
273.148	0.72534	10.2531	0.0004	0.000357	0.0102	0.0030	0.000359	0.0112	0.00526	0.0018	0.2462	0.003	3	2014 03 23	03:12
273.147	0.72823	10.2974	0.0003	0.000357	0.0104	0.0030	0.000358	0.0114	0.00526	0.0018	0.2480	0.003	3	2014 03 23	03:42
273.147	0.73399	10.3860	0.0002	0.000355	0.0102	0.0030	0.000357	0.0112	0.00526	0.0018	0.2428	0.003	3	2014 03 23	04:42
273.148	0.73687	10.4313	0.0003	0.000357	0.0101	0.0030	0.000358	0.0112	0.00526	0.0018	0.2412	-0.004	3	2014 03 23	05:12
273.148	0.73974	10.4730	0.0001	0.000353	0.0100	0.0030	0.000355	0.0110	0.00526	0.0018	0.2378	0.002	3	2014 03 23	05:42
273.148	0.74260	10.5159	0.0001	0.000354	0.0100	0.0030	0.000355	0.0110	0.00526	0.0019	0.2366	0.003	3	2014 03 23	06:12
273.148	0.74545	10.5625	0.0001	0.000354	0.0100	0.0030	0.000355	0.0111	0.00526	0.0019	0.2363	0.003	3	2014 03 23	06:42
273.148	0.74830	10.6051	0.0002	0.000351	0.0100	0.0030	0.000353	0.0110	0.00526	0.0019	0.2339	0.003	3	2014 03 23	07:12
273.148	0.75114	10.6491	0.0001	0.000351	0.0101	0.0030	0.000352	0.0111	0.00526	0.0019	0.2342	0.003	3	2014 03 23	07:42
273.148	0.75398	10.6928	0.0001	0.000349	0.0101	0.0030	0.000350	0.0111	0.00526	0.0019	0.2335	0.002	3	2014 03 23	08:12
273.147	0.75680	10.7358	0.0001	0.000349	0.0099	0.0030	0.000350	0.0109	0.00526	0.0019	0.2298	0.002	3	2014 03 23	08:42
273.150	0.89431	12.8842	0.0002	0.000301	0.0090	0.0030	0.000303	0.0100	0.00526	0.0023	0.1764	-0.000	3	2014 03 23	09:12
273.147	0.89679	12.9252	0.0006	0.000310	0.0094	0.0031	0.000312	0.0104	0.00526	0.0023	0.1818	0.004	3	2014 03 23	09:42
273.147	0.89931	12.9658	0.0006	0.000316	0.0094	0.0031	0.000318	0.0104	0.00526	0.0023	0.1821	0.004	3	2014 03 23	10:12
273.150	0.90184	13.0048	0.0002	0.000310	0.0093	0.0030	0.000312	0.0103	0.00526	0.0023	0.1799	0.004	3	2014 03 23	10:42
273.146	0.90435	13.0473	0.0002	0.000311	0.0093	0.0030	0.000313	0.0104	0.00526	0.0023	0.1801	0.005	3	2014 03 23	11:12
273.148	0.90687	13.0857	0.0005	0.000313	0.0092	0.0030	0.000315	0.0102	0.00526	0.0023	0.1783	0.006	3	2014 03 23	11:42
273.149	0.90937	13.1268	0.0006	0.000309	0.0094	0.0031	0.000311	0.0104	0.00526	0.0023	0.1797	0.004	3	2014 03 23	12:12
273.146	0.91185	13.1653	0.0002	0.000309	0.0093	0.0030	0.000311	0.0103	0.00526	0.0023	0.1776	0.005	3	2014 03 23	12:42
273.147	0.97223	14.1376	0.0007	0.000282	0.0086	0.0031	0.000284	0.0096	0.00526	0.0025	0.1557	0.006	3	2014 03 23	13:42
273.145	0.97453	14.1750	0.0003	0.000287	0.0089	0.0030	0.000290	0.0099	0.00526	0.0025	0.1591	0.006	3	2014 03 23	14:12
273.147	0.97685	14.2117	0.0001	0.000285	0.0087	0.0030	0.000287	0.0097	0.00526	0.0025	0.1564	0.007	3	2014 03 23	14:42
273.144	0.97914	14.2472	0.0003	0.000284	0.0087	0.0030	0.000286	0.0098	0.00526	0.0025	0.1560	0.008	3	2014 03 23	15:12
273.146	0.98144	14.2868	0.0005	0.000285	0.0087	0.0030	0.000287	0.0097	0.00526	0.0025	0.1549	0.010	3	2014 03 23	15:42
273.146	0.98373	14.3239	0.0004	0.000281	0.0087	0.0030	0.000283	0.0097	0.00526	0.0025	0.1540	0.008	3	2014 03 23	16:12
273.144	0.98600	14.3620	0.0002	0.000284	0.0088	0.0030	0.000286	0.0098	0.00526	0.0025	0.1558	0.008	3	2014 03 23	16:42
273.146	0.98828	14.3980	0.0001	0.000281	0.0084	0.0030	0.000284	0.0094	0.00526	0.0025	0.1502	0.009	3	2014 03 23	17:12
273.145	0.99055	14.4334	0.0002	0.000280	0.0085	0.0030	0.000283	0.0095	0.00526	0.0025	0.1512	0.008	3	2014 03 23	17:42
273.145	0.99281	14.4724	0.0004	0.000282	0.0082	0.0030	0.000285	0.0092	0.00526	0.0025	0.1473	0.011	3	2014 03 23	18:12
273.146	0.99508	14.5091	0.0003	0.000278	0.0085	0.0030	0.000280	0.0095	0.00526	0.0026	0.1497	0.008	3	2014 03 23	18:42
273.144	0.99732	14.5454	0.0001	0.000278	0.0084	0.0030	0.000280	0.0094	0.00526	0.0026	0.1485	0.011	3	2014 03 23	19:12
273.146	0.99957	14.5829	0.0002	0.000278	0.0084	0.0030	0.000281	0.0094	0.00526	0.0026	0.1476	0.010	3	2014 03 23	19:42
273.145	1.00181	14.6187	0.0003	0.000277	0.0085	0.0030	0.000279	0.0095	0.00526	0.0026	0.1491	0.012	3	2014 03 23	20:12
273.145	1.00405	14.6559	0.0004	0.000278	0.0082	0.0030	0.000280	0.0092	0.00526	0.0026	0.1447	0.012	3	2014 03 23	20:42
273.146	1.00628	14.6914	0.0004	0.000274	0.0086	0.0030	0.000276	0.0096	0.00526	0.0026	0.1488	0.012	3	2014 03 23	21:12
273.144	1.00850	14.7290	0.0001	0.000276	0.0084	0.0030	0.000279	0.0094	0.00526	0.0026	0.1466	0.014	3	2014 03 23	21:42
273.146	1.01072	14.7640	0.0001	0.000272	0.0084	0.0030	0.000275	0.0094	0.00526	0.0026	0.1454	0.013	3	2014 03 23	22:12
273.145	1.01293	14.8020	0.0001	0.000273	0.0076	0.0030	0.000275	0.0086	0.00526	0.0026	0.1359	0.013	3	2014 03 23	22:42
273.146	1.01513	14.8355	0.0002	0.000273	0.0083	0.0030	0.000275	0.0093	0.00526	0.0026	0.1438	0.015	3	2014 03 23	23:12
273.146	1.01733	14.8722	0.0003	0.000269	0.0083	0.0030	0.000272	0.0093	0.00526	0.0026	0.1440	0.016	3	2014 03 23	23:42
273.145	1.01952	14.9093	0.0002	0.000271	0.0082	0.0030	0.000274	0.0092	0.00526	0.0026	0.1419	0.017	3	2014 03 24	00:12
273.146	1.02170	14.9421	0.0001	0.000268	0.0082	0.0030	0.000270	0.0092	0.00526	0.0026	0.1419	0.016	3	2014 03 24	00:42
273.145	1.02604	15.0153	0.0003	0.000268	0.0082	0.0030	0.000270	0.0092	0.00526	0.0026	0.1411	0.020	3	2014 03 24	01:42
273.146	1.02820	15.0506	0.0003	0.000266	0.0081	0.0030	0.000268	0.0091	0.00526	0.0026	0.1392	0.022	3	2014 03 24	02:12
273.145	1.03035	15.0849	0.0003	0.000267	0.0079	0.0030	0.000269	0.0089	0.00526	0.0027	0.1373	0.024	3	2014 03 24	02:42
273.146	1.03250	15.1215	0.0001	0.000263	0.0078	0.0030	0.000266	0.0089	0.00526	0.0027	0.1357	0.029	3	2014 03 24	03:12
273.144	1.03462	15.1540	0.0001	0.000261	0.0079	0.0030	0.000264	0.0089	0.00526	0.0027	0.1362	0.033	3	2014 03 24	03:42
273.146	1.03675	15.1881	0.0003	0.000262	0.0076	0.0030	0.000264	0.0086	0.00526	0.0027	0.1321	0.040	3	2014 03 24	04:12
273.145	1.03883	15.2215	0.0004	0.000254	0.0070	0.0030	0.000257	0.0080	0.00526	0.0027	0.1247	0.049	3	2014 03 24	04:42
273.144	1.04087	15.2520	0.0003	0.000251	0.0059	0.0030	0.000254	0.0069	0.00526	0.0027	0.1118	0.073	3	2014 03 24	05:12

T (K)	p (MPa)	rho (kg.m-3)	sigma_T (K)	sigma_p (MPa)	sigma_rho (kg.m-3)	u(T) (K)	u(p) (MPa)	u(rho) (kg.m-3)	u(MW) (g/mol)	u[rho(x)] (kg.m-3)	U_c/% (k = 2)	m_sorb (mg)	p_trans	date	time
# test: mp_1403s.dat (continued)															
273.147	1.04283	15.2784	0.0001	0.000228	0.0032	0.0030	0.000231	0.0042	0.00526	0.0027	0.0828	0.113	3	2014 03 24	05:42
273.144	1.04454	15.2950	0.0003	0.000196	0.0020	0.0030	0.000200	0.0031	0.00526	0.0027	0.0691	0.189	3	2014 03 24	06:12
273.145	1.04606	15.2966	0.0004	0.000182	0.0045	0.0030	0.000186	0.0055	0.00526	0.0027	0.0903	0.288	3	2014 03 24	06:42
273.145	1.04744	15.2994	0.0003	0.000164	0.0037	0.0030	0.000168	0.0048	0.00526	0.0027	0.0804	0.376	3	2014 03 24	07:12
273.145	1.04878	15.2979	0.0003	0.000168	0.0057	0.0030	0.000172	0.0067	0.00526	0.0027	0.1019	0.464	3	2014 03 24	07:42
273.146	1.05011	15.2941	0.0002	0.000158	0.0022	0.0030	0.000162	0.0032	0.00526	0.0027	0.0656	0.538	3	2014 03 24	08:12
273.144	1.05140	15.2940	0.0002	0.000163	0.0036	0.0030	0.000167	0.0046	0.00526	0.0027	0.0786	0.608	3	2014 03 24	08:42
273.146	1.05272	15.2912	0.0001	0.000161	0.0033	0.0030	0.000165	0.0043	0.00526	0.0027	0.0761	0.694	3	2014 03 24	09:12
273.145	1.05401	15.2916	0.0003	0.000157	0.0023	0.0030	0.000161	0.0034	0.00526	0.0027	0.0665	0.761	3	2014 03 24	09:42
273.145	1.05530	15.2933	0.0003	0.000164	0.0039	0.0030	0.000168	0.0049	0.00526	0.0027	0.0822	0.828	3	2014 03 24	10:12
273.147	1.05663	15.2897	0.0001	0.000158	0.0022	0.0030	0.000162	0.0032	0.00526	0.0027	0.0655	1.628	3	2014 03 24	10:42
273.145	1.05788	15.2975	0.0003	0.000158	0.0012	0.0030	0.000162	0.0023	0.00526	0.0027	0.0582	1.531	3	2014 03 24	11:12
273.145	1.05919	15.3027	0.0003	0.000162	0.0037	0.0030	0.000166	0.0047	0.00526	0.0027	0.0796	1.616	3	2014 03 24	11:42
273.146	1.06047	15.2934	0.0002	0.000156	0.0007	0.0030	0.000160	0.0018	0.00526	0.0027	0.0553	3.651	3	2014 03 24	12:12
273.144	1.06175	15.2927	0.0002	0.000162	0.0034	0.0030	0.000166	0.0044	0.00526	0.0027	0.0772	7.304	3	2014 03 24	12:42
273.146	1.06306	15.2900	0.0001	0.000159	0.0034	0.0030	0.000163	0.0045	0.00526	0.0027	0.0770	9.556	3	2014 03 24	13:12
273.145	1.06432	15.2638	0.0002	0.000158	0.0064	0.0030	0.000163	0.0074	0.00526	0.0027	0.1097	9.711	3	2014 03 24	13:42
273.146	1.06563	15.2888	0.0003	0.000165	0.0052	0.0030	0.000169	0.0062	0.00526	0.0027	0.0958	10.799	3	2014 03 24	14:12
273.146	1.06691	15.2779	0.0003	0.000153	0.0016	0.0030	0.000157	0.0026	0.00526	0.0027	0.0601	11.839	3	2014 03 24	14:42
273.144	1.06817	15.2782	0.0000	0.000163	0.0050	0.0030	0.000167	0.0060	0.00526	0.0027	0.0937	13.361	3	2014 03 24	15:12
273.147	1.06951	15.2646	0.0004	0.000161	0.0030	0.0030	0.000165	0.0040	0.00526	0.0027	0.0731	13.838	3	2014 03 24	15:42
273.145	1.07075	15.2799	0.0004	0.000153	0.0028	0.0030	0.000157	0.0038	0.00526	0.0027	0.0702	16.577	3	2014 03 24	16:12

____

* Leaking valve resulted in continuously increasing pressure and resulted in large sigma_p and sigma_rho for this test, but time-symmetric weighing design compensated for this effect. Thus, the combined uncertainty is overstated; see McLinden, M. O., J. Chem. Eng. Data 2011, 56, 606-613.

Table S6. Experimental (p, rho, T, x) data and detailed uncertainty information for isotherms measured on the (0.74977 methane + 0.25023 propane) mixture

```
# component(s): /MPW/REFPROP_gcode/REFPROP910/fluids/methane.fld             mole frac:    0.749766
#               /MPW/REFPROP_gcode/REFPROP910/fluids/propane.fld                            0.250234
#
# molar mass:          23.062314
# nc =  2
# Uncertainty factors [all for standard (k = 1) errors]:
# state point:
#   standard uncertainty in T/K:                            0.0030
#   (p_trans = 1): zero offset in p/kPa                     1.000
#                  relative uncertainty in p/ppm:          26.0
#   (p_trans = 2): zero offset in p/kPa                     0.150
#                  relative uncertainty in p/ppm:          20.0
#   (p_trans = 3): zero offset in p/kPa                     0.030
#                  relative uncertainty in p/ppm:          20.0
#   hydrostatic head, uncertainty in L/V height (m):        0.050
# sinker volume
#   u(V_sinker)/ppm at Tref                                28.0
#   u(V_sinker)_temperature coeff [ppm/K]                   0.375
#   u(V_sinker)_pressure coeff [ppm/MPa]                    0.625
# density [except for V_sinker]
#   u_rho/[kg/m^3]                                          0.00100
# sample
#   sample purity, u(rho) [relative error]:                 0.000000
#   gravimetric prep [uncertainty in MW]                    0.000160
#   sorption of sample [uncertainty in MW]                  0.005200
```

```
# test:  mp_1402l.dat
# chiS [specific magnetic susceptibility] = -0.1131E-07 [m3/kg]
```

T (K)	p (MPa)	rho (kg.m-3)	sigma_T (K)	sigma_p (MPa)	sigma_rho (kg.m-3)	u(T) (K)	u(p) (MPa)	u(rho) (kg.m-3)	u(MW) (g/mol)	u[rho(x)] (kg.m-3)	U_c/% (k = 2)	m_sorb (mg)	p_trans	date	time
248.148	0.40848	4.7031	0.0012	0.000617	0.0137	0.0032	0.000618	0.0147	0.00520	0.0011	0.7003	−0.012	3	2014 02 20	18:46
248.146	0.41059	4.7265	0.0004	0.000587	0.0129	0.0030	0.000587	0.0139	0.00520	0.0011	0.6588	−0.010	3	2014 02 20	18:59
248.146	0.41260	4.7496	0.0006	0.000559	0.0122	0.0031	0.000560	0.0132	0.00520	0.0011	0.6243	−0.010	3	2014 02 20	19:12
248.149	0.41451	4.7726	0.0007	0.000535	0.0115	0.0031	0.000535	0.0125	0.00520	0.0011	0.5906	−0.010	3	2014 02 20	19:25
248.146	0.52278	6.0702	0.0004	0.000392	0.0087	0.0030	0.000393	0.0097	0.00520	0.0014	0.3589	−0.006	3	2014 02 20	20:29
248.148	0.52413	6.0860	0.0006	0.000375	0.0083	0.0031	0.000377	0.0093	0.00520	0.0014	0.3442	−0.007	3	2014 02 20	20:42
248.150	0.52542	6.1002	0.0002	0.000362	0.0083	0.0030	0.000364	0.0093	0.00520	0.0014	0.3391	−0.005	3	2014 02 20	20:55
248.150	0.52667	6.1166	0.0004	0.000350	0.0079	0.0030	0.000351	0.0089	0.00520	0.0014	0.3245	−0.006	3	2014 02 20	21:08
248.149	0.60393	7.0575	0.0001	0.000205	0.0052	0.0030	0.000208	0.0062	0.00520	0.0016	0.1963	−0.007	3	2014 02 20	22:11
248.149	0.60464	7.0668	0.0001	0.000197	0.0048	0.0030	0.000200	0.0058	0.00520	0.0016	0.1832	−0.005	3	2014 02 20	22:24
248.148	0.60531	7.0747	0.0002	0.000188	0.0046	0.0030	0.000191	0.0056	0.00520	0.0016	0.1775	−0.007	3	2014 02 20	22:37
248.148	0.60596	7.0820	0.0000	0.000182	0.0045	0.0030	0.000185	0.0055	0.00520	0.0016	0.1775	−0.054	3	2014 02 20	22:50
248.147	0.70432	8.2966	0.0002	0.000187	0.0046	0.0030	0.000190	0.0056	0.00520	0.0019	0.1540	−0.004	3	2014 02 20	23:54
248.147	0.70497	8.3037	0.0003	0.000183	0.0043	0.0030	0.000186	0.0053	0.00520	0.0019	0.1463	−0.002	3	2014 02 21	00:07
248.148	0.70560	8.3116	0.0005	0.000178	0.0042	0.0030	0.000181	0.0052	0.00520	0.0019	0.1439	−0.005	3	2014 02 21	00:20
248.150	0.70622	8.3220	0.0003	0.000173	0.0041	0.0030	0.000176	0.0051	0.00520	0.0019	0.1418	−0.004	3	2014 02 21	00:33
248.147	0.82423	9.8035	0.0002	0.000184	0.0046	0.0030	0.000187	0.0056	0.00520	0.0022	0.1322	0.000	3	2014 02 21	01:38
248.148	0.82487	9.8118	0.0003	0.000181	0.0044	0.0030	0.000184	0.0054	0.00520	0.0022	0.1280	0.002	3	2014 02 21	01:51
248.149	0.82549	9.8205	0.0002	0.000176	0.0044	0.0030	0.000179	0.0055	0.00520	0.0022	0.1286	0.005	3	2014 02 21	02:04
248.148	0.82610	9.8288	0.0003	0.000169	0.0040	0.0030	0.000173	0.0050	0.00520	0.0022	0.1199	0.004	3	2014 02 21	02:17
248.148	0.87238	10.4197	0.0003	0.000096	0.0025	0.0030	0.000102	0.0035	0.00520	0.0024	0.0846	0.006	3	2014 02 21	03:18
248.149	0.87270	10.4221	0.0002	0.000089	0.0023	0.0030	0.000096	0.0033	0.00520	0.0024	0.0813	0.009	3	2014 02 21	03:31
248.148	0.87300	10.4268	0.0003	0.000086	0.0023	0.0030	0.000093	0.0033	0.00520	0.0024	0.0815	0.009	3	2014 02 21	03:44
248.146	0.87330	10.4304	0.0002	0.000082	0.0020	0.0030	0.000089	0.0030	0.00520	0.0024	0.0769	0.007	3	2014 02 21	03:57

T (K)	p (MPa)	rho (kg.m−3)	sigma_T (K)	sigma_p (MPa)	sigma_rho (kg.m−3)	u(T) (K)	u(p) (MPa)	u(rho) (kg.m−3)	u(MW) (g/mol)	u[rho(x)] (kg.m−3)	U_c/% (k = 2)	m_sorb (mg)	p_trans	date	time
# test: mp_1402l.dat (continued)															
248.147	0.89650	10.7247	0.0003	0.000078	0.0016	0.0030	0.000086	0.0026	0.00520	0.0024	0.0699	0.040	3	2014 02 21	04:58
248.146	0.89677	10.7293	0.0001	0.000075	0.0017	0.0030	0.000083	0.0028	0.00520	0.0024	0.0715	0.041	3	2014 02 21	05:11
248.147	0.89703	10.7310	0.0002	0.000075	0.0016	0.0030	0.000083	0.0026	0.00520	0.0024	0.0691	0.044	3	2014 02 21	05:24
248.148	0.89729	10.7332	0.0004	0.000072	0.0017	0.0030	0.000080	0.0028	0.00520	0.0024	0.0712	0.047	3	2014 02 21	05:37
248.146	0.91685	10.8603	0.0001	0.000082	0.0190	0.0030	0.000089	0.0200	0.00520	0.0024	0.3718	0.206	3	2014 02 21	06:38
248.147	0.91714	10.8480	0.0004	0.000084	0.0138	0.0030	0.000091	0.0148	0.00520	0.0024	0.2775	0.596	3	2014 02 21	06:51
248.149	0.91743	10.8638	0.0003	0.000081	0.0031	0.0030	0.000089	0.0041	0.00520	0.0025	0.0902	0.293	3	2014 02 21	07:04
248.149	0.91770	10.8623	0.0001	0.000068	0.0014	0.0030	0.000077	0.0025	0.00520	0.0025	0.0668	0.295	3	2014 02 21	07:17
248.147	0.93655	10.9646	0.0005	0.000083	0.0278	0.0030	0.000090	0.0288	0.00520	0.0025	0.5286	0.335	3	2014 02 21	08:18
248.149	0.93683	10.9950	0.0002	0.000078	0.0035	0.0030	0.000085	0.0045	0.00520	0.0025	0.0959	0.652	3	2014 02 21	08:31
248.149	0.93709	10.9966	0.0002	0.000070	0.0009	0.0030	0.000078	0.0020	0.00520	0.0025	0.0609	0.664	3	2014 02 21	08:44
248.148	0.93734	10.9991	0.0004	0.000071	0.0061	0.0030	0.000079	0.0071	0.00520	0.0025	0.1385	0.659	3	2014 02 21	08:57
248.148	0.95581	11.1205	0.0001	0.000057	0.0041	0.0030	0.000067	0.0052	0.00520	0.0025	0.1043	4.262	3	2014 02 21	09:57
248.147	0.95601	11.1204	0.0003	0.000057	0.0018	0.0030	0.000067	0.0028	0.00520	0.0025	0.0700	4.060	3	2014 02 21	10:10
248.146	0.95621	11.1219	0.0004	0.000055	0.0025	0.0030	0.000065	0.0035	0.00520	0.0025	0.0793	7.850	3	2014 02 21	10:23
248.146	0.95641	11.1142	0.0002	0.000059	0.0051	0.0030	0.000069	0.0061	0.00520	0.0025	0.1197	7.789	3	2014 02 21	10:36

Table S6. Experimental (p, rho, T, x) data and detailed uncertainty information for isotherms measured on the (0.74977 methane + 0.25023 propane) mixture (continued)

test: mp_1402h.dat
chiS [specific magnetic susceptibility] = −0.1237E−07 [m3/kg]

T (K)	p (MPa)	rho (kg.m−3)	sigma_T (K)	sigma_p (MPa)	sigma_rho (kg.m−3)	u(T) (K)	u(p) (MPa)	u(rho) (kg.m−3)	u(MW) (g/mol)	u[rho(x)] (kg.m−3)	U_c/% (k = 2)	m_sorb (mg)	p_trans	date	time
273.146	0.56464	5.9105	0.0007	0.000134	0.0031	0.0031	0.000202	0.0041	0.00520	0.0013	0.1645	0.001	2	2014 02 16	23:23
273.145	0.56512	5.9174	0.0002	0.000134	0.0028	0.0030	0.000201	0.0038	0.00520	0.0013	0.1552	0.002	2	2014 02 16	23:36
273.146	0.56559	5.9235	0.0005	0.000138	0.0028	0.0030	0.000204	0.0038	0.00520	0.0013	0.1544	0.001	2	2014 02 16	23:49
273.148	0.56606	5.9274	0.0006	0.000135	0.0027	0.0031	0.000202	0.0037	0.00520	0.0013	0.1529	0.001	2	2014 02 17	00:02
273.144	0.89492	9.5468	0.0003	0.000034	0.0010	0.0030	0.000155	0.0021	0.00520	0.0022	0.0724	0.001	2	2014 02 17	01:04
273.146	0.89504	9.5485	0.0007	0.000036	0.0010	0.0031	0.000155	0.0021	0.00520	0.0022	0.0723	0.001	2	2014 02 17	01:17
273.148	0.89516	9.5493	0.0005	0.000036	0.0009	0.0030	0.000155	0.0019	0.00520	0.0022	0.0706	0.003	2	2014 02 17	01:30
273.149	0.89528	9.5508	0.0003	0.000034	0.0011	0.0030	0.000155	0.0021	0.00520	0.0022	0.0730	0.002	2	2014 02 17	01:43
273.146	1.19511	12.9760	0.0005	0.000039	0.0007	0.0030	0.000157	0.0018	0.00520	0.0029	0.0597	0.003	2	2014 02 17	02:44
273.147	1.19498	12.9733	0.0004	0.000034	0.0005	0.0030	0.000156	0.0016	0.00520	0.0029	0.0589	0.005	2	2014 02 17	02:57
273.148	1.19485	12.9715	0.0000	0.000038	0.0007	0.0030	0.000157	0.0018	0.00520	0.0029	0.0601	0.004	2	2014 02 17	03:10
273.147	1.19473	12.9699	0.0005	0.000036	0.0006	0.0030	0.000156	0.0017	0.00520	0.0029	0.0595	0.004	2	2014 02 17	03:23
273.146	1.46875	16.2111	0.0002	0.000122	0.0024	0.0030	0.000196	0.0034	0.00520	0.0037	0.0682	0.003	2	2014 02 17	04:24
273.146	1.46835	16.2102	0.0001	0.000115	0.0023	0.0030	0.000191	0.0033	0.00520	0.0037	0.0674	0.005	2	2014 02 17	04:37
273.146	1.46795	16.2040	0.0002	0.000115	0.0023	0.0030	0.000191	0.0033	0.00520	0.0037	0.0675	0.004	2	2014 02 17	04:50
273.147	1.46754	16.1983	0.0002	0.000117	0.0021	0.0030	0.000192	0.0031	0.00520	0.0037	0.0661	0.005	2	2014 02 17	05:03
273.145	1.71881	19.2741	0.0002	0.000156	0.0033	0.0030	0.000219	0.0044	0.00520	0.0043	0.0700	0.004	2	2014 02 17	06:05
273.145	1.71827	19.2697	0.0003	0.000157	0.0034	0.0030	0.000220	0.0045	0.00520	0.0043	0.0707	0.006	2	2014 02 17	06:18
273.147	1.71772	19.2637	0.0005	0.000154	0.0034	0.0030	0.000218	0.0045	0.00520	0.0043	0.0707	0.006	2	2014 02 17	06:31
273.148	1.71718	19.2556	0.0002	0.000155	0.0035	0.0030	0.000218	0.0045	0.00520	0.0043	0.0712	0.005	2	2014 02 17	06:44
273.144	2.05854	23.6096	0.0003	0.000196	0.0045	0.0030	0.000251	0.0055	0.00520	0.0053	0.0709	0.006	2	2014 02 17	07:48
273.146	2.05785	23.6007	0.0006	0.000196	0.0046	0.0031	0.000250	0.0056	0.00520	0.0053	0.0714	0.008	2	2014 02 17	08:01
273.148	2.05715	23.5921	0.0002	0.000202	0.0046	0.0030	0.000255	0.0056	0.00520	0.0053	0.0714	0.007	2	2014 02 17	08:14
273.144	2.35882	27.6171	0.0003	0.000296	0.0066	0.0030	0.000335	0.0077	0.00520	0.0062	0.0791	0.027	2	2014 02 17	09:18
273.146	2.35780	27.6041	0.0005	0.000282	0.0068	0.0030	0.000323	0.0079	0.00520	0.0062	0.0797	0.029	2	2014 02 17	09:31
273.148	2.35679	27.5900	0.0006	0.000292	0.0068	0.0031	0.000332	0.0078	0.00520	0.0062	0.0797	0.029	2	2014 02 17	09:44
273.144	2.35212	27.5267	0.0007	0.000288	0.0071	0.0031	0.000328	0.0082	0.00520	0.0062	0.0814	0.025	2	2014 02 17	10:43
273.146	2.35164	27.5200	0.0006	0.000225	0.0050	0.0031	0.000275	0.0061	0.00520	0.0062	0.0688	0.010	2	2014 02 17	10:56
273.148	2.35053	27.5040	0.0002	0.000305	0.0071	0.0030	0.000343	0.0081	0.00520	0.0062	0.0819	0.024	2	2014 02 17	11:11
273.146	2.34947	27.4917	0.0006	0.000303	0.0069	0.0031	0.000341	0.0079	0.00520	0.0062	0.0809	0.025	2	2014 02 17	11:24
273.147	2.38165	27.9203	0.0002	0.000242	0.0045	0.0030	0.000289	0.0056	0.00520	0.0063	0.0669	0.078	2	2014 02 17	12:25
273.146	2.38079	27.9081	0.0004	0.000247	0.0047	0.0030	0.000293	0.0058	0.00520	0.0063	0.0679	0.072	2	2014 02 17	12:38
273.145	2.37994	27.8981	0.0002	0.000240	0.0050	0.0030	0.000287	0.0061	0.00520	0.0063	0.0689	0.068	2	2014 02 17	12:51
273.145	2.37910	27.8882	0.0003	0.000239	0.0049	0.0030	0.000286	0.0060	0.00520	0.0063	0.0683	0.064	2	2014 02 17	13:04
273.144	2.39281	28.0431	0.0005	0.000213	0.0026	0.0030	0.000265	0.0037	0.00520	0.0063	0.0585	0.210	2	2014 02 17	14:06
273.144	2.39207	28.0392	0.0002	0.000212	0.0021	0.0030	0.000264	0.0033	0.00520	0.0063	0.0572	0.199	2	2014 02 17	14:19
273.145	2.39135	28.0278	0.0005	0.000206	0.0019	0.0030	0.000260	0.0031	0.00520	0.0063	0.0564	0.189	2	2014 02 17	14:32
273.144	2.40447	28.1336	0.0002	0.000196	0.0043	0.0030	0.000252	0.0054	0.00520	0.0063	0.0643	0.377	2	2014 02 17	15:34
273.145	2.40381	28.1227	0.0005	0.000183	0.0012	0.0030	0.000241	0.0025	0.00520	0.0063	0.0541	0.345	2	2014 02 17	15:47
273.147	2.40317	28.1158	0.0005	0.000186	0.0015	0.0030	0.000244	0.0027	0.00520	0.0063	0.0546	0.311	2	2014 02 17	16:00
273.148	2.40249	28.1098	0.0001	0.000195	0.0023	0.0030	0.000250	0.0034	0.00520	0.0063	0.0569	0.281	2	2014 02 17	16:13
273.146	2.41525	28.1674	0.0005	0.000190	0.0069	0.0030	0.000247	0.0079	0.00520	0.0064	0.0762	1.719	2	2014 02 17	17:13
273.147	2.41458	28.1460	0.0003	0.000191	0.0023	0.0030	0.000248	0.0034	0.00520	0.0063	0.0567	2.237	2	2014 02 17	17:26
273.147	2.41388	28.1719	0.0002	0.000202	0.0009	0.0030	0.000256	0.0022	0.00520	0.0064	0.0541	1.979	2	2014 02 17	17:39
273.146	2.41315	28.1473	0.0004	0.000216	0.0030	0.0030	0.000267	0.0041	0.00520	0.0063	0.0599	1.996	2	2014 02 17	17:52

Table S6. Experimental (p, rho, T, x) data and detailed uncertainty information for isotherms measured on the (0.74977 methane + 0.25023 propane) mixture (continued)

test: mp_1402j.dat
chiS [specific magnetic susceptibility] = −0.1237E−07 [m3/kg]

T (K)	p (MPa)	rho (kg.m−3)	sigma_T (K)	sigma_p (MPa)	sigma_rho (kg.m−3)	u(T) (K)	u(p) (MPa)	u(rho) (kg.m−3)	u(MW) (g/mol)	u[rho(x)] (kg.m−3)	U_c/% (k = 2)	m_sorb (mg)	p_trans	date	time
273.148	0.57004	5.9652	0.0007	0.000935	0.0185	0.0031	0.000947	0.0195	0.00520	0.0013	0.7394	0.006	2	2014 02 18	13:51
273.146	0.57331	6.0008	0.0004	0.000935	0.0182	0.0030	0.000947	0.0192	0.00520	0.0014	0.7264	0.010	2	2014 02 18	14:04
273.146	0.57657	6.0381	0.0003	0.000928	0.0182	0.0030	0.000940	0.0192	0.00520	0.0014	0.7201	0.031	2	2014 02 18	14:17
273.147	0.57810	6.0545	0.0006	0.000005	0.0005	0.0031	0.000151	0.0015	0.00520	0.0014	0.0859	0.010	2	2014 02 18	14:30
273.144	0.91739	9.7978	0.0002	0.001135	0.0229	0.0030	0.001145	0.0239	0.00520	0.0022	0.5559	0.006	2	2014 02 18	15:33
273.145	0.91948	9.8222	0.0007	0.000005	0.0005	0.0031	0.000151	0.0016	0.00520	0.0022	0.0657	0.009	2	2014 02 18	15:46
273.147	0.91949	9.8215	0.0007	0.000005	0.0002	0.0031	0.000151	0.0013	0.00520	0.0022	0.0632	0.010	2	2014 02 18	15:59
273.149	0.91948	9.8193	0.0001	0.000005	0.0007	0.0030	0.000151	0.0017	0.00520	0.0022	0.0668	0.009	2	2014 02 18	16:12
273.146	1.22087	13.2729	0.0004	0.000019	0.0004	0.0030	0.000153	0.0016	0.00520	0.0030	0.0576	0.008	2	2014 02 18	17:13
273.147	1.22081	13.2706	0.0004	0.000019	0.0002	0.0030	0.000153	0.0014	0.00520	0.0030	0.0569	0.009	2	2014 02 18	17:26
273.148	1.22056	13.2716	0.0002	0.000107	0.0021	0.0030	0.000186	0.0031	0.00520	0.0030	0.0729	0.010	2	2014 02 18	17:43
273.146	1.22013	13.2659	0.0005	0.000125	0.0024	0.0030	0.000197	0.0034	0.00520	0.0030	0.0768	0.009	2	2014 02 18	17:56
273.145	1.48515	16.4104	0.0003	0.000159	0.0032	0.0030	0.000220	0.0042	0.00520	0.0037	0.0757	0.009	2	2014 02 18	18:57
273.145	1.48460	16.4038	0.0000	0.000154	0.0033	0.0030	0.000217	0.0043	0.00520	0.0037	0.0764	0.010	2	2014 02 18	19:10
273.145	1.48404	16.3982	0.0003	0.000163	0.0038	0.0030	0.000224	0.0048	0.00520	0.0037	0.0810	0.012	2	2014 02 18	19:23
273.147	1.48349	16.3923	0.0004	0.000153	0.0033	0.0030	0.000216	0.0043	0.00520	0.0037	0.0762	0.010	2	2014 02 18	19:36
273.144	1.72873	19.3984	0.0002	0.000150	0.0030	0.0030	0.000215	0.0041	0.00520	0.0044	0.0677	0.010	2	2014 02 18	20:37
273.145	1.72822	19.3914	0.0007	0.000143	0.0030	0.0031	0.000210	0.0041	0.00520	0.0044	0.0675	0.012	2	2014 02 18	20:50
273.147	1.72771	19.3876	0.0009	0.000152	0.0033	0.0031	0.000216	0.0043	0.00520	0.0044	0.0695	0.013	2	2014 02 18	21:03
273.149	1.72719	19.3773	0.0002	0.000151	0.0030	0.0030	0.000215	0.0041	0.00520	0.0044	0.0677	0.012	2	2014 02 18	21:16
273.145	2.06048	23.6342	0.0003	0.000237	0.0052	0.0030	0.000284	0.0063	0.00520	0.0053	0.0764	0.010	2	2014 02 18	22:19
273.147	2.05965	23.6237	0.0006	0.000245	0.0053	0.0031	0.000290	0.0064	0.00520	0.0053	0.0774	0.012	2	2014 02 18	22:32
273.148	2.05880	23.6112	0.0002	0.000243	0.0054	0.0030	0.000289	0.0064	0.00520	0.0053	0.0777	0.014	2	2014 02 18	22:45
273.148	2.05794	23.6009	0.0004	0.000246	0.0054	0.0030	0.000291	0.0064	0.00520	0.0053	0.0777	0.014	2	2014 02 18	22:58
273.146	2.35244	27.5302	0.0002	0.000357	0.0084	0.0030	0.000390	0.0094	0.00520	0.0062	0.0907	0.026	2	2014 02 19	00:00
273.145	2.35119	27.5133	0.0001	0.000358	0.0083	0.0030	0.000391	0.0093	0.00520	0.0062	0.0903	0.026	2	2014 02 19	00:13
273.146	2.34994	27.4976	0.0003	0.000353	0.0084	0.0030	0.000387	0.0094	0.00520	0.0062	0.0905	0.026	2	2014 02 19	00:26
273.147	2.34871	27.4792	0.0003	0.000351	0.0085	0.0030	0.000385	0.0095	0.00520	0.0062	0.0912	0.026	2	2014 02 19	00:39
273.145	2.36010	27.6348	0.0004	0.000361	0.0084	0.0030	0.000394	0.0094	0.00520	0.0062	0.0905	0.031	2	2014 02 19	01:40
273.147	2.35885	27.6180	0.0005	0.000360	0.0085	0.0030	0.000393	0.0095	0.00520	0.0062	0.0912	0.031	2	2014 02 19	01:53
273.148	2.35759	27.4782	0.0001	0.000362	0.0085	0.0030	0.000395	0.0096	0.00520	0.0062	0.0919	0.029	2	2014 02 19	02:06
273.147	2.35632	27.5836	0.0003	0.000364	0.0083	0.0030	0.000396	0.0094	0.00520	0.0062	0.0906	0.029	2	2014 02 19	02:19
273.148	2.36804	27.7403	0.0001	0.000331	0.0074	0.0030	0.000366	0.0085	0.00520	0.0063	0.0842	0.036	2	2014 02 19	03:20
273.147	2.36687	27.7258	0.0002	0.000334	0.0076	0.0030	0.000369	0.0086	0.00520	0.0063	0.0851	0.033	2	2014 02 19	03:33
273.147	2.36570	27.7101	0.0003	0.000330	0.0074	0.0030	0.000366	0.0085	0.00520	0.0063	0.0843	0.034	2	2014 02 19	03:46
273.146	2.36455	27.6954	0.0001	0.000321	0.0074	0.0030	0.000358	0.0084	0.00520	0.0062	0.0838	0.033	2	2014 02 19	03:59
273.146	2.37637	27.8511	0.0003	0.000319	0.0066	0.0030	0.000356	0.0076	0.00520	0.0063	0.0794	0.047	2	2014 02 19	05:00
273.146	2.37525	27.8352	0.0001	0.000311	0.0067	0.0030	0.000348	0.0077	0.00520	0.0063	0.0795	0.047	2	2014 02 19	05:13
273.146	2.37416	27.8220	0.0003	0.000314	0.0069	0.0030	0.000351	0.0079	0.00520	0.0063	0.0807	0.042	2	2014 02 19	05:26
273.147	2.37305	27.8077	0.0003	0.000319	0.0071	0.0030	0.000356	0.0081	0.00520	0.0063	0.0818	0.040	2	2014 02 19	05:39
273.145	2.38486	27.9555	0.0003	0.000301	0.0053	0.0030	0.000339	0.0063	0.00520	0.0063	0.0722	0.084	2	2014 02 19	06:39
273.147	2.38382	27.9421	0.0004	0.000307	0.0052	0.0030	0.000345	0.0062	0.00520	0.0063	0.0721	0.077	2	2014 02 19	06:52
273.148	2.38276	27.9310	0.0002	0.000302	0.0055	0.0030	0.000340	0.0066	0.00520	0.0063	0.0734	0.067	2	2014 02 19	07:05
273.148	2.38169	27.9172	0.0002	0.000306	0.0062	0.0030	0.000344	0.0073	0.00520	0.0063	0.0768	0.062	2	2014 02 19	07:18
273.147	2.39400	28.0412	0.0003	0.000234	0.0025	0.0030	0.000282	0.0036	0.00520	0.0063	0.0589	0.162	2	2014 02 19	08:18
273.147	2.39317	28.0398	0.0001	0.000232	0.0020	0.0030	0.000280	0.0031	0.00520	0.0063	0.0575	0.153	2	2014 02 19	08:31
273.147	2.39233	28.0309	0.0003	0.000243	0.0021	0.0030	0.000289	0.0033	0.00520	0.0063	0.0583	0.147	2	2014 02 19	08:44
273.146	2.39148	28.0231	0.0003	0.000238	0.0025	0.0030	0.000285	0.0036	0.00520	0.0063	0.0591	0.140	2	2014 02 19	08:57

T (K)	p (MPa)	rho (kg.m-3)	sigma_T (K)	sigma_p (MPa)	sigma_rho (kg.m-3)	u(T) (K)	u(p) (MPa)	u(rho) (kg.m-3)	u(MW) (g/mol)	u[rho(x)] (kg.m-3)	U_c/% (k = 2)	m_sorb (mg)	p_trans	date	time
# test: mp_1402j.dat (continued)															
273.147	2.40475	28.1034	0.0003	0.000141	0.0037	0.0030	0.000211	0.0048	0.00520	0.0063	0.0603	0.477	2	2014 02 19	09:57
273.146	2.40424	28.1071	0.0004	0.000145	0.0017	0.0030	0.000214	0.0029	0.00520	0.0063	0.0540	0.242	2	2014 02 19	10:10
273.145	2.40375	28.0993	0.0002	0.000136	0.0024	0.0030	0.000208	0.0035	0.00520	0.0063	0.0557	0.235	2	2014 02 19	10:23
273.145	2.40329	28.0968	0.0002	0.000131	0.0051	0.0030	0.000205	0.0062	0.00520	0.0063	0.0662	0.231	2	2014 02 19	10:36
273.146	2.41619	28.1721	0.0003	0.000145	0.0062	0.0030	0.000214	0.0073	0.00520	0.0064	0.0719	0.242	2	2014 02 19	11:36
273.145	2.41571	28.1485	0.0001	0.000138	0.0042	0.0030	0.000209	0.0053	0.00520	0.0063	0.0622	0.170	2	2014 02 19	11:49
273.146	2.41523	28.1324	0.0004	0.000138	0.0035	0.0030	0.000209	0.0046	0.00520	0.0063	0.0594	0.128	2	2014 02 19	12:02
273.148	2.41473	28.1175	0.0002	0.000140	0.0015	0.0030	0.000211	0.0027	0.00520	0.0063	0.0533	0.107	2	2014 02 19	12:15
273.145	2.42748	28.1783	0.0002	0.000133	0.0100	0.0030	0.000206	0.0111	0.00520	0.0064	0.0929	0.172	2	2014 02 19	13:15
273.146	2.42703	28.1754	0.0005	0.000129	0.0067	0.0030	0.000204	0.0077	0.00520	0.0064	0.0738	0.359	2	2014 02 19	13:28
273.148	2.42655	28.1714	0.0002	0.000143	0.0037	0.0030	0.000213	0.0048	0.00520	0.0064	0.0603	0.019	2	2014 02 19	13:41
273.147	2.42605	28.1523	0.0003	0.000142	0.0029	0.0030	0.000212	0.0040	0.00520	0.0064	0.0574	−0.031	2	2014 02 19	13:54
273.147	2.43881	28.2320	0.0003	0.000155	0.0093	0.0030	0.000221	0.0103	0.00520	0.0064	0.0887	5.493	2	2014 02 19	14:54

Table S6. Experimental (p, rho, T, x) data and detailed uncertainty information for isotherms measured on the (0.74977 methane + 0.25023 propane) mixture (continued)

test: mp_1402m.dat (continuously increase pressure near dew line)
chiS [specific magnetic susceptibility] = −0.1237E−07 [m3/kg]

T (K)	p (MPa)	rho (kg.m−3)	sigma_T (K)	sigma_p (MPa)	sigma_rho (kg.m−3)	u(T) (K)	u(p) (MPa)	u(rho) (kg.m−3)	u(MW) (g/mol)	u[rho(x)] (kg.m−3)	U_c/% (k = 2)	m_sorb (mg)	p_trans	date	time
273.139	0.63368	6.6576	0.0008	0.000064	0.0033	0.0031	0.000164	0.0043	0.00520	0.0015	0.1460	−0.001	2	2014 02 21	21:41
273.142	0.63389	6.6591	0.0010	0.000059	0.0011	0.0031	0.000162	0.0021	0.00520	0.0015	0.0945	−0.009	2	2014 02 21	21:54
273.144	0.63408	6.6634	0.0003	0.000049	0.0013	0.0030	0.000158	0.0023	0.00520	0.0015	0.0979	−0.000	2	2014 02 21	22:07
273.144	0.63424	6.6638	0.0003	0.000043	0.0016	0.0030	0.000156	0.0026	0.00520	0.0015	0.1046	−0.001	2	2014 02 21	22:20
273.143	0.63439	6.6649	0.0003	0.000039	0.0019	0.0030	0.000155	0.0029	0.00520	0.0015	0.1111	−0.004	2	2014 02 21	22:33
273.143	0.63452	6.6669	0.0001	0.000033	0.0021	0.0030	0.000154	0.0031	0.00520	0.0015	0.1141	−0.004	2	2014 02 21	22:46
273.143	0.63464	6.6691	0.0004	0.000031	0.0012	0.0030	0.000154	0.0022	0.00520	0.0015	0.0938	0.001	2	2014 02 21	22:59
273.145	0.63474	6.6705	0.0007	0.000025	0.0012	0.0031	0.000153	0.0022	0.00520	0.0015	0.0938	0.003	2	2014 02 21	23:12
273.145	1.20776	13.1207	0.0006	0.000027	0.0010	0.0031	0.000154	0.0021	0.00520	0.0030	0.0615	0.003	2	2014 02 22	00:38
273.147	1.20784	13.1213	0.0004	0.000019	0.0014	0.0030	0.000153	0.0025	0.00520	0.0030	0.0651	−0.007	2	2014 02 22	00:51
273.148	1.20788	13.1183	0.0001	0.000003	0.0008	0.0030	0.000152	0.0019	0.00520	0.0030	0.0603	−0.009	2	2014 02 22	01:04
273.146	1.20787	13.1193	0.0007	0.000009	0.0008	0.0031	0.000152	0.0018	0.00520	0.0030	0.0597	−0.007	2	2014 02 22	01:17
273.145	1.20783	13.1168	0.0003	0.000014	0.0013	0.0030	0.000153	0.0023	0.00520	0.0030	0.0637	−0.010	2	2014 02 22	01:30
273.144	1.20776	13.1169	0.0002	0.000021	0.0004	0.0030	0.000153	0.0015	0.00520	0.0030	0.0578	−0.011	2	2014 02 22	01:43
273.146	1.20768	13.1159	0.0006	0.000028	0.0006	0.0031	0.000154	0.0017	0.00520	0.0030	0.0589	−0.009	2	2014 02 22	01:56
273.148	1.20756	13.1166	0.0005	0.000033	0.0009	0.0030	0.000156	0.0020	0.00520	0.0030	0.0609	−0.009	2	2014 02 22	02:09
273.144	1.84603	20.8702	0.0002	0.000220	0.0044	0.0030	0.000269	0.0055	0.00520	0.0047	0.0766	−0.007	2	2014 02 22	03:26
273.145	1.84525	20.8599	0.0007	0.000231	0.0054	0.0031	0.000278	0.0064	0.00520	0.0047	0.0835	−0.006	2	2014 02 22	03:39
273.148	1.84444	20.8501	0.0007	0.000234	0.0047	0.0031	0.000281	0.0058	0.00520	0.0047	0.0792	−0.005	2	2014 02 22	03:52
273.149	1.84360	20.8417	0.0001	0.000243	0.0049	0.0030	0.000288	0.0059	0.00520	0.0047	0.0792	0.006	2	2014 02 22	04:05
273.147	1.84273	20.8307	0.0007	0.000253	0.0052	0.0031	0.000297	0.0062	0.00520	0.0047	0.0831	0.016	2	2014 02 22	04:18
273.145	1.84183	20.8214	0.0006	0.000255	0.0054	0.0031	0.000298	0.0065	0.00520	0.0047	0.0851	0.016	2	2014 02 22	04:31
273.144	1.84093	20.8075	0.0001	0.000255	0.0056	0.0030	0.000298	0.0066	0.00520	0.0047	0.0863	0.015	2	2014 02 22	04:44
273.146	1.84004	20.7969	0.0008	0.000252	0.0058	0.0031	0.000296	0.0068	0.00520	0.0047	0.0876	0.014	2	2014 02 22	04:57
273.144	2.04924	23.4832	0.0005	0.000289	0.0063	0.0030	0.000328	0.0074	0.00520	0.0053	0.0876	0.005	2	2014 02 22	06:05
273.146	2.04822	23.4695	0.0010	0.000291	0.0068	0.0031	0.000330	0.0079	0.00520	0.0053	0.0876	0.005	2	2014 02 22	06:18
273.149	2.04719	23.4559	0.0003	0.000302	0.0068	0.0030	0.000339	0.0079	0.00520	0.0053	0.0893	0.003	2	2014 02 22	06:31
273.148	2.04612	23.4420	0.0004	0.000306	0.0067	0.0030	0.000343	0.0077	0.00520	0.0053	0.0893	0.005	2	2014 02 22	06:44
273.146	2.04504	23.4289	0.0008	0.000318	0.0070	0.0031	0.000354	0.0080	0.00520	0.0053	0.0893	0.005	2	2014 02 22	06:57
273.144	2.04394	23.4156	0.0003	0.000307	0.0069	0.0030	0.000344	0.0079	0.00520	0.0053	0.0899	0.002	2	2014 02 22	07:10
273.145	2.04288	23.4005	0.0004	0.000309	0.0073	0.0030	0.000346	0.0083	0.00520	0.0053	0.0928	0.004	2	2014 02 22	07:23
273.147	2.04180	23.3876	0.0007	0.000310	0.0072	0.0031	0.000347	0.0082	0.00520	0.0053	0.0928	0.006	2	2014 02 22	07:36
273.149	2.03979	23.3606	0.0002	0.000322	0.0072	0.0030	0.000358	0.0082	0.00520	0.0053	0.0928	0.004	2	2014 02 22	08:00
273.147	2.03865	23.3488	0.0006	0.000324	0.0071	0.0031	0.000359	0.0081	0.00520	0.0053	0.0928	0.005	2	2014 02 22	08:13
273.145	2.03753	23.3327	0.0004	0.000318	0.0073	0.0030	0.000354	0.0084	0.00520	0.0053	0.0928	0.007	2	2014 02 22	08:26
273.145	2.05152	23.5118	0.0005	0.000137	0.0031	0.0030	0.000207	0.0042	0.00520	0.0053	0.0620	0.003	2	2014 02 22	08:51
273.147	2.05088	23.5058	0.0003	0.000177	0.0042	0.0030	0.000236	0.0052	0.00520	0.0053	0.0620	0.011	2	2014 02 22	09:05
273.147	2.06482	23.5827	0.0001	0.005782	0.1513	0.0030	0.005784	0.1523	0.00520	0.0053	1.4436	−1.092	2	2014 02 22	09:18
273.146	2.07232	23.8662	0.0005	0.007155	0.1465	0.0030	0.007157	0.1475	0.00520	0.0054	1.4664	0.650	2	2014 02 22	09:31
273.144	2.09389	24.0653	0.0002	0.001125	0.0266	0.0030	0.001135	0.0277	0.00520	0.0054	0.2652	−0.002	2	2014 02 22	09:55
273.145	2.09773	24.1182	0.0004	0.001085	0.0255	0.0030	0.001096	0.0265	0.00520	0.0054	0.2652	0.005	2	2014 02 22	10:08
273.147	2.10149	24.1677	0.0003	0.001061	0.0251	0.0030	0.001072	0.0262	0.00520	0.0055	0.2502	0.001	2	2014 02 22	10:21
273.147	2.10547	24.2185	0.0003	0.001049	0.0251	0.0030	0.001060	0.0261	0.00520	0.0055	0.2488	0.002	2	2014 02 22	10:35
273.146	2.10912	24.2690	0.0005	0.001038	0.0249	0.0030	0.001049	0.0259	0.00520	0.0055	0.2466	0.003	2	2014 02 22	10:48
273.144	2.11274	24.3153	0.0003	0.001028	0.0247	0.0030	0.001040	0.0257	0.00520	0.0055	0.2466	0.006	2	2014 02 22	11:01
273.144	2.11632	24.3626	0.0002	0.001024	0.0244	0.0030	0.001036	0.0254	0.00520	0.0055	0.2466	0.006	2	2014 02 22	11:14
273.147	2.12290	24.4505	0.0004	0.001013	0.0240	0.0030	0.001025	0.0250	0.00520	0.0055	0.2466	0.006	2	2014 02 22	11:38
273.147	2.12641	24.4951	0.0001	0.000996	0.0240	0.0030	0.001008	0.0250	0.00520	0.0055	0.2466	0.008	2	2014 02 22	11:51

test: mp_1402m.dat (continued)

T (K)	p (MPa)	rho (kg.m−3)	sigma_T (K)	sigma_p (MPa)	sigma_rho (kg.m−3)	u(T) (K)	u(p) (MPa)	u(rho) (kg.m−3)	u(MW) (g/mol)	u[rho(x)] (kg.m−3)	U_c/% (k = 2)	m_sorb (mg)	p_trans	date	time
273.146	2.13015	24.5466	0.0005	0.000988	0.0237	0.0030	0.001001	0.0247	0.00520	0.0055	0.2466	0.010	2	2014 02 22	12:05
273.145	2.13360	24.5904	0.0003	0.000984	0.0235	0.0030	0.000997	0.0245	0.00520	0.0055	0.2466	0.007	2	2014 02 22	12:18
273.144	2.13704	24.6365	0.0001	0.000987	0.0235	0.0030	0.000999	0.0245	0.00520	0.0056	0.2466	0.009	2	2014 02 22	12:31
273.146	2.14047	24.6819	0.0005	0.000979	0.0231	0.0030	0.000992	0.0241	0.00520	0.0056	0.2466	0.009	2	2014 02 22	12:44
273.147	2.14388	24.7264	0.0003	0.000971	0.0231	0.0030	0.000984	0.0241	0.00520	0.0056	0.2466	0.010	2	2014 02 22	12:57
273.146	2.15009	24.8089	0.0006	0.000955	0.0226	0.0031	0.000967	0.0237	0.00520	0.0056	0.2466	0.009	2	2014 02 22	13:21
273.144	2.15367	24.8547	0.0003	0.000962	0.0230	0.0030	0.000975	0.0240	0.00520	0.0056	0.2466	0.010	2	2014 02 22	13:35
273.144	2.15702	24.8993	0.0003	0.000957	0.0227	0.0030	0.000970	0.0237	0.00520	0.0056	0.2466	0.012	2	2014 02 22	13:48
273.146	2.16035	24.9422	0.0006	0.000945	0.0222	0.0031	0.000958	0.0232	0.00520	0.0056	0.2466	0.012	2	2014 02 22	14:01
273.147	2.16364	24.9856	0.0003	0.000938	0.0222	0.0030	0.000951	0.0232	0.00520	0.0056	0.2163	0.014	2	2014 02 22	14:14
273.147	2.16692	25.0305	0.0004	0.000932	0.0224	0.0030	0.000945	0.0234	0.00520	0.0056	0.2163	0.011	2	2014 02 22	14:27
273.145	2.17017	25.0717	0.0006	0.000926	0.0223	0.0031	0.000940	0.0233	0.00520	0.0057	0.2163	0.009	2	2014 02 22	14:40
273.144	2.17640	25.0378	0.0003	0.000923	0.0220	0.0030	0.000936	0.0230	0.00520	0.0056	0.2163	0.011	2	2014 02 22	15:05
273.145	2.17963	25.1994	0.0007	0.000919	0.0220	0.0031	0.000932	0.0230	0.00520	0.0057	0.2163	0.010	2	2014 02 22	15:18
273.147	2.18285	25.2430	0.0004	0.000920	0.0222	0.0030	0.000933	0.0233	0.00520	0.0057	0.2163	0.010	2	2014 02 22	15:31
273.148	2.18605	25.2845	0.0002	0.000907	0.0219	0.0030	0.000921	0.0229	0.00520	0.0057	0.2108	0.013	2	2014 02 22	15:44
273.146	2.18921	25.3273	0.0006	0.000902	0.0218	0.0031	0.000916	0.0228	0.00520	0.0057	0.2094	0.013	2	2014 02 22	15:57
273.144	2.19236	25.3697	0.0003	0.000902	0.0217	0.0030	0.000915	0.0227	0.00520	0.0057	0.2094	0.010	2	2014 02 22	16:10
273.143	2.19552	25.4115	0.0005	0.000901	0.0215	0.0030	0.000915	0.0225	0.00520	0.0057	0.2094	0.009	2	2014 02 22	16:23
273.147	2.20159	25.4896	0.0006	0.000895	0.0215	0.0031	0.000909	0.0226	0.00520	0.0058	0.2094	0.011	2	2014 02 22	16:48
273.148	2.20471	25.5307	0.0001	0.000879	0.0215	0.0030	0.000893	0.0225	0.00520	0.0058	0.2094	0.011	2	2014 02 22	17:01
273.146	2.20779	25.5727	0.0006	0.000883	0.0212	0.0031	0.000897	0.0223	0.00520	0.0058	0.2094	0.011	2	2014 02 22	17:14
273.144	2.21087	25.6152	0.0004	0.000883	0.0212	0.0030	0.000897	0.0222	0.00520	0.0058	0.2094	0.012	2	2014 02 22	17:27
273.144	2.21397	25.6574	0.0002	0.000880	0.0212	0.0030	0.000894	0.0222	0.00520	0.0058	0.2094	0.011	2	2014 02 22	17:40
273.145	2.21704	25.6969	0.0005	0.000876	0.0209	0.0030	0.000890	0.0219	0.00520	0.0058	0.2094	0.012	2	2014 02 22	17:53
273.147	2.22010	25.7374	0.0004	0.000870	0.0207	0.0030	0.000884	0.0217	0.00520	0.0058	0.2094	0.013	2	2014 02 22	18:06
273.147	2.22315	25.7785	0.0001	0.000869	0.0208	0.0030	0.000883	0.0218	0.00520	0.0058	0.2094	0.013	2	2014 02 22	18:19
273.145	2.22868	25.8536	0.0004	0.000853	0.0203	0.0030	0.000867	0.0214	0.00520	0.0058	0.1936	0.014	2	2014 02 22	18:43
273.144	2.23167	25.8908	0.0001	0.000854	0.0201	0.0030	0.000868	0.0211	0.00520	0.0058	0.1917	0.013	2	2014 02 22	18:56
273.145	2.23465	25.9317	0.0004	0.000853	0.0201	0.0030	0.000867	0.0212	0.00520	0.0058	0.1917	0.014	2	2014 02 22	19:09
273.146	2.23763	25.9725	0.0004	0.000846	0.0203	0.0030	0.000860	0.0213	0.00520	0.0059	0.1919	0.013	2	2014 02 22	19:22
273.147	2.24059	26.0120	0.0001	0.000835	0.0201	0.0030	0.000850	0.0212	0.00520	0.0059	0.1919	0.012	2	2014 02 22	19:35
273.146	2.24351	26.0516	0.0004	0.000831	0.0201	0.0030	0.000846	0.0212	0.00520	0.0059	0.1919	0.012	2	2014 02 22	19:48
273.145	2.24644	26.0898	0.0005	0.000836	0.0201	0.0030	0.000850	0.0212	0.00520	0.0059	0.1898	0.013	2	2014 02 22	20:01
273.144	2.24936	26.1288	0.0002	0.000836	0.0200	0.0030	0.000851	0.0211	0.00520	0.0059	0.1898	0.012	2	2014 02 22	20:14
273.146	2.25475	26.1999	0.0004	0.000827	0.0198	0.0030	0.000842	0.0209	0.00520	0.0059	0.1868	0.013	2	2014 02 22	20:38
273.147	2.25788	26.2426	0.0002	0.000824	0.0200	0.0030	0.000839	0.0210	0.00520	0.0059	0.1873	0.013	2	2014 02 22	20:52
273.146	2.26076	26.2824	0.0003	0.000821	0.0199	0.0030	0.000836	0.0209	0.00520	0.0059	0.1863	0.013	2	2014 02 22	21:05
273.145	2.26363	26.3205	0.0004	0.000817	0.0198	0.0030	0.000832	0.0208	0.00520	0.0059	0.1855	0.014	2	2014 02 22	21:18
273.144	2.26649	26.3583	0.0001	0.000816	0.0199	0.0030	0.000831	0.0209	0.00520	0.0059	0.1855	0.012	2	2014 02 22	21:31
273.144	2.26936	26.3988	0.0004	0.000820	0.0198	0.0030	0.000835	0.0209	0.00520	0.0060	0.1855	0.012	2	2014 02 22	21:44
273.146	2.27223	26.4363	0.0005	0.000816	0.0195	0.0030	0.000831	0.0206	0.00520	0.0060	0.1829	0.015	2	2014 02 22	21:57
273.147	2.27770	26.5104	0.0004	0.000803	0.0199	0.0030	0.000818	0.0209	0.00520	0.0060	0.1840	0.014	2	2014 02 22	22:22
273.145	2.28051	26.5485	0.0004	0.000807	0.0198	0.0030	0.000822	0.0208	0.00520	0.0060	0.1836	0.014	2	2014 02 22	22:35
273.144	2.28331	26.5854	0.0002	0.000801	0.0195	0.0030	0.000817	0.0205	0.00520	0.0060	0.1812	0.013	2	2014 02 22	22:48
273.144	2.28613	26.6247	0.0004	0.000805	0.0195	0.0030	0.000820	0.0205	0.00520	0.0060	0.1809	0.014	2	2014 02 22	23:01
273.146	2.28896	26.6618	0.0005	0.000802	0.0196	0.0030	0.000817	0.0206	0.00520	0.0060	0.1811	0.015	2	2014 02 22	23:14
273.147	2.29176	26.6994	0.0002	0.000799	0.0194	0.0030	0.000814	0.0204	0.00520	0.0060	0.1794	0.015	2	2014 02 22	23:27
273.146	2.29452	26.7362	0.0005	0.000787	0.0194	0.0030	0.000802	0.0204	0.00520	0.0060	0.1789	0.015	2	2014 02 22	23:40
273.143	2.29983	26.8082	0.0002	0.000790	0.0193	0.0030	0.000805	0.0203	0.00520	0.0060	0.1778	0.014	2	2014 02 23	00:05
273.144	2.30260	26.8478	0.0004	0.000790	0.0194	0.0030	0.000806	0.0204	0.00520	0.0061	0.1783	0.014	2	2014 02 23	00:18
273.146	2.30537	26.8847	0.0006	0.000785	0.0190	0.0031	0.000800	0.0200	0.00520	0.0061	0.1752	0.016	2	2014 02 23	00:31
273.147	2.30811	26.9193	0.0003	0.000784	0.0190	0.0030	0.000799	0.0201	0.00520	0.0061	0.1754	0.015	2	2014 02 23	00:44
273.147	2.31084	26.9576	0.0003	0.000779	0.0192	0.0030	0.000794	0.0202	0.00520	0.0061	0.1760	0.015	2	2014 02 23	00:57

test: mp_1402m.dat (continued)

T	p	rho	sigma_T	sigma_p	sigma_rho	u(T)	u(p)	u(rho)	u(MW)	u[rho(x)]	U_c/%	m_sorb	p_trans	date	time
(K)	(MPa)	(kg.m-3)	(K)	(MPa)	(kg.m-3)	(K)	(MPa)	(kg.m-3)	(g/mol)	(kg.m-3)	(k = 2)	(mg)			
273.145	2.31356	26.9933	0.0005	0.000772	0.0191	0.0030	0.000788	0.0202	0.00520	0.0061	0.1752	0.017	2	2014 02 23	01:10
273.144	2.31627	27.0310	0.0002	0.000773	0.0189	0.0030	0.000788	0.0199	0.00520	0.0061	0.1735	0.014	2	2014 02 23	01:23
273.146	2.32150	27.1026	0.0005	0.000780	0.0188	0.0030	0.000796	0.0199	0.00520	0.0061	0.1729	0.016	2	2014 02 23	01:48
273.147	2.32420	27.1402	0.0002	0.000771	0.0188	0.0030	0.000786	0.0198	0.00520	0.0061	0.1722	0.018	2	2014 02 23	02:01
273.147	2.32688	27.1749	0.0004	0.000762	0.0188	0.0030	0.000778	0.0199	0.00520	0.0061	0.1717	0.019	2	2014 02 23	02:14
273.145	2.32954	27.2099	0.0005	0.000757	0.0188	0.0030	0.000773	0.0198	0.00520	0.0061	0.1708	0.019	2	2014 02 23	02:27
273.143	2.33221	27.2469	0.0002	0.000762	0.0184	0.0030	0.000778	0.0195	0.00520	0.0061	0.1689	0.020	2	2014 02 23	02:40
273.144	2.33488	27.2831	0.0005	0.000768	0.0186	0.0030	0.000784	0.0196	0.00520	0.0062	0.1701	0.017	2	2014 02 23	02:53
273.146	2.33755	27.3197	0.0006	0.000757	0.0182	0.0031	0.000773	0.0192	0.00520	0.0062	0.1669	0.016	2	2014 02 23	03:06
273.148	2.34020	27.3538	0.0001	0.000753	0.0181	0.0030	0.000769	0.0191	0.00520	0.0062	0.1658	0.014	2	2014 02 23	03:19
273.145	2.34504	27.4194	0.0007	0.000743	0.0185	0.0031	0.000759	0.0195	0.00520	0.0062	0.1658	0.011	2	2014 02 23	03:43
273.143	2.34764	27.4570	0.0002	0.000746	0.0182	0.0030	0.000763	0.0192	0.00520	0.0062	0.1653	0.016	2	2014 02 23	03:56
273.144	2.35027	27.4918	0.0004	0.000757	0.0181	0.0030	0.000773	0.0192	0.00520	0.0062	0.1655	0.018	2	2014 02 23	04:09
273.146	2.35290	27.5277	0.0007	0.000743	0.0178	0.0031	0.000759	0.0188	0.00520	0.0062	0.1628	0.020	2	2014 02 23	04:22
273.147	2.35549	27.5632	0.0003	0.000742	0.0178	0.0030	0.000759	0.0188	0.00520	0.0062	0.1623	0.025	2	2014 02 23	04:35
273.147	2.35807	27.5977	0.0004	0.000734	0.0180	0.0030	0.000751	0.0190	0.00520	0.0062	0.1629	0.026	2	2014 02 23	04:48
273.145	2.36064	27.6312	0.0006	0.000736	0.0177	0.0031	0.000753	0.0188	0.00520	0.0062	0.1614	0.024	2	2014 02 23	05:01
273.144	2.36320	27.6659	0.0001	0.000735	0.0177	0.0030	0.000751	0.0187	0.00520	0.0062	0.1607	0.028	2	2014 02 23	05:14
273.146	2.36795	27.7305	0.0005	0.000734	0.0170	0.0030	0.000750	0.0180	0.00520	0.0063	0.1564	0.031	2	2014 02 23	05:38
273.147	2.37049	27.7632	0.0001	0.000719	0.0176	0.0030	0.000736	0.0186	0.00520	0.0063	0.1592	0.029	2	2014 02 23	05:51
273.147	2.37320	27.8004	0.0005	0.000712	0.0167	0.0030	0.000729	0.0178	0.00520	0.0063	0.1536	0.038	2	2014 02 23	06:05
273.144	2.37571	27.8339	0.0005	0.000714	0.0164	0.0030	0.000731	0.0174	0.00520	0.0063	0.1516	0.033	2	2014 02 23	06:18
273.144	2.37819	27.8661	0.0002	0.000709	0.0160	0.0030	0.000727	0.0171	0.00520	0.0063	0.1489	0.041	2	2014 02 23	06:31
273.145	2.38068	27.8979	0.0005	0.000711	0.0154	0.0030	0.000728	0.0164	0.00520	0.0063	0.1450	0.049	2	2014 02 23	06:44
273.146	2.38318	27.9278	0.0004	0.000711	0.0148	0.0030	0.000728	0.0158	0.00520	0.0063	0.1415	0.059	2	2014 02 23	06:57
273.146	2.38766	27.9813	0.0003	0.000686	0.0123	0.0030	0.000704	0.0133	0.00520	0.0063	0.1260	0.069	2	2014 02 23	07:21
273.145	2.39024	28.0098	0.0004	0.000678	0.0109	0.0030	0.000696	0.0119	0.00520	0.0063	0.1180	0.078	2	2014 02 23	07:35
273.144	2.39260	28.0337	0.0001	0.000673	0.0085	0.0030	0.000691	0.0095	0.00520	0.0063	0.1061	0.112	2	2014 02 23	07:48
273.145	2.39494	28.0527	0.0003	0.000658	0.0060	0.0030	0.000677	0.0071	0.00520	0.0063	0.0950	0.126	2	2014 02 23	08:01
273.146	2.39724	28.0719	0.0005	0.000660	0.0046	0.0030	0.000678	0.0056	0.00520	0.0063	0.0900	0.143	2	2014 02 23	08:14
273.147	2.39985	28.0908	0.0002	0.000637	0.0029	0.0030	0.000656	0.0040	0.00520	0.0063	0.0836	0.160	2	2014 02 23	08:29
273.146	2.40206	28.1003	0.0005	0.000622	0.0023	0.0030	0.000642	0.0035	0.00520	0.0063	0.0813	0.135	2	2014 02 23	08:42
273.144	2.40606	28.1226	0.0002	0.000622	0.0017	0.0030	0.000642	0.0028	0.00520	0.0063	0.0801	0.169	2	2014 02 23	09:06
273.144	2.40824	28.1298	0.0004	0.000620	0.0023	0.0030	0.000639	0.0034	0.00520	0.0063	0.0810	0.198	2	2014 02 23	09:19
273.146	2.41042	28.1409	0.0004	0.000620	0.0021	0.0030	0.000639	0.0032	0.00520	0.0063	0.0806	0.210	2	2014 02 23	09:32
273.147	2.41257	28.1465	0.0002	0.000610	0.0030	0.0030	0.000630	0.0041	0.00520	0.0063	0.0820	0.208	2	2014 02 23	09:45
273.146	2.41486	28.1463	0.0004	0.000601	0.0021	0.0030	0.000621	0.0032	0.00520	0.0063	0.0792	0.196	2	2014 02 23	09:59
273.145	2.41696	28.1544	0.0002	0.000598	0.0028	0.0030	0.000618	0.0039	0.00520	0.0064	0.0805	0.172	2	2014 02 23	10:12
273.144	2.41906	28.1489	0.0000	0.000607	0.0048	0.0030	0.000627	0.0059	0.00520	0.0063	0.0871	0.139	2	2014 02 23	10:25
273.146	2.42299	28.1612	0.0003	0.000603	0.0052	0.0030	0.000624	0.0062	0.00520	0.0064	0.0879	0.143	2	2014 02 23	10:49
273.147	2.42508	28.1467	0.0002	0.000595	0.0023	0.0030	0.000615	0.0035	0.00520	0.0063	0.0793	0.098	2	2014 02 23	11:02
273.146	2.42715	28.1875	0.0003	0.000587	0.0015	0.0030	0.000608	0.0027	0.00520	0.0064	0.0771	0.130	2	2014 02 23	11:15
273.145	2.42936	28.1980	0.0003	0.000586	0.0022	0.0030	0.000607	0.0034	0.00520	0.0064	0.0784	0.127	2	2014 02 23	11:29
273.144	2.43143	28.1931	0.0001	0.000596	0.0028	0.0030	0.000617	0.0039	0.00520	0.0064	0.0804	-0.003	2	2014 02 23	11:42
273.145	2.43352	28.1857	0.0003	0.000597	0.0029	0.0030	0.000618	0.0040	0.00520	0.0064	0.0806	0.018	2	2014 02 23	11:55
273.146	2.43560	28.1986	0.0003	0.000592	0.0025	0.0030	0.000613	0.0037	0.00520	0.0064	0.0795	-0.011	2	2014 02 23	12:08
273.146	2.43937	28.2162	0.0004	0.000579	0.0026	0.0030	0.000600	0.0037	0.00520	0.0064	0.0787	0.029	2	2014 02 23	12:32
273.145	2.44139	28.2192	0.0003	0.000577	0.0051	0.0030	0.000598	0.0062	0.00520	0.0064	0.0860	0.042	2	2014 02 23	12:45
273.144	2.44358	28.2203	0.0001	0.000584	0.0016	0.0030	0.000605	0.0028	0.00520	0.0064	0.0770	0.038	2	2014 02 23	12:59
273.145	2.44564	28.2251	0.0005	0.000587	0.0034	0.0030	0.000608	0.0045	0.00520	0.0064	0.0813	0.042	2	2014 02 23	13:12
273.146	2.44769	28.2412	0.0003	0.000577	0.0022	0.0030	0.000598	0.0033	0.00520	0.0064	0.0776	0.055	2	2014 02 23	13:25
273.147	2.44971	28.2501	0.0001	0.000572	0.0045	0.0030	0.000594	0.0056	0.00520	0.0064	0.0834	0.057	2	2014 02 23	13:38

T	p	rho	sigma_T	sigma_p	sigma_rho	u(T)	u(p)	u(rho)	u(MW)	u[rho(x)]	U_c/%	m_sorb	p_trans	date	time
(K)	(MPa)	(kg.m−3)	(K)	(MPa)	(kg.m−3)	(K)	(MPa)	(kg.m−3)	(g/mol)	(kg.m−3)	(k = 2)	(mg)			
# test: mp_1402m.dat (continued)															
273.146	2.45171	28.2439	0.0003	0.000568	0.0054	0.0030	0.000590	0.0065	0.00520	0.0064	0.0864	5.003	2	2014 02 23	13:51
273.144	2.45540	28.2937	0.0001	0.000574	0.0038	0.0030	0.000595	0.0049	0.00520	0.0064	0.0812	6.150	2	2014 02 23	14:15
273.145	2.45758	28.2877	0.0003	0.000584	0.0043	0.0030	0.000605	0.0054	0.00520	0.0064	0.0837	6.504	2	2014 02 23	14:29
273.146	2.45961	28.3323	0.0004	0.000575	0.0025	0.0030	0.000596	0.0036	0.00520	0.0064	0.0780	7.037	2	2014 02 23	14:42
273.146	2.46160	28.2972	0.0002	0.000563	0.0051	0.0030	0.000585	0.0061	0.00520	0.0064	0.0848	7.107	2	2014 02 23	14:55
273.146	2.46357	28.3017	0.0004	0.000562	0.0046	0.0030	0.000583	0.0057	0.00520	0.0064	0.0831	7.662	2	2014 02 23	15:08
273.144	2.46554	28.3148	0.0002	0.000568	0.0045	0.0030	0.000590	0.0056	0.00520	0.0064	0.0831	7.621	2	2014 02 23	15:21
273.144	2.46753	28.3253	0.0002	0.000571	0.0033	0.0030	0.000592	0.0044	0.00520	0.0064	0.0796	8.787	2	2014 02 23	15:34
273.147	2.47139	28.3450	0.0004	0.000567	0.0012	0.0030	0.000588	0.0024	0.00520	0.0064	0.0751	9.828	2	2014 02 23	15:59
273.147	2.47336	28.3402	0.0001	0.000557	0.0050	0.0030	0.000579	0.0061	0.00520	0.0064	0.0842	10.240	2	2014 02 23	16:12
273.146	2.47531	28.3710	0.0004	0.000551	0.0067	0.0030	0.000573	0.0077	0.00520	0.0064	0.0901	10.572	2	2014 02 23	16:25
273.144	2.47725	28.3821	0.0003	0.000561	0.0045	0.0030	0.000582	0.0056	0.00520	0.0064	0.0826	11.012	2	2014 02 23	16:38
273.144	2.47921	28.3972	0.0003	0.000566	0.0021	0.0030	0.000587	0.0033	0.00520	0.0064	0.0765	11.685	2	2014 02 23	16:51
273.145	2.48118	28.4026	0.0006	0.000566	0.0040	0.0031	0.000588	0.0050	0.00520	0.0064	0.0811	12.142	2	2014 02 23	17:04
273.147	2.48315	28.4063	0.0003	0.000553	0.0061	0.0030	0.000575	0.0072	0.00520	0.0064	0.0880	12.282	2	2014 02 23	17:17
273.145	2.48682	28.4099	0.0005	0.000539	0.0049	0.0030	0.000562	0.0060	0.00520	0.0064	0.0825	13.281	2	2014 02 23	17:42
273.144	2.48872	28.4345	0.0003	0.000545	0.0033	0.0030	0.000567	0.0044	0.00520	0.0064	0.0777	13.973	2	2014 02 23	17:55
273.144	2.49064	28.4626	0.0003	0.000553	0.0007	0.0030	0.000575	0.0021	0.00520	0.0064	0.0734	14.702	2	2014 02 23	18:08
273.145	2.49259	28.4577	0.0006	0.000557	0.0017	0.0031	0.000579	0.0028	0.00520	0.0064	0.0750	15.479	2	2014 02 23	18:21
273.147	2.49452	28.4413	0.0003	0.000551	0.0024	0.0030	0.000573	0.0035	0.00520	0.0064	0.0759	15.266	2	2014 02 23	18:34

Table S6. Experimental (p, rho, T, x) data and detailed uncertainty information for isotherms measured on the (0.74977 methane + 0.25023 propane) mixture (continued)

test: mp_1402o.dat (continuously increase pressure near dew line)
chiS [specific magnetic susceptibility] = -0.1237E-07 [m3/kg]

T (K)	p (MPa)	rho (kg.m-3)	sigma_T (K)	sigma_p (MPa)	sigma_rho (kg.m-3)	u(T) (K)	u(p) (MPa)	u(rho) (kg.m-3)	u(MW) (g/mol)	u[rho(x)] (kg.m-3)	U_c/% (k = 2)	m_sorb (mg)	p_trans	date	time
273.145	2.18427	25.2658	0.0001	0.000530	0.0128	0.0030	0.000553	0.0138	0.00520	0.0057	0.1322	-0.002	2	2014 02 24	16:30
273.147	2.18612	25.2901	0.0007	0.000530	0.0127	0.0031	0.000553	0.0137	0.00520	0.0057	0.1309	0.000	2	2014 02 24	16:43
273.149	2.18795	25.3156	0.0006	0.000513	0.0123	0.0031	0.000536	0.0133	0.00520	0.0057	0.1276	0.001	2	2014 02 24	16:56
273.151	2.26123	26.2933	0.0002	0.001330	0.0325	0.0030	0.001340	0.0335	0.00520	0.0059	0.2929	-0.001	2	2014 02 24	17:12
273.149	2.26578	26.3549	0.0008	0.001215	0.0295	0.0031	0.001225	0.0305	0.00520	0.0059	0.2675	-0.013	2	2014 02 24	17:25
273.147	2.26934	26.4055	0.0006	0.000917	0.0224	0.0031	0.000931	0.0234	0.00520	0.0060	0.2675	0.005	2	2014 02 24	17:38
273.145	2.27247	26.4474	0.0001	0.000885	0.0213	0.0030	0.000898	0.0223	0.00520	0.0060	0.2675	0.006	2	2014 02 24	17:51
273.148	2.27833	26.5255	0.0006	0.000848	0.0203	0.0031	0.000862	0.0213	0.00520	0.0060	0.2675	0.007	2	2014 02 24	18:16
273.149	2.28127	26.5644	0.0001	0.000827	0.0200	0.0030	0.000842	0.0210	0.00520	0.0060	0.2675	0.007	2	2014 02 24	18:29
273.148	2.28413	26.6046	0.0005	0.000820	0.0200	0.0030	0.000835	0.0210	0.00520	0.0060	0.2675	0.009	2	2014 02 24	18:42
273.146	2.28698	26.6430	0.0005	0.000801	0.0193	0.0030	0.000816	0.0203	0.00520	0.0060	0.2675	0.010	2	2014 02 24	18:55
273.145	2.28981	26.6816	0.0002	0.000801	0.0193	0.0030	0.000817	0.0203	0.00520	0.0060	0.2675	0.007	2	2014 02 24	19:08
273.145	2.29282	26.7209	0.0003	0.000797	0.0193	0.0030	0.000812	0.0203	0.00520	0.0060	0.2675	0.008	2	2014 02 24	19:22
273.146	2.29559	26.7572	0.0003	0.000792	0.0194	0.0030	0.000807	0.0204	0.00520	0.0060	0.2675	0.008	2	2014 02 24	19:35
273.146	2.30064	26.8278	0.0004	0.000771	0.0191	0.0030	0.000787	0.0201	0.00520	0.0061	0.2675	0.011	2	2014 02 24	19:59
273.144	2.30334	26.8645	0.0003	0.000764	0.0187	0.0030	0.000780	0.0197	0.00520	0.0061	0.2675	0.008	2	2014 02 24	20:12
273.144	2.30602	26.9020	0.0001	0.000763	0.0187	0.0030	0.000779	0.0197	0.00520	0.0061	0.1721	0.013	2	2014 02 24	20:25
273.144	2.30870	26.9366	0.0003	0.000764	0.0185	0.0030	0.000780	0.0195	0.00520	0.0061	0.1721	0.008	2	2014 02 24	20:38
273.145	2.31157	26.9747	0.0003	0.000763	0.0186	0.0030	0.000779	0.0196	0.00520	0.0061	0.1721	0.010	2	2014 02 24	20:52
273.146	2.31421	27.0112	0.0002	0.000748	0.0184	0.0030	0.000764	0.0194	0.00520	0.0061	0.1721	0.012	2	2014 02 24	21:05
273.145	2.31681	27.0465	0.0004	0.000744	0.0185	0.0030	0.000760	0.0195	0.00520	0.0061	0.1721	0.008	2	2014 02 24	21:18
273.143	2.32161	27.1119	0.0001	0.000734	0.0181	0.0030	0.000751	0.0191	0.00520	0.0061	0.1721	0.010	2	2014 02 24	21:42
273.144	2.32420	27.1473	0.0003	0.000733	0.0180	0.0030	0.000749	0.0190	0.00520	0.0061	0.1721	0.010	2	2014 02 24	21:55
273.145	2.32677	27.1819	0.0004	0.000733	0.0179	0.0030	0.000750	0.0189	0.00520	0.0061	0.1721	0.011	2	2014 02 24	22:08
273.146	2.32952	27.2180	0.0002	0.000719	0.0179	0.0030	0.000736	0.0189	0.00520	0.0061	0.1721	0.011	2	2014 02 24	22:22
273.145	2.33206	27.2517	0.0004	0.000718	0.0178	0.0030	0.000735	0.0188	0.00520	0.0061	0.1721	0.010	2	2014 02 24	22:35
273.143	2.33458	27.2872	0.0003	0.000713	0.0175	0.0030	0.000731	0.0185	0.00520	0.0062	0.1721	0.011	2	2014 02 24	22:48
273.143	2.33710	27.3223	0.0001	0.000713	0.0175	0.0030	0.000730	0.0185	0.00520	0.0062	0.1721	0.012	2	2014 02 24	23:01
273.146	2.34174	27.3836	0.0005	0.000716	0.0176	0.0030	0.000733	0.0186	0.00520	0.0062	0.1607	0.014	2	2014 02 24	23:25
273.146	2.34423	27.4175	0.0000	0.000707	0.0174	0.0030	0.000724	0.0185	0.00520	0.0062	0.1594	0.014	2	2014 02 24	23:38
273.146	2.34688	27.4543	0.0005	0.000696	0.0173	0.0030	0.000713	0.0183	0.00520	0.0062	0.1578	0.016	2	2014 02 24	23:52
273.144	2.34933	27.4886	0.0004	0.000699	0.0173	0.0030	0.000717	0.0183	0.00520	0.0062	0.1576	0.016	2	2014 02 25	00:05
273.143	2.35179	27.5201	0.0001	0.000695	0.0168	0.0030	0.000712	0.0178	0.00520	0.0062	0.1545	0.018	2	2014 02 25	00:18
273.143	2.35422	27.5542	0.0003	0.000697	0.0170	0.0030	0.000715	0.0180	0.00520	0.0062	0.1553	0.020	2	2014 02 25	00:31
273.145	2.35667	27.5873	0.0005	0.000695	0.0166	0.0030	0.000713	0.0176	0.00520	0.0062	0.1530	0.020	2	2014 02 25	00:44
273.146	2.36114	27.6467	0.0004	0.000681	0.0168	0.0030	0.000699	0.0178	0.00520	0.0062	0.1530	0.026	2	2014 02 25	01:08
273.144	2.36353	27.6797	0.0005	0.000679	0.0163	0.0030	0.000697	0.0173	0.00520	0.0062	0.1501	0.027	2	2014 02 25	01:21
273.143	2.36609	27.7125	0.0001	0.000678	0.0161	0.0030	0.000696	0.0172	0.00520	0.0063	0.1488	0.030	2	2014 02 25	01:35
273.144	2.36847	27.7465	0.0004	0.000677	0.0160	0.0030	0.000695	0.0171	0.00520	0.0063	0.1479	0.034	2	2014 02 25	01:48
273.145	2.37085	27.7765	0.0005	0.000681	0.0159	0.0030	0.000699	0.0169	0.00520	0.0063	0.1473	0.040	2	2014 02 25	02:01
273.146	2.37321	27.8086	0.0000	0.000668	0.0157	0.0030	0.000687	0.0167	0.00520	0.0063	0.1452	0.045	2	2014 02 25	02:14
273.145	2.37554	27.8397	0.0003	0.000662	0.0154	0.0030	0.000680	0.0164	0.00520	0.0063	0.1429	0.048	2	2014 02 25	02:27
273.143	2.37979	27.8959	0.0001	0.000656	0.0139	0.0030	0.000675	0.0149	0.00520	0.0063	0.1339	0.062	2	2014 02 25	02:51
273.144	2.38227	27.9254	0.0004	0.000662	0.0137	0.0030	0.000680	0.0148	0.00520	0.0063	0.1331	0.078	2	2014 02 25	03:05
273.145	2.38456	27.9520	0.0005	0.000648	0.0127	0.0030	0.000667	0.0137	0.00520	0.0063	0.1263	0.091	2	2014 02 25	03:18
273.146	2.38681	27.9786	0.0001	0.000637	0.0113	0.0030	0.000656	0.0123	0.00520	0.0063	0.1182	0.104	2	2014 02 25	03:31
273.145	2.38901	28.0035	0.0003	0.000624	0.0098	0.0030	0.000643	0.0108	0.00520	0.0063	0.1095	0.126	2	2014 02 25	03:44
273.144	2.39118	28.0220	0.0002	0.000615	0.0078	0.0030	0.000635	0.0088	0.00520	0.0063	0.0996	0.142	2	2014 02 25	03:57
273.144	2.39332	28.0383	0.0002	0.000607	0.0005	0.0030	0.000627	0.0019	0.00520	0.0063	0.0777	0.117	2	2014 02 25	04:10
273.146	2.39740	28.0658	0.0003	0.000596	0.0009	0.0030	0.000617	0.0022	0.00520	0.0063	0.0772	0.147	2	2014 02 25	04:35

T (K)	p (MPa)	rho (kg.m-3)	sigma_T (K)	sigma_p (MPa)	sigma_rho (kg.m-3)	u(T) (K)	u(p) (MPa)	u(rho) (kg.m-3)	u(MW) (g/mol)	u[rho(x)] (kg.m-3)	U_c/% (k = 2)	m_sorb (mg)	p_trans	date	time
# test: mp_1402o.dat (continued)															
273.146	2.39947	28.0869	0.0002	0.000582	0.0029	0.0030	0.000603	0.0040	0.00520	0.0063	0.0797	0.254	2	2014 02 25	04:48
273.145	2.40149	28.0994	0.0004	0.000567	0.0019	0.0030	0.000589	0.0031	0.00520	0.0063	0.0766	0.271	2	2014 02 25	05:01
273.143	2.40350	28.1087	0.0002	0.000570	0.0006	0.0030	0.000591	0.0020	0.00520	0.0063	0.0750	0.295	2	2014 02 25	05:14
273.143	2.40551	28.1165	0.0002	0.000577	0.0010	0.0030	0.000599	0.0023	0.00520	0.0063	0.0758	0.324	2	2014 02 25	05:27
273.145	2.40754	28.1237	0.0006	0.000579	0.0023	0.0031	0.000600	0.0035	0.00520	0.0063	0.0782	0.336	2	2014 02 25	05:40
273.146	2.40954	28.1339	0.0002	0.000569	0.0005	0.0030	0.000590	0.0019	0.00520	0.0063	0.0747	0.354	2	2014 02 25	05:53
273.144	2.41326	28.1484	0.0007	0.000551	0.0013	0.0031	0.000573	0.0025	0.00520	0.0063	0.0743	0.377	2	2014 02 25	06:18
273.143	2.41521	28.1518	0.0001	0.000562	0.0026	0.0030	0.000584	0.0037	0.00520	0.0064	0.0776	0.396	2	2014 02 25	06:31
273.144	2.41719	28.1543	0.0005	0.000565	0.0031	0.0030	0.000587	0.0042	0.00520	0.0064	0.0789	0.402	2	2014 02 25	06:44
273.145	2.41916	28.1652	0.0005	0.000560	0.0028	0.0030	0.000582	0.0039	0.00520	0.0064	0.0779	0.413	2	2014 02 25	06:57
273.146	2.42110	28.1635	0.0001	0.000546	0.0010	0.0030	0.000568	0.0023	0.00520	0.0064	0.0736	0.426	2	2014 02 25	07:10
273.145	2.42300	28.1810	0.0005	0.000539	0.0042	0.0030	0.000562	0.0053	0.00520	0.0064	0.0805	0.448	2	2014 02 25	07:23
273.144	2.42488	28.1809	0.0004	0.000544	0.0029	0.0030	0.000566	0.0040	0.00520	0.0064	0.0770	0.468	2	2014 02 25	07:36
273.143	2.42681	28.1658	0.0002	0.000551	0.0030	0.0030	0.000573	0.0041	0.00520	0.0064	0.0779	0.493	2	2014 02 25	07:49
273.146	2.43038	28.1954	0.0004	0.000542	0.0028	0.0030	0.000565	0.0039	0.00520	0.0064	0.0767	5.052	2	2014 02 25	08:13
273.146	2.43227	28.2042	0.0001	0.000534	0.0014	0.0030	0.000557	0.0026	0.00520	0.0064	0.0733	5.515	2	2014 02 25	08:26
273.145	2.43414	28.2075	0.0005	0.000525	0.0028	0.0030	0.000548	0.0039	0.00520	0.0064	0.0753	5.999	2	2014 02 25	08:39
273.143	2.43599	28.2169	0.0003	0.000527	0.0027	0.0030	0.000551	0.0038	0.00520	0.0064	0.0754	6.158	2	2014 02 25	08:52
273.143	2.43788	28.2236	0.0002	0.000541	0.0012	0.0030	0.000564	0.0024	0.00520	0.0064	0.0734	6.759	2	2014 02 25	09:05
273.144	2.43979	28.2331	0.0006	0.000551	0.0020	0.0031	0.000573	0.0032	0.00520	0.0064	0.0754	6.998	2	2014 02 25	09:18
273.146	2.44170	28.2321	0.0005	0.000537	0.0018	0.0030	0.000559	0.0030	0.00520	0.0064	0.0740	7.374	2	2014 02 25	09:31
273.146	2.44355	28.2493	0.0002	0.000523	0.0025	0.0030	0.000546	0.0037	0.00520	0.0064	0.0746	7.672	2	2014 02 25	09:44
273.144	2.44692	28.2429	0.0003	0.000517	0.0026	0.0030	0.000540	0.0038	0.00520	0.0064	0.0744	8.337	2	2014 02 25	10:08
273.143	2.44890	28.2691	0.0002	0.000527	0.0091	0.0030	0.000550	0.0101	0.00520	0.0064	0.1002	8.707	2	2014 02 25	10:22
273.144	2.45077	28.2784	0.0004	0.000533	0.0115	0.0030	0.000556	0.0125	0.00520	0.0064	0.1133	9.058	2	2014 02 25	10:35
273.146	2.45263	28.2788	0.0004	0.000525	0.0020	0.0030	0.000549	0.0032	0.00520	0.0064	0.0737	9.178	2	2014 02 25	10:48
273.146	2.45444	28.3064	0.0002	0.000509	0.0031	0.0030	0.000533	0.0042	0.00520	0.0064	0.0751	10.204	2	2014 02 25	11:01
273.145	2.45622	28.2971	0.0006	0.000505	0.0048	0.0031	0.000529	0.0059	0.00520	0.0064	0.0803	10.194	2	2014 02 25	11:14
273.143	2.45800	28.3044	0.0003	0.000511	0.0032	0.0030	0.000535	0.0043	0.00520	0.0064	0.0755	9.765	2	2014 02 25	11:27
273.143	2.45981	28.3210	0.0003	0.000523	0.0028	0.0030	0.000546	0.0039	0.00520	0.0064	0.0752	10.836	2	2014 02 25	11:40
273.146	2.46333	28.3257	0.0003	0.000509	0.0024	0.0030	0.000533	0.0035	0.00520	0.0064	0.0733	11.583	2	2014 02 25	12:05
273.146	2.46510	28.3397	0.0002	0.000500	0.0045	0.0030	0.000524	0.0055	0.00520	0.0064	0.0786	12.309	2	2014 02 25	12:18
273.145	2.46684	28.3384	0.0007	0.000496	0.0060	0.0031	0.000521	0.0070	0.00520	0.0064	0.0841	12.684	2	2014 02 25	12:31
273.143	2.46860	28.3296	0.0003	0.000503	0.0049	0.0030	0.000527	0.0060	0.00520	0.0064	0.0806	13.582	2	2014 02 25	12:44
273.143	2.47038	28.3545	0.0003	0.000519	0.0014	0.0030	0.000542	0.0026	0.00520	0.0064	0.0720	13.493	2	2014 02 25	12:57
273.145	2.47246	28.3620	0.0005	0.000517	0.0028	0.0030	0.000540	0.0040	0.00520	0.0064	0.0748	13.875	2	2014 02 25	13:12
273.146	2.47573	28.3720	0.0003	0.000494	0.0042	0.0030	0.000518	0.0052	0.00520	0.0064	0.0772	14.996	2	2014 02 25	13:36
273.144	2.47744	28.3871	0.0007	0.000480	0.0076	0.0031	0.000505	0.0086	0.00520	0.0064	0.0902	14.962	2	2014 02 25	13:49
273.142	2.47916	28.4089	0.0003	0.000499	0.0043	0.0030	0.000523	0.0054	0.00520	0.0064	0.0781	15.783	2	2014 02 25	14:02
273.142	2.48093	28.4109	0.0003	0.000510	0.0033	0.0030	0.000534	0.0044	0.00520	0.0064	0.0756	15.742	2	2014 02 25	14:15

References for Supplementary Information
(numbering continues from main paper)

12. Gernert, J. & Span, R. EOS-CG: A Helmholtz energy mixture model for humid gases and CCS mixtures. *J. Chem. Thermodynamics* **93**, 274-293 (2016).
13. Kunz, O. & Wagner, W. The GERG-2008 wide-range equation of state for natural gases and other mixtures: An expansion of GERG-2004. *J. Chem. Engr. Data* **57**, 3032-3091 (2012).
20. McLinden, M. O. & Richter, M. Application of a two-sinker densimeter for phase-equilibrium measurements: A new technique for the detection of dew points and measurements on the (methane + propane) System. *J. Chem. Thermodyn.* **99**, 105-115, doi:10.1016/j.jct.2016.03.035 (2016).
22. Ahmad, M., Gernert, J. & Wilbers, E. Effect of impurities in captured CO_2 on liquid–vapor equilibrium. *Fluid Phase Equilibria* **363**, 149-155, doi:10.1016/j.fluid.2013.11.009 (2014).
23. Coquelet, C. *et al.* Isothermal P, x, y data for the argon + carbon dioxide system at six temperatures from 233.32 to 299.21 K and pressures up to 14 MPa. *Fluid Phase Equilibria* **273**, 38-43 (2008).
24. Kaminishi, G., Arai, Y., Saito, S. & Maeda, S. Vapor-liquid equilibria for binary and ternary systems containing carbon dioxide. *J. Chem. Eng. Japan* **1**, 109-116 (1968).
25. Köpke, D. & Eggers, R. Experimentelle Untersuchungen des Phasengleichgewichts des Systems Kohlendioxid/Argon und Literaturauswertung zum System Kohlendioxid/Schwefeldioxid. *Chemie Ingenieur Technik* **79**, 1235-1239 (2007).
26. Sarashina, E., Arai, Y. & Saito, S. The P-V-T-X relation for the carbon dioxide-argon system. *J. Chem. Eng. Japan* **4**, 379-381 (1971).
27. Tsankova, G., Richter, M., Madigan, A., Stanwix, P.L., May, E.F., Span, R., Characterisation of a microwave re-entrant cavity resonator for phase-equilibrium measurements and new dew-point data for a (0.25 argon + 0.75 carbon dioxide) mixture. *J. Chem. Thermodyn.* **101**, 395-404, doi:10.1016/j.jct.2016.06.005 (2016).
28. Akers, W. W.; Kelley, R. E.; Lipscomb, T. G., Carbon dioxide-propane system, low-temperature phase equilibria. *Ind. Eng. Chem.* **1954,** 46, 2535.
29. Calado, J. C. G.; Garcia, G. A.; Staveley, L. A. K., Thermodynamics of the liquid system methane + propane. *J. Chem. Soc., Faraday Trans. 1* **1974**, 70, 1445-1451.
30. Cheung, H.; Wang, D. I., Solubility of volatile gases in hydrocarbon solvents at cryogenic temperatures. *Ind. Eng. Chem. Fundam.* **1964**, 3, 355-361.
31. Cutler, A. J. B.; Morrison, J. A., Excess Thermodynamic functions for liquid mixtures of methane + propane. *Trans. Faraday Soc.* **1965**, 61, 429-442.
32. Joffe, J., Vapor-liquid equilibria by the pseudocritical method. *Ind. Eng. Chem. Fundam.* **1976**, 15, 298-304.
33. Kalra, H.; Robinson, D. B., An apparatus for the simultaneous measurement of equilibrium phase. *Cryogenics* **1975**, 15, 409-412.

34. Kandil, M. E.; Marsh, K. N.; Goodwin, A. R. H., A re-entrant resonator for the measurement of phase boundaries: dew points for ($0.4026CH_4 + 0.5974C_3H_8$). *J. Chem. Thermodynamics* **2005**, 37, 684-691.

35. May, E. F.; Guo, J. Y.; Oakley, J. H.; Hughes, T. J.; Graham, B. F.; Marsh, K. N.; Huang, S. H., Reference quality vapor−liquid equilibrium data for the binary systems methane + ethane, + propane, + butane, and + 2−methylpropane, at temperatures from (203 to 273) K and pressures to 9 MPa. *J. Chem. Engr. Data* **2015,** 60, 3606−3620.

36. McLinden, M. O. & Richter, M. Application of a two-sinker densimeter for phase-equilibrium measurements: A new technique for the detection of dew points and measurements on the (methane + propane) System. J. Chem. Thermodyn. 99, 105-115, doi:10.1016/j.jct.2016.03.035 (2016).

37. Poon, D. P. L.; Lu, B. C.-Y., Phase equilibria for systems containing nitrogen, methane and propane. *Adv. Cryo. Eng.* **1974**, 19, 292-299.

38. Powers, J. E.; Manker, E. A.; Mather, A. E.; Yesavage, V. F. In Vapor-liquid equilibrium data for the methane-propane system, AIChE 65th National Meeting, May 4-7, 1969.

39. Price, R. A.; Kobayashi, R., Low temperature vapor-liquid equilibrium in light hydrocarbon mixtures: Methane–ethane–propane System. *J. Chem. Eng. Data* **1959**, 4, 40-52.

40. Price, A.R. Low temperature vapor-liquid equilibrium in light hydrocarbon mixtures: methane-ethane-propane system, Rice University, PhD Dissertation, 1957.

41. Reamer, H. H.; Sage, B. H.; Lacey, W. N., Phase equilibria in hydrocarbon systems, volumetric and phase behavior of the methane-propane system. *Ind. Eng. Chem.* **1950,** *42*, 534-539.

42. Roof, J. G.; Baron, J. D., Critical loci of binary mixtures of propane with methane, carbon dioxide, and nitrogen. *J. Chem. Eng. Data* **1967**, 12, 292-293.

43. Sage, B. H.; Lacey, W. N.; Schaafsma, J. G., Phase equilibria in hydrocarbon systems, II. Methane-propane system. *Ind. Eng. Chem.* **1934**, 26, 214-217.

44. Skripka, V. G.; Nikitina, I. E.; Zhdanovich, L. A.; Sirotin, A. G.; Benyaminovich, O. A., Liquid-vapor phase equilibrium at low temperatures in binary systems, components produced from natural gas. *Gazov Promst.* **1970**, 15 (12), 35-36.

45. Stoeckli, H. F.; Staveley, L. A. K., Low Temperature Thermodynamics. The Excess Gibbs Function and the Volumes of Mixing for the System Methane + Propane at 90.68K. *Helv. Chim. Acta* **1970**, 53, 1961-1964.

46. Webster, L. A.; Kidnay, A. J., Vapor-liquid equilibria for the methane-propane-carbon dioxide systems at 230 K and 270 K. *J. Chem. Eng. Data* **2001**, 46, 759-764.

47. Wichterle, I.; Kobayashi, R., Vapor-Liquid Equilibrium of Methane-Propane System at Low Temperatures and High Pressures. *J. Chem. Eng. Data* **1972**, 17, 4-9.

48. Wiese, H. C.; Jacobs, J.; Sage, B. H., Phase equilibria in the hydrocarbon systems. Phase behavior in the methane-propane-n-butane System. *J. Chem. Eng. Data* **1970**, *15*, 82-91.

49. Wilson, G. M., Vapor-liquid equilibria of nitrogen, methane, ethane and propane binary mixtures at LNG temperatures from total pressure measurements. *Adv. Cryo. Eng.* **1975**, 20, 164-171.
